# Iron(II) and Copper(I) Control the Total Regioselectivity in the Hydrobromination of Alkenes

**DOI:** 10.1021/acs.orglett.1c02186

**Published:** 2021-07-28

**Authors:** Daniel A. Cruz, Victoria Sinka, Pedro de Armas, Hugo Sebastian Steingruber, Israel Fernández, Víctor S. Martín, Pedro O. Miranda, Juan I. Padrón

**Affiliations:** † Molecular Sciences Department, Instituto de Productos Naturales y Agrobiología, Consejo Superior de Investigaciones Científicas (IPNA-CSIC), Avenida Astrofísico Francisco Sánchez 3, 38206 La Laguna, Tenerife, Islas Canarias, Spain; ‡ “Síntesis Orgánica Sostenible, Unidad Asociada al CSIC”, Departamento de Química Orgánica, Instituto Universitario de Bio-Orgánica “Antonio González” (CIBICAN), Universidad de La Laguna, Avenida Francisco Sánchez 2, 38206 La Laguna, Tenerife, Islas Canarias, Spain; § Departamento de Química Orgánica I y Centro de Innovación en Química Avanzada (ORFEO-CINQA), Facultad de Ciencias Químicas, Universidad Complutense de Madrid, 28040 Madrid, Spain

## Abstract

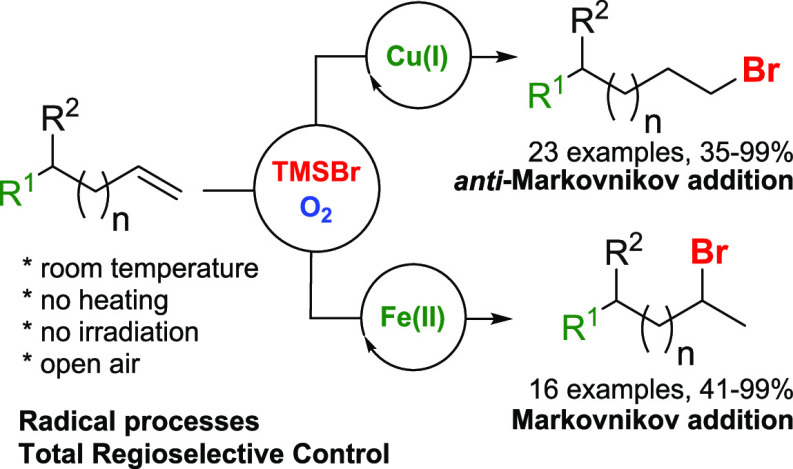

A new method that allows the complete control of the regioselectivity of the hydrobromination reaction of alkenes is described. Herein, we report a radical procedure with TMSBr and oxygen as common reagents, where the formation of the *anti*-Markovnikov product occurs in the presence of parts per million amounts of the Cu(I) species and the formation of the Markovnikov product occurs in the presence of 30 mol % iron(II) bromide. Density functional theory calculations combined with Fukui’s radical susceptibilities support the obtained results.

Terminal olefins are valuable synthons in organic synthesis. In addition to their abundance in natural products,^1,2^ olefins can be modified into a great variety of functional groups using many readily available processes.^3,4^ Among them, HX additions are an important class of reactions that allow the interconversion of alkenes into synthetically useful intermediates.^5^ This addition reaction shows a divergence in regioselectivity from an ionic pathway that affords the Markovnikov addition product to a free radical pathway that gives rise to the *anti*-Markovnikov addition product. Among these types of reactions, the hydrobromination reaction typically requires quite harsh conditions that are not compatible with several functional groups and, most frequently, leads to the formation of a mixture of regioisomers due to the occurrence of these two competitive processes (Scheme 1a).^6^ Considering a common free radical scenario, the Markovnikov addition product is not a trivial target as it would imply the addition of a hydrogen atom to the olefin at the first step, which is usually effected by transition metal hydrides in a catalytic oxidation and reduction process.^7^ To date, common radical reaction conditions able to achieve complete regioselective control (with slight modifications and no need to alter the nature of the process) remain unexplored.

**Scheme 1:**
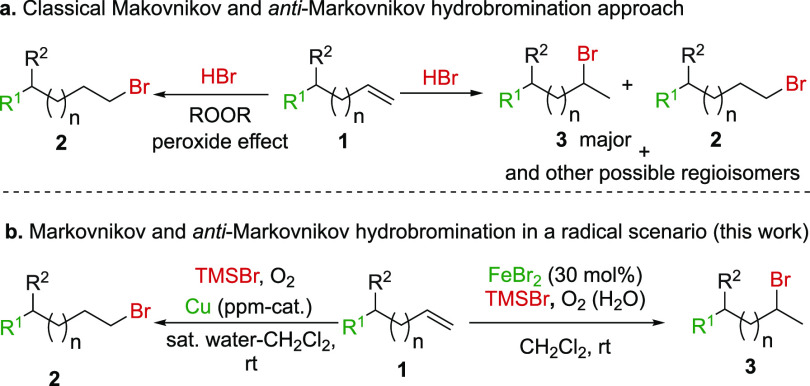
Hydrobromination Addition Reaction Approaches

Herein, we report the use of TMSBr and oxygen as common reagents in a free radical hydrobromination process. Its combination with different transition metals such as Cu(I) and Fe(II) successfully leads to complete regioselectivity control in the hydrobromination reaction of alkenes (Scheme 1b).

During our work on the synthesis of tetrahydropyrans through Prins cyclization,^8^ traces of Markovnikov hydrobrominated products of the alkene were detected when using the Lewis acid system FeBr_3_/TMSBr in dry dichloromethane at room temperature. To verify this result in detail, we decided to carefully study the effect of each reagent. First, we set up a reaction under nitrogen atmosphere and used an excess of TMSBr as the brominating agent. After a ten-day reaction, we observed the formation of a product that turned out to be the *anti*-Markovnikov hydrobrominated olefin in a 70% yield. Since abnormal addition products are widely accepted to be formed via a radical pathway with the help of an initiator (Scheme 1a), we decided to clarify the nature of the whole process.

We initiated an optimization process by considering the nature of the initial alkene, bromotrimethylsilane (TMSBr), the proton source, oxygen, initiators, transition metals, and solvents. The effect of these variables on the process was carefully investigated (for a detailed explanation of the identification of the elements involved in the process, see the Supporting Information).

After the optimization process, we developed an effective and simple method that consists of the following components: (a) water-saturated CH_2_Cl_2_, (b) oxygen (present in the reaction media), (c) amylene (2-methyl-2-butene) as an initiator, (d) TMSBr, and (e) Cu as the transition metal (present in commercially available TMSBr batches). The synergistic action of all and each of these species makes the course of the reaction possible (Scheme 1b).^9^

Next, we decided to explore the scope of the reaction using different substrates (Figure 1). When a plain 11 carbon chain was used, the reaction proceeded with a quantitative yield (>99%) (**2a**). The introduction of a hydroxyl group inhibited the reaction (**2b**). To get more complexity in the molecule, we decided to use branched alkenes and different *O*-benzoylated alcohols, diversifying their chain lengths. In all cases, the reaction afforded the corresponding products in excellent to quantitative yields (**2c**–**2h**). Next, when an aromatic substrate, such as allylbenzene, was used, the reaction also proceeded with a good yield (**2i**). This behavior was maintained when *trans*-β-methylstyrenes were used (**2j**–**2l**). The introduction of a halogenated group such as bromine proceeded with an excellent yield of the product (**2k**), while the presence of a nitro group gave only a moderate yield (**2l**). Finally, we increased the complexity of the molecule using different substituted aromatic homoallyl ethers (**2m**–**2o**). The yield of the addition reaction was not affected by the presence of either electron-withdrawing or electron-donating groups (**2m, 2n**). On the other hand, the use of substrates with different heteroatoms like allylthiophenol worked well (**2p**). The versatility of the reaction was showcased by the use of carbohydrate derivatives, which gave the primary bromide in moderate to excellent yields and with no anomerization (**2q** and **2r**, respectively). When using the furan derivative **2s**, the reaction proceeded with complete chemoselectivity as the the allylic position is more reactive than the vinylic one. Fukui’s radical susceptibilities showed a divergence between the allylic (higher values) and vinylic positions, indicating that the radical addition should occur at the allylic position (see Figure S4 in the Supporting Information). Moreover, substrates containing esters and nitrogen also worked well, with excellent to moderate yields (**2t**–**2v**). No reaction was observed in the presence of a ketone (**2w**). The reaction was scalable up to 2 g (**2d**, 89%) and no further purification was required (Figure 1).

**Figure 1:**
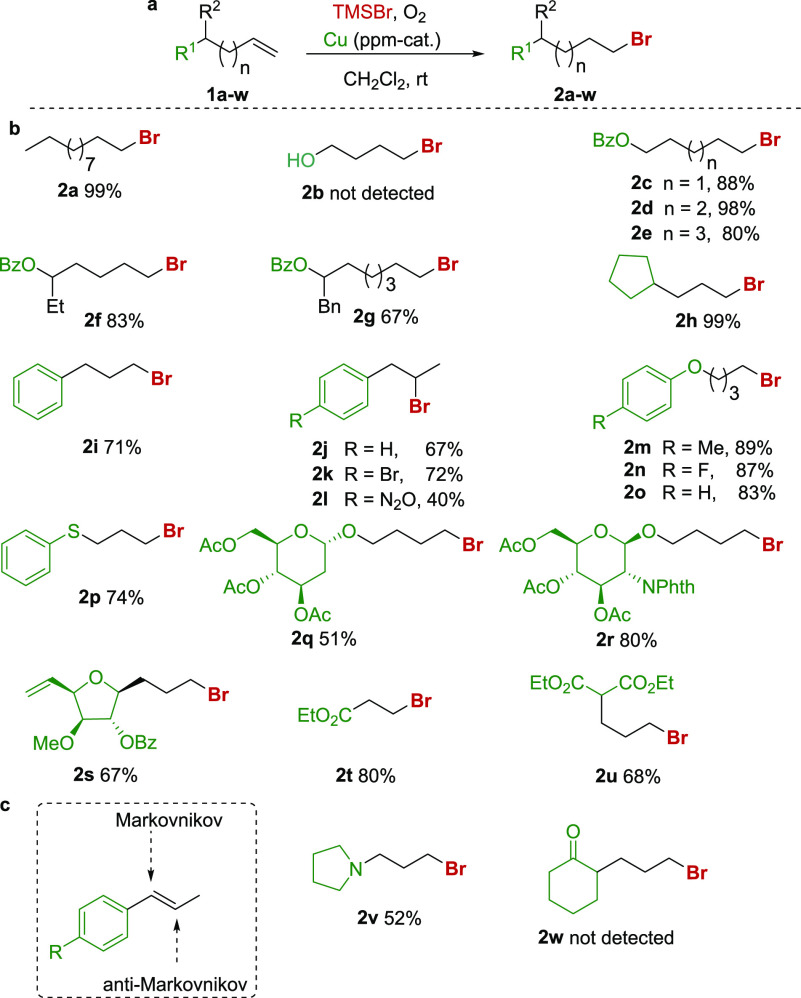
Reaction conditions. (a) Alkene (1.2 mmol, 1.0 equiv), TMSBr (3.0 equiv), CH_2_Cl_2_ (0.1 M), saturated H_2_O (Milli-Q), O_2_ (present in the solvent and the Milli-Q water), amylene (170 ppm), and CuBr (present in the commercial TMSBr). (b) Scope and yields of the *anti*-Markovnikov hydrobromination of alkenes. (c) Markovnikov and *anti*-Markovnikov orientations in styrene derivatives.

With these results in hand, we focused on the idea of reversing the reaction pathway toward the exclusive formation of the corresponding Markovnikov product. The existence of a putative amount of metalloradicals containing iron(II), which are able to modulate the reactivity of *S*-adenosyl methionine systems,^10^ showed us that the presence of iron(II) would act as a modulator in the outcome of the reaction. Moreover, iron(II) catalysts are present in a wide number of radical processes,^11−13^ making them an attractive alternative for modifying radical processes without altering their nature.

Before conducting the experiments, we carried out density functional theory (DFT) calculations at the dispersion-corrected PCM-B3LYP-D3/def2-SVP level on the AcO—(CH_2_)_3_—CH=CH_2_ substrate. According to the computed Fukui’s radical susceptibilities (*R*), the radical addition should preferentially occur at the terminal carbon atom of the C=C double bond, which is fully consistent with the experimental results described below (Figure 2a). At variance, the coordination of the alkene and carbonyl groups of the substrate to FeBr_2_ should provoke a dramatic change in the selectivity of the radical addition in view of the higher *R* value that was computed for the internal carbon atom as compared to that for the terminal one. Therefore, our calculations predict that the presence of FeBr_2_ could efficiently revert the selectivity of the transformation and indicate that the coordination of the C=C double bond to the transition metal is mandatory to produce the Markovnikov product (Figure 2b).

**Figure 2:**
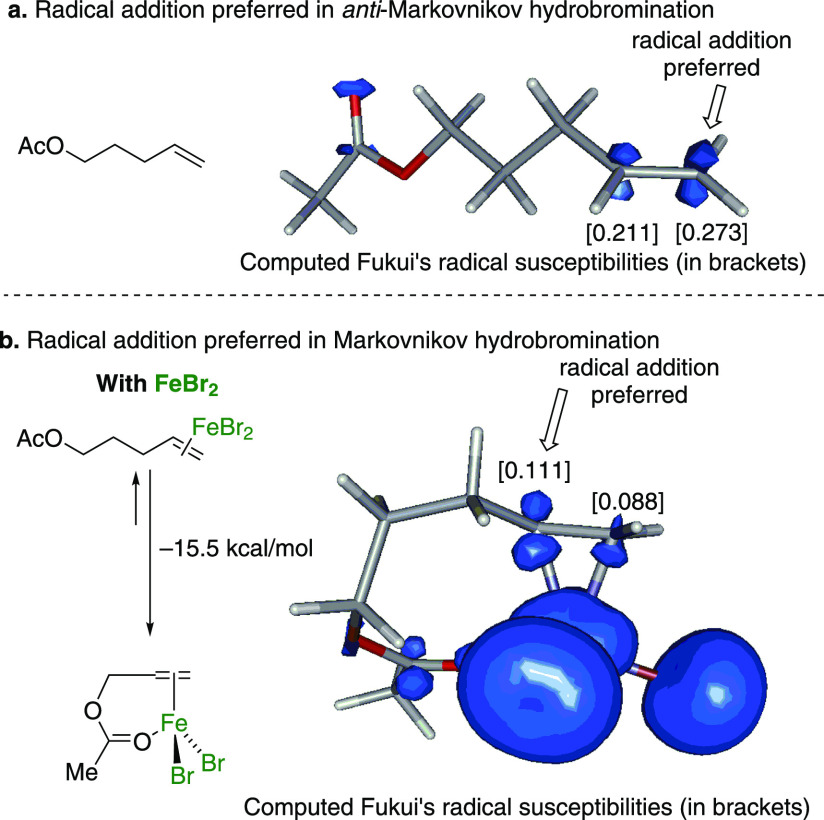
Preferred radical addition in the hydrobromination of AcO—(CH_2_)_3_—CH=CH_2_.

Encouraged by these results, BzO—(CH_2_)_4_—CH=CH_2_ was treated with FeBr_2_–TMSBr in dichloromethane, resulting in the exclusive formation of the Markovnikov reaction product (Table S4 in the Supporting Information). We screened different reaction conditions for the catalytic system and found that 0.3 equiv of FeBr_2_ alongside 3.0 equiv of TMSBr were the best (90% reaction yield). We also verified whether oxygen was involved in the process. To this end, we checked the following sets of reactions: (a) open-air, (b) under a nitrogen atmosphere, and (c) under a nitrogen atmosphere with deoxygenated DCM. The open-air reaction afforded a quantitative yield, while that under a nitrogen atmosphere only worked with a 30% yield due to the oxygen already present in DCM. In the last case, i.e., under an inert atmosphere and deoxygenated DCM, the reaction did not proceed at all. On the other hand, a proton source is also necessary and comes from the moisture of the air (see the Supporting Information). Therefore, it became evident that the presence of oxygen and moisture is essential for the success of the reaction. On the contrary, the presence of amylene in the reaction media is irrelevant, as it can be seen from the experimental results (Table S4 in the Supporting Information).

With these optimized reaction conditions in hand, we selected representative substrates to confirm if the regioselectivity of the hydrobromination reaction could be controlled (Figure 3). In all cases, the treatment of the substrates with the FeBr_2_-TMSBr catalytic system afforded the corresponding Markovnikov product in good to excellent yields. Thus, long-chain substrates and nonprotected alcohols afforded the desired products with good yields (**3a** and **3b**, respectively). The use of *O*-benzoylated alcohols also gave good to excellent yields of the products (**3c**–**3e**).

**Figure 3:**
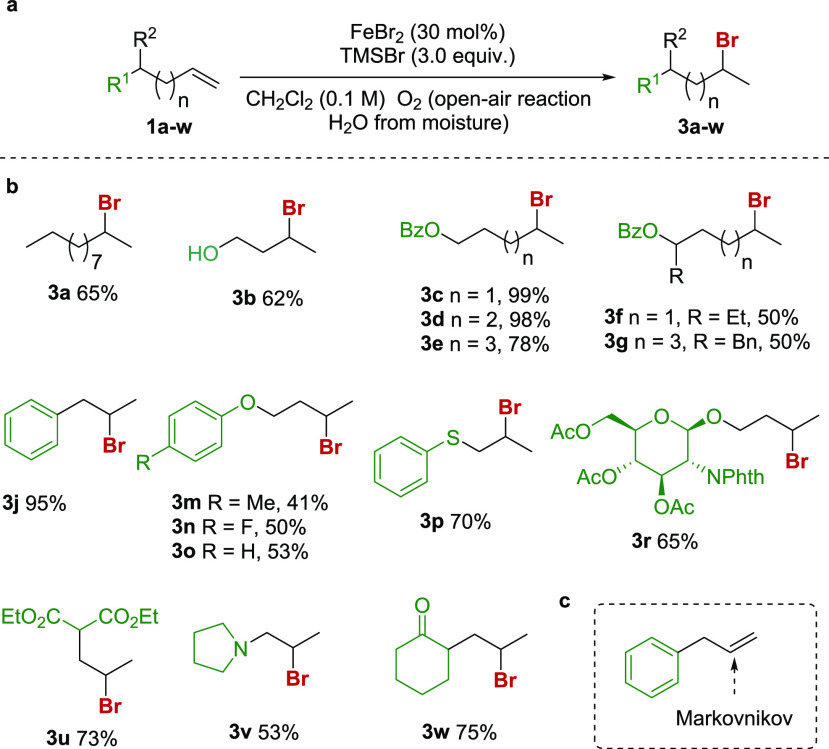
Reaction conditions. (a) Alkene (1.2 mmol, 1.0 equiv), FeBr_2_ (0.3 equiv), TMSBr (3.0 equiv), CH_2_Cl_2_ (0.1 M), O_2_ (present in the air, the solvent, and H_2_O from moisture). (b) Scope and yields of the Markovnikov hydrobromination of alkenes. (c) The Markovnikov orientation in allyl benzene.

With these optimized reaction conditions in hand, we selected representative substrates to confirm if the regioselectivity of the hydrobromination reaction can be controlled (Figure 3). In all cases, treatment of the substrates with the FeBr_2_-TMSBr catalytic system afforded the corresponding Markovnikov product in good to excellent yields. Thus, long-chain substrates and nonprotected alcohols afforded the desired products with good yields (**3a, 3b**). The use of *O*-benzoylated alcohols also gave good to excellent yields (**3c**-3**e**). When the reaction was carried out using secondary *O*-benzoylated alcohols, although the reaction was clean, it proceeded only with a moderate yield (**3f**, **3g**). The use of aromatic substrate like allylbenzene also proceeded with excellent yield (**3j**), while the use of aromatic ethers gave moderate yields (**3m**–**3o**), a pattern that was maintained when different substituents (methyl and fluor) were introduced in the ring (**3m**, **3n**). The carbohydrate derivative **3r** gave the Markovnikov bromide in good yield and with no anomerization. In this particular case, the brominated derivative was attained as a diastereomeric mixture (**3r**). The use of a thiol derivative also gave a good yield (**3p**), while the pyrrolidine derivative gave a moderate yield (**3v**). In addition, the use of substrates like diethyl allylmalonate and 2-allylcyclohexanone proved to be successful (**3u**–**3w**).

A plausible mechanism of the *anti*-Markovnikov process is proposed accordingly (Figure 4a). Cu(I) in the presence of O_2_ catalyzes the formation of the hydroperoxide **A**,^14^ and releases a Cu(II) ion via single-electron transfer (SET). Then, the oxygen–oxygen bond of the hydroperoxide breaks homolytically due to the action of Cu(I), producing the peroxyl radical **B**. This radical initiates the chain reaction with HBr,^15^ forming bromine radicals and the corresponding trimethylsilylether **C**. Bromine radicals add to the alkene to form the most stable radical intermediate **D**, which evolves toward the final product via a reaction with HBr, which was generated in a parallel process as indicated in Figure 4. Indeed, when the reaction was carried out in the presence of deuterium oxide (D_2_O), the hydrogen in the final bromoalkane **2** was entirely exchanged by deuterium (Figure 4a; see the Supporting Information for further details).

**Figure 4:**
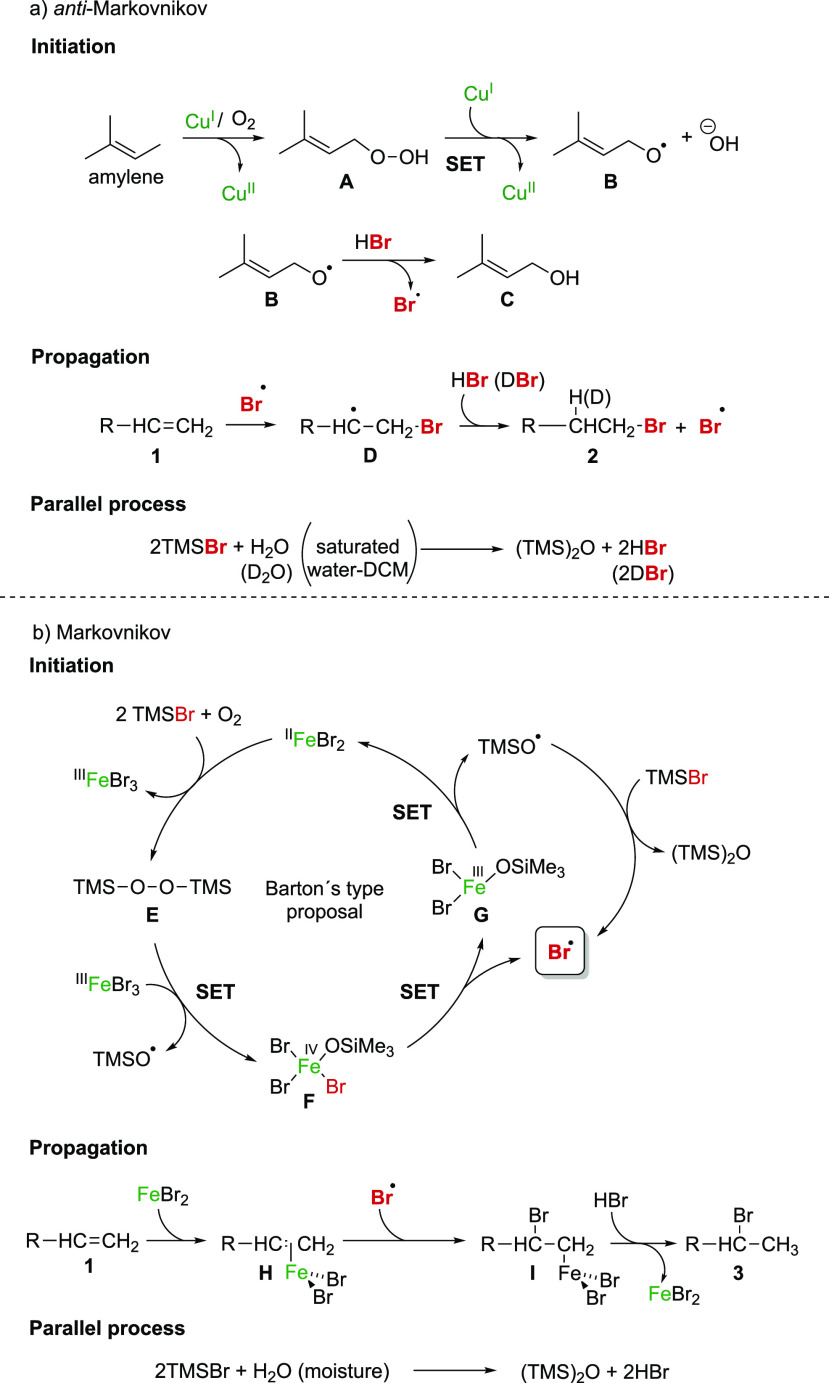
Proposed mechanisms for the anti-Markovnikov and Markovnikov hydrobromination.

In order to locate the rate-determining step of the reaction, kinetic isotopic effects (KIE) studies were performed.^16,17^ A comparison of the kinetic constants obtained with water and deuterium oxide revealed a kinetic isotope effect of 3/4. Therefore, the isotopic substitution bond is not broken during the rate-determining step, which is consistent with the hypothesis by Mayo et al.^18^ on the slow formation of bromine radicals with oxygen.

In the radical Markovnikov process, FeBr_2_ in the presence of O_2_ and TMSBr catalyzes the formation of bis(trimethylsilyl)peroxide **E**,^14,19^ which is an effective oxidant. The FeBr_3_ generated in the process is oxidized by the action of **E** to give the iron(IV) species **F**. Next, the subsequent formation of bromine radicals, which release the iron(III) species **G**, ensures the regeneration of iron(II) in the system, as proposed by Barton and Chabot (Figure 4b).^20^

Once this bromine radical is formed, addition to the internal carbon atom of the FeBr_2_-coordinated C=C double bond of **H** gives rise to the Markovnikov brominated product with the concomitant regeneration of the FeBr_2_ due to the presence of HBr, which is generated in a parallel process by reaction of the TMSBr with the humidity that carries the oxygen (Figure 4b).^21^

In summary, we have developed reaction conditions that allow us efficient control of the regioselectivity of the radical addition to C=C double bonds in the hydrobromination reaction (Markovnikov and *anti*-Markovnikov). The absence of irradiation and heating makes the anti-Markovnikov addition reaction proceed smoothly and rather spontaneous, in a simple and scalable manner. At variance, the use of FeBr_2_ leads to the exclusive formation of the corresponding Markonikov regioisomer, with no need for metal hydrides and oxidation and reduction processes.

## Supporting Information Available

Complete experimental procedures, optimization, characterization, kinetic isotopic effect, ^1^H and 13C NMR spectra for all new compounds, and computational details (PDF)
