# Supplementary material for: Iron(II) and Copper(I) Control the Total Regioselectivity in the Hydrobromination of Alkenes
Source: Org Lett. 2021 Jul 28;23(15):6105–9. doi: 10.1021/acs.orglett.1c02186 (PMC8397429; doi:10.1021/acs.orglett.1c02186)
Supplement: Supplementary file 1 — ol1c02186_si_001.pdf [file ol1c02186_si_001.pdf]

# Supporting Information

# Iron(II) and copper(I) control the Total Regioselectivity in the Hydrobromination of Alkenes

Daniel A. Cruz,[b] Victoria Sinka,[a] Pedro de Armas,[a] Hugo Sebastian Steingruber,[b] Israel Fernández,[c] Víctor S. Martín,[b] Pedro O. Miranda,*[a] and Juan I. Padrón*[a].

[a] Ms. V. Sinka, Dr. P. de Armas, Dr. P. O. Miranda, Dr. J. I. Padrón
Molecular Sciences Department. Instituto de Productos Naturales y Agrobiología, Consejo Superior de Investigaciones Científicas (IPNA-CSIC). Avda. Astrofísico Francisco Sánchez 3, 38206 La Laguna, Tenerife, Islas Canarias, Spain. E-mail: pmiranda@ipna.csic.es; jipadron@ipna.csic.es

[b] Dr. D. A. Cruz, MsC. H. S. Steingruber, Dr. V. S. Martín
Instituto Universitario de Bio-Orgánica "Antonio González" (CIBICAN), "Síntesis Orgánica Sostenible, Unidad Asociada al CSIC", Departamento de Química Orgánica
Universidad de La Laguna
C/ Francisco Sánchez 2, 38206 La Laguna, Tenerife, Islas Canarias, Spain

[c] Dr. I. Fernández
Departamento de Química Orgánica I y Centro de Innovación en Química Avanzada (ORFEO-CINQA), Facultad de Ciencias Químicas
Universidad Complutense de Madrid
28040 Madrid, Spain

## Table of Contents

Experimental procedures ........ S3
Material and Methods ........ S3
General procedure 1 (*anti*-Markovnikov hydrobromination) ........ S3
General procedure 2 (Markovnikov hydrobromination) ........ S3
General procedure 3 (water-saturated solvent) ........ S3
Identification and optimization of the elements involved in hydrobromination reaction ........ S4
Effect of the initiators and transition metals in the *anti*-Markovnikov hydrobromination ........ S8
Singular results explanation based on computed Fukui´s radical susceptibilities ........ S12
Experimental Procedure and characterization data for starting materials ........ S13
Characterization data for *anti*-Markovnikov hydrobromination products ........ S18
Characterization data for Markovnikov hydrobromination products ........ S23
Mechanistic experiments ........ S28
Computational details ........ S33
$^{1}$H- and $^{13}$C-NMR spectra for starting materials and final products ........ S37
References ........ S116

## Experimental Procedures

### Material and Methods*:*

NMR spectra were recorded on a Bruker Advance instrument. $^{1}H$ NMR spectra were recorded at 400, 500 and 600 MHz, and $^{13}C$ NMR were recorded at 100, 125 and 150 MHz, VTU 298.0 °K. Chemical shifts were reported in parts per million. The residual solvent peak was used as an internal reference ($CDCl_3$: $\delta_H$ 7.26, $\delta_C$ 77.0).

Optical rotations were measured on a Perkin-Elmer 343 Polarimeter by using a Na lamp. HRESMS data were performed on a Micromass Autospec LCT Premier XE orthogonal acceleration time-of-lap spectrometer (oa-TOF) by addition of $CHO_2H$ (10% in water).

All measurements of trace metals determination were made using a Perkin Elmer ICP OES Avio 500.

For analytical thin-layer chromatography, silica gel ready-foils was used, respectively, being developed with 254 nm UV light and/or spraying with a solution of phosphomolybdic acid solution (20 wt. % in ethanol) and heating or potassium permanganate stain and heating. Column chromatography was performed using silica gel (0.015-0.04 mm) and *n*-hexane/EtOAc or DCM/MeOH solvent systems. Radial chromatography was performed using silica gel (1, 2 and 4 mm disc) and *n*-hexane/EtOAc solvent systems. All reagents were obtained from commercial sources (Aldrich® and Alfa Aesar®) and used without any further purification. Solvents were dried and distilled before use.

### General procedure 1 (*anti*-Markovnikov hydrobromination)

To a 0.1 M solution of alkene in a water-saturated dichloromethane solution (see general procedure 3), was added TMSBr (3.0 equiv). The reaction was followed by TLC and, when the reaction reached complete conversion, water was added, and the reaction mixture was extracted with DCM. The combined organic layers were dried over magnesium sulphate anhydrous, filtered, and the solvent was removed under reduced pressure. Unless otherwise noted, the crude reaction mixture was purified by flash silica gel column chromatography (*n*-hexane/EtOAc 95:5 solvent system).

### General procedure 2 (Markovnikov hydrobromination)

To a 0.1 M solution of alkene in dry, non-deoxygenated, dichloromethane solution, was added $FeBr_2$ (0.3 equiv) followed by TMSBr (3.0 equiv). The reaction was stirred at room temperature and followed by TLC. When the reaction reached complete conversion water was added, and the reaction mixture was extracted with DCM. The combined organic layers were dried over magnesium sulphate anhydrous, filtered, and the solvent was removed under reduced pressure. Unless otherwise noted, the crude reaction mixture was purified by flash silica gel column chromatography (*n*-hexane/EtOAc 95:5 solvent system).

### General procedure 3 (water-saturated solvent)

A water saturated solution of dichloromethane was obtained by vigorously shaking it in a separatory funnel with 50% (v/v) Milli-Q® water, and separating the organic phase from the water phase, after allowing the mixture to stand for 3 hrs. The solvent was kept in a closed flask, and it was used for the next two weeks.

**Identification and optimization of the elements involved in the hydrobromination reaction:**

Regarding the free radical reaction, a very important component is dioxygen ($O_2$), which exhibits a triplet ground state.[1] During the first half of the past century, dioxygen was believed to play a key role in the anti-Markovnikov hydrobromination of alkenes, based on the studies (light, solvent, peroxides, etc.) of several authors such as Bauer,[2,3] Sherrill,[4] Kharasch,[5,6] and Smith, among others.[7–10] However, the role of dioxygen faded into obscurity due, on the one hand to the lack of reproducibility from laboratory to laboratory, and on the other hand to studies in the field that concluded that either the light and/or irradiation or the impurities of starting materials were responsible for the reaction outcome.[6,11–14] Moreover, the unusual combination of strong π bonding and weak σ bonding in $O_2$, alongside with its 100 kcal/mol resonance energy for the triplet diradical, enables the molecule to form complexes with transition metals such as copper or iron.[15] In fact, copper oxygen species are very important intermediates involved in a variety of synthetic organic reactions that take place under smooth conditions.[16]

We initiated an optimization process by considering the nature of the initial alkene, bromotrimethylsilane (TMSBr), proton source, oxygen, initiators, transition metals and solvents. The effect of these variables on the process was carefully investigated.

*a) Proton source*

Water is an essential part of the process acting as a proton source in the outcome of the reaction. Saturation of the solvent with water is mandatory in order to ensure that the reaction takes place in short reaction times (30 minutes) and with high yields.

*b) Solvent*

Water-saturated dichloromethane is found to be the ideal solvent.[17] However, the reaction also takes place with either water-saturated chloroform or water-saturated 1,2-dichloroethane, which afford similar reaction yields. The reaction proceeds using 0.1 M concentrations.

*c) Oxygen*

Hansen solubility parameters (HSPs) of oxygen are rather similar to those of dichloromethane.[18,19] The similarity of the HSPs values makes dichloromethane an excellent way to provide oxygen to the media.[20]

*d) Initiator*

In an interesting review by Mayo et al., it is described the rapid formation of peroxides in the presence of highly reactive alkenes such as stilbene or allyl bromide.[21] This led us to hypothesize that the presence of a highly reactive alkene should be responsible for the formation of the peroxide. However, our starting material was not reactive enough to promote the formation of peroxides by itself in such a short time. Delving into the reaction mixture, we found that commercial dichloromethane is typically stabilized with amylene, a highly reactive alkene used as a solvent stabilizer due to its ability as a radical scavenger.[22] At first sight, amylene seemed the wrong candidate to promote the reaction. However, when the hydrobromination reaction was carried out in amylene-free dichloromethane, no reaction took place. In sharp contrast, when a small portion of amylene (20 ppm) was added to initiator-free solvent, the reaction was completed in 30 minutes. The same behavior was observed when other alkenes such as cyclohexene (10 ppm), and 1,5-dicyclooctadiene (COD, 5 ppm) were used.

*e) Bromine source*

Despite its use as a mild brominating agent, TMSBr may decompose upon exposure to air moisture or water. To preserve it and commercialize it, metal traces of Cu and Ag are added to it, since both metals are used to trap traces of bromine from the decomposition of TMSBr. The use of equimolar amounts of TMSBr proved to be relatively unsuccessful since no complete conversion was achieved. Tuning out reaction conditions showed that an excess of TMSBr was necessary to achieve full conversion of the reaction in short times.

*f) Transition metal*

The identification of amylene and/or COD as initiators did not answer the question about the formation of the peroxyl radical. It was previously described that oxygen can add to alkenes to form peroxides.[23,24] The RO–OR bond dissociation energy for peroxide homolysis is ~ 36 kcal/mol, which makes these species very useful as a radical source by thermal decomposition.[25–27] The ease of radical formation by thermal cleavage of the O–O bond makes peroxide initiators valuable molecules in the field of radical reactions, since they act as initiators of the whole process. Similarly, the azo-initiators are another family of initiators being 2,2´-azo(bisisobutyronitrile) (AIBN) the most prominent one, with a bond dissociation energy of 72 kcal/mol. Usually, reactions promoted by AIBN require heating up to 80 ºC.[28]

Since our reaction takes place at room temperature, with no need of heating or irradiation, we hypothesized that the metal traces present in the TMSBr as stabilizers should be responsible somehow for the homolytic cleavage. In fact, transition metals were initially suspected of being involved in the *anti*-Markovnikov addition in the early studies reported on the topic.[29,30]

Thus, the effective reaction mixture would be formed by the water-saturated halogenated solvent (which carries oxygen dissolved on it and the alkene initiator), and CuBr or AgBr as transition metal (included in the TMSBr). In order to verify this statement, we proceeded to use home-made TMSBr with no traces of transition metal (checked by ICP-MS).[31] When we ran the reaction with our home-made TMSBr, no reaction took place. However, when we added traces (10 ppm) of AgBr or CuBr to the reaction mixture, we achieved the brominated product in 30 minutes and with the same yields commented above (see section "Effect of the initiators and transition metals in the *anti*-Markovnikov hydrobromination").

**Table S1.** Optimization of the *anti*-Markovnikov hydrobromination reaction conditions.[a]

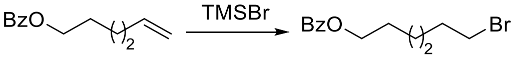


1d 2d

| Entry | TMSBr (equiv.) | Solvent | Oxygen source | Initiator | T (ºC) | Yield (%)[b] | T (h) |
|---|---|---|---|---|---|---|---|
| 1 | 1.0 | DCM | air | none | rt | NR | 240 |
| 2 | 2.0 | DCM | air | none | rt | 50 | 240 |
| 3 | 3.0 | DCM | air | none | rt | 70 | 240 |
| 4 | 3.0 | DCM | air | light | rt | 5 | 100 |
| 5 | 3.0 | DCM | air | LED | rt | NR | 216 |
| 6 | 3.0 | DCE | air | AIBN | 80 | NR | 50 |
| 7 | 3.0 | DCE | air | none | 80 | 55 | 144 |
| 8[c] | 3.0 | DCE | air | MW | 80 | 34 | 100 |

| | | | | | | | |
|---|---|---|---|---|---|---|---|
| 9[d] | 3.0 | DCM | air | none | rt | NR | 240 |
| 10[e,f] | 3.0 | DCM | air | none | rt | 99 | 0.5 |
| 11[e] | 3.0 | DCM | $O_2$ | none | rt | 97 | 0.5 |
| 12[e,g] | 3.0 | DCM | none | none | rt | NR | 240 |
| 13[h] | 3.0 | $CHCl_3$ | air | none | rt | 95 | 0.5 |
| 14[h] | 3.0 | $CHCl_3$ | $O_2$ | none | rt | 96 | 0.5 |

[a] Reaction conditions: **1a** (0.5 mmol), TMSBr, solvent (0.1 M), rt. [b] Yield of isolated product. [c] MW at 200 W. [d] With 4.0 equiv of water. [e] DCM-saturated water. [f] 73% yield with 1.0 equiv of TMSBr, 81% yield with 2.0 equiv of TMSBr. [g] deoxygenated. [h] $CHCl_3$-saturated water. DCM = dichloromethane, DCE = dichloroethane, AIBN = 2,2'-azobis(2-metilpropionitrilo), MW = microwave, NR = not reacted.

The effect of variables such as solvent, initiator and temperature were systematically studied and are shown in Table S1. The choice of solvent was made carefully, in order to improve the oxygen supply of the process. The Hansen solubility parameters (HSPs) have proven to be a powerful and practical way to understand the solubility in various solvents.[24] In fact, the relationship between HSPs and solubility for many substances has been reported,[25] including studies on oxygen solubility.[26] As a solvent we chose dichloromethane (DCM) based on the similitudes of HSPs values of both DCM and oxygen, physical attributes that make it an excellent solvent able to dissolve and provide oxygen to the media. We carried out all the reactions in an open-air manner, in order to facilitate bromine radical formation through oxygen transfer. Thus, we found the best reaction conditions using 3.0 equiv. of TMSBr in DCM, although the reaction took ten days to reach to conclusion (Entry 3). In order to speed up the reaction we proceed to irradiate using visible light with limited success (Entry 4). We also irradiated using green LED, since their maximum emission wavelength is 525 nm, (which is a suitable wavelength for bromo photoactivation),[27] with poor results (Entry 5). Moreover, the addition of classical initiators such as AIBN proved to be fruitless (Entry 6). At this point, we decided to use 1,2-dichloroethane as a solvent because of its similar behavior with DCM, and the possibility of increasing the temperature of the reaction up to 80 ºC (Entry 7). These conditions afforded **2d** with moderate yields. Microwave conditions did not show any improvement (Entry 8). Thereupon, we decided to add water to the reaction mixture in order to secure the proton source necessary for achieving the completion of the reaction. No product formation was observed (Entry 9). Next, we used water-saturated DCM as a solvent (Entry 10). In this occasion, the reaction took place in 30 min. and with almost quantitative yields. Since the reaction was performed in open air atmosphere, we decided to clarify the importance of the oxygen in the reaction. Thus, we proceed to run the reaction using water saturated solvent under $O_2$ atmosphere. These conditions afforded the desired product in excellent yields (Entry 11). Following this result, we decided to deoxygenate the solvent and perform the reaction in $N_2$ atmosphere. No product formation was observed (Entry 12). As a final step, we also explored other halogenated solvents, such as chloroform, with similar results.

**Table S2.** Effect of water in the hydrobromination *anti*-Markovnikov reaction outcome.

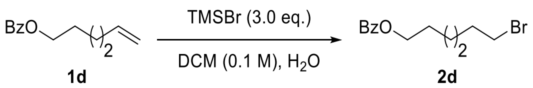


**1d** **2d**

| Entry | Water | Time (h) | Yield (%) |
|---|---|---|---|

| | | | |
|---|---|---|---|
| 1 | 0.45 mg/mL DCM | 42 | <5 |
| 2 | 2.0 mg/mL DCM | 52 | <5 |
| 3 | 40.0 mg/mL DCM | 50 | NR |
| 4 | Saturated | 0.5 | 98 |

NR = not reacted

**Table S3:** Hydrobromination reaction using commercial HBr (48% wt in water)

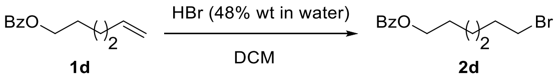


| Entry | HBr 48% wt (equiv.) | Time (h) | Yield (%) |
|---|---|---|---|
| 1 | 1.0 | 24 | NR |
| 2 | 3.0 | 24 | NR |
| 3 | 10.0 | 50 | NR |

**Table S4:** Optimization of the Markovnikov hydrobromination reaction conditions.

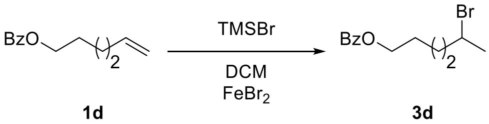


| Entry | TMSBr (equiv.) | $FeBr_2$ (equiv.) | Amylene (ppm) | Yield (%) |
|---|---|---|---|---|
| 1 | 3.0 | 0.1 | - | 45 |
| 2 | 3.0 | 0.2 | - | 47 |
| 3 | 3.0 | 0.3 | - | 90 |
| 4 | 3.0 | 0.5 | - | 90 |
| 5 | 3.0 | 1.0 | - | 85 |
| 6 | 2.0 | 0.3 | - | 45 |
| 7 | 1.0 | 0.3 | - | 43 |
| 8 | 3.0 | 0.3 | 10 | 90 |

To check the relevance of oxygen in the Markovnikov addition, two reactions were set: A) open-air; B) under nitrogen atmosphere. The open-air reaction finished with a quantitave yield, while under nitrogen atmosphere only worked with a 30% yield, after 20 hours, due to the oxygen present in DCM (see table S5). Therefore, it became evident that the presence of oxygen in stoichiometric amount is essential for the reaction completion.

**Table S5:** Effect of oxygen amount in the Markovnikov addition.

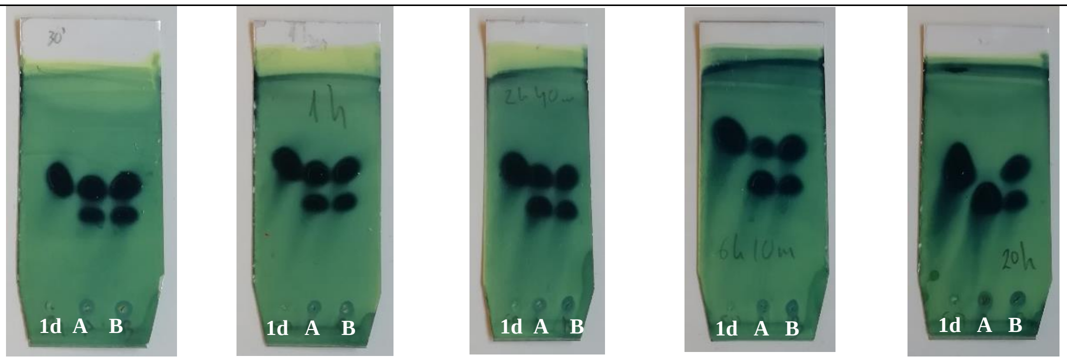


| | | | | |
|---|---|---|---|---|
| 0.5 h | 1 h | 2 h 40 min | 6 h 10 min | 20 h |

## Effect of the initiators and transition metals in the *anti*-Markovnikov hydrobromination

In this section, the exploration conducted in order to figure out the role of each component in the *anti*-Markovnikov hydrobromination reaction is described.

First, Fig. S1 shows the influence of the different initiator types, different transition metals, as well as their combination.

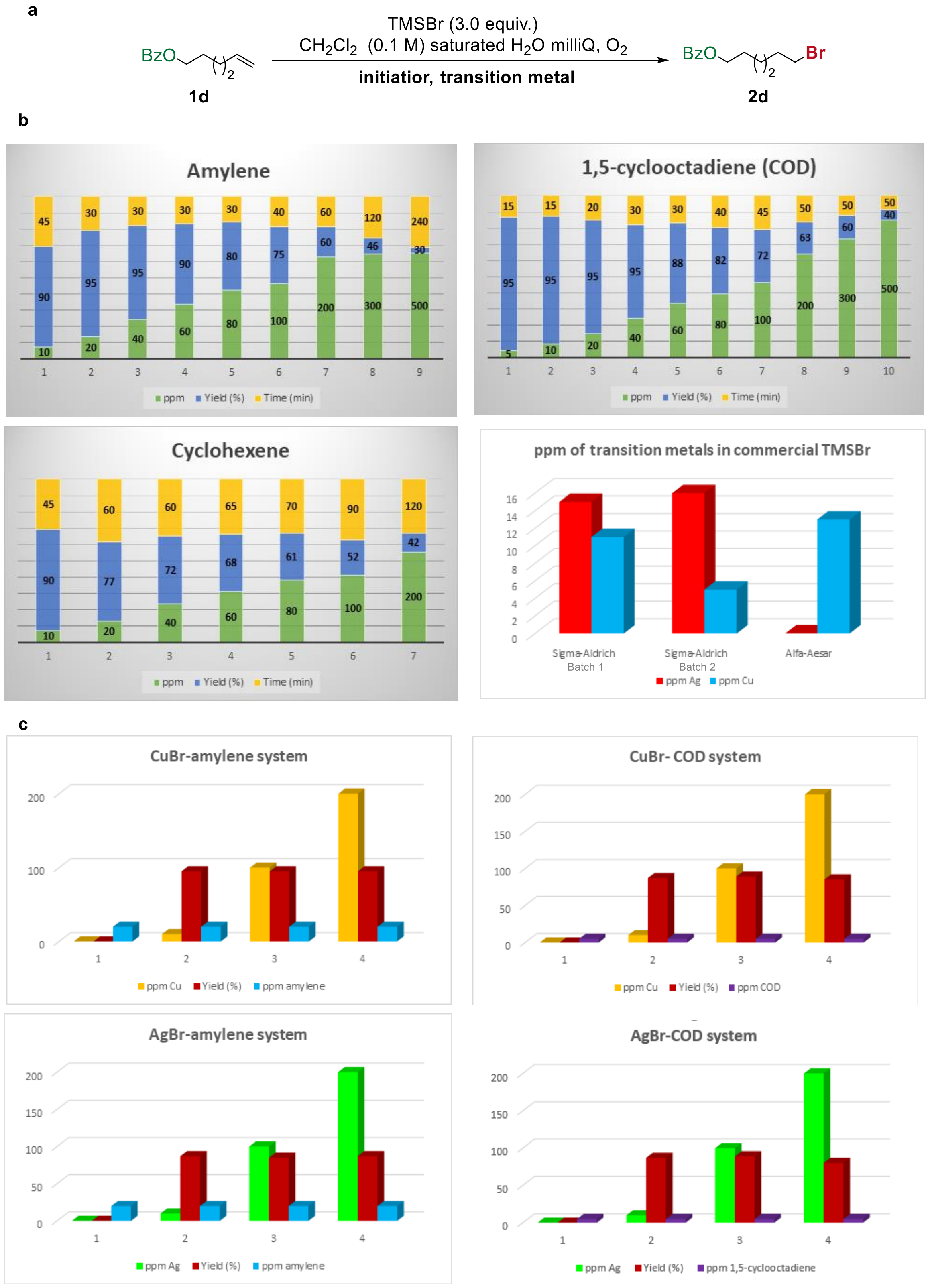


**Figure S1. *Anti*-Markovnikov hydrobromination of alkenes**. **a**, General scheme. **b**, Influence of different amounts of initiators in reaction times and yields. ppm of transition metals found in commercially available TMSBr from Sigma-Aldrich® (2 different batches) and Alfa-Aesar®. **c**, Influence of Cu(I) and Ag(I) combined with the initiators amylene and 1,5-cyclooctadiene in the reaction outcome.

As it can be seen in Fig. S1, as the amount of amylene increased, the corresponding reaction times became longer and yields lower, a pattern that was observed for COD as well. Differently, the use of cyclohexene

was not particularly successful as it led to even longer reaction times and lower yields than COD and amylene.
Analysis of metal traces by ICP-MS is shown in Fig. S1 for three commercially available batches of TMSBr (2 x Sigma-Aldrich® and Alfa-Aesar®). As it can be seen, the batches from Sigma-Aldrich presented significant amounts of Cu (presumably CuBr) and Ag (presumably AgBr). At variance, the batch from Alfa-Aesar only showed the presence of Cu.

Then, we screened to combine different transition metal-initiators, which are also shown in Fig. S1.

a) CuBr-amylene

The system proved to be successful in achieving the desired product using 20 ppm of amylene. Variations on CuBr amounts did not affect reaction yields. *Optimal Amount of CuBr*: 10 ppm.
We have found that many commercially available batches of DCM do not contain amylene. In these cases it is possible to add up to 170 ppm of amylene, in order to ease the reaction set up, with no compromise on the outcome of the process.

b) CuBr-COD

This system showed lower reaction yields than the previous one throughout the whole screening. Therefore, it was disregarded since it is less effective than the CuBr-amylene system.

c) AgBr-amylene

The same pattern observed with the CuBr-COD system was observed during the screening. Therefore, the system was also disregarded in favor of the CuBr-amylene system.

d) AgBr-COD

This system shows a similar pattern than the CuBr-amylene system, with excellent yields and short reaction times. In this case, only 5 ppm of COD are required for the reaction to occur. *Optimal Amount of AgBr*: 10 ppm.

Finally, with all the data gathered from the essays described above, two methods were proposed to check which initiator/metal transition combination was better (Method A and Method B).

**Method A:**

To a 0.1 M solution of alkene in a water saturated dichloromethane solution (containing 20 ppm of amylene), was added home-made TMSBr (3.0 equiv) followed by 10 ppm of CuBr freshly prepared. The reaction was followed by TLC, and when the reaction reached complete conversion water was added, and the reaction mixture was extracted with DCM. The combined organic layers were dried over magnesium sulphate anhydrous, filtered and the solvent was removed under reduced pressure. Unless otherwise noted, the crude reaction mixture was purified by flash silica gel column chromatography (*n*-hexane/EtOAc solvent system).

**Method B:**

To a 0.1 M solution of alkene in a water saturated dichloromethane solution (containing 5 ppm of 1,5-cyclooctadiene), was added home-made TMSBr (3.0 equiv) followed by 10 ppm of AgBr freshly prepared. The reaction was followed by TLC, and when the reaction reached complete conversion water was added, and the reaction mixture was extracted with DCM. The combined organic layers were dried over

magnesium sulphate anhydrous, filtered and the solvent was removed under reduced pressure. Unless otherwise noted, the crude reaction mixture was purified by flash silica gel column chromatography (*n*-hexane/EtOAc solvent system).

Fig. S2 shows the complete scope and the comparison of the outcome for Method A and Method B.

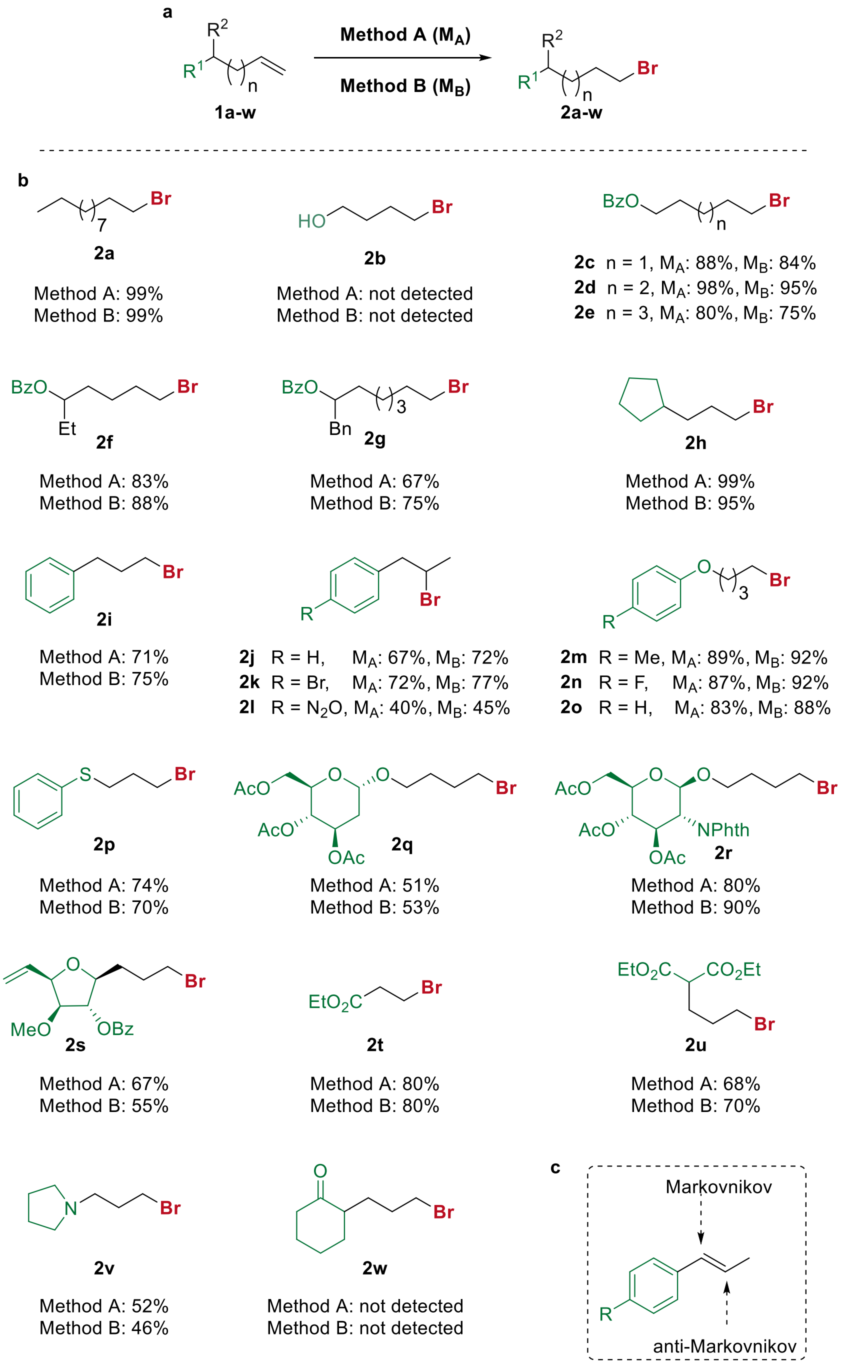


**Figure S2. *Anti*-Markovnikov hydrobromination reaction conditions**. **a,** Method A: alkene (1.2 mmol, 1.0 equiv.), TMSBr (3.0 equiv.), $CH_2Cl_2$ (0.1 M) saturated with $H_2O$ milliQ, $O_2$, amylene (170 ppm), CuBr (10 ppm). Method B: alkene (1.2 mmol, 1.0 equiv.), TMSBr (3.0 equiv.), $CH_2Cl_2$ (0.1 M) saturated with $H_2O$ milliQ, $O_2$, 1,5-cyclooctadiene (5 ppm), AgBr (10 ppm). **b**, Scope and yields of the *anti*-Markovnikov hydrobromination of alkenes comparing both methods. **c**, Markovnikov and *anti*-Markovnikov orientation in styrene derivatives.

## Singular results explanation based on computed Fukui´s radical susceptibilities for alkene substrates 1y and 1s

Computed studies revealed the exception of methyl acrylate where the *anti*-Markovnikov hydrobromination is always favored even in the presence of iron(II) bromide. Our calculations indicate that coordination of the C=C double-bond to the transition metal is mandatory to produce the Markovnikov product. This is the case of methyl acrylate **1y**, where the most stable isomer exhibits only coordination from the carbonyl oxygen atom. In this situation, the computed Fukui´s radical susceptibilites are similar to those computed for the non-coordinated species (**1y**).

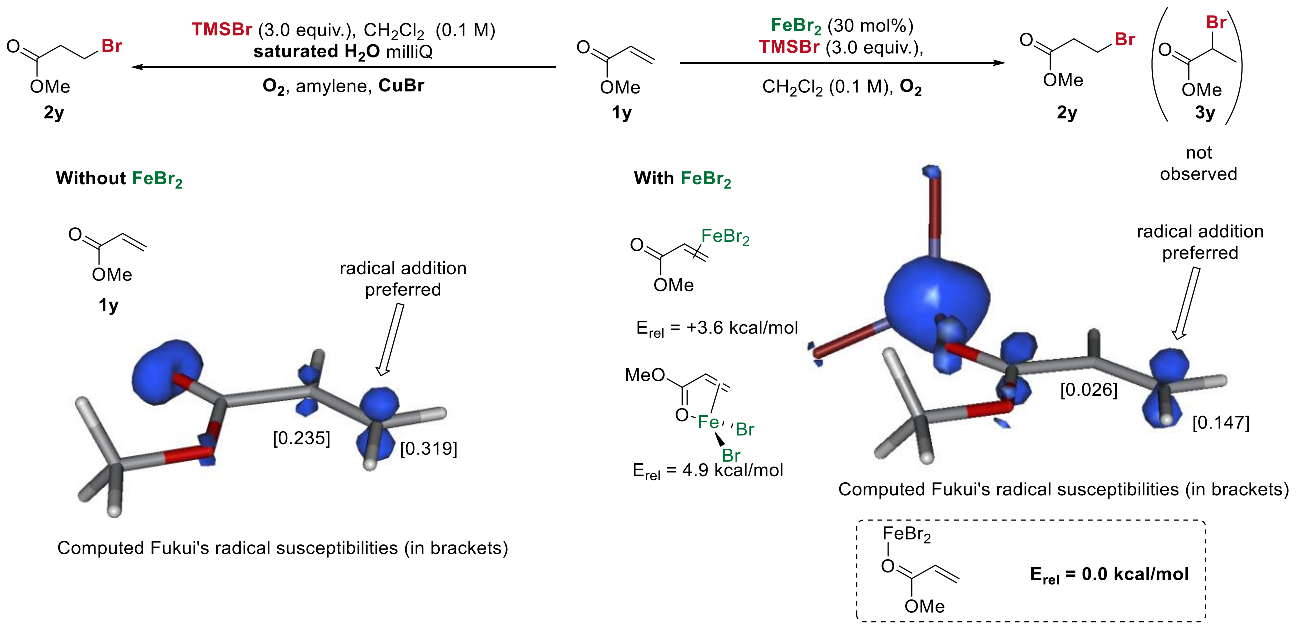


**Figure S3. Anomalous Markovnikov hydrobromination of alkene 1y**.

Fukui's radical susceptibilities showed a divergence between allylic and vinylic positions, indicating that the radical addition should occur at the allylic position, which is in agreement with the experimental data observed.

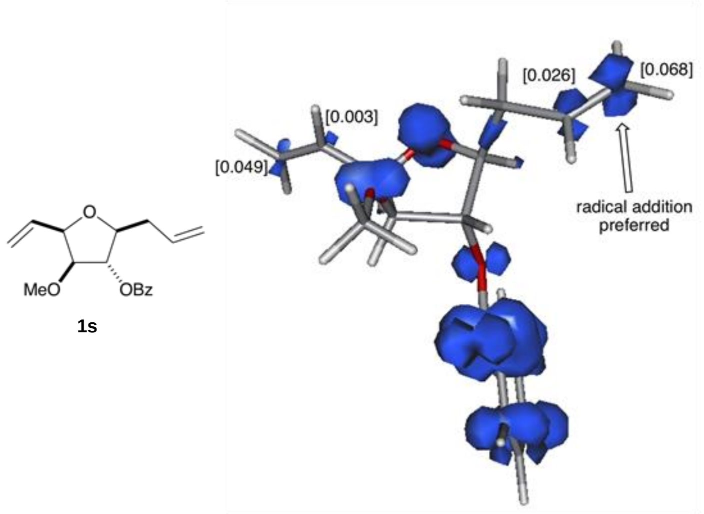


**Figure S4. Computed Fukui´s radical susceptibilities for compound 1s**. Computed Fukui´s radical susceptibilities show the allylic moiety as the preferential position for the radical addition.

## Experimental procedure and characterization data for starting materials

1-undecene (**1a**), 3-buten-1-ol (**1b**), Allylcyclopentane (**1h**), Allylbenzene (**1i**), *trans*-β-methylstyrene (**1j**), Ethyl acrylate (**1t**), Diethyl allylmalonate (**1u**), 2-allylcyclohexanone (**2w**) were purchased from Aldrich® and used without any further purification.

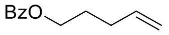

**pent-4-en-1-yl benzoate (1c):** To a 0.1 M solution of 5-penten-1-ol (3.48 mmol.) in dry DCM (0.1 M), were sequentially added benzoyl chloride BzCl (5.2 mmol, 1.5 equiv.), $Et_3N$ (5.22 mmol, 1.5 equiv.) and a catalytic amount of *N,N*-dimethylaminopyridine (DMAP). The reaction was followed by TLC, and when the reaction reached complete conversion water was added, and the reaction mixture was extracted with DCM. The combined organic layers were dried over magnesium sulphate anhydrous, filtered and the solvent was removed under reduced pressure. The crude reaction mixture was purified by flash silica gel column chromatography (*n*-hexane/EtOAc 95:5), to obtain 660 mg of **1c** (99%). Spectroscopic data match with those reported in literature.[32]

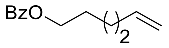

**hex-4-en-1-yl benzoate (1d):** To a 0.1 M solution of 5-hexen-1-ol (10.0 mmol.) in dry DCM (0.1 M), were sequentially added benzoyl chloride BzCl (15.0 mmol, 1.5 equiv.), $Et_3N$ (15.0 mmol, 1.5 equiv.) and a catalytic amount of *N,N*-dimethylaminopyridine (DMAP). The reaction was followed by TLC, and when the reaction reached complete conversion water was added, and the reaction mixture was extracted with DCM. The combined organic layers were dried over magnesium sulphate anhydrous, filtered and the solvent was removed under reduced pressure. The crude reaction mixture was purified by flash silica gel column chromatography (*n*-hexane/EtOAc 95:5), to obtain 2 g of **1d** (99%). Spectroscopic data match with those reported in literature.[33]

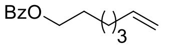

**hept-4-en-1-yl benzoate (1e):** To a 0.1 M solution of 7-hepten-1-ol (0.175 mmol.) in DCM, were sequentially added benzoyl chloride (2.63 mmol, 1.5 equiv.), $Et_3N$ (2.63 mmol, 1.5 equiv.) and a catalytic amount of *N,N*-dimethylaminopyridine (DMAP). The reaction was followed by TLC, and when the reaction reached complete conversion water was added, and the reaction mixture was extracted with DCM. The combined organic layers were dried over magnesium sulphate anhydrous, filtered and the solvent was removed under reduced pressure. The crude reaction mixture was purified by flash silica gel column chromatography (*n*-hexane/EtOAc 95:5), to obtain 380 mg of **1e** (99%).
$^1$H-NMR ($CDCl_3$, 400 MHz): δ 8.05 (d, *J* = 7.6 *Hz*, 2H), 7.55 (t, *J* = 7.5 *Hz*, 1H), 7.44 (m, 2H), 5.81 (ddt, *J* = 6.7, 10.2 *&* 17.0 *Hz*, 1H), 5.05-4.92 (m, 2H), 4.32 (t, *J* = 7.6 *Hz*, 2H), 2.10 (m, 2H), 1.78 (m, 2H), 1.47 (m, 4H). $^{13}$C-NMR ($CDCl_3$, 100 MHz): δ 166.7 (C), 138.7 (CH), 132.8 (CH), 129.5 (2 x CH), 128.3 (2 x CH), 114.5 ($CH_2$), 65.0 ($CH_2$), 33.6 ($CH_2$), 28.6 ($CH_2$), 28.5 ($CH_2$), 25.5 ($CH_2$). HRMS ($ESI^+$): *m/z* $[M+Na]^+$ calcd. for $C_{14}H_{18}O_2$: 241.1204; found: 241.1198.

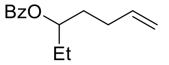

**hept-6-en-3-yl-benzoate (1f):** To a 0.1 M solution of 6-hepten-3-ol (8.8 mmol.) in dry DCM (0.1 M), were sequentially added benzoyl chloride BzCl (13.2 mmol, 1.5 equiv.), $Et_3N$ (13.2 mmol, 1.5 equiv.) and a catalytic amount of *N,N*-dimethylaminopyridine (DMAP). The reaction was followed by TLC, and when the reaction reached complete conversion water was added, and the reaction mixture was extracted with DCM. The combined organic layers were dried over magnesium sulphate anhydrous, filtered and the solvent was removed under reduced pressure. The crude reaction mixture was purified by flash silica gel column chromatography (*n*-hexane/EtOAc 95:5), to obtain 1.6 g of **1f** (84%).

$^1$H-NMR ($CDCl_3$, 400 MHz): δ 8.06 (d, *J* = 8.5 *Hz*, 2H), 7.56 (tt, *J* = 1.3 & 7.4 *Hz*, 1H), 7.44 (t, *J* = 7.6 *Hz*, 2H), 5.83 (ddt, *J* = 6.6, 10.3 & 17.0 *Hz*, 1H), 5.16-5.06 (m, 1H), 5.06-4.92 (m, 2H), 2.15 (m, 2H), 1.88-1.66 (m, 4H), 0.96 (t, *J* = 7.5 *Hz*, 3H). $^{13}$C-NMR ($CDCl_3$, 100 MHz): δ 166.3 (C), 137.9 (CH), 132.7 (CH), 130.8 (C), 129.5 (2 x CH), 128.3 (2 x CH), 114.9 ($CH_2$), 75.6 (CH), 32.9 ($CH_2$), 29.6 ($CH_2$), 27.1 ($CH_2$), 9.5 ($CH_3$). HRMS ($ESI^+$): *m/z* $[M+Na]^+$ calcd. for $C_{14}H_{18}O_2$: 241.1204; found: 241.1202.

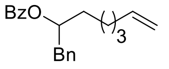


**1-phenyloct-7-en-2-yl benzoate (1g):** To a 0.1 M solution of 1-phenyloct-7-en-2-ol (0.73 mmol.) in dry DCM (0.1 M), were sequentially added benzoyl chloride BzCl (1.10 mmol, 1.5 equiv.), $Et_3N$ (1.10 mmol, 1.5 equiv.) and a catalytic amount of *N,N*-dimethylaminopyridine (DMAP). The reaction was followed by TLC, and when the reaction reached complete conversion water was added, and the reaction mixture was extracted with DCM. The combined organic layers were dried over magnesium sulphate anhydrous, filtered and the solvent was removed under reduced pressure. The crude reaction mixture was purified by flash silica gel column chromatography (*n*-hexane/EtOAc 95:5), to obtain 200 mg of **1g** (91%).

$^1$H-NMR ($CDCl_3$, 400 MHz): δ 8.02 (dd, *J* = 1.2 *&* 8.3 *Hz*, 2H), 7.55 (tt, *J* = 1.3 *&* 7.4 *Hz*, 1H), 7.44 (m, 2H), 7.31-7.16 (m, 5H), 5.76 (ddt, *J* = 6.7, 10.3 *&* 17.1 *Hz*, 1H), 5.32 (m, 1H), 5.00-4.93 (ddd, *J* = 1.6, 3.6 *&* 17.1 *Hz*, 1H), 4.93-4.88 (m, 1H), 3.04 (dd, *J* = 6.4 *&* 13.8 *Hz*, 1H), 2.93 (dd, *J* = 6.3 *&* 13.8 *Hz*, 1H), 2.02 (m, 2H), 1.68 (m, 2H), 1.48-1.34 (m, 4H). $^{13}$C-NMR ($CDCl_3$, 100 MHz): δ 166.1 (C), 138.7 (CH), 137.6 (C), 132.7 (CH), 130.7 (C), 129.5 (4 x CH), 128.4 (2 x CH), 128.3 (2 x CH), 126.4 (CH), 114.4 ($CH_2$), 75.4 (CH), 40.6 ($CH_2$), 33.5 ($CH_2$), 33.2 ($CH_2$), 28.7 ($CH_2$), 24.8 ($CH_2$). HRMS ($ESI^+$): *m/z* $[M+Na]^+$ calcd. for $C_{21}H_{24}O_2$: 331.1674; found: 331.1674.

.

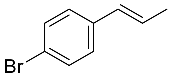


**(*E*)-1-bromo-4-(prop-1-en-1-yl)benzene (1k):** Ethyltriphenylphosphonium bromide (12.5 mmol., 1.50 equiv.) was charged to a flame dried 250 ml round bottom flask after cooling under nitrogen. The solvent (25 mL diethyl ether, anhydrous) was added and the mixture was stirred at 0 ºC while potassium tertbutoxide (12.5 mmol., 1.5 equiv.) was added in several small portions. The mixture turned yellow/orange and was stirred for 30 minutes before 4-bromobenzaldehyde (8.32 mmol.) was added dropwise at 0 ºC. The mixture was allowed to warm slowly to ambient temperature with stirring for 3-12 hours. Upon completion the reaction mixture was quenched by the addition of saturated aqueous ammonium chloride. The organic material was extracted into diethyl ether (3 x 100 mL), washed with saturated aqueous sodium chloride, dried with anhydrous sodium sulfate, and concentrated under vacuum. The final styrenyl derivatives were purified by vacuum distillation or column chromatography (*n*-hexane/EtOAc 95:5) to afford 1.32 g. of **1k** (81%). Spectroscopic data match with those reported in literature.[34]

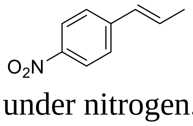


**(*E*)-1-nitro-4-(prop-1-en-1-yl)benzene (1l):** Ethyltriphenylphosphonium bromide (12.5 mmol., 1.50 equiv) was charged to a flame dried 250 ml round bottom flask after cooling under nitrogen. The solvent (25 mL diethyl ether, anhydrous) was added and the mixture was stirred at 0 ºC while potassium tertbutoxide (12.5 mmol., 1.5 equiv.) was added in several small portions. The mixture turned yellow/orange and was stirred for 30 minutes before 4-bromobenzaldehyde (8.32 mmol.) was added dropwise at 0 ºC. The mixture was allowed to warm slowly to ambient temperature with stirring for 3-12 hours. Upon completion the reaction mixture was quenched by the addition of saturated aqueous ammonium chloride. The organic material was extracted into diethyl ether (3 x 100 mL), washed with

saturated aqueous sodium chloride, dried with anhydrous sodium sulfate, and concentrated under vacuum. The final styrenyl derivatives were purified by vacuum distillation or column chromatography (*n*-hexane/EtOAc 95:5) to afford 640 mg. of **1l** (47%). Spectroscopic data match with those reported in literature.[35]

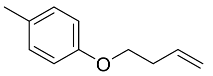

**1-(but-3-en-1-yloxy)-4-methylbenzene (1m):** To a solution of 4-methylphenol (10.6 mmol.) in dry THF (0.1 M) were sequencially added 3-buten-1-ol (11.7 mmol., 1.1 equiv.), triphenylphosphine (11.7 mmol., 1.1 eq) and DIAD (11.7 mmol., 1.1 eq). The reaction was followed by TLC, and when it reached complete conversion, it was extracted with DCM (3x 25 mL) and water. The organic phase was dried with magnesium sulphate, evaporate to dryness and purified by column chromatography using *n*-hexane/AcOEt (95:5) as eluents, to obtain 1.02 g of **1m** (60%). Spectroscopic data match with those reported in literature.[36]

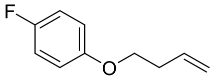

**1-(but-3-en-1-yloxy)-4-fluorobenzene (1n):** To a solution of 4-fluorophenol (10.6 mmol.) in dry THF (0.1 M) were sequencially added 3-buten-1-ol (11.7 mmol., 1.1 equiv.), triphenylphosphine (11.7 mmol., 1.1 eq) and DIAD (11.7 mmol., 1.1 eq). The reaction was followed by TLC, and when it reached complete conversion, it was extracted with DCM (3x 25 mL) and water. The organic phase was dried with magnesium sulphate, evaporate to dryness and purified by column chromatography using *n*-hexane/AcOEt (95:5) as eluents, to obtain 1.46 g of **1n** (83%). $^1$H-NMR ($CDCl_3$, 400 MHz): δ 6.96 (m, 2H), 6.84 (dd, $J$ = 4.3 *&* 9.1 *Hz*, 2H), 5.90 (ddt, $J$ = 6.7, 10.3 *&* 17.1 *Hz*, 1H), 5.17 (ddd, $J$ = 1.6, 3.3 *&* 17.2 *Hz*, 1H), 5.11 (ddd, $J$ = 1.1, 2.7 *&* 10.2 *Hz*, 1H), 3.98 (t, $J$ = 6.8 *Hz*, 2H), 2.53 (ct, $J$ = 1.3 *&* 6.6 *Hz*, 2H). $^{13}$C-NMR ($CDCl_3$, 100 MHz): δ 157.2 (C, $J$ = 238.3 *Hz*), 155.0 (C, $J$ = 1.8 *Hz*), 134.3 (CH), 117.1 ($CH_2$), 115.7 (2 x CH, $J$ = 23.0 *Hz*), 115.6 (2 x CH, $J$ = 7.7 *Hz*), 67.9 ($CH_2$), 33.6 ($CH_2$). HRMS ($ESI^+$):*m/z* $[M+Na]^+$ calcd. for $C_{10}H_{11}FO$: 189.0686; found: 189.0677.

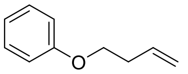

**(but-3-en-1-yloxy)benzene (1o):** To a solution of 4-phenol (10.6 mmol.) in dry THF (0.1 M) were sequencially added 3-buten-1-ol (11.7 mmol., 1.1 equiv.), triphenylphosphine (11.7 mmol., 1.1 eq) and DIAD (11.7 mmol., 1.1 eq). The reaction was followed by TLC, and when it reached complete conversion, it was extracted with DCM (3x 25 mL) and water. The organic phase was dried with magnesium sulphate, evaporate to dryness and purified by column chromatography using *n*-hexane/AcOEt (95:5) as eluents, to obtain 1.26 g of **1o** (80%). Spectroscopic data match with those reported in literature.

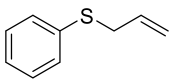

A**llyl(phenyl)sulfane (1p):** To a stirred solution of K2CO3 (4.1 g, 30 mmol, 1 equiv) in dry ethanol (50 mL) was added thiophenol (3 mL, 30 mmol, 1 equiv) followed by allyl bromide (10 mL, 120 mmol, 4 equiv). The reaction was vigorously stirred at room temperature overnight. Then, it was filtered through a pad of Celite® and evaporated to dryness. The orange residue was purified by column chromatography using *n*-pentane as eluent to afford 4.0 g of pure **1p** as a pale yellow liquid (88%). Spectroscopic data match with those reported in literature.[37]

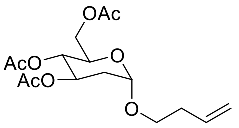

**(*2R, 3S, 4R, 6S*)-2-(acetoxymethyl)-6-(but-3-en-1-yloxy)tetrahydro-*2H*-pyran-3,4-diyl diacetate (1q):** To a solution of tri-*O*-acetyl-D-glucal (3.67 mmol.) in dry DCM (0.05 M), 3-buten-1-ol (7.35 mmol., 2.0 equiv.) was added followed by triphenylphosphine hydrobromide (1.80 mmol., 0.5 equiv.). The reaction was stirred overnight and then it was quenched by the addition of water and extracted with DCM (3 x 20 mL). The

organic phase was dried over magnesium sulphate and evaporated under reduced pressure. The remaining solid was purified by column chromatography using *n*-hexane/AcOEt (7:3) as eluent to obtain 800 mg of **1q** (63%).

$^1$H-NMR ($CDCl_3$, 400 MHz): δ 5.81 (ddt, *J* = 6.8, 10.3 & 17.1 *Hz*, 1H), 5.31 (ddd, *J* = 5.4, 9.4 & 11.6 *Hz*, 1H), 5.15-4.92 (m, 4H), 4.29 (dd, *J* = 4.7 & 12.2 *Hz*, 1H), 4.05 (dd, *J* = 2.3 & 12.2 *Hz*, 1H), 3.97 (ddd, *J* = 2.3, 4.6 & 10.1 *Hz*, 1H), 3.66 (dt, *J* = 6.9 & 9.7 *Hz*, 1H), 3.46 (dt, *J* = 6.7 & 9.6 *Hz*, 1H), 2.34 (ddt, *J* = 1.2, 6.7 & 13.5 *Hz*, 2H), 2.23 (ddd, *J* = 1.2, 5.3 & 12.9 *Hz*, 1H), 2.08 (s, 3H), 2.03 (s, 3H), 2.00 (s, 3H), 1.81 (ddd, *J* = 3.7, 11.7 & 12.9 *Hz*, 1H). $^{13}$C-NMR ($CDCl_3$, 100 MHz): δ 170.7 (C), 170.2 (C), 169.9 (C), 134.8 (CH), 116.8 ($CH_2$), 96.9 (CH), 69.5 (CH), 69.1 (CH), 67.9 (CH), 67.2 ($CH_2$), 62.4 ($CH_2$), 35.0 ($CH_2$), 33.8 ($CH_2$), 21.0 ($CH_3$), 20.7 (2 x $CH_3$). HRMS (ESI$^+$):*m/z* $[M+Na]^+$ calcd. for $C_{16}H_{24}O_8$: 367.1369; found: 367.1372.

$[\alpha]_D^{25}$: +109.2 (*c* = 1.02, $CHCl_3$).

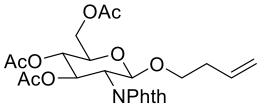


**(2*R*, 3*S*, 4*R*, 5*R*, 6*R*)-2-(acetoxymethyl)-6-(but-3-en-1-yloxy) -5-phthalimidyl-tetrahydro-2*H*-piran-3,4-diyl diacetate (1r)**: To a solution of 1,3,4,6-tetra-*O*-acetyl-2-deoxy-2-phthalimido-β-D-glucopyranose (0.65 mmol.) in dry DCM (0.1 M) were added 3-buten-1-ol (0.65 mmol., 1.0 equiv.) and $BF_3 \cdot Et_2O$ (0.65 mmol., 1.0 equiv.). The reaction was stirred at room temperature overnight, and it was quenched by additioning water. The reaction was extracted with DCM, and the organic phase was dried over magnesium sulphate, and evaporated under reduced pressure. The remaining oil was purified by column chromatography using *n*-hexane/AcOEt (6:4) as eluents, to obtain 240 mg. of **1r**. (77%)

$^1$H-NMR ($CDCl_3$, 400 MHz):δ 7.85 (dd, *J* = 3.1 & 5.6 *Hz*, 2H), 7.74 (dd, *J* = 3.1 & 5.5 *Hz*, 2H), 5.79 (dd, *J* = 9.1 & 10.8 *Hz*, 1H), 5.51 (ddt, *J* = 6.7, 10.3 & 17.1 *Hz*, 1H), 5.36 (d, *J* = 8.5 *Hz*, 1H), 5.17 (dd, *J* = 9.2 & 10.1 *Hz*, 1H), 4.81 (dc, *J* = 1.5 & 17.2 *Hz*, 1H), 4.62 (d, *J* = 10.3 *Hz*, 1H), 4.32 (m, 2H), 4.17 (dd, *J* = 2.4 & 12.2 *Hz*, 1H), 3.88 (m, 2H), 3.49 (dt, *J* = 6.9 & 9.7 *Hz*, 1H), 2.18 (m, 2H), 2.11 (s, 3H), 2.03 (s, 3H), 1.86 (s, 3H). $^{13}$C-NMR ($CDCl_3$, 100 MHz): δ 170.7 (C), 170.1 (C), 169.5 (C), 134.3 (CH), 134.2 (CH), 131.5 (C), 123.5 (CH), 116.5 ($CH_2$), 98.2 (CH), 71.9 (CH), 70.8 (CH), 69.2 ($CH_2$), 69.1 (CH), 62.1 ($CH_2$), 54.6 (CH), 33.6 ($CH_2$), 20.7 ($CH_3$), 20.6 ($CH_3$), 20.4 ($CH_3$). HRMS (ESI$^+$): *m/z* $[M+Na]^+$ calcd. for $C_{24}H_{27}NO_{10}$: 512.1533; found: 512.1528.

$[\alpha]_D^{25}$: +16.4 (*c* = 1.05, $CHCl_3$).

**Figure S5.** *Synthesis of **1s**.*

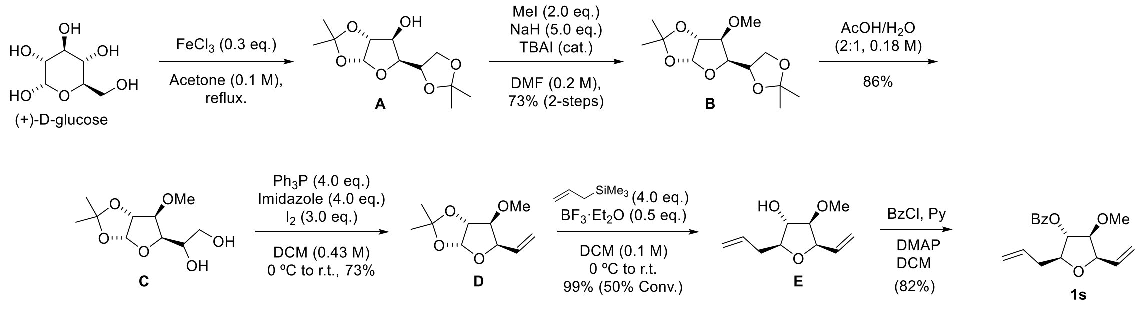

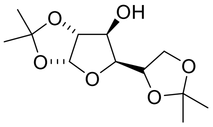

**(3a*R*,5*S*,6*S*,6a*R*)-5-((*S*)-2,2-dimethyl-1,3-dioxolan-4-yl)-2,2-dimethyltetrahidrofuro[2,3-*d*][1,3]-dioxol-6-ol (A):** Following the procedure reported by Srivastava et al.,[38] to a solution of (+)-*D*-glucose (27.75 mmol.) in dry acetone (0.1 M) was added $FeCl_3$ (9.0 mmol.) and the mixture was refluxed for 5 hours. Then, a 10% aqueous solution of $K_2CO_3$ was added, and the acetone was evaporated under reduced pressure. The remaining mixture was extracted with DCM, the organic phase dried over magnesium sulphate and evaporated to afford the product **A** as a crude which was used directly for the next step without any further purification.

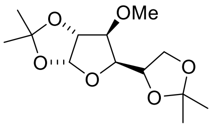

**(3a*R*,5*R*,6*S*,6a*R*)-5-((*S*)-2,2-dimethil-1,3-dioxolan-4-yl)-6-methoxy-2,2-dimethyltetrahydrofuro[2,3-*d*][1,3]-dioxol (B):** Following the reported procedure by Chang, C. T.,[39] to a solution of the alcohol **A** in dry DMF (0.2 M) were added MeI (55.5 mmol., 2.0 equiv.) and sodium hydride (139 mmol., 5.0 equiv.) and a catalytic amount of tetrabutylammonium iodide (TBAI). The reaction was stirred at r.t. overnight, and it was extracted with DCM. The crude reaction mixture was purified by column chromatography using *n*-hexane/AcOEt (8:2) to afford 5.6 g (20.14 mmol.) of product **B** (73% over two steps). Spectroscopic data match with those reported in the literature.[40]

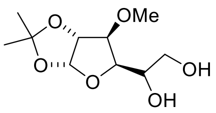

**(*S*)-1-((3a*R*,5*R*,6*S*,6a*R*)-6-methoxy-2,2-dimethyltetrahydrofuro[2,3-*d*][1,3]-dioxol-5-yl)ethane-1,2-diol (C):** Acetonide **B** (18.85 mmol.) was dissolved in a mixture of AcOH:$H_2O$ (2:1, 0.18 M), and it was stirred at r. t. for two days. Then, the mixture was cooled down to 0 ºC, and it was quenched with the addition of an aqueous KOH solution (500 mL, 2 M), in order to adjust the pH = 6. Afterwards, the pH was readjusted to nearly 7 by small portions of $Na_2CO_3$. The water was eliminated under reduced pressure, and the remaining mixture was extracted with DCM and EtOAc (5 x 25 mL), and evaporated under reduced to afford 3.80 g (16.21 mmol.) of crude diol **C** (86%), which were used without any further purification.

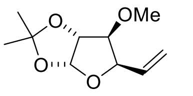

**(3a*R*,5*R*,6*S*,6a*R*)-6-methoxy-2,2-dimethyl-5-vinyltetrahydrofuro[2,3-*d*][1,3]-dioxol (D):** Following the procedure reported by Sharma, G. V. M.,[41] to a solution of diol **C** (7.68 mmol.) in dry DCM (0.43 M) at 0 ºC, it was added triphenylphosphine (30.74 mmol., 4.0 equiv.), imidazole (30.74 mmol., 4.0 equiv.) and iodine (23.05 mmol., 3.0 equiv.). The reaction was stirred at room temperature for 2 hours, and then it was quenched by carefully adding a saturated aqueous solution of NaOH until the reaction mixture turns clear. Then, it was extracted with DCM and EtOAc (3 x 25 mL) and washed with brine. The combined organic layers were dried over $MgSO_4$ and evaporated under reduced pressure. The remaining oil was purified by column chromatography using *n*-hexane/AcOEt (95:5) to afford 1.13 g (5.64 mmol.) of product **D** (73%). Spectroscopic data match with those reported in the literature.[42]

$[\alpha]_D^{25}$: -76.8 ($c$ = 1.16, $CHCl_3$).

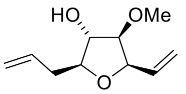

**(2*S*,3*S*,4*R*,5*R*)-2-allyl-4-methoxy-5-vinyltetrahydrofuran-3-ol (E):** Following the procedure reported by Tellado et al.,[43] to a solution of isopropiliden **D** (14.6 mmol.) in dry DCM (0.1 M) at 0 ºC, it was added allyl-TMS (58.4 mmol., 4.0 equiv.) and $BF_3{\cdot}Et_2O$ (7.31 mmol., 0.5 equiv.) dropwise. The reaction mixture was allowed to reach to r.t. and it was stirred overnight. Then, it was quenched with the addition of 3 x HCl (1 M), 3 x $NaHCO_3$ and 3 x NaCl. The aqueous phases were extracted with EtOAc, dried over magnesium sulphate and evaporated under reduced pressure. The crude reaction mixture was purified by column chromatography using *n*-

hexane/AcOEt (7:3) to afford 1.40 g (7.6 mmol.) (99% yield, 50% conversion). The starting material unreacted was recovered and submitted again to the reaction conditions.
$^{1}$H-NMR ($CDCl_3$, 400 MHz): δ 6.01-5.90 (ddd, *J* = 7.2, 10.5 *&* 17.5 *Hz*, 1H), 5.90-5.81 (ddt, *J* = 7.1, 10.1 *&* 17.2 *Hz*, 1H), 5.42-5.23 (m, 2H), 5.20-5.07 (m, 2H), 4.45 (dd, *J* = 5.3 *&* 7.1 *Hz*, 1H), 3.99 (dd, J = 3.0 & 5.0 Hz, 1H), 3.75-3.69 (dt, *J* = 5.1 *&* 6.7 *Hz*, 1H), 3.69-3.66 (dd, *J* = 3.0 *&* 5.1 *Hz*, 1H), 3.38 (s, 3H), 2.54-2.35 (m, 2H), 1.93 (brs, 1H). $^{13}$C-NMR ($CDCl_3$, 100 MHz): δ 134.2 (CH), 133.8 (CH), 118.2 ($CH_2$), 117.6 ($CH_2$), 88.9 (CH), 83.2 (CH), 81.3 (CH), 79.2 (CH), 57.9 ($CH_3$), 38.0 ($CH_2$). HRMS ($ESI^+$): *m/z* $[M+Na]^+$ calcd. for $C_{10}H_{16}O_3$: 207.0997; found: 207.1001.
$[\alpha]_D^{25}$: -30.8 (*c* = 1.20, $CHCl_3$).

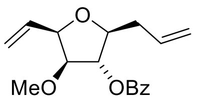

**(2*S*,3*S*,4*R*,5*R*)-2-allyl-4-methoxy-5-vinyltetrahydrofuran-3-yl benzoate (1s):** To a 0.1 M solution of **E** (5.58 mmol.) in dry DCM (0.1 M), were sequentially added benzoyl chloride (BzCl) (16.7 mmol, 3.0 equiv.), $Et_3N$ (16.7 mmol, 3.0 equiv.) and a catalytic amount of *N,N*-dimethylaminopyridine (DMAP). The reaction was followed by TLC, and when the reaction reached complete conversion water was added, and the reaction mixture was extracted with DCM. The combined organic layers were dried over magnesium sulphate anhydrous, filtered and the solvent was removed under reduced pressure. The crude reaction mixture was purified by flash silica gel column chromatography *n*-hexane/EtOAc (85:15), to obtain 1.3 g (4.51 mmol.) of **1s** (81%).
$^{1}$H-NMR ($CDCl_3$, 400 MHz): δ 8.04 (m, 2H), 7.60 (tt, *J* = 1.3 *&* 7.5 *Hz*, 1H), 7.47 (t, *J* = 7.6 *Hz*, 2H), 6.03 (ddd, *J* = 7.1, 10.4 *&* 17.4 *Hz*, 1H), 5.89 (ddt, *J* = 7.0, 10.2 *&* 17.1 *Hz*, 1H), 5.47-5.29 (m, 2H), 5.25 (dd, *J* = 0.9 *&* 2.8 *Hz*, 1H), 5.22-5.13 (ddd, *J* = 1.5, 3.3 *&* 17.1 *Hz*, 1H), 5.12-5.06 (m, 1H), 4.47 (dd, J = 3.8 & 7.0 Hz, 1H), 4.09 (td, *J* = 2.8 *&* 6.8 *Hz*, 1H), 3.77 (dd, *J* = 0.8 *&* 3.8 *Hz*, 1H), 3.49 (s, 3H), 2.56 (m, 2H). $^{13}$C-NMR ($CDCl_3$, 100 MHz): δ 165.6 (C), 134.1 (CH), 133.4 (CH), 133.0 (CH), 129.7 (2 x CH), 128.5 (2 x CH), 118.6 ($CH_2$), 117.5 ($CH_2$), 86.8 (CH), 82.7 (2 x CH), 79.9 (CH), 57.9 ($CH_3$), 38.2 ($CH_2$). HRMS ($ESI^+$): *m/z* $[M+Na]^+$ calcd. for $C_{17}H_{20}O_4$: 311.1259; found: 311.1256.
$[\alpha]_D^{25}$: -29.2 (*c* = 1.05, $CHCl_3$).

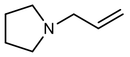

**1-allylpyrrolidine (1v):** Allyl bromide (1 mL, 1.1 equiv) was added to a stirred mixture of pyrrolidine (2.1 mL, 2 equiv) and dry diethyl ether (5 mL). The reaction was stirred at room temperature overnight, and the precipitate formed was removed by filtration. The filtrate was evaporated to dryness to afford 1 g (9.0 mmol.) of **1v** (70%). Spectroscopic data match with those reported in the literature.[44]

## Characterization data for *anti*-Markovnikov hydrobromination products

The reactions were carried out following the general procedure **1** described above.

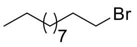

**1-bromoundecane (2a):** Following General Procedure **1**, to a solution of 1-undecene (0.25 mL, 1.2 mmol.) in water saturated DCM (12 mL), was added TMSBr (0.47 mL, 3.6 mmol.) to afford 280 mg (1.2 mmol.) of **2a** as a colorless oil (>99%). Spectroscopic data match with those reported in the literature.[45]
$^{1}$H-NMR ($CDCl_3$, 400 MHz): δ 3.41 (t, *J* = 6.9 *Hz*, 2H), 1.85 (m, 2H), 1.42 (m, 2H), 1.32-1.25 (m, 14H), 0.88 (t, *J* = 6.8 *Hz*, 3H).

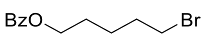


**5-bromopentyl benzoate (2c):** Following General Procedure **1**, to a solution of **1c** (95 mg, 0.5 mmol.) in water saturated DCM (containing 20 ppm of amylene or 5 ppm of 1,5-cyclooctadiene) (5 mL), was added TMSBr (0.20 mL, 1.5 mmol.) to afford 120 mg (0.44 mmol.) of **2c** as a pale yellow oil (88%). Spectroscopic data match with those reported in the literature.[46]

$^1$H-NMR ($CDCl_3$, 400 MHz): δ 8.04 (m, 2H), 7.56 (m, 1H), 7.44 (m, 2H), 4.34 (t, *J* = 6.5 *Hz*, 2H), 3.44 (t, *J* = 6.7 *Hz*, 2H), 1.95 (m, 2H), 1.81 (m, 2H), 1.62 (m, 2H).

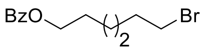


**6-bromohexyl benzoate (2d):** Following General Procedure **1**, to a solution of **1d** (100 mg, 0.5 mmol.) in water saturated DCM (containing 20 ppm of amylene or 5 ppm of 1,5-cyclooctadiene) (5 mL), was added TMSBr (0.20 mL, 1.5 mmol.) to afford 140 mg (0.49 mmol.) of **2d** as a pale yellow oil (98%).

$^1$H-NMR ($CDCl_3$, 400 MHz): δ 8.04 (dd, *J* = 1.3 & 8.4 *Hz*, 2H), 7.56 (tt, *J* = 1.2 & 7.4 *Hz*, 1H), 7.44 (t, *J* = 7.7 *Hz*, 2H), 4.33 (t, *J* = 6.6 *Hz*, 2H), 3.42 (t, *J* = 6.7 *Hz*, 2H), 1.95-1.84 (m, 2H), 1.84-1.74 (m, 2H), 1.56-1.43 (m, 4H).

$^{13}$C-NMR ($CDCl_3$, 100 MHz):δ 166.6 (C), 132.8 (CH), 130.4 (C), 129.5 (2 x CH), 128.3 (2 x CH), 64.8 ($CH_2$), 33.7 ($CH_2$), 32.6 ($CH_2$), 28.6 ($CH_2$), 27.8 ($CH_2$), 25.3 ($CH_2$).

HRMS (ESI$^+$): *m/z* $[M+Na]^+$ calcd. for $C_{13}H_{17}O_2Br$: 307.0310; found: 307.0305.

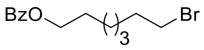


**7-bromoheptyl benzoate (2e):** Following General Procedure **1**, to a solution of **1e** (110 mg, 0.5 mmol.) in water saturated DCM (containing 20 ppm of amylene or 5 ppm of 1,5-cyclooctadiene) (5 mL), was added TMSBr (0.20 mL, 1.5 mmol.) to afford 120 mg (0.40 mmol.) of **2e** as a pale yellow oil (80%). Spectroscopic data match with those reported in the literature.[47]

$^1$H-NMR ($CDCl_3$, 400 MHz): δ 8.04 (m, 2H), 7.56 (m, 1H), 7.44 (m, 2H), 4.32 (t, *J* = 6.6 *Hz*, 2H), 3.41 (t, *J* = 6.9 *Hz*, 2H), 1.87 (m, 2H), 1.78 (dt, *J* = 6.8 & 14.8 *Hz*, 2H), 1.51-1.43 (m, 4H), 1.43-1.37 (m, 2H).

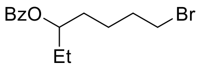


**7-bromoheptan-3-yl benzoate (2f):** Following General Procedure **1**, to a solution of **1f** (110 mg, 0.5 mmol.) in water saturated DCM (containing 20 ppm of amylene or 5 ppm of 1,5-cyclooctadiene) (5 mL), was added TMSBr (0.20 mL, 1.5 mmol.) to afford 124 mg (0.414 mmol.) of **2f** as a pale yellow oil (83%).

$^1$H-NMR ($CDCl_3$, 400 MHz): δ 8.05 (dd, *J* = 1.4 & 2.5 *Hz*, 2H), 7.56 (tt, *J* = 1.2 & 7.4 *Hz*, 1H), 7.44 (t, *J* = 7.7 *Hz*, 2H), 5.09 (dt, *J* = 6.2 & 12.4 *Hz*, 1H), 3.40 (t, *J* = 6.8 *Hz*, 2H), 1.97-1.82 (m, 2H), 1.78-1.63 (m, 4H), 1.61-1.46 (m, 2H), 0.96 (t, *J* = 7.5 *Hz*, 3H).$^{13}$C-NMR ($CDCl_3$, 100 MHz): δ 166.4 (C), 132.8 (CH), 130.7 (C), 129.5 (2 x CH), 128.3 (2 x CH), 75.7 (CH), 33.5 ($CH_2$), 32.8 ($CH_2$), 32.5 ($CH_2$), 27.0 ($CH_2$), 23.9 ($CH_2$), 9.6 ($CH_3$). HRMS (ESI$^+$): *m/z* $[M+Na]^+$ calcd. for $C_{14}H_{19}O_2Br$: 321.0466; found: 321.0477.

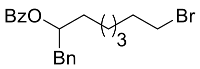


**8-bromo-1-phenyloctan-2-yl benzoate (2g):** Following General Procedure **1**, to a solution of **1g** (150 mg, 0.49 mmol.) in water saturated DCM (containing 20 ppm of amylene or 5 ppm of 1,5-cyclooctadiene) (5 mL), was added TMSBr (0.19 mL, 1.47 mmol.) to afford 130 mg (0. 33 mmol.) of **2g** as a pale yellow oil (67%).

$^1$H-NMR ($CDCl_3$, 400 MHz): δ 8.07 (dd, *J* = 1.3 & 8.4 *Hz*, 2H), 7.61 (tt, *J* = 1.3 & 7.4 *Hz*, 1H), 7.50 (t, *J* = 8.0 *Hz*, 2H), 7.37-7.22 (m, 5H), 5.38 (m, 1H), 3.42 (t, *J* = 6.8 *Hz*, 2H), 3.04 (ddd, *J* = 6.3, 13.8 & 53.5 *Hz*, 2H), 1.87 (q, *J* = 7.0 *Hz*, 2H), 1.80-1.67 (m, 2H), 1.56-1.29 (m, 6H).$^{13}$C-NMR ($CDCl_3$, 100 MHz): δ 166.1 (C), 137.5 (CH), 132.8 (CH), 130.6 (C), 129.5 (3 x CH), 128.4 (2 x CH), 128.3 (2 x CH), 126.5

(CH), 75.3 (CH), 40.6 ($CH_2$), 33.8 ($CH_2$), 33.3 ($CH_2$), 32.6 ($CH_2$), 28.5 ($CH_2$), 28.0 ($CH_2$), 25.2 ($CH_2$). HRMS ($ESI^+$): *m/z* $[M+Na]^+$ calcd. for $C_{21}H_{25}O_2Br$: 411.0936; found: 411.0938.

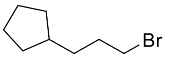


**(3-bromopropyl)ciclopentane (2h):** Following General Procedure **1**, to a solution of allylcyclopentane (0.17 mL, 1.2 mmol.) in water saturated DCM (containing 20 ppm of amylene or 5 ppm of 1,5-cyclooctadiene) (12 mL), was added TMSBr (0.47 mL, 3.6 mmol.) to afford 230 mg (1.20 mmol.) of **2h** as a colorless oil (>99%).

$^1$H-NMR ($CDCl_3$, 400 MHz): δ 3.40 (t, J = 6.9 *Hz*, 2H), 1.87 (m, 2H), 1.80-1.71 (m, 3H), 1.64-1.57 (m, 2H), 1.55-1.47 (m, 2H), 1.46-1.39 (m, 2H) 1.14-1.00 (m, 2H). $^{13}$C-NMR ($CDCl_3$, 100 MHz): δ 39.4 (CH), 34.7 ($CH_2$), 34.3 ($CH_2$), 32.6 (2 x $CH_2$), 32.2 ($CH_2$), 25.1 (2 x $CH_2$). HRMS ($EI^+$): m/z $[M]^+$ calcd. for $C_8H_{15}Br$: 190.0357; found: 190.0358.

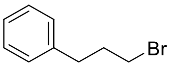


**(3-bromopropyl)benzene (2i):** Following General Procedure **1**, to a solution of allylbenzene (0.16 mL, 1.2 mmol.) in water saturated DCM (containing 20 ppm of amylene or 5 ppm of 1,5-cyclooctadiene) (12 mL), was added TMSBr (0.47 mL, 3.6 mmol.) to afford 170 mg (0.85 mmol.) of **2i** as a pale yellow oil (71%). Spectroscopic data match with those reported in literature.[48]

$^1$H-NMR ($CDCl_3$, 400 MHz): δ 7.31 (m, 2H), 2.19 (m, 3H), 3.40 (t, *J* = 6.6 *Hz*, 2H), 2.79 (t, *J* = 7.4 *Hz*, 2H), 2.18 (dt, *J* = 6.8 & 14.6 *Hz*, 2H).

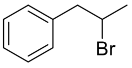


**(2-bromopropyl)benzene (2j):** Following General Procedure **1**, to a solution of trans-β-methylstyrene (0.79 mL, 0.6 mmol.) in water saturated DCM (containing 20 ppm of amylene or 5 ppm of 1,5-cyclooctadiene) (6 mL), was added TMSBr (0.24 mL, 1.8 mmol.) to afford 80 mg (0.40 mmol.) of **2j** as a pale yellow oil (67%).

$^1$H-NMR ($CDCl_3$, 400 MHz): δ 7.35-7.29 (t, J = 7.3 *Hz*, 2H), 7.29-7.23 (t, *J* = 7.26 *Hz*, 1H), 7.23-7.18 (d, *J* = 7.1 *Hz*, 2H), 4.31 (sex, *J* = 6.9 *Hz*, 1H), 3.23 (dd, *J* = 7.0 *&* 14.0 *Hz*, 1H), 3.07 (dd, *J* = 7.3 *&* 13.9 *Hz*, 1H), 1.70 (d, *J* = 6.7 *Hz*, 3H). $^{13}$C-NMR ($CDCl_3$, 100 MHz): δ 138.5 (C), 129.2 (2 x CH), 128.4 (2 x CH), 126.8 (CH), 50.5 (CH), 47.5 ($CH_2$), 25.7 ($CH_3$). HRMS ($ESI^+$): *m/z* $[M]^+$ calcd. for $C_9H_{11}Br$: 198.0044; found: 198.0048.

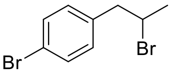


**1-bromo-4-(2-bromopropyl)benzene (2k):** Following General Procedure **1**, to a solution of **1k** (120 mg, 0.6 mmol.) in water saturated DCM (containing 20 ppm of amylene or 5 ppm of 1,5-cyclooctadiene) (6 mL), was added TMSBr (0.24 mL, 1.8 mmol.) to afford 120 mg (0.43 mmol.) of **2k** as a yellow oil (72%).

$^1$H-NMR ($CDCl_3$, 400 MHz): δ 7.44 (d, *J* = 8.3 *Hz*, 2H), 7.08 (d, *J* = 8.3 *Hz*, 2H), 4.25 (sex, *J* = 6.8 *Hz*, 1H), 3.09 (ddd, *J* = 7.5, 14.2 *&* 40.9 *Hz*, 2H), 1.70 (d, *J* = 6.6 *Hz*, 3H). $^{13}$C-NMR ($CDCl_3$, 100 MHz): δ 137.4 (C), 131.5 (2 x CH), 130.9 (2 x CH), 120.8 (C), 49.9 (CH), 46.7 ($CH_2$), 25.7 ($CH_3$). HRMS ($EI^+$): *m/z* $[M]^+$ calcd. for $C_9H_{10}Br_2$: 277.9129; found: 277.9134.

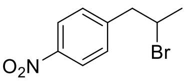


**1-(2-bromopropyl)-4-nitrobenzene (2l):** Following General Procedure **1**, to a solution of **1l** (98 mg, 0.6 mmol.) in water saturated DCM (containing 20 ppm of amylene or 5 ppm of 1,5-cyclooctadiene) (6 mL), was added TMSBr (0.24 mL, 1.8 mmol.) to afford 58 mg (0.24 mmol.) of **2l** as an intense yellow oil (40%). Spectroscopic data match with those reported in literature.[49]

$^1$H-NMR ($CDCl_3$, 400 MHz): δ 8.19 (d, *J* = 8.8 *Hz*, 2H), 7.39 (d, *J* = 8.7 *Hz*, 2H), 4.30 (sex, *J* = 6.8 *Hz*, 1H), 3.23 (d, *J* = 6.8 *Hz*, 2H), 1.76 (d, *J* = 6.6 *Hz*, 3H).

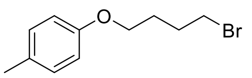

**1-(4-bromobutoxy)-4-methylbenzene (2m):** Following General Procedure **1**, to a solution of **1m** (190 mg, 1.2 mmol.) in water saturated DCM (containing 20 ppm of amylene or 5 ppm of 1,5-cyclooctadiene) (12 mL), was added TMSBr (0.47 mL, 3.6 mmol.) to afford 260 mg (1.07 mmol.) of **2m** as a colorless oil (89%). Spectroscopic data match with those reported in literature.[50]

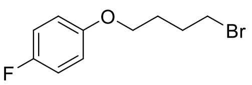

**1-(4-bromobutoxy)-4-fluorobenzene (2n):** Following General Procedure **1**, to a solution of **1n** (200 mg, 1.2 mmol.) in water saturated DCM (containing 20 ppm of amylene or 5 ppm of 1,5-cyclooctadiene) (12 mL), was added TMSBr (0.47 mL, 3.6 mmol.) to afford 260 mg (1.05 mmol.) of **2n** as a colorless oil (87%). Spectroscopic data match with those reported in literature.[51]

$^1$H-NMR ($CDCl_3$, 400 MHz): δ 6.97 (m, 2H), 6.82 (m, 2H), 3.95 (t, *J* = 6.1 *Hz*, 2H), 3.45 (t, *J* = 6.6 *Hz*, 2H), 2.07 (m, 2H), 1.93 (m, 2H).

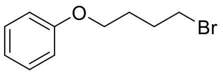

**(4-bromobutoxy)benzene (2o):** Following General Procedure **1**, to a solution of **1o** (180 mg, 1.2 mmol.) in water saturated DCM (containing 20 ppm of amylene or 5 ppm of 1,5-cyclooctadiene) (12 mL), was added TMSBr (0.47 mL, 3.6 mmol.) to afford 230 mg (1.00 mmol.) of **2o** as a colorless oil (83%). Spectroscopic data match with those reported in literature.[52]

$^1$H-NMR ($CDCl_3$, 400 MHz): δ 7.28 (m, 2H), 6.95 (m, 1H), 6.89 (m, 2H), 4.00 (t, *J* = 6.0 *Hz*, 2H), 3.50 (t, *J* = 6.6 *Hz*, 2H), 2.08 (m, 2H), 1.95 (m, 2H).

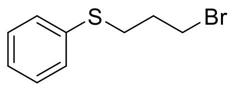

(**3-bromopropyl)-phenylsulfane (2p):** Following General Procedure **1**, to a solution of **1p** (280 mg, 1.9 mmol.) in water saturated DCM (containing 20 ppm of amylene or 5 ppm of 1,5-cyclooctadiene) (19 mL), was added TMSBr (0.75 mL, 5.5 mmol.) to afford 315 mg (1.4 mmol.) of **2p** as a yellow oil (74%).

$^1$H-NMR ($CDCl_3$, 500 MHz): δ 7.39 (t, *J* = 7 *Hz*, 2H), 7.32 (t, *J* = 7 *Hz*, 2H), 7.28 (t, *J* = 7 *Hz*, 1H), 3.56 (t, *J* = 6 *Hz*, 2H), 3.10 (t, *J* = 7 *Hz*, 2H), 2.18 (quint, *J* = 6 *Hz*, 2H). $^{13}$C-NMR ($CDCl_3$, 125 MHz): δ 135.6 (C), 129.6 (2 x CH), 129.0 (2 x CH), 126.3 (CH), 32.0 ($CH_2$), 31.9 ($CH_2$), 31.8 ($CH_2$), 31.6 ($CH_2$). HRMS ($EI^+$): *m/z* $[M]^+$ calcd. for $C_9H_{11}BrS$: 229.9765; found: 229.9766.

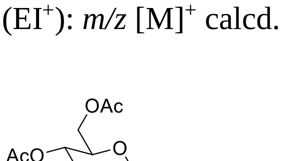

**(2*R*, 3*S*, 4*R*, 6*S*)-2-(acetoxymethyl)-6-(4-bromobutoxy) tetrahydro-2*H*-pyran-3,4-diyl diacetate (2q):** Following General Procedure **1**, to a solution of **1q** (150 mg, 0.43 mmol.) in water saturated DCM (containing 20 ppm of amylene or 5 ppm of 1,5-cyclooctadiene) (4.5 mL), was added TMSBr (0.17 mL, 1.3 mmol.) to afford 93 mg (0.22 mmol.) of **2q** as a sticky colorless oil. (51%).

$^1$H-NMR ($CDCl_3$, 400 MHz): δ 5.29 (ddd, *J* = 5.5, 9.5 *&* 11.7 *Hz*, 1H), 4.96 (m, 2H), 4.28 (dd, *J* = 4.8 *&* 12.2 *Hz*, 1H), 4.04 (dd, *J* = 2.3 *&* 12.2 *Hz*, 1H), 3.92 (ddd, *J* = 2.3, 4.7 *&* 10.1 *Hz*, 1H), 3.67 (m, 1H), 3.44 (t, *J* = 6.6 *Hz*, 2H), 2.22 (ddd, *J* = 1.1, 5.4 *&* 13.0 *Hz*, 1H), 2.08 (s, 3H), 2.03 (s, 3H), 2.00 (s, 3H), 1.95 (m, 2H), 1.86-1.70 (m, 3H). $^{13}$C-NMR ($CDCl_3$, 100 MHz):δ 170.7 (C), 170.2 (C), 169.9 (C), 96.9 (CH), 69.4 (CH), 69.1 (CH), 67.9 (CH), 66.7 ($CH_2$), 62.4 ($CH_2$), 35.0 ($CH_2$), 33.4 ($CH_2$), 29.5 ($CH_2$), 28.0 ($CH_2$),

20.9 ($CH_3$), 20.7 ($CH_3$), 20.6 ($CH_3$). HRMS (ESI$^+$): *m/z* $[M+Na]^+$ calcd. for $C_{16}H_{25}O_8Br$: 447.0630; found: 447.0628. $[\alpha]_D^{25}$: +69.0 (*c* = 1.00, $CHCl_3$).

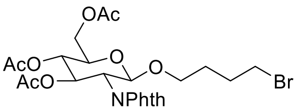


**(2*R*, 3*S*, 4*R*, 5*R*, 6*R*)-2-(acetoxymethyl)-6-(4-bromobutoxy)-5-phthalimidyl-tetrahydro-2*H*-pyran-3,4-diyl diacetate (2r):** Following General Procedure **1**, to a solution of **1r** (170 mg, 0.35 mmol.) in water saturated DCM (containing 20 ppm of amylene or 5 ppm of 1,5-cyclooctadiene) (3.5 mL), was added TMSBr (0.14 mL, 1.05 mmol.) to afford 160 mg (0.28 mmol.) of **2r** as a sticky pale yellow oil (80%).

$^1$H-NMR ($CDCl_3$, 400 MHz): δ 7.86 (dd, *J* = 3.0 & 5.5 *Hz*, 2H), 7.74 (dd, *J* = 3.0 & 5.5 *Hz*, 2H), 5.78 (dd, *J* = 9.1 & 10.8 *Hz*, 1H), 5.36 (dd, *J* = 8.5 *Hz*, 1H), 5.17 (dd, *J* = 9.2 & 10.1 *Hz*, 1H), 4.31 (m, 2H), 4.18 (dd, *J* = 2.4 & 12.2 *Hz*, 1H), 3.86 (m, 2H), 3.48 (ddd, *J* = 5.7, 6.9 & 10.1 *Hz*, 1H), 3.19 (t, *J* = 6.5 *Hz*, 2H), 2.12 (s, 3H), 2.03 (s, 3H), 1.86 (s, 3H), 1.76-1.65 (m, 2H), 1.64-1.54 (m, 3H). $^{13}$C-NMR ($CDCl_3$, 100 MHz): δ 170.7 (C), 170.1 (C), 169.4 (C), 134.3 (2 x CH), 131.3 (2 x C), 123.6 (2 x CH), 98.2 (CH), 71.9 (CH), 70.8 (CH), 69.1 (CH), 68.9 ($CH_2$), 62.0 ($CH_2$), 54.6 (CH), 33.0 ($CH_2$), 29.1 ($CH_2$), 27.9 ($CH_2$), 20.7 ($CH_3$), 20.6 ($CH_3$), 20.4 ($CH_3$). HRMS (ESI$^+$): *m/z* $[M+Na]^+$ calcd. for $C_{24}H_{28}O_{10}NBr$: 592.0794; found: 592.0790. $[\alpha]_D^{25}$: +17.1 (*c* = 1.03, $CHCl_3$).

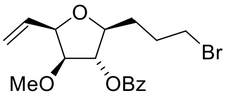


**(2*S*, 3*S*, 4*S*, 5*R*)-2-(3-bromopropyl)-4-methoxy-5-vinyltetrahydrofuran-3-yl benzoate (2s):** Following General Procedure **1**, to a solution of **1s** (150 mg, 0.52 mmol.) in water saturated DCM (containing 20 ppm of amylene or 5 ppm of 1,5-cyclooctadiene) (5 mL), was added TMSBr (0.21 mL, 1.56 mmol.) to afford 130 mg (0.35 mmol.) of **2s** as a pale yellow oil (67%).

$^1$H-NMR ($CDCl_3$, 400 MHz): δ 8.04 (dd, *J* = 1.4 & 8.5 *Hz*, 2H), 7.60 (tt, *J* = 1.3 & 7.4 *Hz*, 1H), 7.47 (t, *J* = 7.6 *Hz*, 2H), 6.02 (ddd, *J* = 7.0, 10.4 & 17.4 *Hz*, 1H), 5.42 (dt, *J* = 1.43 & 17.3 *Hz*, 1H), 5.32 (ddd, *J* = 1.0, 1.5 & 10.4 *Hz*, 1H), 5.19 (dd, *J* = 0.7 & 2.8 *Hz*, 1H), 4.46 (dd, *J* = 3.7 & 6.9 *Hz*, 1H), 4.01 (ddd, *J* = 2.9, 4.7 & 8.4 *Hz*, 1H), 3.77 (dd, *J* = 0.7 & 3.7 *Hz*, 1H), 3.48 (s, 3H), 3.51-3.41 (m, 2H), 2.19-2.06 (m, 1H), 2.06-1.82 (m, 3H). $^{13}$C-NMR ($CDCl_3$, 100 MHz): δ 165.7 (C), 133.4 (CH), 132.9 (CH), 129.7 (2 x CH), 129.5 (C), 128.5 (2 x CH), 118.7 ($CH_2$), 86.7 (CH), 82.7 (CH), 82.6 (CH), 80.7 (CH), 58.1 ($CH_3$), 33.5 ($CH_2$), 32.4 ($CH_2$), 29.3 ($CH_2$). HRMS (ESI$^+$): *m/z* $[M+Na]^+$ calcd. for $C_{17}H_{21}O_4Br$: 391.0521; found: 391.0520. $[\alpha]_D^{25}$: -29.2 (*c* = 1.05, $CHCl_3$).

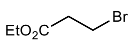


**Ethyl 3-bromopropanoate (2t):** Following General Procedure 1, to a solution of ethyl acrilate (0.1 mL, 0.94 mmol.) in water saturated DCM (containing 20 ppm of amylene or 5 ppm of 1,5-cyclooctadiene) (10 mL), was added TMSBr (0.4 mL, 2.8 mmol.) to afford 136 mg (0.75 mmol.) of **2t** as a colorless liquid (80%).

$^1$H-NMR ($CDCl_3$, 500 MHz): δ 4.22 (q, *J* = 7 *Hz*, 2H), 3.60 (t, *J* = 7 *Hz*, 2H), 2.93 (t, *J* = 6.5 *Hz*, 2H), 1.30 (t, *J* = 7.5 *Hz*, 3H). $^{13}$C-NMR ($CDCl_3$, 125 MHz): δ 170 (C), 61.0 ($CH_2$), 37.8 ($CH_2$), 25.9 ($CH_2$), 14.2 ($CH_3$). HRMS (EI$^+$): *m/z* $[M]^+$ calcd. for $C_5H_9BrO_2$: 179.9786; found: 179.9784.

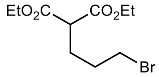


**Diethyl 2-(3-bromopropyl)malonate (2u):** Following General Procedure **1**, to a solution of diethyl allylmalonate (0.2 mL, 1.0 mmol.) in water saturated DCM (containing 20 ppm of amylene or 5 ppm of 1,5-cyclooctadiene) (10 mL), was added TMSBr (0.4 mL, 3 mmol.) to afford 190 mg (0.68 mmol.) of **2u** as a yellow oil (68%).

$^1$H-NMR ($CDCl_3$, 400 MHz): δ 4.12 (q, *J* = 6 *Hz*, 4H), 3.33 (t, *J* = 6 *Hz*, 2H), 3.26 (t, *J* = 7 *Hz*, 1H), 1.95 (m, 2H), 1.82 (m, 2H), 1.18 (t, *J* = 7 *Hz*, 6H). $^{13}$C-NMR ($CDCl_3$, 100 MHz): δ 169.3 (C), 61.5 (2 x $CH_2$),

51.4 (CH), 32.8 ($CH_2$), 30.6 ($CH_2$), 27.6 ($CH_2$), 14.4 (2 x $CH_3$). HRMS ($EI^+$): *m/z* $[M]^+$ calcd. for $C_{10}H_{17}BrO_4$: 280.0310; found: 280.0308.

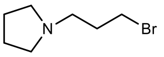

**1- (3-bromopropyl)pyrrolidine (2v):** Following General Procedure **1**, to a solution of **1v** (300 mg, 2.7 mmol.) in water saturated DCM (containing 20 ppm of amylene or 5 ppm of 1,5-cyclooctadiene) (25 mL), was added TMSBr (1.1 mL, 8.1 mmol.) to afford 270 mg (1.4 mmol.) of **2v** as a yellow liquid (52%) after one week stirring at room temperature. The product was purified using silica gel column chromatography with DCM:MeOH (8:2) as eluent.

$^1$H-NMR ($CDCl_3$, 400 MHz): δ 4.49 (t, *J* = 8 *Hz,* 2H), 3.83 (t, *J* = 6 *Hz,* 2H), 3.36 (m, 2H), 2.65 (quint, *J* = 8 *Hz*, 1H), 2.01 (m, 3H). $^{13}$C-NMR ($CDCl_3$, 100 MHz): δ 63.6 ($CH_2$), 63.4 ($CH_2$), 54.3 ($CH_2$), 52.6 ($CH_2$), 23.7 ($CH_2$), 21.6 ($CH_2$), 15.4 ($CH_2$). HRMS ($EI^+$): *m/z* $[M]^+$ calcd. for $C_7H_{14}BrN$: 191.0310; found: 191.0313.

## Characterization data for Markovnikov hydrobromination products

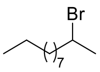

**2-bromoundecane (3a):** Following General Procedure **2**, to a solution of 1-undecene (0.28 mL, 1.3 mmol.) in dry DCM (13 mL), $FeBr_2$ (84 mg, 0.39 mmol.) followed by TMSBr (0.51 mL, 3.9 mmol.) were added to afford 198 mg (0.84 mmol.) of **3a** as a yellow liquid (65%). Spectroscopic data match with those reported in literature.[53]

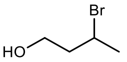

**3-bromobutan-1-ol (3b):** Following General Procedure **2**, to a solution of 4-buten-1-ol (0.12 mL, 1.39 mmol.) in dry DCM (14 mL), $FeBr_2$ (90 mg, 0.42 mmol.) followed by TMSBr (0.55 mL, 4.2 mmol.) were added to afford 132 mg (0.86 mmol.) of **3b** as a pale yellow liquid (62%).

$^1$H-NMR ($CDCl_3$, 500 MHz): δ 4.35 (m, 1H), 3.85 (m, 2H), 2.05 (m, 2H), 1.81 (brs, 1H), 1.78 (d, *J* = 7 *Hz*, 2H). $^{13}$C-NMR ($CDCl_3$, 125 MHz): δ 60.9 ($CH_2$), 48.0 (CH), 43.3 ($CH_2$), 26.7 ($CH_3$). HRMS ($EI^+$): *m/z* $[M]^+$ calcd. for $C_4H_9BrO$: 151.9837; found: 151.9845.

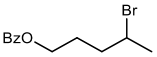

**4-bromopentyl benzoate (3c):** Following General Procedure **2**, to a solution of **1c** (100 mg, 0.52 mmol.) in dry DCM (4 mL), $FeBr_2$ (34 mg, 0.16 mmol.) followed by TMSBr (0.20 mL, 1.56 mmol.) were added to afford 140 mg (0.516 mmol.) of **3c** as a pale yellow liquid (99%).

$^1$H-NMR ($CDCl_3$, 400 MHz): δ 8.04 (dd, *J* = 1.2 & 8.2 *Hz,* 2H), 7.57 (m, 1H), 7.45 (t, *J* = 7.6 *Hz,* 2H), 4.36 (dt, *J* = 2.3 & 5.7 *Hz,* 2H), 4.19 (sext, *J* = 6.5 *Hz,* 1H), 2.09-1.99 (m, 1H), 1.96 (dd, *J* = 7.2 & 14 *Hz,* 2H), 1.93-1.87 (m, 1H), 1.75 (d, *J* = 6.7 *Hz,* 3H). $^{13}$C-NMR ($CDCl_3$, 100 MHz): δ 166.5 (C), 132.9 (CH), 130.3 (C), 129.6 (2 x CH), 128.4 (2 x CH), 64.2 ($CH_2$), 50.7 (CH), 37.7 ($CH_2$), 27.2 ($CH_2$), 26.5 ($CH_3$). HRMS ($EI^+$): *m/z* $[M]^+$ calcd. for $C_{12}H_{15}BrO_2$: 270.0255; found: 270.0263.

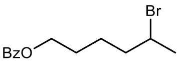

**5-bromohexyl benzoate (3d):** Following General Procedure **2**, to a solution of **1d** (270 mg, 1.3 mmol.) in dry DCM (13 mL), $FeBr_2$ (84 mg, 0.39 mmol.) followed by TMSBr (0.51 mL, 3.9 mmol.) were added to afford 365 mg (1.28 mmol.) of **3d** as a pale yellow liquid (98%).

$^1$H-NMR ($CDCl_3$, 400 MHz): δ 8.04 (dd, *J* = 1.2 & 8.2 *Hz,* 2H), 7.56 (m, 1H), 7.44 (t, *J* = 8 *Hz,* 2H), 4.34 (t, *J* = 6.5 *Hz,* 2H), 4.15 (sext, *J* = 6.5 *Hz,* 1H), 1.95-1.75 (m, 4H), 1.72 (d, *J* = 6.7 *Hz,* 3H), 1.70-1.65 (m, 1H), 1.65-1.54 (m, 1H). $^{13}$C-NMR ($CDCl_3$, 100 MHz): δ 166.6 (C), 132.9 (CH), 130.4 (C), 129.5 (2 x

CH), 128.3 (2 x CH), 64.6 ($CH_2$), 51.1 (CH), 40.7 ($CH_2$), 28.1 ($CH_2$), 26.4 ($CH_3$), 24.4 ($CH_2$). HRMS ($EI^+$): *m/z* $[M+ Na]^+$ calcd. for $C_{13}H_{17}BrO_2$: 307.0310; found: 307.0303.

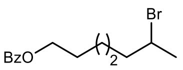


**6-bromoheptyl benzoate (3e):** Following General Procedure **2**, to a solution of **1e** (100 mg, 0.46 mmol.) in dry DCM (5 mL), $FeBr_2$ (30 mg, 0.14 mmol.) followed by TMSBr (0.18 mL, 1.38 mmol.) were added to afford 108 mg (0.36 mmol.) of **3e** as a pale yellow liquid (78%).

$^1$H-NMR ($CDCl_3$, 400 MHz): δ 8.04 (d, *J* = 8.2 *Hz*, 2H), 7.56 (t, *J* = 7.4 *Hz*, 1H), 7.44 (t, *J* = 8 *Hz*, 2H), 4.33 (t, *J* = 6.5 *Hz*, 2H), 4.14 (sext, *J* = 6.5 *Hz*, 1H), 1.88-1.74 (m, 5H), 1.71 (d, *J* = 6.7 *Hz*, 3H), 1.66-1.53 (m, 1H), 1.53-1.40 (m, 2H). $^{13}$C-NMR ($CDCl_3$, 100 MHz): δ 166.6 (C), 132.8 (CH), 130.4 (C), 129.5 (2 x CH), 128.3 (2 x CH), 64.9 ($CH_2$), 51.6 (CH), 41.0 ($CH_2$), 28.6 ($CH_2$), 27.5 ($CH_2$), 26.4 ($CH_3$), 25.5 ($CH_2$). HRMS ($EI^+$): *m/z* $[M+ Na]^+$ calcd. for $C_{14}H_{19}BrO_2$: 321.0466; found: 321.0465.

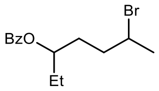


**6-bromoheptan-3-yl benzoate (3f):** Following General Procedure **2**, to a solution of **1f** (280 mg, 1.3 mmol.) in dry DCM (13 mL), $FeBr_2$ (86 mg, 0.39 mmol.) followed by TMSBr (0.51 mL, 3.9 mmol.) were added to afford 194 mg (0.65 mmol.) of **3f** as a colorless liquid (50%). The product was obtained as an inseparable mixture of diastereomers.

$^1$H-NMR ($CDCl_3$, 400 MHz): δ *syn:anti* mixture 8.04 (m, 4H), 7.56 (m, 2H), 7.45 (m, 4H), 5.25-5.16 (m, 1H), 4.23-4.08 (m, 1H), 4.06-3.93 (m, 1H), 2.07-1.73 (m, 12H), 1.70 (d, *J* = 6.6 *Hz*, 3H), 1.37 (d, *J* = 6.3 *Hz*, 3H), 1.03 (t, *J* = 7.3 Hz, 3H), 0.97 (t, *J* = 7.4 Hz, 3H). $^{13}$C-NMR ($CDCl_3$, 100 MHz): δ 166.1 (2 x C), 132.8 (2 x CH), 130.4 (C), 129.5 (2 x CH), 128.4 (2 x CH), 128.3 (2 x CH), 75.0 (CH), 70.7 (CH), 59.3 (CH), 51.2 (CH), 36.6 ($CH_2$), 34.4 ($CH_2$), 34.0 ($CH_2$), 32.3 ($CH_2$), 31.9 ($CH_2$), 27.2 ($CH_2$), 26.5 ($CH_2$), 20.2 ($CH_3$), 12.1 ($CH_3$), 9.6 ($CH_3$). HRMS ($EI^+$): *m/z* $[M+Na]^+$ calcd. for $C_{14}H_{19}BrO_2$: 321.0466; found: 321.0463.

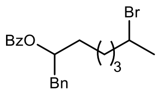


**7-bromo-1-phenyloctan-2-yl benzoate (3g):** Following General Procedure **2**, to a solution of **1g** (200 mg, 0.65 mmol.) in dry DCM (6.5 mL), $FeBr_2$ (41 mg, 0.19 mmol.) followed by TMSBr (0.26 mL, 1.95 mmol.) were added to afford 127 mg (0.326 mmol.) of **3g** as a pale yellow oil (50%). The product was obtained as an inseparable mixture of diastereomers.

$^1$H-NMR ($CDCl_3$, 400 MHz): δ *syn:anti* mixture 8.02 (d, *J* = 8 *Hz*, 2H), 7.56 (m, 1H), 7.44 (t, *J* = 7 *Hz*, 2H), 7.32-7.17 (m, 5H), 5.33 (m, 1H), 4.08 (m, 1H), 3.05 (dd, *J* = 6.2 & 13.6 *Hz*, 1H), 2.93 (dd, *J* = 6.4 & 14 *Hz*, 1H), 1.86-1.68 (m, 4H), 1.67 (d, *J* = 6.7 *Hz*, 3H), 1.55-1.30 (m, 4H). $^{13}$C-NMR ($CDCl_3$, 100 MHz): δ 166.1 (C), 137.4 (C), 132.8 (CH), 130.6 (C), 129.5 (4 x CH), 128.4 (2 x CH), 128.3 (2 x CH), 126.5 (CH), 75.3 (2 x CH), 51.6 (CH), 51.5 (CH), 40.9 (2 x $CH_2$), 40.6 (2 x $CH_2$), 33.3 (2 x $CH_2$), 27.6 (2 x $CH_2$), 26.4 (2 x $CH_3$), 24.8 (2 x $CH_2$). HRMS ($EI^+$): *m/z* $[M+Na]^+$ calcd. for $C_{21}H_{25}BrO_2$: 411.0936; found: 411.0920.

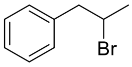


**(2-bromopropyl)benzene (3j):** Following General Procedure **2**, to a solution of allylbenzene (0.11 mL, 0.85 mmol.) in dry DCM (8.5 mL), $FeBr_2$ (54 mg, 0.25 mmol.) followed by TMSBr (0.34 mL, 2.55 mmol.) were added to afford 160 mg (0.804 mmol.) of **3j** as a pale yellow oil (95%).

$^1$H-NMR ($CDCl_3$, 400 MHz): δ 7.35-7.29 (t, J = 7.3 *Hz*, 2H), 7.29-7.23 (t, *J* = 7.26 *Hz*, 1H), 7.23-7.18 (d, *J* = 7.1 *Hz*, 2H), 4.31 (sex, *J* = 6.9 *Hz*, 1H), 3.23 (dd, *J* = 7.0 *&* 14.0 *Hz*, 1H), 3.07 (dd, *J* = 7.3 *&* 13.9 *Hz*, 1H), 1.70 (d, *J* = 6.7 *Hz*, 3H). $^{13}$C-NMR ($CDCl_3$, 100 MHz): δ 138.5 (C), 129.2 (2 x CH), 128.4 (2 x CH), 126.8 (CH), 50.5 (CH), 47.5 ($CH_2$), 25.7 ($CH_3$). HRMS ($ESI^+$): *m/z* $[M]^+$ calcd. for $C_9H_{11}Br$: 198.0044; found: 198.0048.

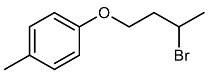

**1-(3-bromobutoxy)-4-methylbenzene (3m):** Following General Procedure **2**, to a solution of **1m** (80 mg, 0.49 mmol.) in dry DCM (5 mL), $FeBr_2$ (32 mg, 0.15 mmol.) followed by TMSBr (0.19 mL, 1.47 mmol.) were added to afford 48 mg (0.20 mmol.) of **3m** as a pale yellow oil (41%). The product was purified by radial chromatography 1mm disc using *n*-hexane/EtOAc system.

$^1$H-NMR ($CDCl_3$, 500 MHz): δ 7.09 (d, *J* = 8.2 *Hz*, 2H), 6.81 (d, *J* = 8.6 *Hz*, 2H), 4.41 (m, 1H), 4.10 (m, 2H), 2.29 (s, 3H), 2.28-2.17 (m, 1H), 1.80 (d, *J* = 6.7 *Hz*, 3H). $^{13}$C-NMR ($CDCl_3$, 125 MHz): δ 156.6 (C), 130.1 (C), 129.9 (2 x CH), 114.4 (2 x CH), 65.9 ($CH_2$), 47.7 (CH), 40.6 ($CH_2$), 26.6 ($CH_3$), 20.4 ($CH_3$). HRMS ($ESI^+$): *m/z* $[M]^+$ calcd. for $C_{11}H_{15}BrO$: 242.0306; found: 242.0303.

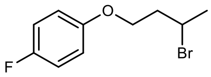

**1-(3-bromobutoxy)-4-fluorobenzene (3n):** Following General Procedure **2**, to a solution of **1n** (83 mg, 0.5 mmol.) in dry DCM (5 mL), $FeBr_2$ (33 mg, 0.15 mmol.) followed by TMSBr (0.20 mL, 1.50 mmol.) were added to afford 62 mg (0.25 mmol.) of **3n** as a pale yellow oil (50%). The product was purified by radial chromatography 1mm disc using *n*-hexane/EtOAc system.

$^1$H-NMR ($CDCl_3$, 500 MHz): δ 6.97 (brt, *J* = 8.7 *Hz*, 2H), 6.84 (dd, *J* = 9.2 *&* 4.3 *Hz*, 2H), 4.40 (m, 1H), 4.09 (m, 2H), 2.30-2.23 (m, 1H), 2.23-2.15 (m, 1H), 1.80 (d, *J* = 6.9 *Hz*, 3H). $^{13}$C-NMR ($CDCl_3$, 125 MHz): δ 157.2 (C, d, *J* = 237 *Hz*), 154.8 (C, d, *J* = 1.6 *Hz*), 115.9 (CH), 115.7 (CH), 115.8 (CH), 115.5 (CH), 66.5 ($CH_2$), 47.5 (CH), 40.5 ($CH_2$), 26.6 ($CH_3$). HRMS ($ESI^+$): *m/z* $[M]^+$ calcd. for $C_{10}H_{12}BrFO$: 246.0056; found: 246.0045.

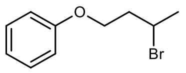

**3-(bromobutoxy)benzene (3o):** Following General Procedure **2**, to a solution of **1o** (75 mg, 0.51 mmol.) in dry DCM (5 mL), $FeBr_2$ (32 mg, 0.15 mmol.) followed by TMSBr (0.20 mL, 1.53 mmol.) were added to afford 63 mg (0.27 mmol.) of **3o** as a pale yellow oil (53%). The product was purified by radial chromatography 1mm disc using *n*-Hex/EtOAc system.

$^1$H-NMR ($CDCl_3$, 500 MHz): δ 7.29 (dd, *J* = 8.7 *&* 7.4 *Hz*, 2H), 6.96 (brt, *J* = 7.4 *Hz*, 1H), 6.92 (dd, *J* = 8.7 *&* 0.9 *Hz*, 2H), 4.42 (m, 1H), 4.41 (m, 2H), 2.32-2.25 (m, 1H), 2.25-2.18 (m, 1H), 1.81 (d, *J* = 6.8 *Hz*, 3H). $^{13}$C-NMR ($CDCl_3$, 125 MHz): δ 158.7 (C), 129.5 (2 x CH), 120.9 (CH), 114.6 (2 x CH), 65.7 ($CH_2$), 47.7 (CH), 40.5 ($CH_2$), 26.6 ($CH_3$). HRMS ($ESI^+$): *m/z* $[M]^+$ calcd. for $C_{10}H_{13}BrO$: 228.0150; found: 228.0143.

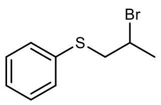

**(2-bromopropylphenyl)sulfane (3p):** Following General Procedure **2**, to a solution of **1p** (1.4 g, 9.32 mmol.) in dry DCM (90 mL), $FeBr_2$ (604 mg, 2.80 mmol.) followed by TMSBr (3.69 mL, 28.0 mmol.) were added. The liquid obtained was distilled (b. p. 110-112 ºC, 1 mm Hg), to afford 1.5 g (6.49 mmol.) of **3p** as a colorless liquid (70%).

A $^1$HNMR analysis revealed that the colorless liquid contained an inseparable mixture of structural isomers **3p** and (1-bromopropan-2-yl)phenylsulfane (75:25).

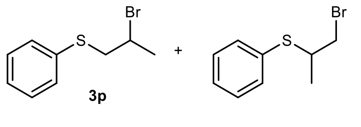


Similar mixtures has been reported in the reaction of 2-methylsulfoniloxy-1-phenylthiopropane with lithium bromide[54] and 2-phenylthio-1-propanol with phosphorus tribromide.[55]

Spectroscopic data for **3p**: $^1$H-NMR ($CDCl_3$, 400 MHz): δ 7.40-7.17 (m, 7H), 4.08 (m, 1H), 3.53 (dd, *J* = 5 & 10 *Hz*, 1H), 3.13 (dd, *J* = 4 & 10 *Hz*, 1H), 1.75 (d, *J* = 6.6 *Hz*, 3H). $^{13}$C-NMR ($CDCl_3$, 100 MHz): δ 132.9 (C), 130.6 (2 x CH), 129.5 (2 x CH), 127.2 (CH), 47.5 ($CH_2$), 44.4 (CH), 24.9 ($CH_3$).
Spectroscopic data for (1-bromopropan-2-yl)phenylsulfane: $^1$H-NMR ($CDCl_3$, 400 MHz): δ 7.40-7.17 (m, 7H), 3.53 (d, *J* = 4 *Hz*, 1H), 3.40 (m, 1H), 3.26 (t, *J* = 10 *Hz*, 1H), 1.75 (d, *J* = 6.7 *Hz*, 3H). $^{13}$C-NMR ($CDCl_3$, 100 MHz): δ 135.5(C), 130.6 (2 x CH), 129.5 (2 x CH), 128.1 (CH), 44.5 (CH), 38.2 ($CH_2$), 19.3 ($CH_3$). HRMS (ESI$^+$): *m/z* $[M]^+$ calcd. for $C_9H_{11}BrS$: 229.9765; found: 229.9760.

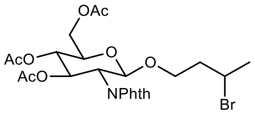


**(2*R*, 3*S*, 4*R*, 5*R*, 6*R*)-2-(acetoxymethyl)-6-(3-bromobutoxy)-5-(1,3-dioxoisoindolin-2-yl)tetrahydro-2*H*-pyran-3,4-siyl diacetate (3r):** Following General Procedure **2**, to a solution of **1r** (280 mg, 0.57 mmol.) in dry DCM (6 mL), $FeBr_2$ (36 mg, 0.17 mmol.) followed by TMSBr (0.22 mL, 1.71 mmol.) were added. The reaction was stirred for 5 days at 50 ºC to afford 210 mg (0.368 mmol.) of **3r** as a sticky oil (65%). The product was obtained as an inseparable mixture of diastereomers.
$^1$H-NMR ($CDCl_3$, 400 MHz): δ 7.77 (m, 2H), 7.67 (m, 2H), 5.71 (ddd, *J* = 9, 11 & 26 *Hz*, 1H), 5.28 (dd, *J* = 8 & 11 *Hz*, 1H), 5.07 (dt, *J* = 3 & 10 *Hz*, 1H), 4.27-4.19 (m, 2H), 4.09 (m, 1H), 3.99-3.77 (m, 3H), 3.55 (m, 1H), 2.02 (d, *J* = 3 *Hz*, 3H), 1.95 (s, 3H), 1.84 (m, 2H), 1.77 (d, *J* = 4 *Hz*, 3H), 1.43 (dd, *J* = 7 & 10 *Hz*, 3H). $^{13}$C-NMR ($CDCl_3$, 100 MHz): δ mixture of diastereomers 171.0 (C), 170.9 (C), 170.4 (C), 170.3 (C), 169.8 (C), 169.7 (C), 168.0 (C), 134.7 (CH), 134.6 (CH), 131.7 (C), 123.9 (CH), 98.8 (CH), 98.3 (CH), 72.2 (CH), 72.1 (CH), 71.1 (CH), 70.9 (CH), 69.3 ($CH_2$), 68.4 (CH), 68.2 (CH), 62.3 ($CH_2$), 54.9 (CH), 54.8 (CH), 47.6 (CH), 40.8 (CH), 40.5 (CH), 26.6 ($CH_3$), 26.5 ($CH_3$), 21.0 ($CH_3$), 20.9 ($CH_3$), 20.7 ($CH_3$). HRMS (ESI$^+$): *m/z* $[M+Na]^+$ calcd. for $C_{24}H_{28}BrNO_{10}$: 592.0897; found: 592.0899.

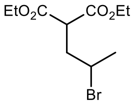


**Diethyl 2-(2-bromopropyl)malonate (3u):** Following General Procedure **2**, to a solution of diethyl allylmalonate (0.2 mL, 1 mmol.) in dry DCM (10 mL), $FeBr_2$ (65 mg, 0.3 mmol.) followed by TMSBr (0.40 mL, 3 mmol.) were added to afford 205 mg (0.73 mmol.) of **3u** as a pale yellow oil (73%).
$^1$H-NMR ($CDCl_3$, 400 MHz): δ 4.17-4.01 (m, 5H), 3.61 (m, 1H), 2.30 (m, 1H), 2.17 (m, 1H), 1.65 (m, 3H), 1.87 (m, 6H). $^{13}$C-NMR ($CDCl_3$, 100 MHz): δ 169.1 (C), 168.9 (C), 61.9 ($CH_2$), 61.8 ($CH_2$), 51.1 (CH), 48.4 (CH), 39.9 ($CH_2$), 26.8 ($CH_3$), 14.3 ($CH_3$), 14.2 ($CH_3$). HRMS (ESI$^+$): *m/z* $[M]^+$ calcd. for $C_{10}H_{17}BrO_4$: 280.0310; found: 280.0314.

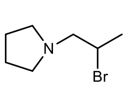


**1-(2-bromopropyl)pyrrolidine (3v):** Following General Procedure **2**, to a solution of **1v** (400 mg, 3.6 mmol.) in dry DCM (36 mL), $FeBr_2$ (233 mg, 1.08 mmol.) followed by TMSBr (1.4 mL, 10.8 mmol.) were added to afford 366 mg (1.90 mmol.) of **3v** as a pale yellow oil (53%) after 3 weeks reaction time. The product was purified by column chromatography using DCM/MeOH system.
$^1$H-NMR ($CDCl_3$, 400 MHz): δ 4.19 (m, 1H), 3.80 (dd, *J* = 5 & 10 *Hz*, 1H), 3.68 (m, 1H), 3.50 (t, *J* = 12 *Hz*, 1H), 2.59-2.38 (m, 3H), 1.77 (d, *J* = 7 *Hz*, 3H), 1.70 (m, 4H). $^{13}$C-NMR ($CDCl_3$, 100 MHz): δ 68.3 ($CH_2$), 55.1 ($CH_2$), 51.4 ($CH_2$), 38.0 (CH), 24.5 ($CH_3$), 23.8 ($CH_2$), 23.6 ($CH_2$). HRMS (ESI$^+$): *m/z* $[M]^+$ calcd. for $C_7H_{14}BrN$: 191.0310; found: 191.0306.

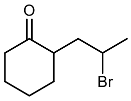


**2-(2-bromopropyl)cyclohexan-1-one (3w):** Following General Procedure **2**, to a solution of 2-allylcyclohexanone (0.2 mL, 1.34 mmol.) in dry DCM (13 mL), $FeBr_2$ (86 mg, 0.40 mmol.)

followed by TMSBr (0.53 mL, 4.04 mmol.) were added to afford 220 mg (1.00 mmol.) of **3w** as a colorless oil (75%). The product was obtained as an inseparable mixture of diastereomers.

$^{1}$H-NMR ($CDCl_3$, 400 MHz): δ *syn:anti* mixture 4.24 (m, 1H), 4.10 (m, 1H), 2.67-2.51 (m, 2H), 2.38-2.25 (m, 4H), 2.17 (m, 2H), 2.03 (m, 3H), 1.82 (m, 2H), 1.72-1.52 (m, 10H), 1.45-1.17 (m, 5H). $^{13}$C-NMR ($CDCl_3$, 100 MHz): δ 212.8 (C), 212.3 (C), 52.1 (CH), 49.8 (CH), 49.4 ($CH_2$), 49.3 ($CH_2$), 42.7 ($CH_2$), 42.4 ($CH_2$), 42.0 ($CH_2$), 40.4 ($CH_2$), 35.7 ($CH_2$), 33.3 ($CH_2$), 28.6 ($CH_2$), 28.1 ($CH_2$), 27.5 ($CH_3$), 27.1 ($CH_3$), 25.7 ($CH_2$), 25.4 ($CH_2$). HRMS (ESI$^+$): *m/z* $[M]^+$ calcd. for $C_9H_{15}BrO$: 218.0306; found: 218.0309.

## Mechanistic experiments

### Radical clock experiments

*Anti*-Markovnikov reaction. In the presence of the radical clock ((1-(2-phenylcyclopropyl)vinyl)benzene, (*Nat. Catal.* **2021**, *4*, 28-35) the reaction was totally inhibited and the starting material **1d** was recovered. In the case of the Markovnikov reaction, in the presence of the same radical clock the yield of the reaction decreased from 98% to 70%.

***anti*-Markovnikov hydrobromination of alkenes**

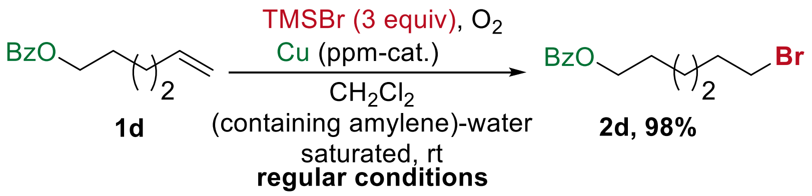


**Radical Clock experiment**

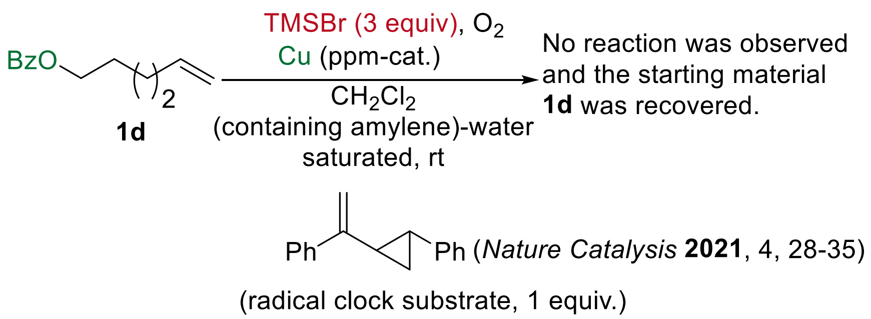


**Markovnikov hydrobromination of alkenes**

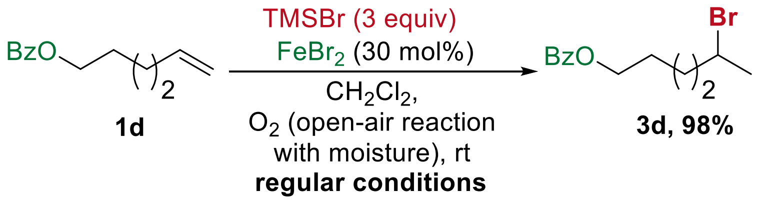


**Radical Clock experiment**

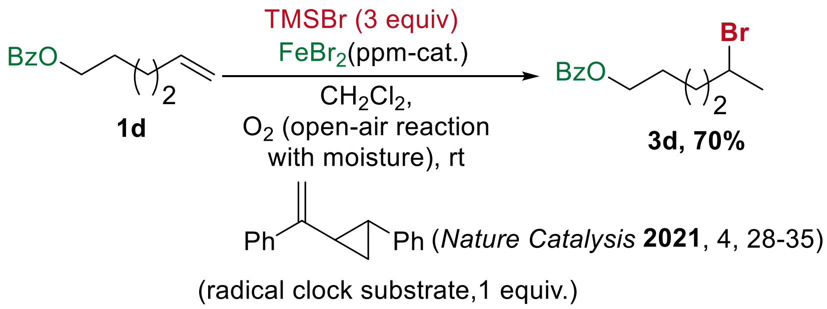

## KIE experiments

### *Anti*-Markovnikov hydrobromination reaction.

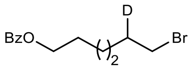

**6-bromohexyl-5-*d* benzoate (4d):** Following General Procedure **1**, to a solution of **1d** (100 mg, 0.5 mmol.) in $D_2O$-saturated DCM (containing 20 ppm of amylene or 5 ppm of 1,5-cyclooctadiene) (5 mL), was added TMSBr (0.20 mL, 1.50 mmol.) to afford 130 mg (0.45 mmol.) of **4d** as a colorless oil (90%)

$^1$H-NMR ($CDCl_3$, 400 MHz): δ 8.04 (dd, $J$ = 1 & 8 *Hz*, 2H), 7.56 (m, 1H), 7.44 (m, 2H), 4.33 (t, $J$ = 6 *Hz*, 2H), 3.41 (d, J = 6.7 *Hz*, 2H), 1.89 (m, 1H), 1.79 (m, 2H), 1.51 (m, 4H). $^{13}$C-NMR ($CDCl_3$, 100 MHz): δ 166.6(C), 132.8 (CH), 130.4 (C), 129.5 (2 x CH), 128.3 (2 x CH), 64.8 ($CH_2$), 33.5 ($CH_2$), 32.3 (t, $J$ = 20 *Hz*, CH), 28.6 ($CH_2$), 27.7 ($CH_2$), 25.3 ($CH_2$). HRMS (ESI$^+$): *m/z* $[M+Na]^+$ calcd. for $C_{13}H_{16}BrO_2D$: 308.372; found: 308.383.

**Table S6:** Kinetic studies.

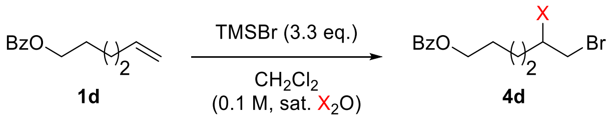


| Entry | X | Time (min) |
|---|---|---|
| 1 | D | 40 |
| 2 | H | 30 |

### Markovnikov hydrobromination reaction.

In order to study KIE in this reaction, we carried out several experiments using deuterated silane, solvent ($CD_2Cl_2$) and deuterium oxide. No deuterium incorporation was observed either using deuterated silane or $CD_2Cl_2$ or both at the same time. The work-up of the reaction was performed using $D_2O$ and no incorporation of deuterium was observed either. It was neither observed after the combination, in the same reaction, of all deuterated species commented above. We will comment the proton source later (referee 2) and throughout this letter.

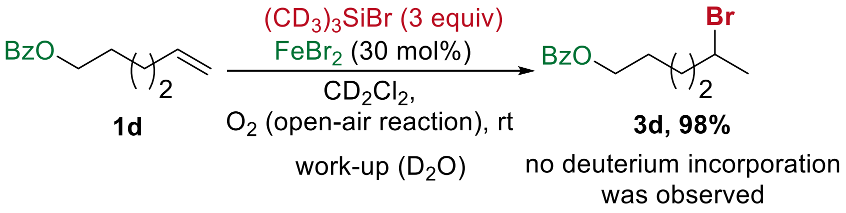


## Radical scavengers

### *Anti*-Markovnikov hydrobromination reaction.

First, we ran the reaction with 1 equiv. of TEMPO as a radical scavenger. However, we obtained the dibrominated derivative as the only final compound in 55% yield. When we increased the amount of TEMPO (2 equiv.), the yield of the dibrominated compound rised up to 70%.

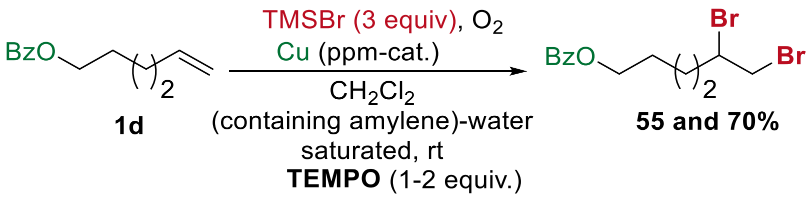


A molecule of TEMPO can act as a radical scavenger or as catalyst in organic oxidation reactions. In our case, TEMPO acts as an oxidant generating $Br_2$ in situ leading to the dibrominated compound in 55% yield when 1 equiv. was used or 70% yield with 2 equiv. This fact matches the precedents described in *J. Org. Chem*. **2015**, *80*, 3701-3707 and *Green Chem*. **2015**, *17*, 3285.
In both cases, the authors observed the dibromination of olefins with HBr and DMSO. In our case, TEMPO acts as oxidant such as DMSO. In fact, when we use DMSO instead of TEMPO the dibrominated derivative was also isolated.

In the reaction media, and as a parallel process, TMSBr reacts with the water present in the solvent to produce HBr smoothly. This HBr in presence of TEMPO or DMSO leads to the dibrominated product.

In addition, we used hydroquinone as another radical scavenger (benzene 1-4 diol). In this case, the reaction was inhibited and the starting material recovered. The same result was obtained using a different radical scavenger such as thiophenol.

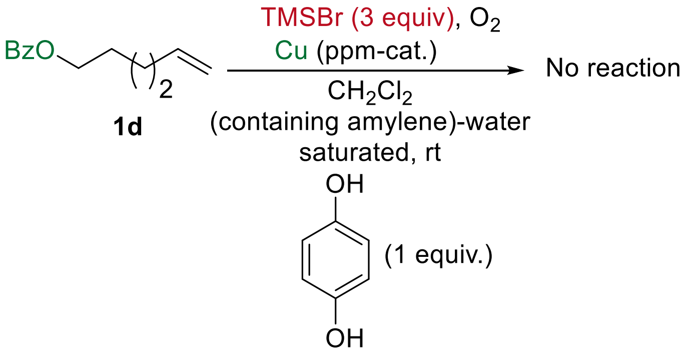


**Positive result with KI starch paper.**

We also performed some qualitative essays using KI starch paper (Starch and Potassium iodide paper) to detect the presence of peroxides. Oxidizing agents react with potassium iodide to produce elemental iodine which reacts with the starch to form a blue-violet color reaction. In our case, the final color turns blue-violet confirming the presence of peroxides.

**Markovnikov hydrobromination reaction.**

As in the *anti*-Markovnikov reaction, in the presence of TEMPO the corresponding dibrominated derivative was the only isolable product.

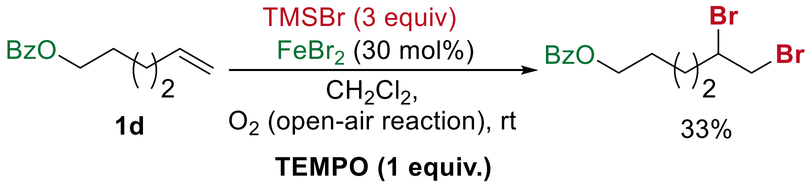


This result indirectly shows that HBr must be produced, in situ, for the dibrominated compound to be generated.

Next, following the procedure described in the *anti*-Markovnikov reaction, we also checked hydroquinone as radical scavenger.

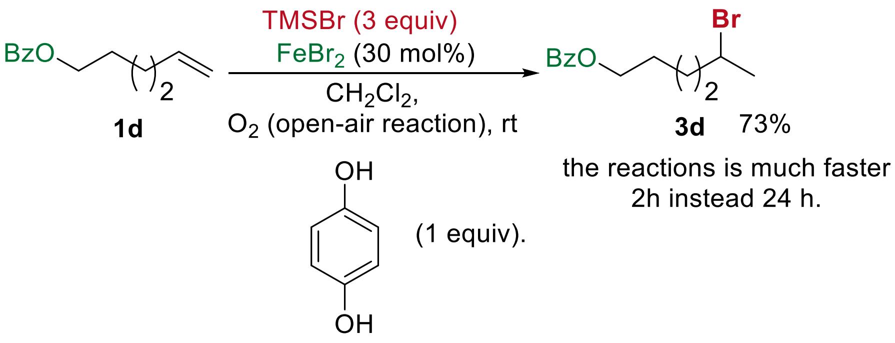


In this case, the Markovnikov product was obtained with a lower yield (73%) but the reaction proceeded much faster, 2h instead 24h without hydroquinone.

Furthermore, the Markovnikov product was also obtained in the presence of thiophenol, as a radical scavenger, but in this case we did not observe any significant change in the reaction rate.

**Negative result with KI starch paper.**

The potassium iodide starch paper is used to detect the presence of oxidizing agents such as nitrite, free chlorine, iodine and peroxide. We did not observe the characteristic positive blue-violet color of this test. Therefore, no peroxide as intermediate was detected.

In the cases of the possible radical scavengers (hydroquinone and thiophenol), we hypothesize two possible scenarios, namely acting as oxidizing agent or as Hydrogen Atom Donor (HAD).

As oxidizing agent, the hydroquinone with oxygen could generate hydrogen peroxide and quinone.

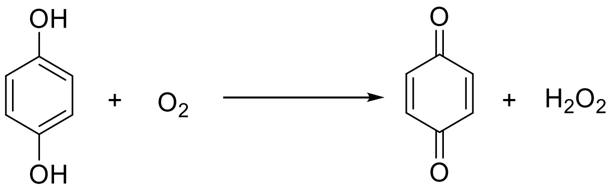


However, the presence of the peroxide should be detected with KI starch paper and this test was negative. This fact reinforces the hypothesis that hydroquinone and thiophenol act as Hydrogen Atom Donor (HAD) at the final stage, accelerating the reaction rate. Precedents of iron(II) and HAD can be found in Kwon O. et al. *Science* **2019**, *364*, 681–685 and references cited therein.

**<u>Study of the possible superoxo species</u>**

We have performed EPR spectroscopy (NMR services at the University of Santiago de Compostela, Spain) and ESI-MS (mass spectrometry service at IPNA-CSIC) and no traces of Cu(II)-superoxo and Fe(III)-superoxo intermediates were detected. In addition, we have treated both reactions with a $NaH^{13}CO_3$ solution to trap the superoxo intermediates (Carrillo et al. *Chem. Commun.* **2015**, *51*, 7027-7030). In the presence of this intermediate, we should have been able to detect $NaHC^{13}O_4^-$ as the final species, and detect it through $^{13}C$ NMR. However, we did not observe any signal in the corresponding $^{13}C$ NMR experiments either.

$$NaH^{13}CO_3^- + H_2O_2 \rightleftharpoons NaH^{13}CO_4^- + H_2O$$

As a result of all these experiments, we could not detect any traces of Cu(II)- and Fe(III)-superoxo species as intermediates or the presence of hydrogen peroxide.

**Lack of influence of the Copper(I) salts in the Markovnikov reaction**

It is very interesting to prove that the small amount of Cu(I) present in the commercial TMSBr has no influence in the Markovnikov hydrobromation catalyzed by iron(II) bromide.

First, we ran the reaction using homemade Cu-free TMSBr (checked by ICP). The Markovnikov hydrobromination worked well leading the **3d** compound in 99% yield.

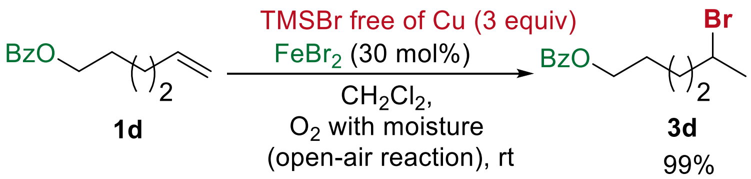


Next, we ran two experiments without $FeBr_2$, the first with TMSBr free of Cu(I) and the second with commercially TMSBr that contains Cu(I). In both cases, no reaction at all was observed with a reaction time of 7 hours. After that time, 30 mol% of $FeBr_2$ was added to each reaction, and now the Markovnikov product **3d** was observed in both cases, confirming the importance of iron(II) salt in this processes.

## Computational Details

All the calculations reported in this paper were performed with the Gaussian 09 suite of programs.[56] Electron correlation was partially taken into account using the hybrid functional usually denoted as B3LYP[57–59] in conjunction with the D3 dispersion correction suggested by Grimme et al.[60] using the double-ζ quality plus polarization def2-SVP[61] basis set for all atoms. All species were characterized by frequency calculations,[62] and have positive definite Hessian matrices. Solvents effects were taken into account using the Polarizable Continuum Model (PCM)[63–65] during the geometry optimizations. This level is denoted PCM($CH_2Cl_2$)-B3LYP-D3/def2-SVP.

Condensed Fukui indices calculation is based on frontier molecular orbitals and computation of electron density distribution for neutral molecule and ± ions with geometry optimized for neutral molecule. Radical susceptibility ($R$) is defined as:

$R(\mathrm{A}) = ½ (P_{\mathrm{A}}(N + 1) – P_{\mathrm{A}}( N –1))$

where $P$ stands for A atom occupancy (computed using the NBO 6.0 program).[66]

Cartesian coordinates (in Å) and total energies energies (in a. u., ZPVE included) of all the stationary points discussed in the text. All calculations have been performed at the PCM($CH_2Cl_2$)-B3LYP-D3/def2-SVP.

**AcO-($CH_2$)$_3$-CH=$CH_2$:** E = -423.942409

| | | | |
|---|---|---|---|
| C | 0.310432000 | 0.472756000 | -0.073483000 |
| H | 0.325634000 | 1.112148000 | 0.825072000 |
| H | 0.429411000 | 1.136733000 | -0.946114000 |
| C | -0.966821000 | -0.341996000 | -0.159487000 |
| H | -1.025038000 | -1.014254000 | 0.712943000 |
| H | -0.920421000 | -0.986564000 | -1.054062000 |
| C | -2.217744000 | 0.548472000 | -0.215786000 |
| H | -2.127773000 | 1.228715000 | -1.083946000 |
| H | -2.263191000 | 1.188577000 | 0.682222000 |
| O | 1.417592000 | -0.443505000 | -0.021020000 |
| C | 2.649709000 | 0.087258000 | 0.058104000 |
| O | 2.856548000 | 1.279434000 | 0.083074000 |
| C | 3.704439000 | -0.988331000 | 0.111824000 |
| H | 3.537090000 | -1.628184000 | 0.991823000 |
| H | 3.627774000 | -1.630881000 | -0.778537000 |
| H | 4.699576000 | -0.531243000 | 0.162981000 |
| C | -3.488003000 | -0.245343000 | -0.344942000 |
| H | -3.556792000 | -0.886880000 | -1.234206000 |
| C | -4.500571000 | -0.235658000 | 0.527524000 |
| H | -4.471501000 | 0.385651000 | 1.429793000 |
| H | -5.396539000 | -0.844198000 | 0.373078000 |

**AcO-($CH_2$)$_3$-CH=$CH_2$(FeBr2) complex1:** E = -6835.333184

| | | | |
|---|---|---|---|
| C | 3.394341000 | 0.138185000 | -0.074805000 |
| H | 3.491109000 | 1.043723000 | 0.546441000 |
| H | 3.257243000 | 0.471318000 | -1.117322000 |
| C | 2.242442000 | -0.735573000 | 0.388007000 |
| H | 2.421146000 | -1.045644000 | 1.431096000 |
| H | 2.220652000 | -1.655224000 | -0.220482000 |
| C | 0.900340000 | 0.005633000 | 0.276832000 |
| H | 0.720338000 | 0.283061000 | -0.773222000 |
| H | 0.927541000 | 0.943491000 | 0.852371000 |
| O | 4.599386000 | -0.637607000 | 0.020792000 |
| C | 5.751219000 | -0.017277000 | -0.290934000 |
| O | 5.805948000 | 1.142636000 | -0.630635000 |
| C | 6.926218000 | -0.951556000 | -0.160736000 |
| H | 6.968162000 | -1.358656000 | 0.861056000 |
| H | 6.800377000 | -1.802568000 | -0.847534000 |
| H | 7.854434000 | -0.415842000 | -0.391578000 |
| C | -0.223898000 | -0.862802000 | 0.761485000 |
| H | -0.374237000 | -1.790399000 | 0.199132000 |
| C | -0.837602000 | -0.735802000 | 1.987287000 |
| H | -0.554567000 | 0.071660000 | 2.676117000 |
| H | -1.418330000 | -1.564770000 | 2.414052000 |
| Fe | -2.249814000 | 0.067288000 | 0.720333000 |
| Br | -1.690062000 | 2.092283000 | -0.268196000 |
| Br | -3.052216000 | -1.521275000 | -0.783314000 |

**AcO-($CH_2$)$_3$-CH=$CH_2$(FeBr2) complex2:** E = -6835.357921

| | | | |
|---|---|---|---|
| O | -3.284681000 | -0.641709000 | 0.521416000 |

| | | | |
|---|---|---|---|
| C | -2.330705000 | -1.533047000 | 0.499760000 |
| C | -1.515847000 | 1.494877000 | -1.709471000 |
| O | -1.140481000 | -1.272488000 | 0.306592000 |
| Fe | 0.534888000 | -0.355102000 | -0.166593000 |
| H | -1.052177000 | 2.284087000 | -1.099530000 |
| H | -1.667892000 | 1.932073000 | -2.713988000 |
| C | -2.987829000 | 0.772178000 | 0.324414000 |
| H | -2.066979000 | 1.020286000 | 0.870710000 |
| H | -3.837358000 | 1.266378000 | 0.809408000 |
| C | -2.904383000 | 1.145218000 | -1.161349000 |
| H | -3.358689000 | 0.330253000 | -1.750757000 |
| H | -3.552603000 | 2.020570000 | -1.322241000 |
| C | -2.799116000 | -2.942013000 | 0.696630000 |
| H | -2.027670000 | -3.515591000 | 1.225981000 |
| H | -3.754748000 | -2.970306000 | 1.233825000 |
| H | -2.939391000 | -3.395177000 | -0.298862000 |
| Br | 0.490732000 | 1.579953000 | 1.233375000 |
| Br | 2.865537000 | -0.851867000 | -0.108399000 |
| C | -0.532935000 | 0.368100000 | -1.914395000 |
| H | -0.958073000 | -0.563073000 | -2.315395000 |
| C | 0.825190000 | 0.568254000 | -2.044417000 |
| H | 1.263671000 | 1.554137000 | -1.861589000 |
| H | 1.450462000 | -0.141820000 | -2.591359000 |

**$MeO_2C$-CH=$CH_2$:** E = -306.158109

| | | | |
|---|---|---|---|
| C | -1.493152000 | 0.378901000 | -0.000161000 |
| H | -2.001535000 | 1.346795000 | 0.000301000 |
| C | -2.172366000 | -0.774189000 | -0.000078000 |
| H | -1.653521000 | -1.736041000 | -0.000219000 |
| H | -3.265592000 | -0.785587000 | 0.001261000 |
| C | -0.011673000 | 0.487248000 | -0.000175000 |
| O | 0.581597000 | 1.546067000 | 0.000085000 |
| O | 0.610166000 | -0.703758000 | -0.000067000 |
| C | 2.040126000 | -0.678057000 | 0.000089000 |
| H | 2.421719000 | -0.162575000 | -0.894612000 |
| H | 2.421411000 | -0.159285000 | 0.893024000 |
| H | 2.365798000 | -1.725203000 | 0.002053000 |

**$MeO_2C$-CH=$CH_2$(FeBr2) complex1:** E = -6717.543959

| | | | |
|---|---|---|---|
| C | 0.505453000 | -1.463519000 | -0.325765000 |
| H | -0.086545000 | -1.638884000 | -1.226532000 |
| C | 0.112661000 | -1.952301000 | 0.903870000 |
| H | 0.821498000 | -1.978855000 | 1.738808000 |
| H | -0.806733000 | -2.539822000 | 1.001787000 |
| Fe | -0.643930000 | -0.020476000 | 0.802778000 |
| Br | 0.478953000 | 1.821769000 | -0.075132000 |
| Br | -2.620819000 | -0.375156000 | -0.352063000 |
| C | 1.910537000 | -1.034926000 | -0.615209000 |
| O | 2.343021000 | -0.916907000 | -1.737815000 |
| O | 2.632313000 | -0.845258000 | 0.495262000 |
| C | 3.977207000 | -0.387634000 | 0.314653000 |
| H | 4.551095000 | -1.099208000 | -0.297305000 |
| H | 3.979537000 | 0.595198000 | -0.180473000 |
| H | 4.410817000 | -0.309931000 | 1.318435000 |

**$MeO_2C$-CH=$CH_2$(FeBr2) complex2:** E = -6717.541802

| | | | |
|---|---|---|---|
| C | 2.376744000 | -0.730086000 | 0.119465000 |
| O | 1.710879000 | 0.081258000 | -0.543805000 |
| Fe | -0.266876000 | -0.170605000 | -0.283232000 |
| Br | -0.566002000 | 2.123455000 | 0.263819000 |

| | | | |
|---|---|---|---|
| Br | -2.443442000 | -1.093438000 | -0.294103000 |
| C | 1.701484000 | -1.668365000 | 1.035935000 |
| H | 2.204314000 | -2.595818000 | 1.317556000 |
| C | 0.453532000 | -1.352784000 | 1.433978000 |
| H | 0.081878000 | -0.279402000 | 1.406806000 |
| H | -0.158765000 | -1.995915000 | 2.066394000 |
| O | 3.676931000 | -0.816155000 | 0.051279000 |
| C | 4.369293000 | 0.109588000 | -0.816422000 |
| H | 5.432477000 | -0.131991000 | -0.718154000 |
| H | 4.030232000 | -0.023637000 | -1.853054000 |
| H | 4.170408000 | 1.140938000 | -0.493087000 |

**$MeO_2C-CH=CH_2$(FeBr2) complex3:** E = -6717.549621

| | | | |
|---|---|---|---|
| C | -2.751192000 | -0.516356000 | -1.130779000 |
| H | -1.997490000 | -1.002812000 | -1.754324000 |
| C | -4.060469000 | -0.665180000 | -1.372774000 |
| H | -4.812535000 | -0.178411000 | -0.746920000 |
| H | -4.408814000 | -1.280512000 | -2.205750000 |
| C | -2.220240000 | 0.296490000 | -0.024291000 |
| O | -1.011489000 | 0.447136000 | 0.207845000 |
| O | -3.115419000 | 0.887092000 | 0.734450000 |
| C | -2.639859000 | 1.708905000 | 1.819381000 |
| H | -2.091419000 | 1.092400000 | 2.545351000 |
| H | -1.973757000 | 2.493986000 | 1.434977000 |
| H | -3.535352000 | 2.143865000 | 2.275675000 |
| Fe | 0.601611000 | -0.148907000 | -0.654757000 |
| Br | 1.038762000 | -2.106672000 | 0.547257000 |
| Br | 1.996190000 | 1.677703000 | -0.199054000 |

# NMR spectra

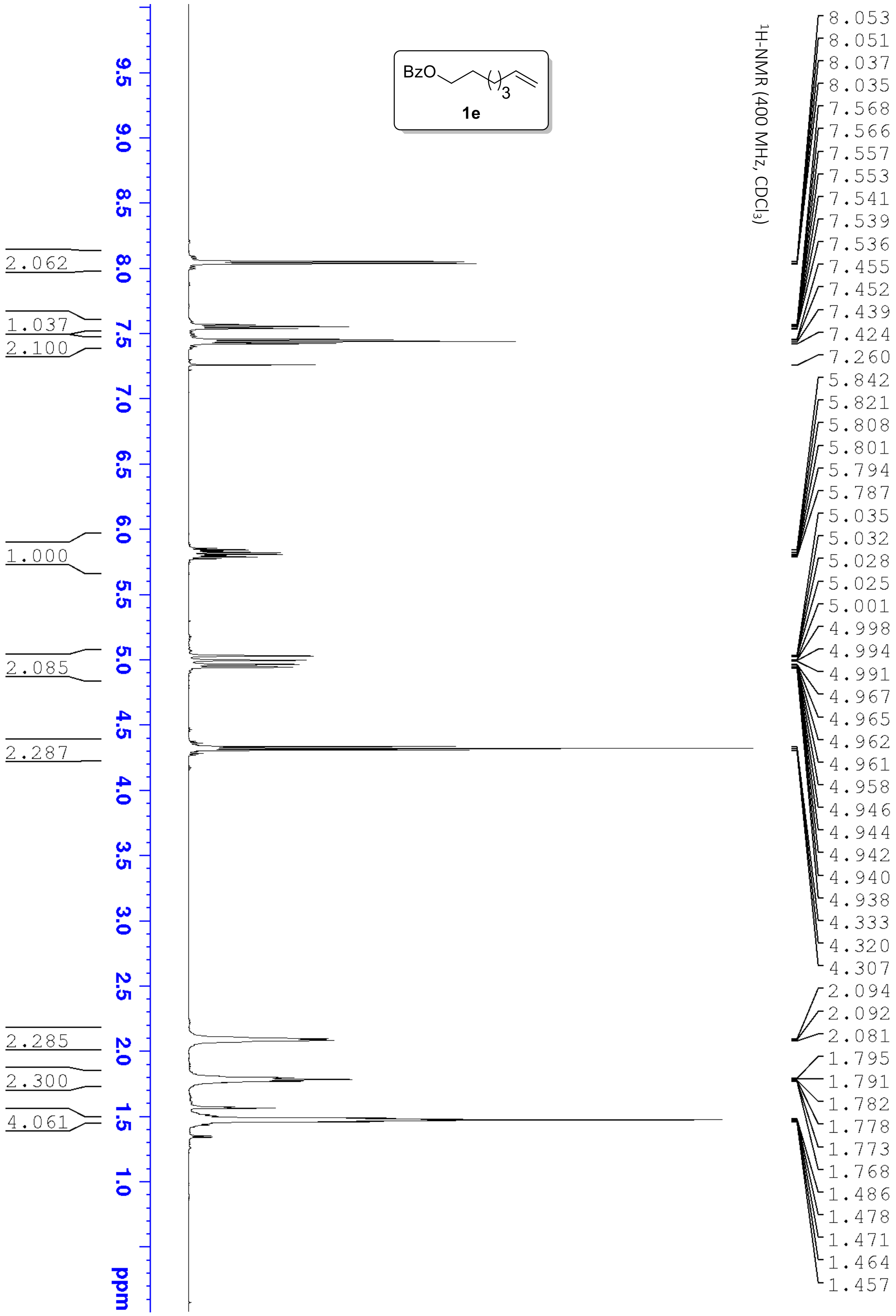

1H-NMR (400 MHz, CDCl3)
BzO
3
1e
8.053
8.051
8.037
8.035
7.568
7.566
7.557
7.553
7.541
7.539
7.536
7.455
7.452
7.439
7.424
7.260
5.842
5.821
5.808
5.801
5.794
5.787
5.035
5.032
5.028
5.025
5.001
4.998
4.994
4.991
4.967
4.965
4.962
4.961
4.958
4.946
4.944
4.942
4.940
4.938
4.333
4.320
4.307
2.094
2.092
2.081
1.795
1.791
1.782
1.778
1.773
1.768
1.486
1.478
1.471
1.464
1.457
2.062
1.037
2.100
1.000
2.085
2.287
2.285
2.300
4.061
9.5
9.0
8.5
8.0
7.5
7.0
6.5
6.0
5.5
5.0
4.5
4.0
3.5
3.0
2.5
2.0
1.5
1.0
ppm

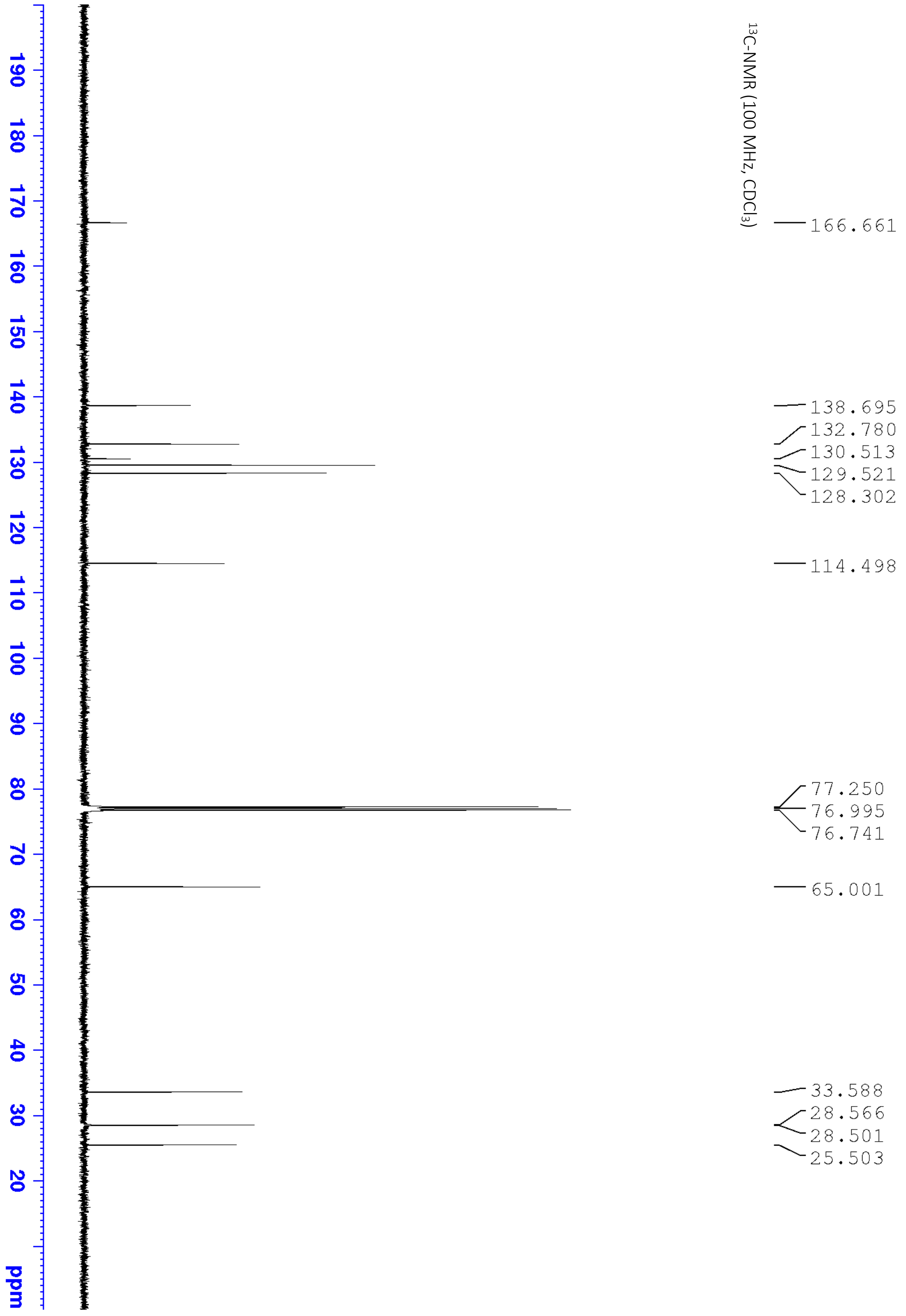

13C-NMR (100 MHz, CDCl3)
166.661
138.695
132.780
130.513
129.521
128.302
114.498
77.250
76.995
76.741
65.001
33.588
28.566
28.501
25.503
190
180
170
160
150
140
130
120
110
100
90
80
70
60
50
40
30
20
ppm

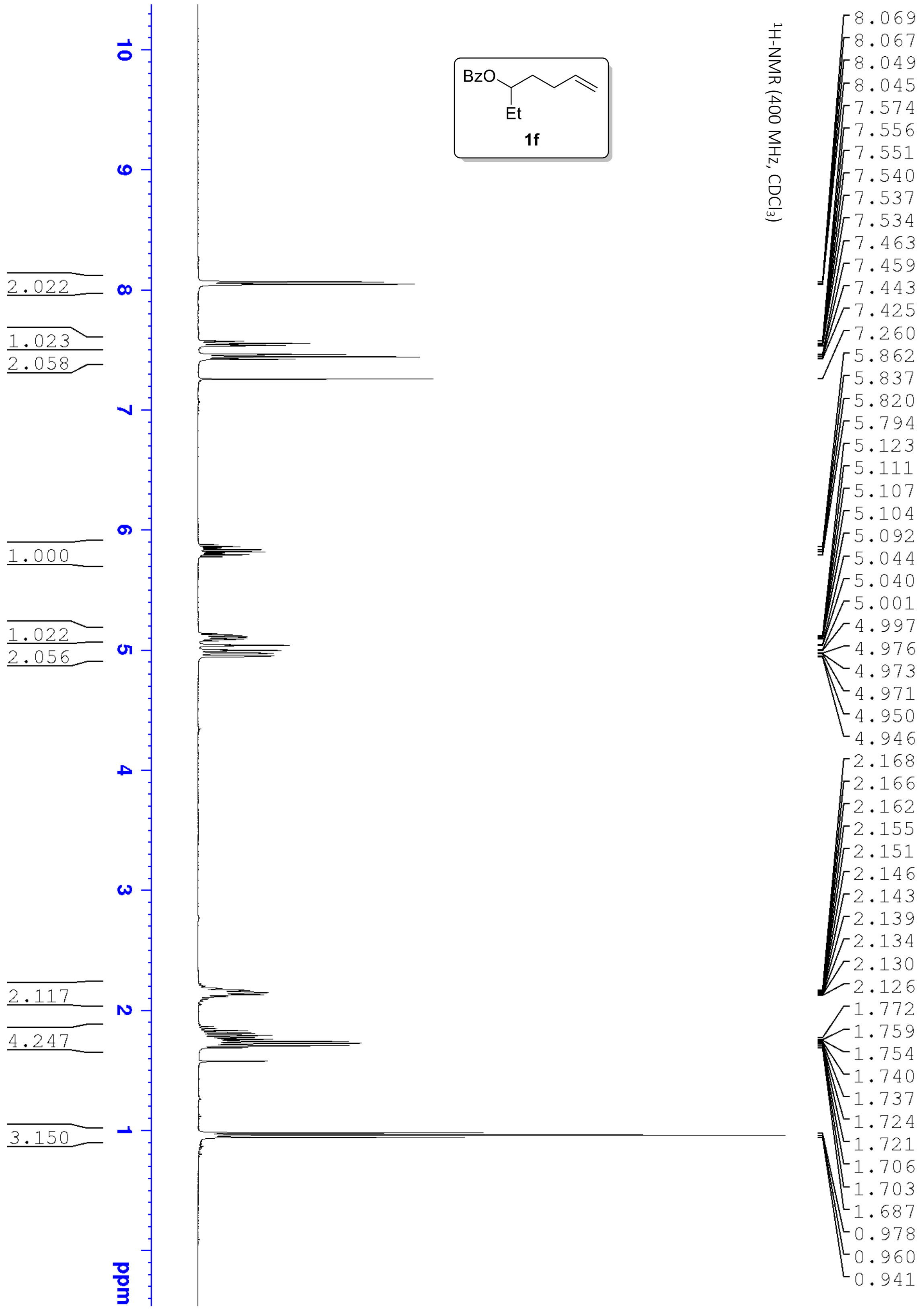

1H-NMR (400 MHz, CDCl3)
BzO
Et
1f
8.069
8.067
8.049
8.045
7.574
7.556
7.551
7.540
7.537
7.534
7.463
7.459
7.443
7.425
7.260
5.862
5.837
5.820
5.794
5.123
5.111
5.107
5.104
5.092
5.044
5.040
5.001
4.997
4.976
4.973
4.971
4.950
4.946
2.168
2.166
2.162
2.155
2.151
2.146
2.143
2.139
2.134
2.130
2.126
1.772
1.759
1.754
1.740
1.737
1.724
1.721
1.706
1.703
1.687
0.978
0.960
0.941
2.022
1.023
2.058
1.000
1.022
2.056
2.117
4.247
3.150
10
9
8
7
6
5
4
3
2
1
ppm

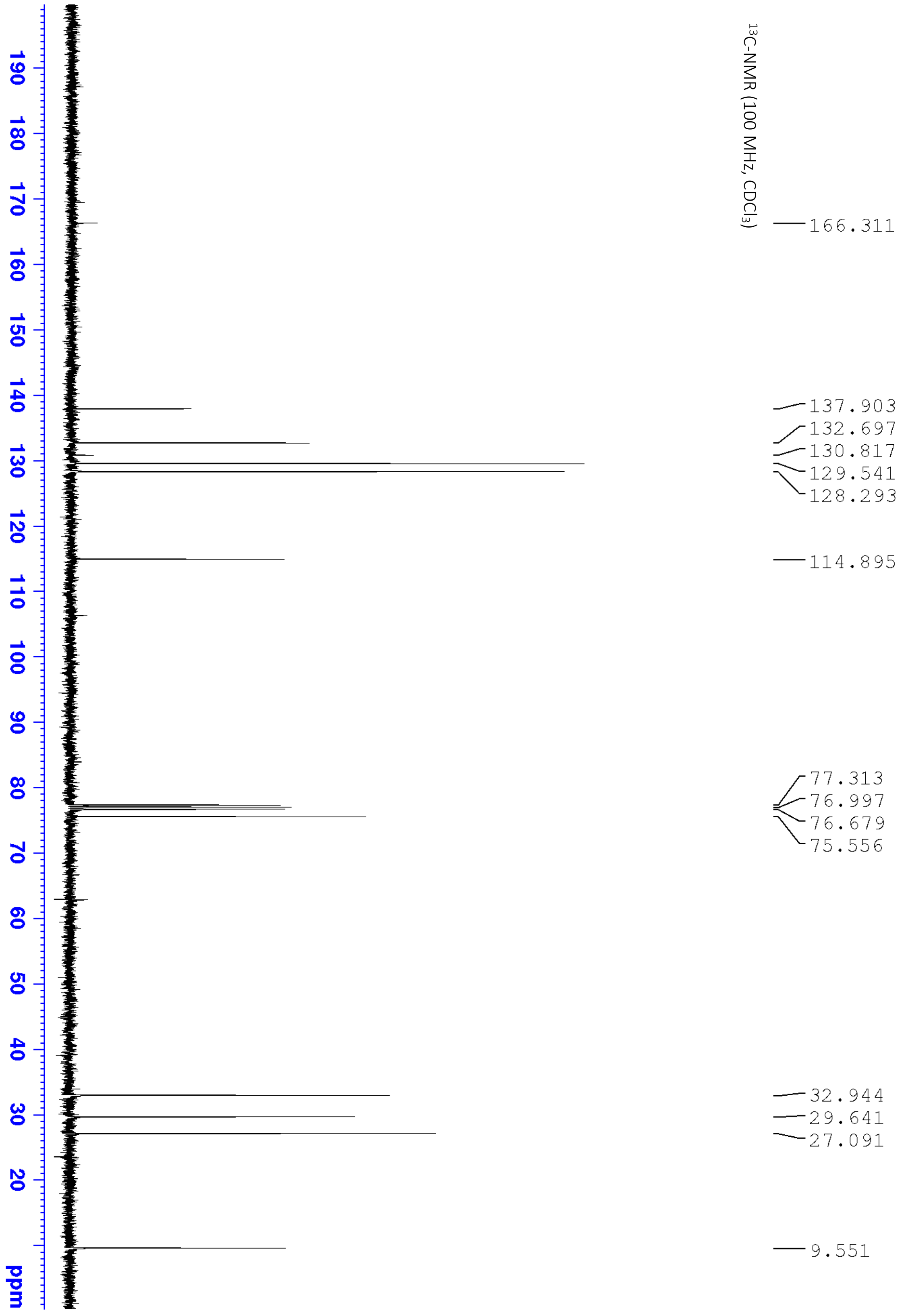

13C-NMR (100 MHz, CDCl3)
166.311
137.903
132.697
130.817
129.541
128.293
114.895
77.313
76.997
76.679
75.556
32.944
29.641
27.091
9.551
ppm

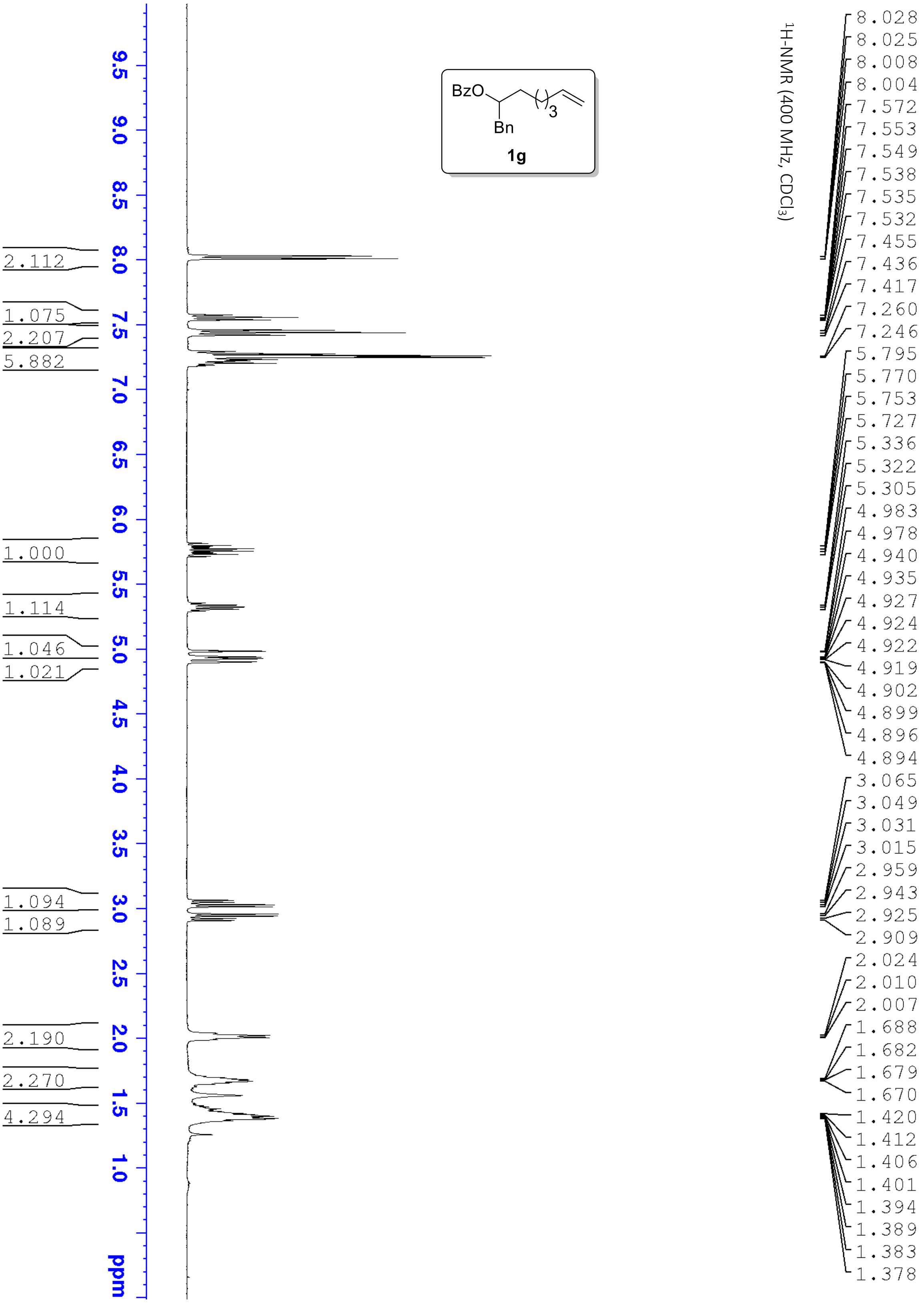

1H-NMR (400 MHz, CDCl3)
BzO
Bn
3
1g
8.028
8.025
8.008
8.004
7.572
7.553
7.549
7.538
7.535
7.532
7.455
7.436
7.417
7.260
7.246
5.795
5.770
5.753
5.727
5.336
5.322
5.305
4.983
4.978
4.940
4.935
4.927
4.924
4.922
4.919
4.902
4.899
4.896
4.894
3.065
3.049
3.031
3.015
2.959
2.943
2.925
2.909
2.024
2.010
2.007
1.688
1.682
1.679
1.670
1.420
1.412
1.406
1.401
1.394
1.389
1.383
1.378
2.112
1.075
2.207
5.882
1.000
1.114
1.046
1.021
1.094
1.089
2.190
2.270
4.294
9.5
9.0
8.5
8.0
7.5
7.0
6.5
6.0
5.5
5.0
4.5
4.0
3.5
3.0
2.5
2.0
1.5
1.0
ppm

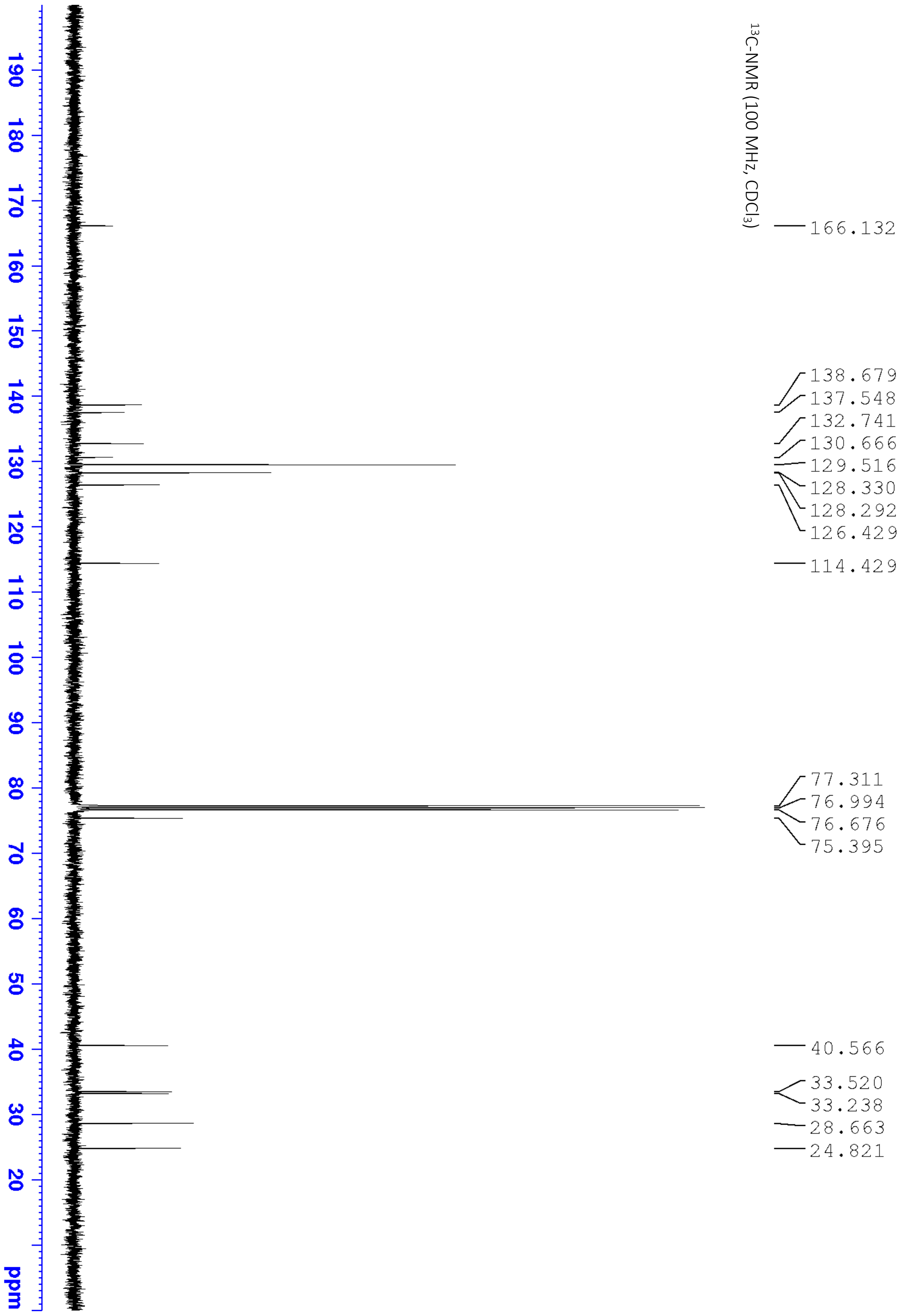

13C-NMR (100 MHz, CDCl3)
166.132
138.679
137.548
132.741
130.666
129.516
128.330
128.292
126.429
114.429
77.311
76.994
76.676
75.395
40.566
33.520
33.238
28.663
24.821
ppm

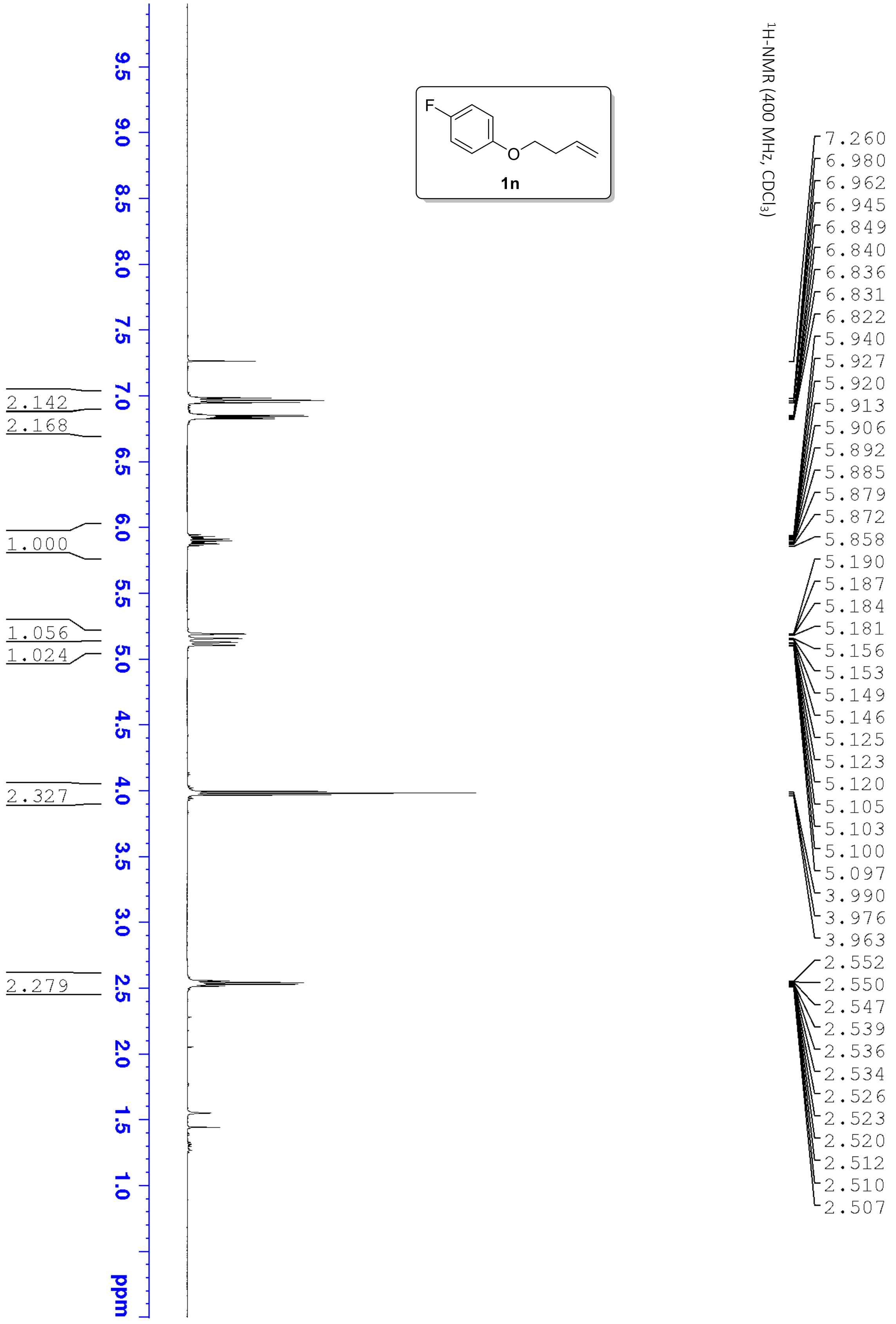

F
O
1n
1H-NMR (400 MHz, CDCl3)
7.260
6.980
6.962
6.945
6.849
6.840
6.836
6.831
6.822
5.940
5.927
5.920
5.913
5.906
5.892
5.885
5.879
5.872
5.858
5.190
5.187
5.184
5.181
5.156
5.153
5.149
5.146
5.125
5.123
5.120
5.105
5.103
5.100
5.097
3.990
3.976
3.963
2.552
2.550
2.547
2.539
2.536
2.534
2.526
2.523
2.520
2.512
2.510
2.507
2.142
2.168
1.000
1.056
1.024
2.327
2.279
9.5
9.0
8.5
8.0
7.5
7.0
6.5
6.0
5.5
5.0
4.5
4.0
3.5
3.0
2.5
2.0
1.5
1.0
ppm

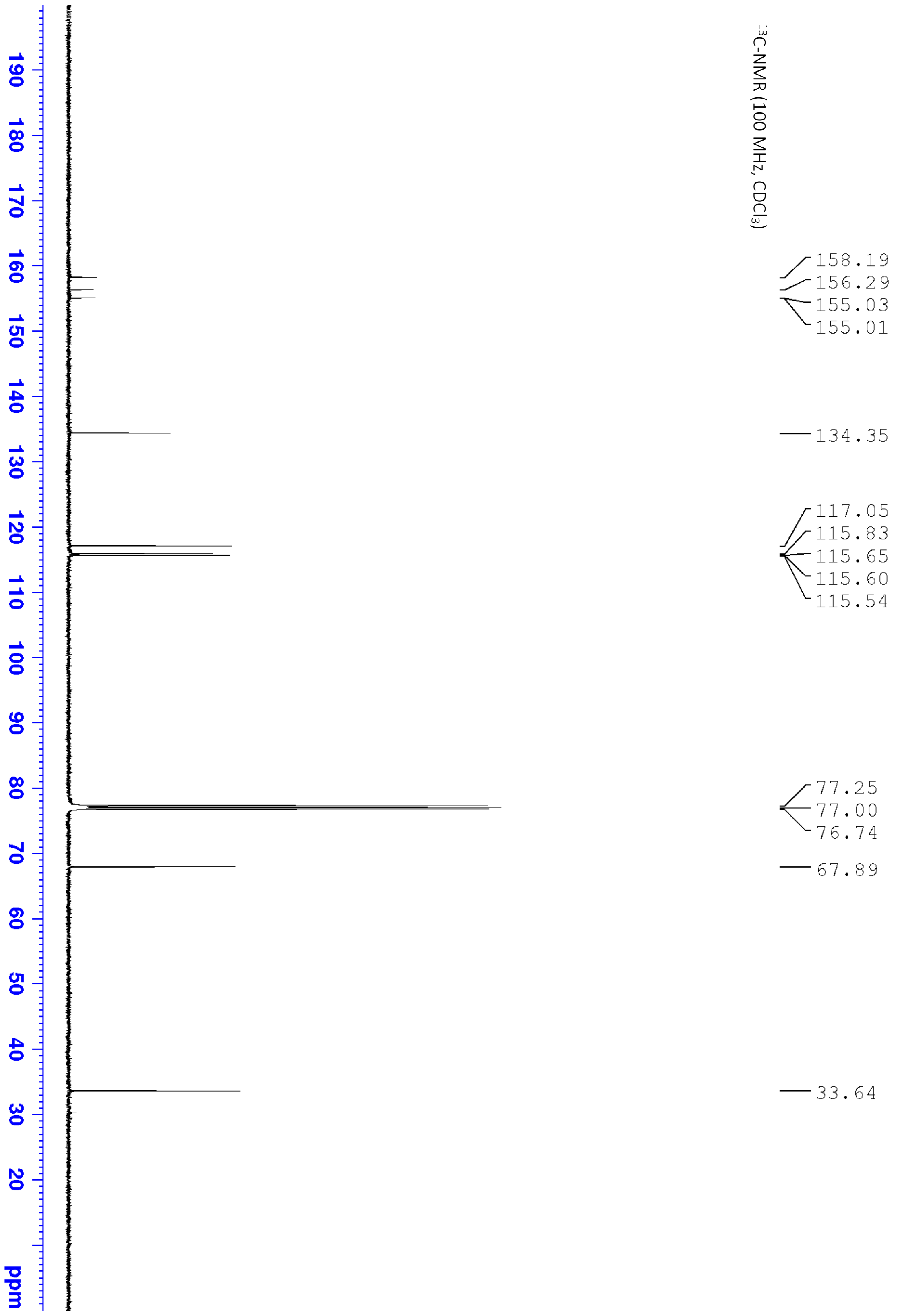

13C-NMR (100 MHz, CDCl3)
158.19
156.29
155.03
155.01
134.35
117.05
115.83
115.65
115.60
115.54
77.25
77.00
76.74
67.89
33.64
190
180
170
160
150
140
130
120
110
100
90
80
70
60
50
40
30
20
ppm

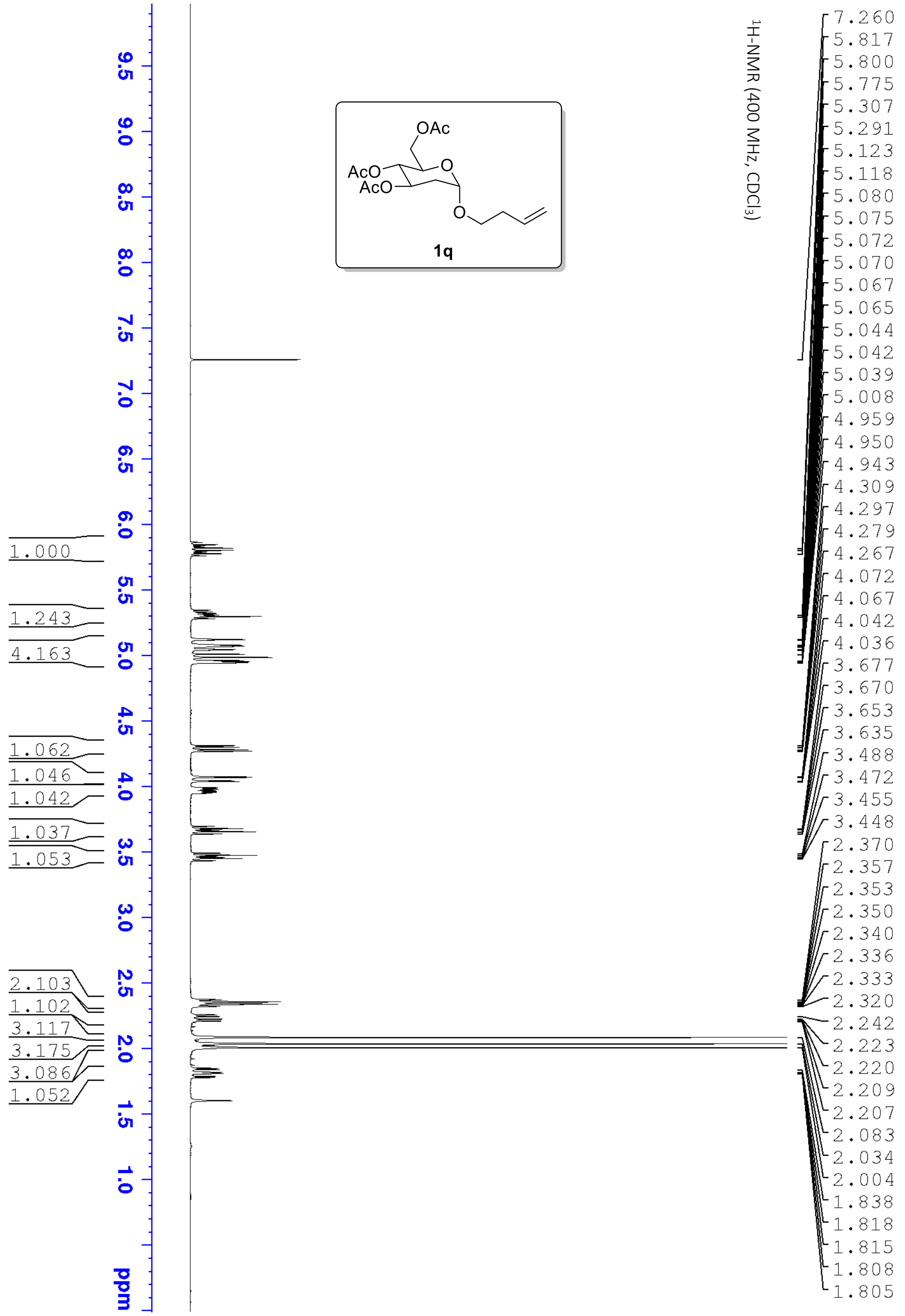
1H-NMR (400 MHz, CDCl3)
OAc
AcO
AcO
O
O
1q
7.260
5.817
5.800
5.775
5.307
5.291
5.123
5.118
5.080
5.075
5.072
5.070
5.067
5.065
5.044
5.042
5.039
5.008
4.959
4.950
4.943
4.309
4.297
4.279
4.267
4.072
4.067
4.042
4.036
3.677
3.670
3.653
3.635
3.488
3.472
3.455
3.448
2.370
2.357
2.353
2.350
2.340
2.336
2.333
2.320
2.242
2.223
2.220
2.209
2.207
2.083
2.034
2.004
1.838
1.818
1.815
1.808
1.805
1.000
1.243
4.163
1.062
1.046
1.042
1.037
1.053
2.103
1.102
3.117
3.175
3.086
1.052
9.5
9.0
8.5
8.0
7.5
7.0
6.5
6.0
5.5
5.0
4.5
4.0
3.5
3.0
2.5
2.0
1.5
1.0
ppm

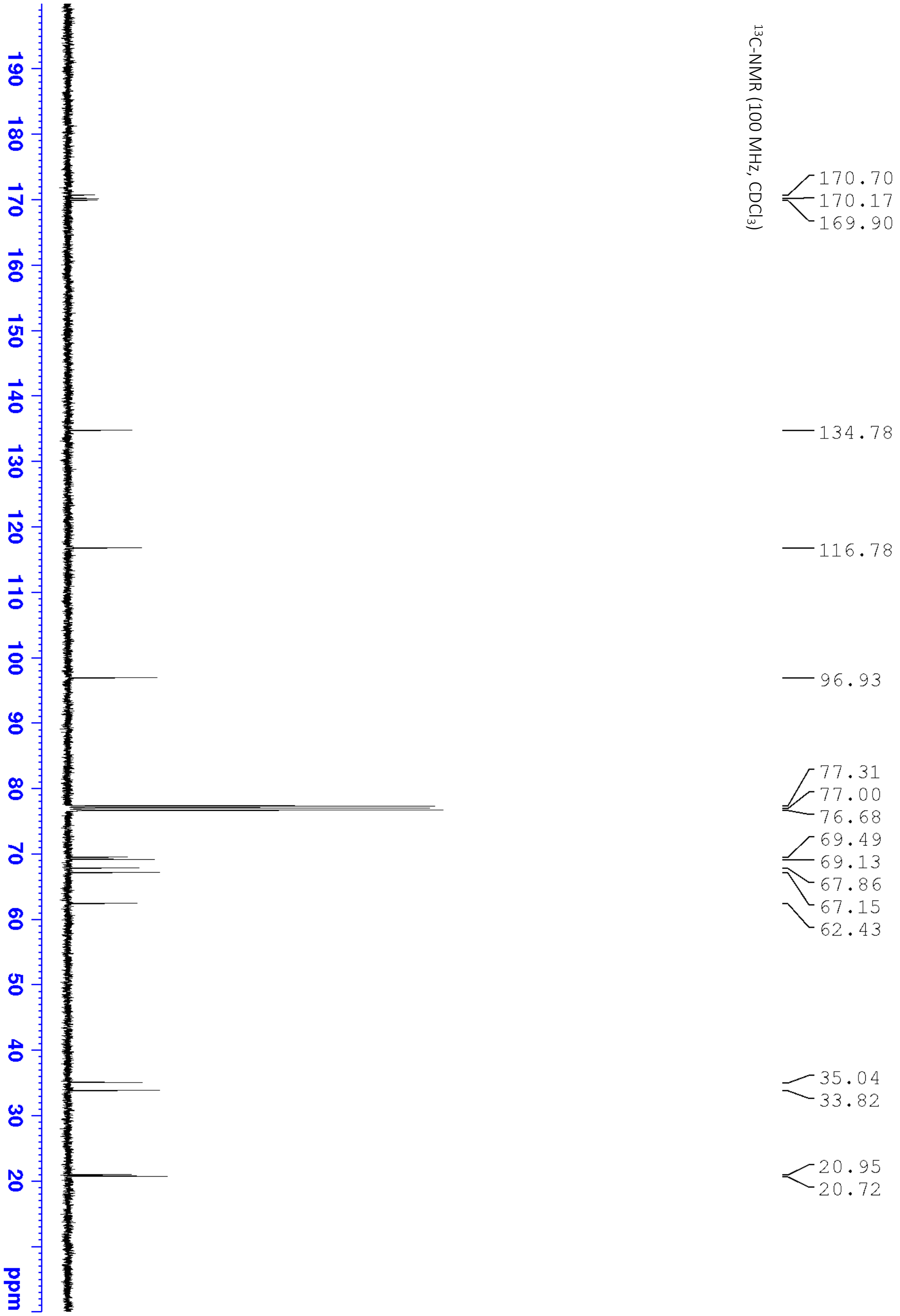
13C-NMR (100 MHz, CDCl3)
170.70
170.17
169.90
134.78
116.78
96.93
77.31
77.00
76.68
69.49
69.13
67.86
67.15
62.43
35.04
33.82
20.95
20.72
190
180
170
160
150
140
130
120
110
100
90
80
70
60
50
40
30
20
ppm

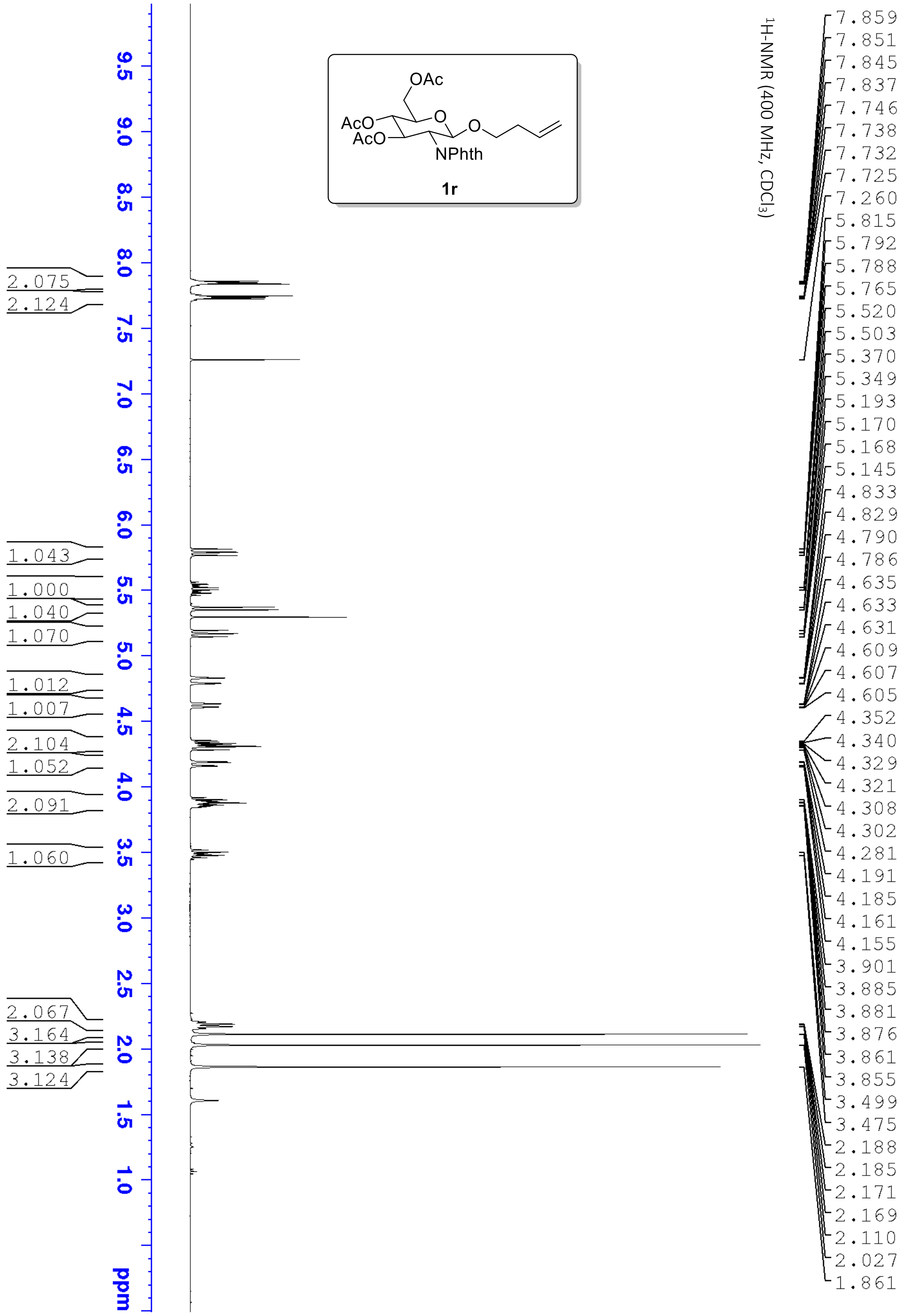

1H-NMR (400 MHz, CDCl3)
OAc
AcO
AcO
O
O
NPhth
1r
7.859 7.851 7.845 7.837 7.746 7.738 7.732 7.725 7.260 5.815 5.792 5.788 5.765 5.520 5.503 5.370 5.349 5.193 5.170 5.168 5.145 4.833 4.829 4.790 4.786 4.635 4.633 4.631 4.609 4.607 4.605 4.352 4.340 4.329 4.321 4.308 4.302 4.281 4.191 4.185 4.161 4.155 3.901 3.885 3.881 3.876 3.861 3.855 3.499 3.475 2.188 2.185 2.171 2.169 2.110 2.027 1.861
2.075
2.124
1.043
1.000
1.040
1.070
1.012
1.007
2.104
1.052
2.091
1.060
2.067
3.164
3.138
3.124
9.5 9.0 8.5 8.0 7.5 7.0 6.5 6.0 5.5 5.0 4.5 4.0 3.5 3.0 2.5 2.0 1.5 1.0 ppm

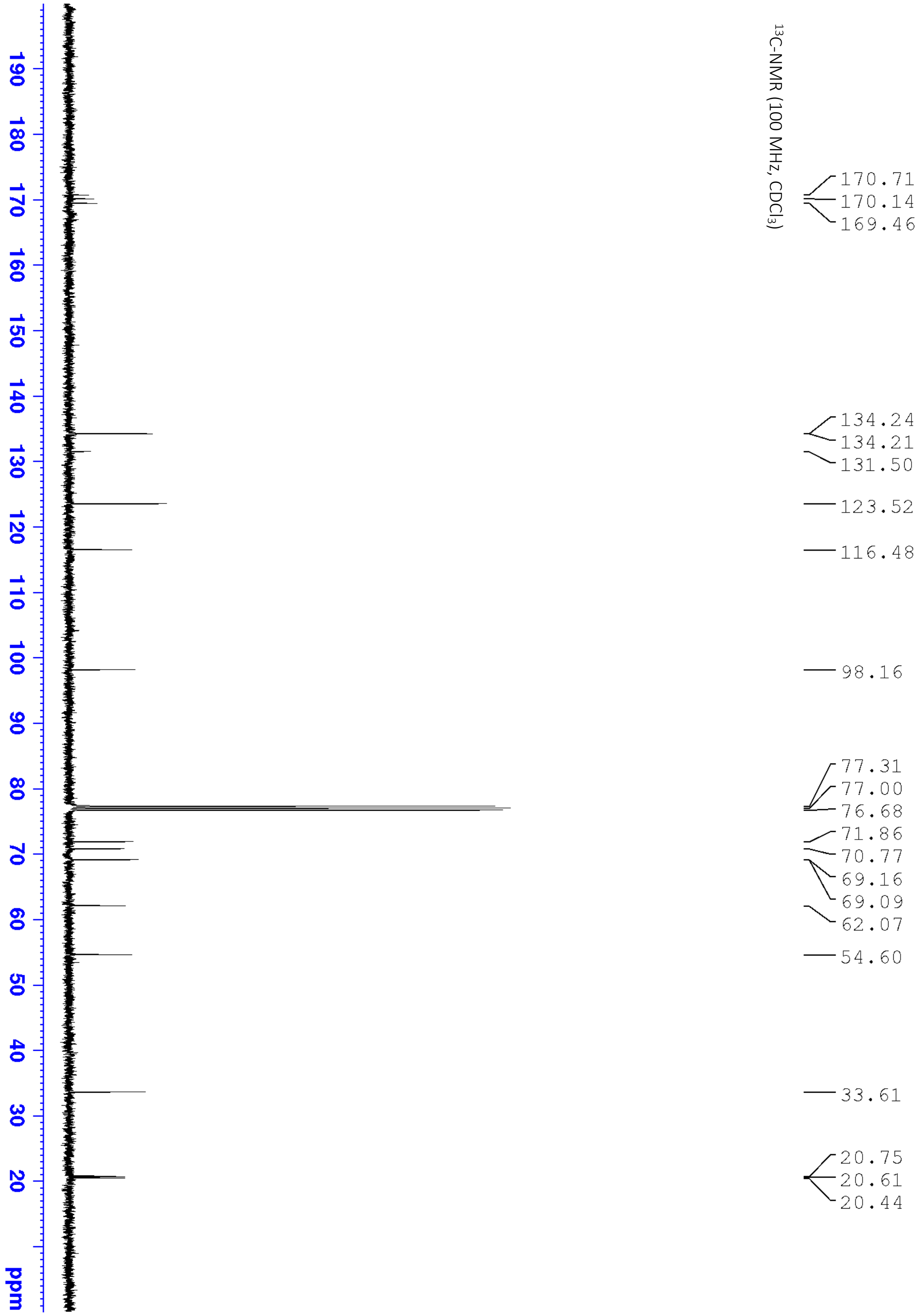

$^{13}C$-NMR (100 MHz, $CDCl_3$)
170.71
170.14
169.46
134.24
134.21
131.50
123.52
116.48
98.16
77.31
77.00
76.68
71.86
70.77
69.16
69.09
62.07
54.60
33.61
20.75
20.61
20.44
190
180
170
160
150
140
130
120
110
100
90
80
70
60
50
40
30
20
ppm

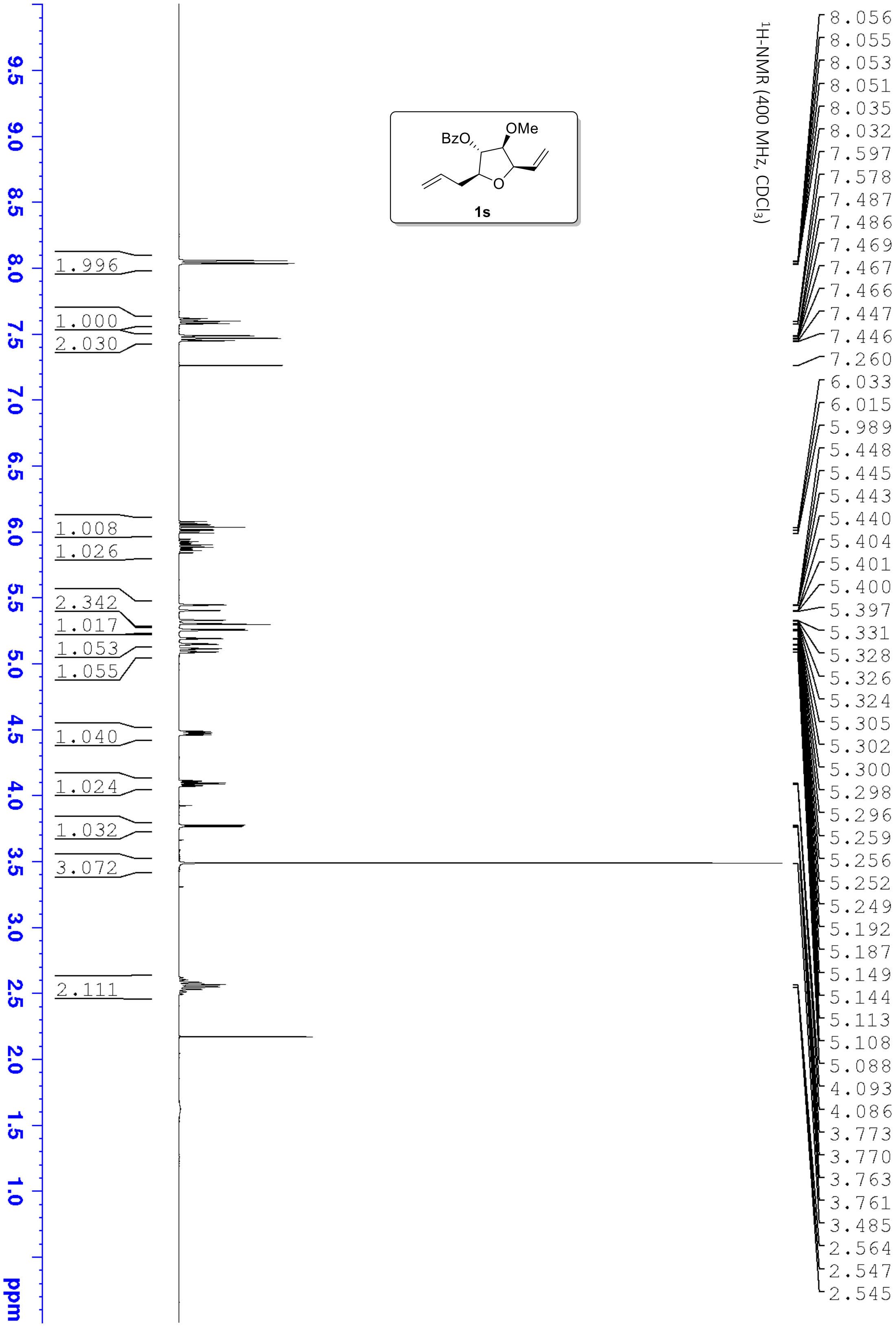

1H-NMR (400 MHz, CDCl3)
BzO
OMe
O
1s
8.056
8.055
8.053
8.051
8.035
8.032
7.597
7.578
7.487
7.486
7.469
7.467
7.466
7.447
7.446
7.260
6.033
6.015
5.989
5.448
5.445
5.443
5.440
5.404
5.401
5.400
5.397
5.331
5.328
5.326
5.324
5.305
5.302
5.300
5.298
5.296
5.259
5.256
5.252
5.249
5.192
5.187
5.149
5.144
5.113
5.108
5.088
4.093
4.086
3.773
3.770
3.763
3.761
3.485
2.564
2.547
2.545
1.996
1.000
2.030
1.008
1.026
2.342
1.017
1.053
1.055
1.040
1.024
1.032
3.072
2.111
9.5
9.0
8.5
8.0
7.5
7.0
6.5
6.0
5.5
5.0
4.5
4.0
3.5
3.0
2.5
2.0
1.5
1.0
ppm

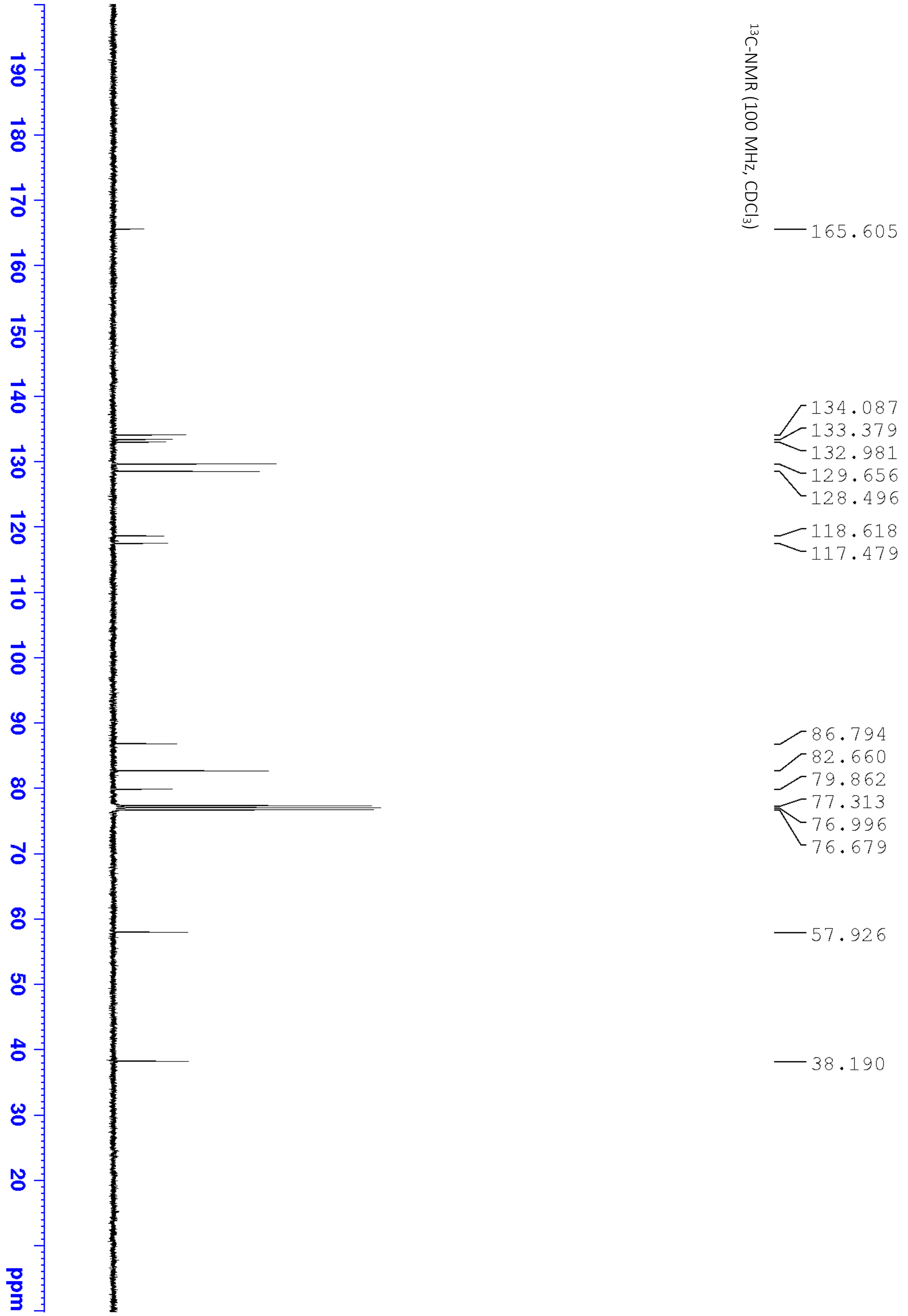

13C-NMR (100 MHz, CDCl3)
165.605
134.087
133.379
132.981
129.656
128.496
118.618
117.479
86.794
82.660
79.862
77.313
76.996
76.679
57.926
38.190
ppm

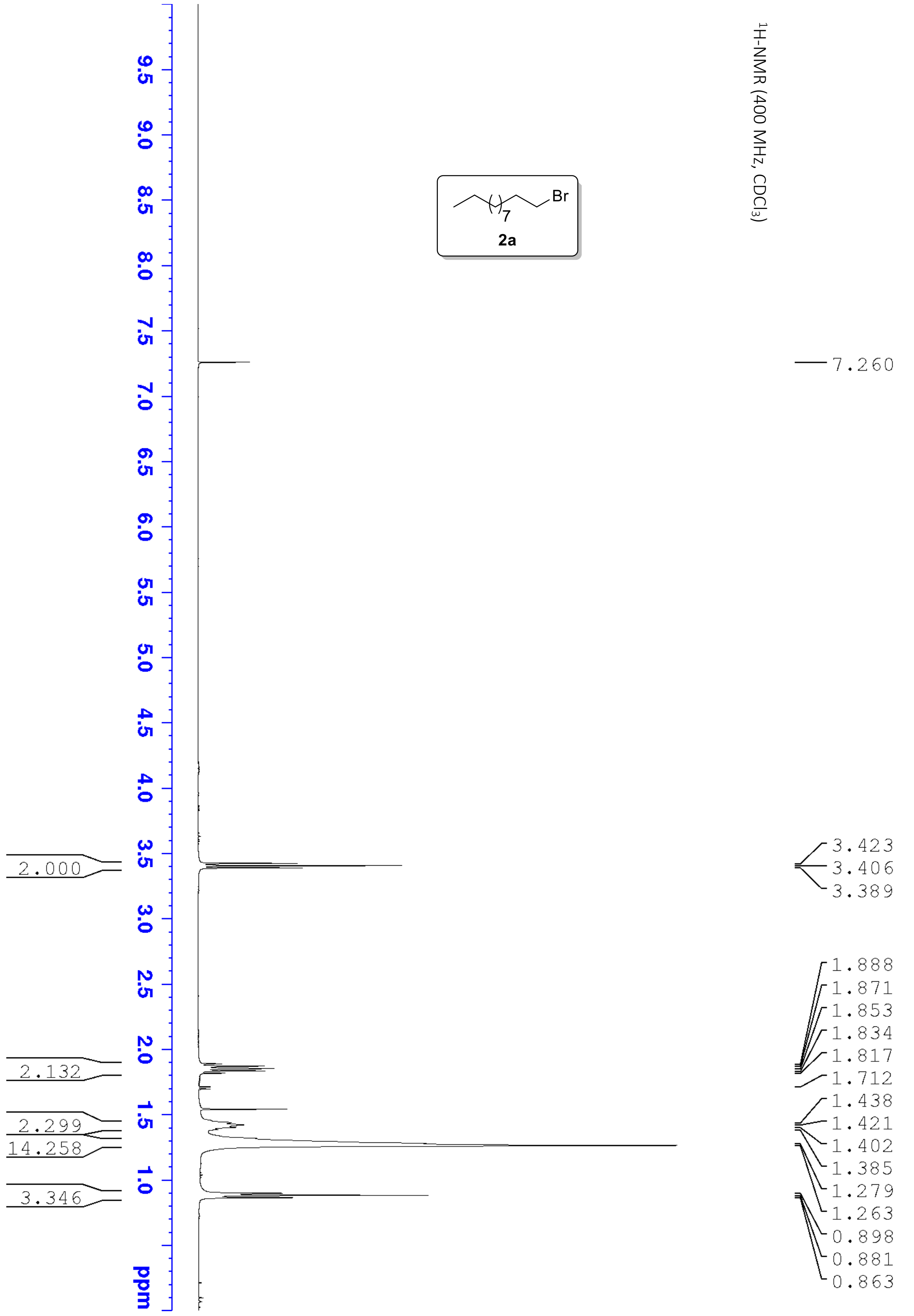
1H-NMR (400 MHz, CDCl3)
Br
7
2a
7.260
3.423
3.406
3.389
1.888
1.871
1.853
1.834
1.817
1.712
1.438
1.421
1.402
1.385
1.279
1.263
0.898
0.881
0.863
2.000
2.132
2.299
14.258
3.346
9.5
9.0
8.5
8.0
7.5
7.0
6.5
6.0
5.5
5.0
4.5
4.0
3.5
3.0
2.5
2.0
1.5
1.0
ppm

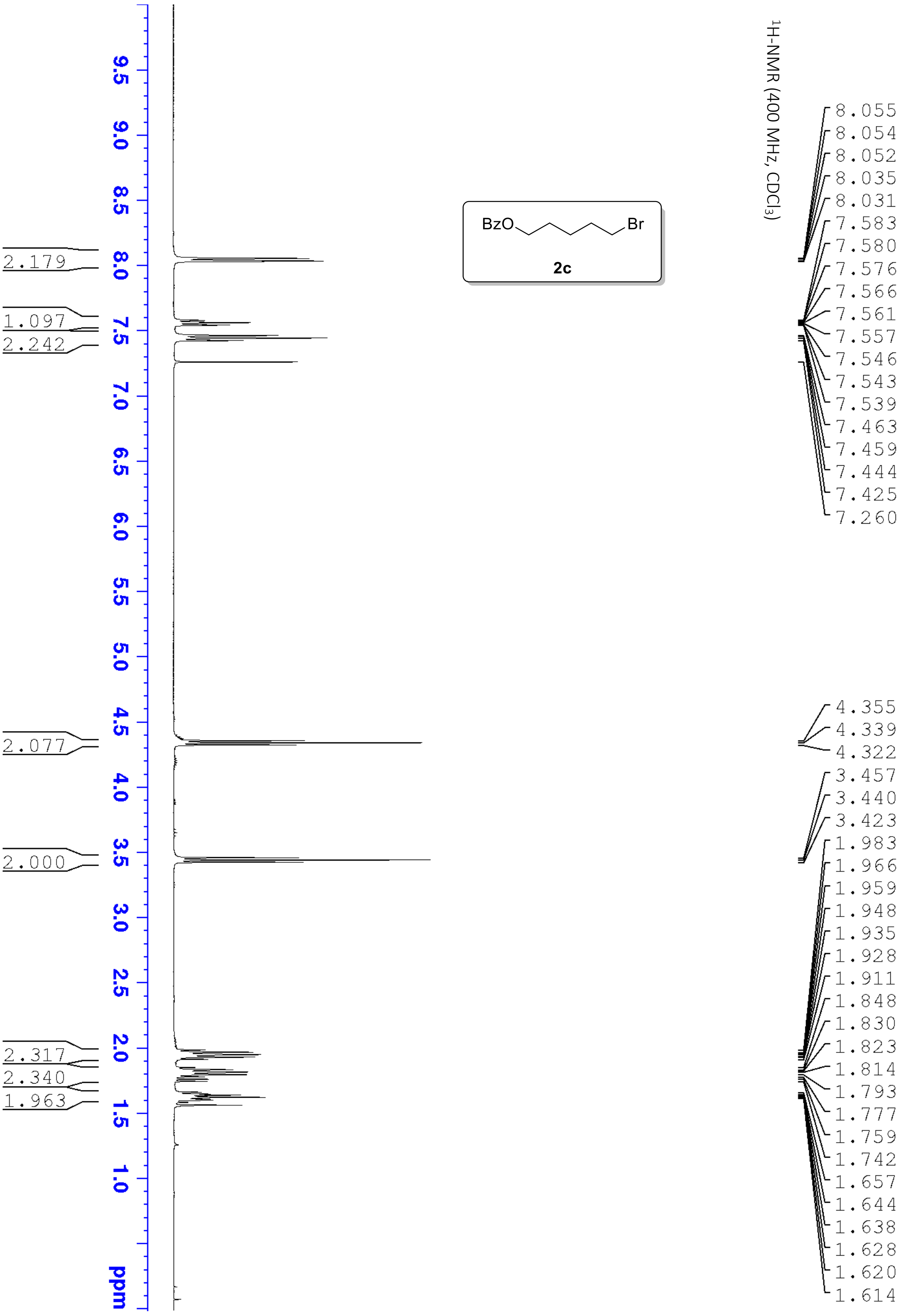

¹H-NMR (400 MHz, CDCl₃)
BzO
Br
2c
8.055
8.054
8.052
8.035
8.031
7.583
7.580
7.576
7.566
7.561
7.557
7.546
7.543
7.539
7.463
7.459
7.444
7.425
7.260
4.355
4.339
4.322
3.457
3.440
3.423
1.983
1.966
1.959
1.948
1.935
1.928
1.911
1.848
1.830
1.823
1.814
1.793
1.777
1.759
1.742
1.657
1.644
1.638
1.628
1.620
1.614
2.179
1.097
2.242
2.077
2.000
2.317
2.340
1.963
9.5
9.0
8.5
8.0
7.5
7.0
6.5
6.0
5.5
5.0
4.5
4.0
3.5
3.0
2.5
2.0
1.5
1.0
ppm

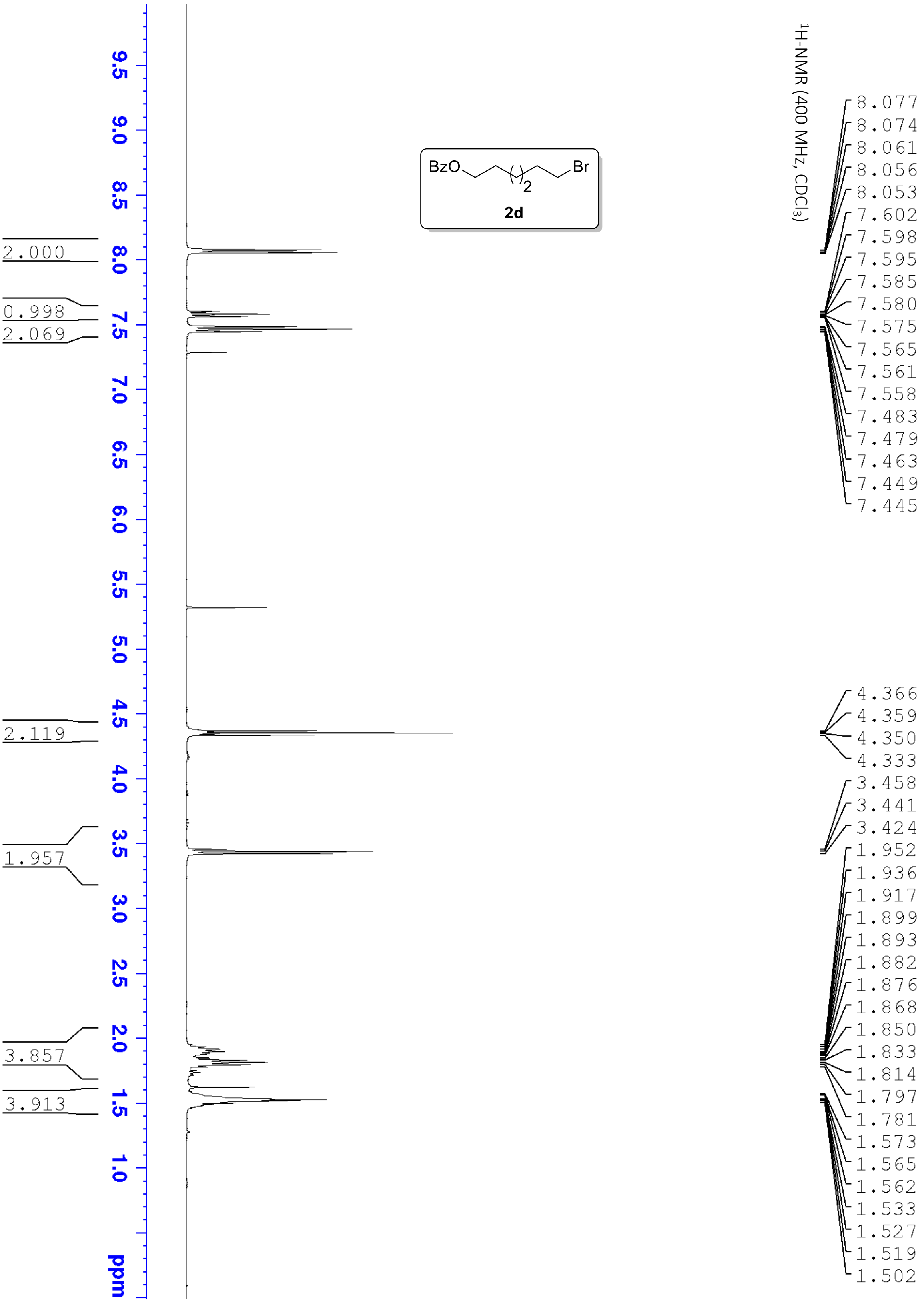
BzO
Br
2
2d
1H-NMR (400 MHz, CDCl3)
8.077
8.074
8.061
8.056
8.053
7.602
7.598
7.595
7.585
7.580
7.575
7.565
7.561
7.558
7.483
7.479
7.463
7.449
7.445
4.366
4.359
4.350
4.333
3.458
3.441
3.424
1.952
1.936
1.917
1.899
1.893
1.882
1.876
1.868
1.850
1.833
1.814
1.797
1.781
1.573
1.565
1.562
1.533
1.527
1.519
1.502
2.000
0.998
2.069
2.119
1.957
3.857
3.913
9.5
9.0
8.5
8.0
7.5
7.0
6.5
6.0
5.5
5.0
4.5
4.0
3.5
3.0
2.5
2.0
1.5
1.0
ppm

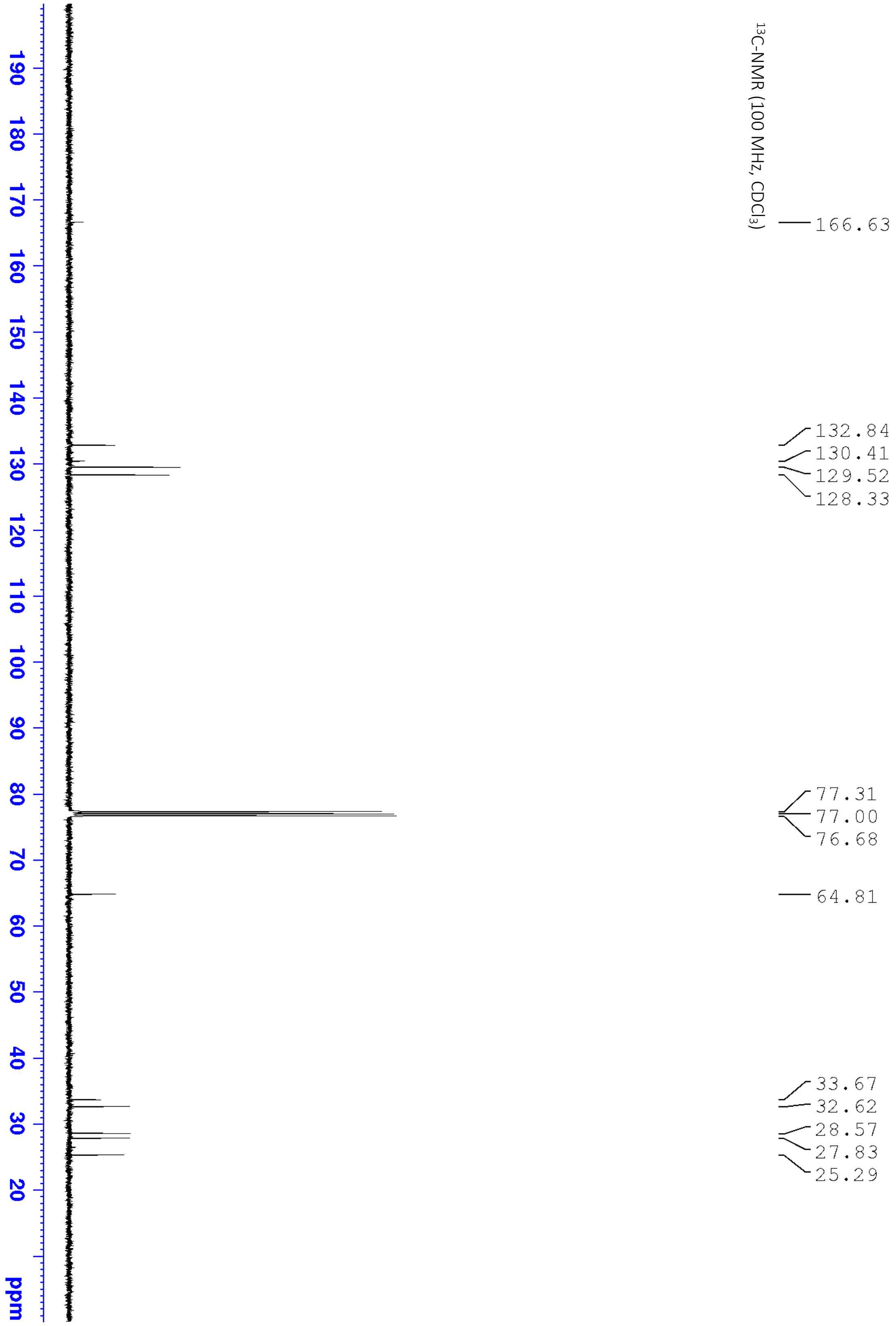

13C-NMR (100 MHz, CDCl3)
166.63
132.84
130.41
129.52
128.33
77.31
77.00
76.68
64.81
33.67
32.62
28.57
27.83
25.29
190
180
170
160
150
140
130
120
110
100
90
80
70
60
50
40
30
20
ppm

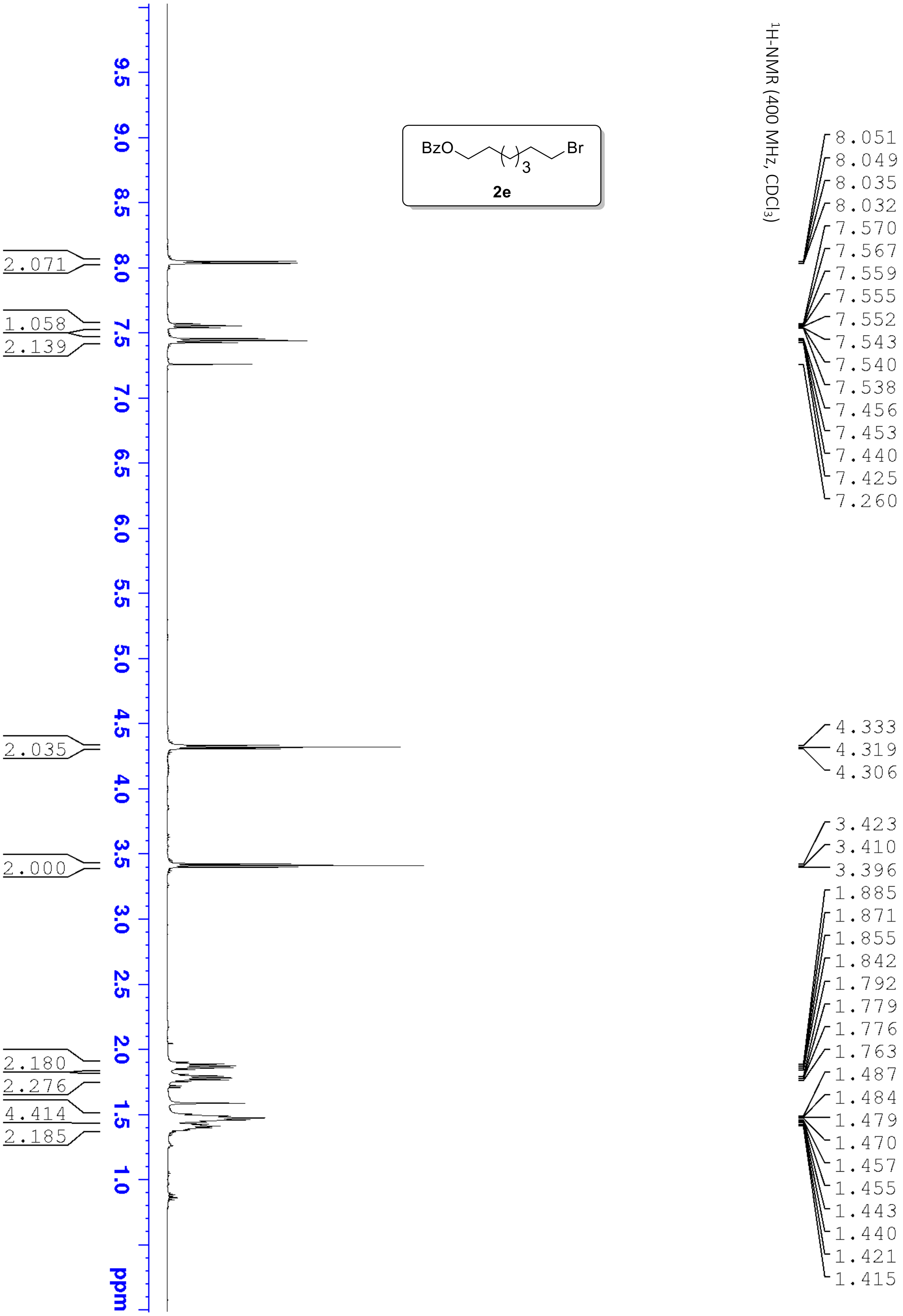
1H-NMR (400 MHz, CDCl3)
BzO
3
Br
2e
ppm

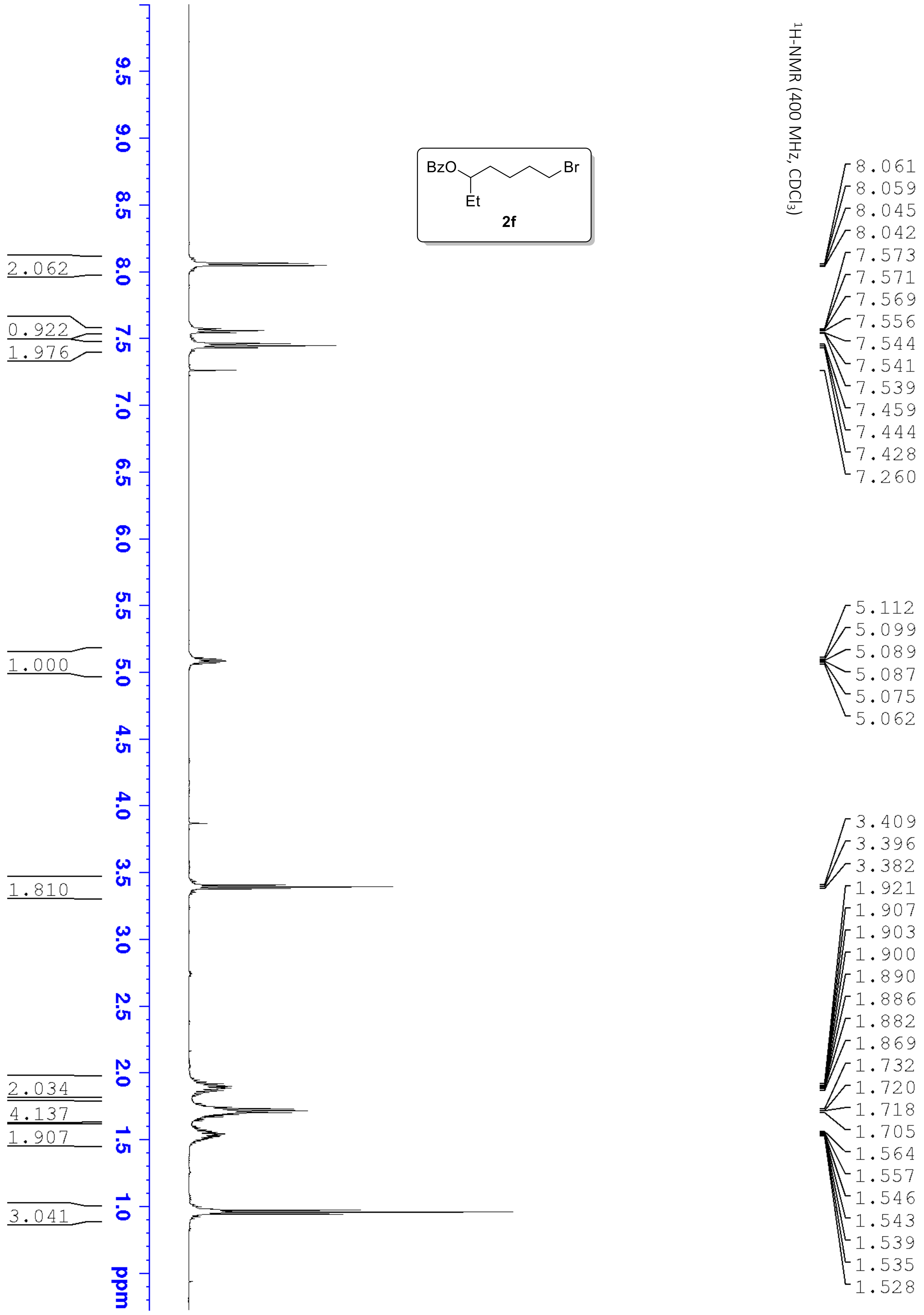
1H-NMR (400 MHz, CDCl3)
BzO
Br
Et
2f
8.061 8.059 8.045 8.042 7.573 7.571 7.569 7.556 7.544 7.541 7.539 7.459 7.444 7.428 7.260
5.112 5.099 5.089 5.087 5.075 5.062
3.409 3.396 3.382 1.921 1.907 1.903 1.900 1.890 1.886 1.882 1.869 1.732 1.720 1.718 1.705 1.564 1.557 1.546 1.543 1.539 1.535 1.528
2.062
0.922
1.976
1.000
1.810
2.034
4.137
1.907
3.041
9.5 9.0 8.5 8.0 7.5 7.0 6.5 6.0 5.5 5.0 4.5 4.0 3.5 3.0 2.5 2.0 1.5 1.0 ppm

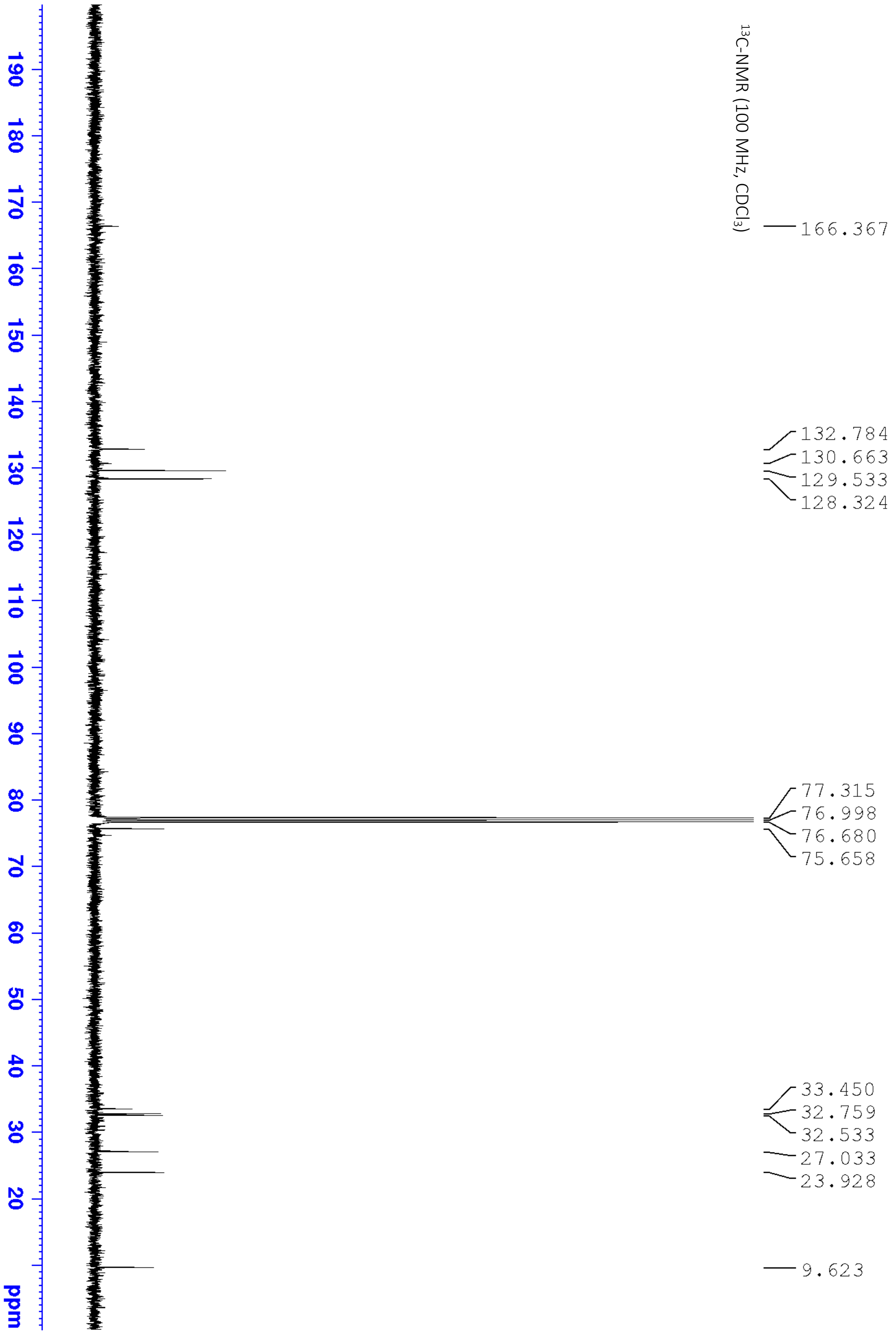

13C-NMR (100 MHz, CDCl3)
166.367
132.784
130.663
129.533
128.324
77.315
76.998
76.680
75.658
33.450
32.759
32.533
27.033
23.928
9.623
190
180
170
160
150
140
130
120
110
100
90
80
70
60
50
40
30
20
ppm

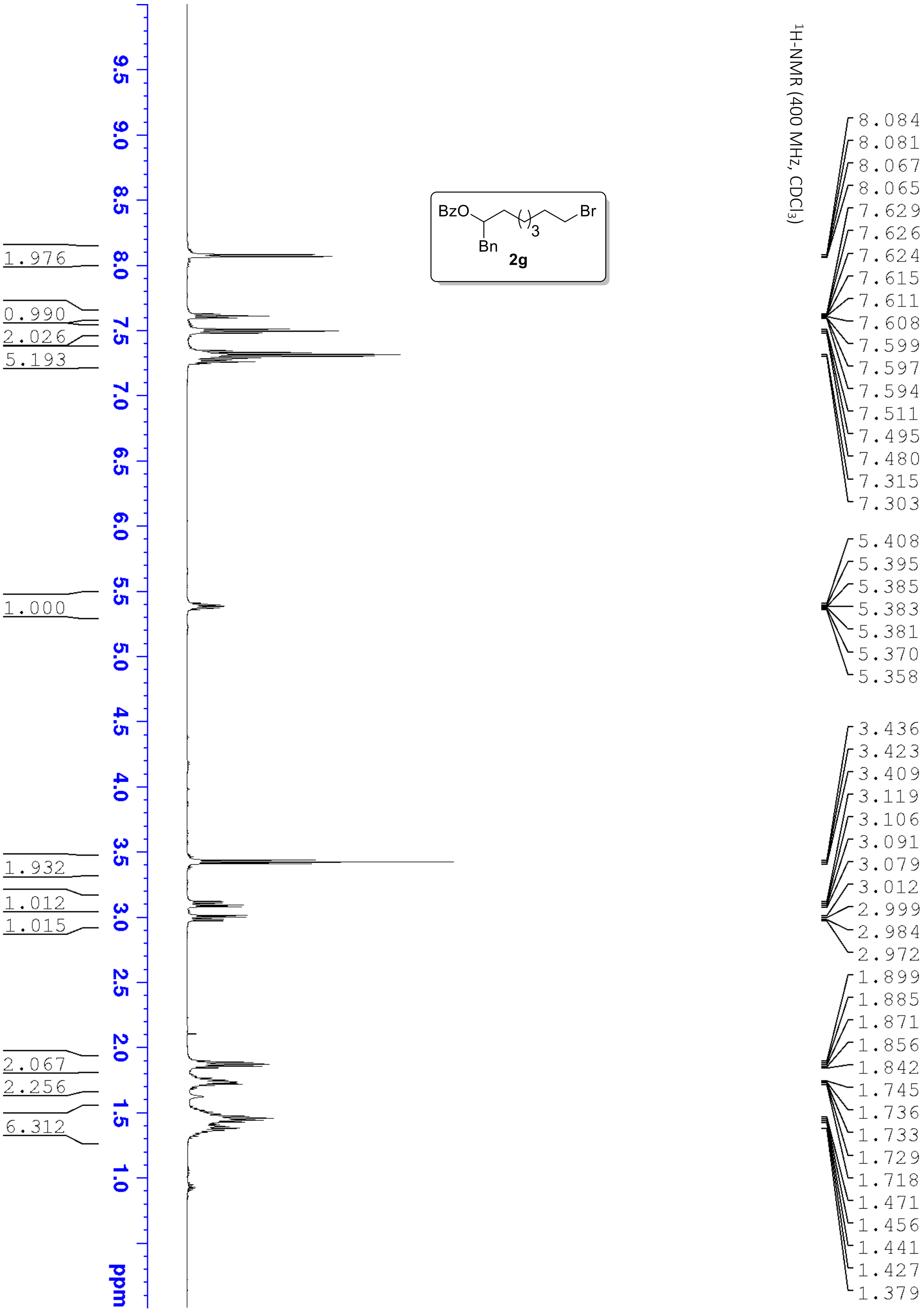
1H-NMR (400 MHz, CDCl3)
BzO
Bn
3
Br
2g
8.084
8.081
8.067
8.065
7.629
7.626
7.624
7.615
7.611
7.608
7.599
7.597
7.594
7.511
7.495
7.480
7.315
7.303
5.408
5.395
5.385
5.383
5.381
5.370
5.358
3.436
3.423
3.409
3.119
3.106
3.091
3.079
3.012
2.999
2.984
2.972
1.899
1.885
1.871
1.856
1.842
1.745
1.736
1.733
1.729
1.718
1.471
1.456
1.441
1.427
1.379
9.5
9.0
8.5
8.0
7.5
7.0
6.5
6.0
5.5
5.0
4.5
4.0
3.5
3.0
2.5
2.0
1.5
1.0
ppm
1.976
0.990
2.026
5.193
1.000
1.932
1.012
1.015
2.067
2.256
6.312

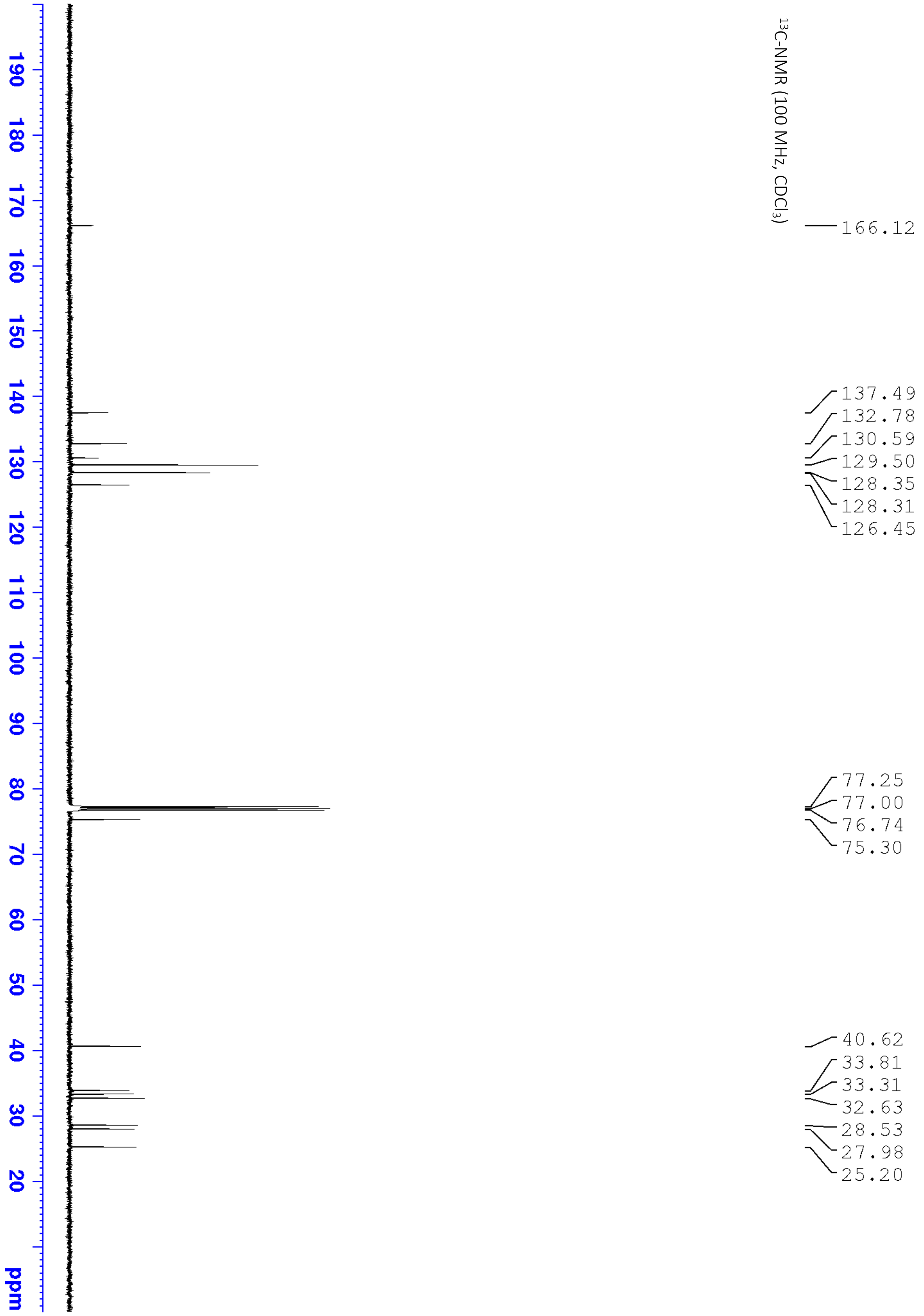

$^{13}$C-NMR (100 MHz, CDCl$_3$)
166.12
137.49
132.78
130.59
129.50
128.35
128.31
126.45
77.25
77.00
76.74
75.30
40.62
33.81
33.31
32.63
28.53
27.98
25.20
190
180
170
160
150
140
130
120
110
100
90
80
70
60
50
40
30
20
ppm

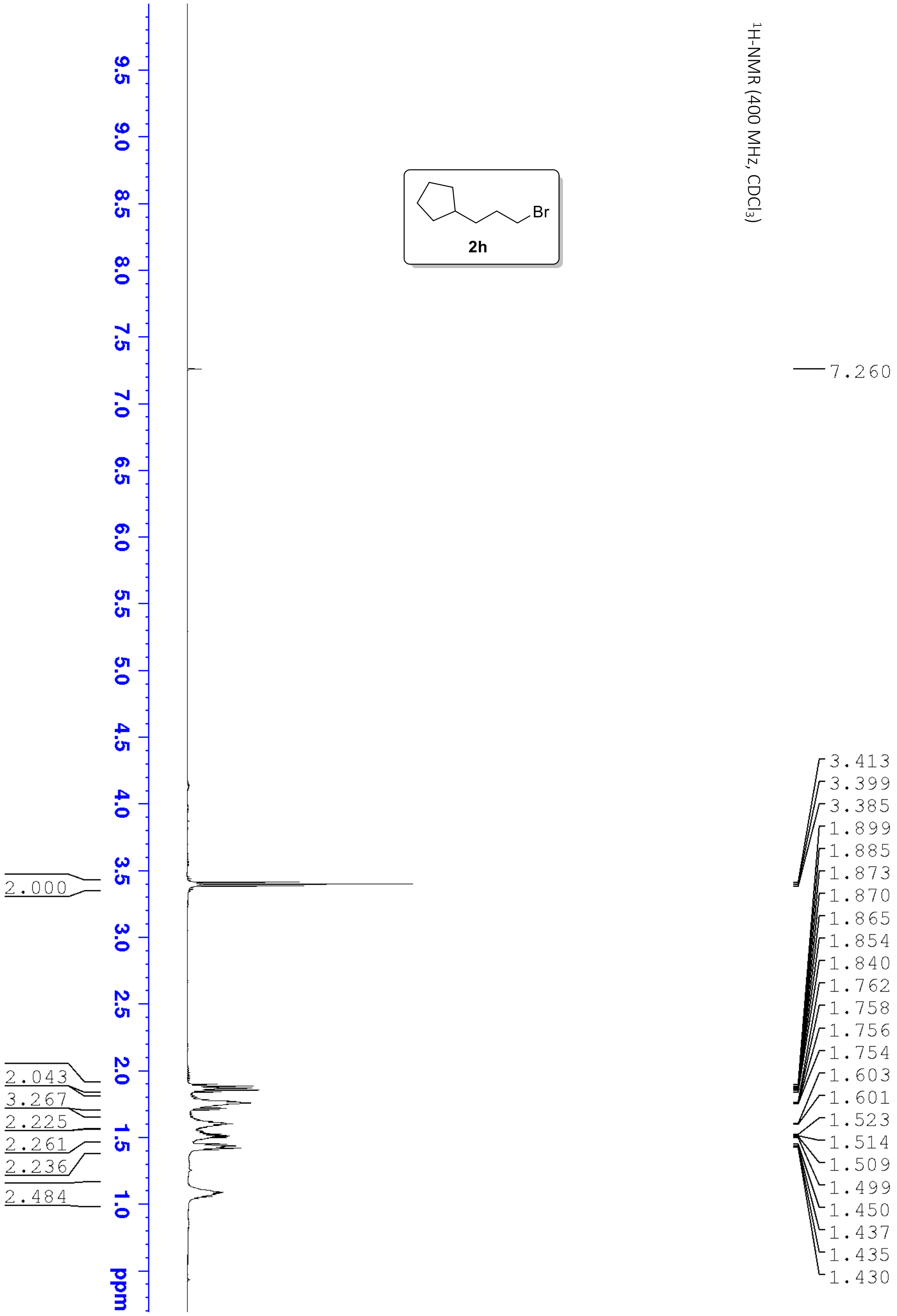

1H-NMR (400 MHz, CDCl3)
Br
2h
7.260
3.413
3.399
3.385
1.899
1.885
1.873
1.870
1.865
1.854
1.840
1.762
1.758
1.756
1.754
1.603
1.601
1.523
1.514
1.509
1.499
1.450
1.437
1.435
1.430
2.000
2.043
3.267
2.225
2.261
2.236
2.484
9.5
9.0
8.5
8.0
7.5
7.0
6.5
6.0
5.5
5.0
4.5
4.0
3.5
3.0
2.5
2.0
1.5
1.0
ppm

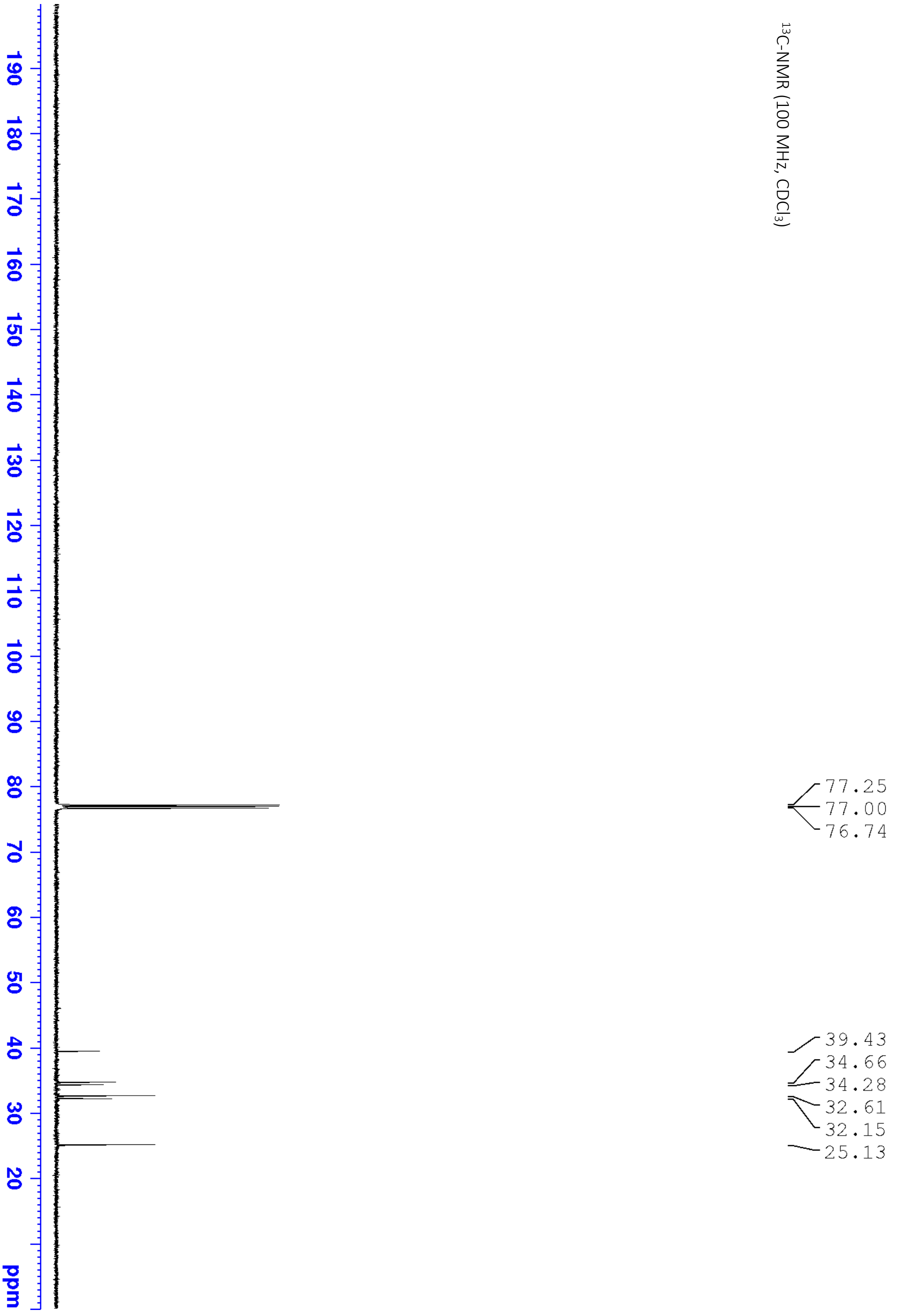
13C-NMR (100 MHz, CDCl3)
77.25
77.00
76.74
39.43
34.66
34.28
32.61
32.15
25.13
ppm

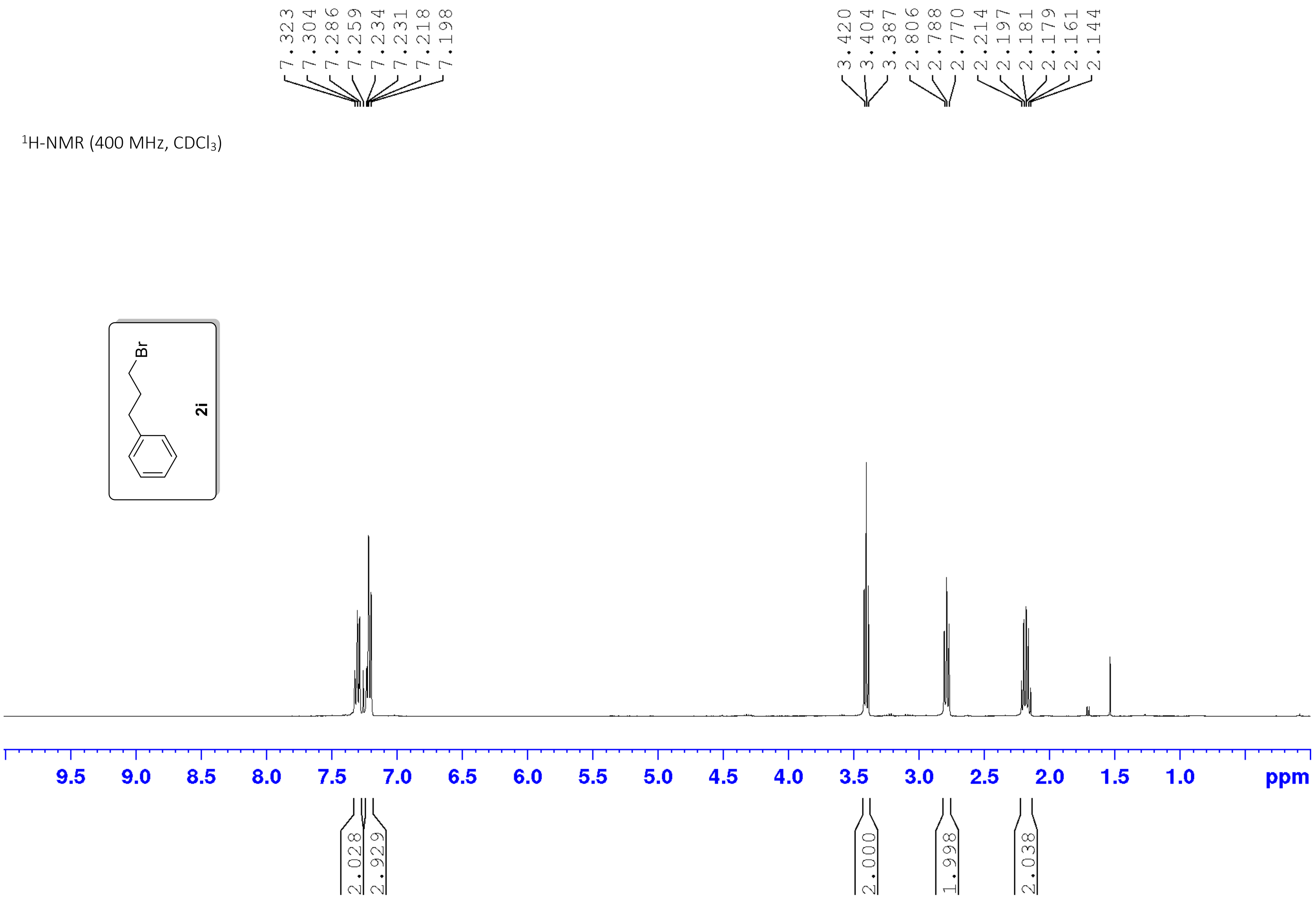

$^1$H-NMR (400 MHz, $CDCl_3$)
Br
2i
7.323
7.304
7.286
7.259
7.234
7.231
7.218
7.198
3.420
3.404
3.387
2.806
2.788
2.770
2.214
2.197
2.181
2.179
2.161
2.144
9.5
9.0
8.5
8.0
7.5
7.0
6.5
6.0
5.5
5.0
4.5
4.0
3.5
3.0
2.5
2.0
1.5
1.0
ppm
2.028
2.929
2.000
1.998
2.038

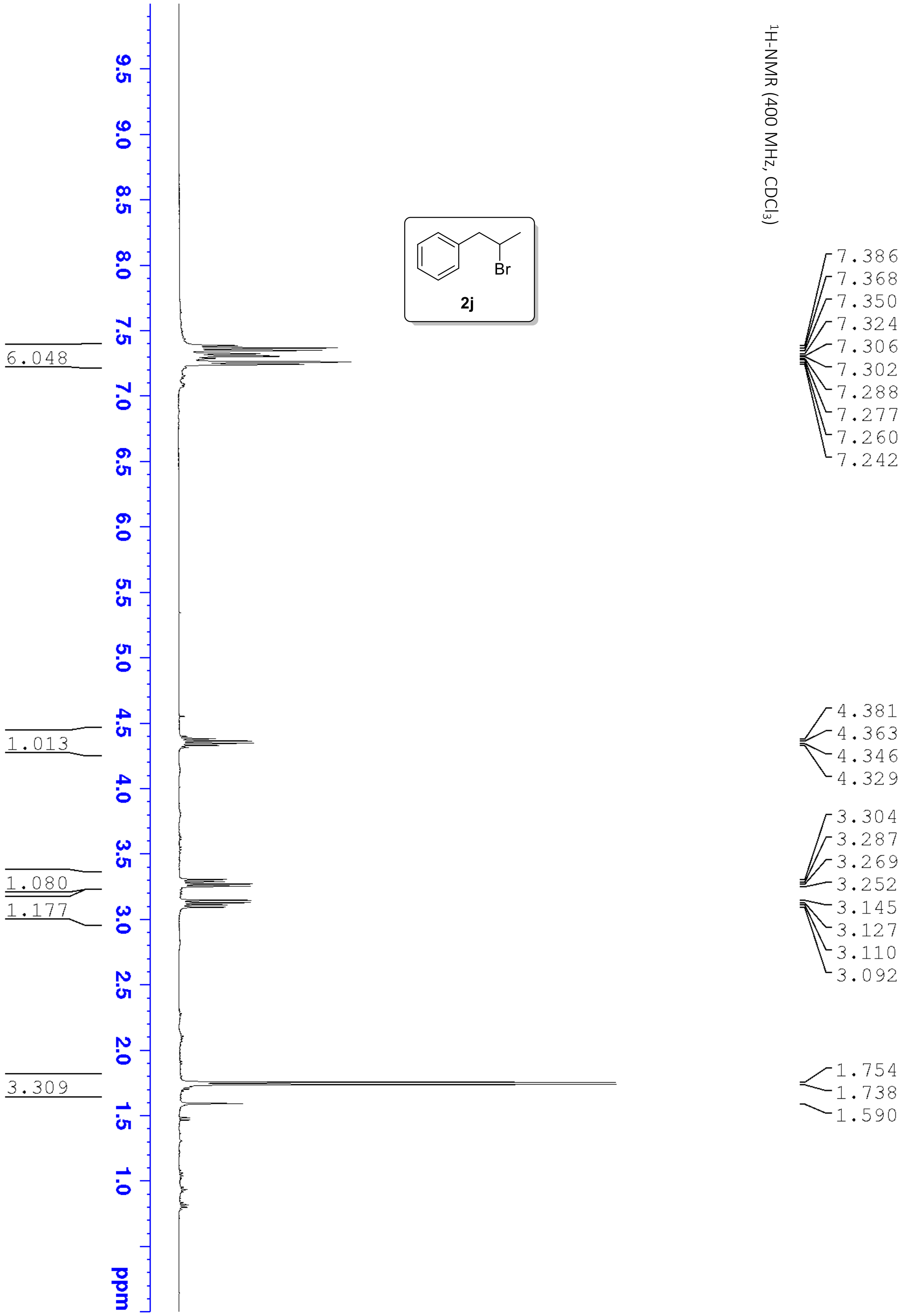
¹H-NMR (400 MHz, CDCl3)
Br
2j
7.386
7.368
7.350
7.324
7.306
7.302
7.288
7.277
7.260
7.242
4.381
4.363
4.346
4.329
3.304
3.287
3.269
3.252
3.145
3.127
3.110
3.092
1.754
1.738
1.590
6.048
1.013
1.080
1.177
3.309
9.5
9.0
8.5
8.0
7.5
7.0
6.5
6.0
5.5
5.0
4.5
4.0
3.5
3.0
2.5
2.0
1.5
1.0
ppm

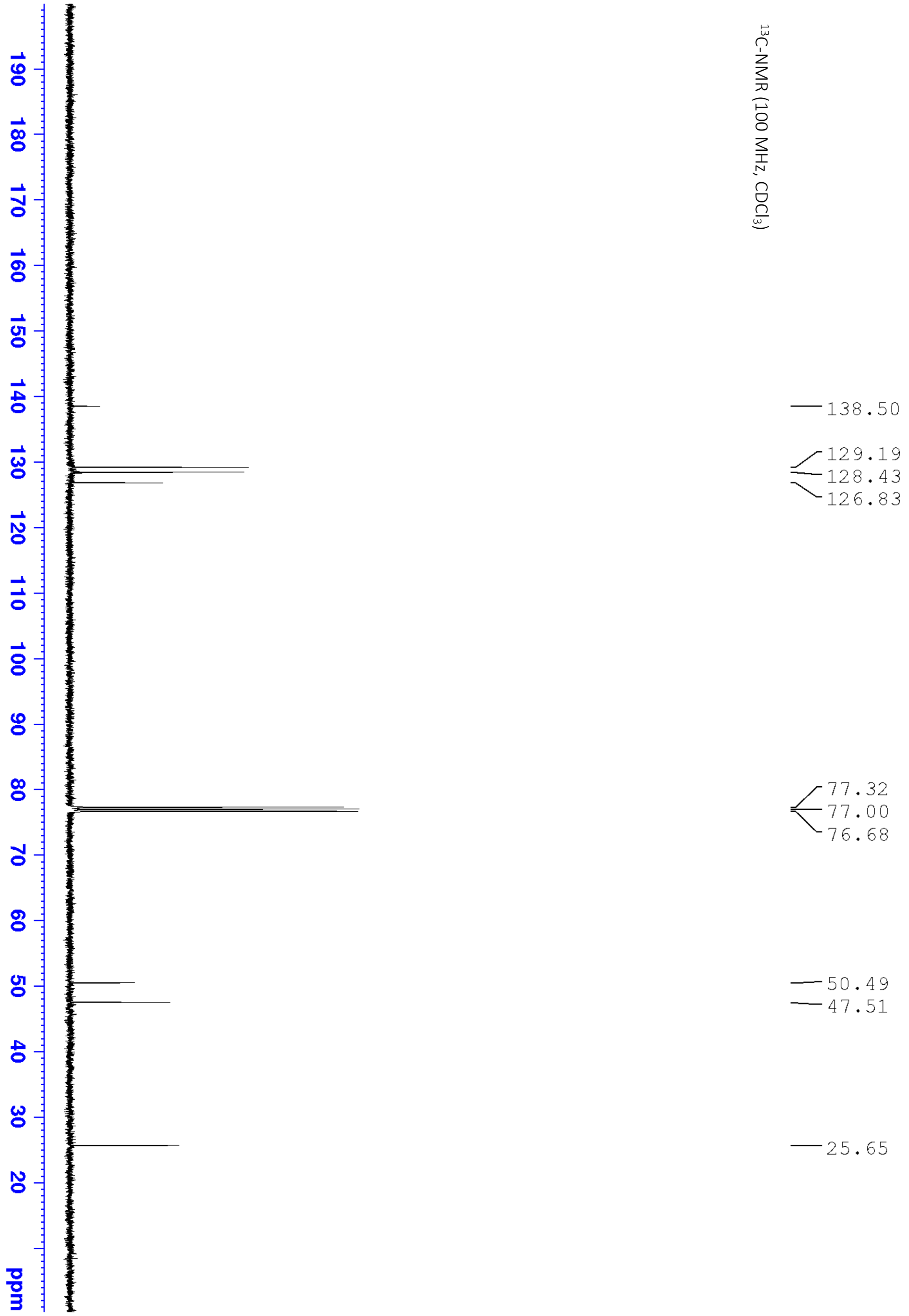
$^{13}$C-NMR (100 MHz, CDCl$_3$)
138.50
129.19
128.43
126.83
77.32
77.00
76.68
50.49
47.51
25.65
190
180
170
160
150
140
130
120
110
100
90
80
70
60
50
40
30
20
ppm

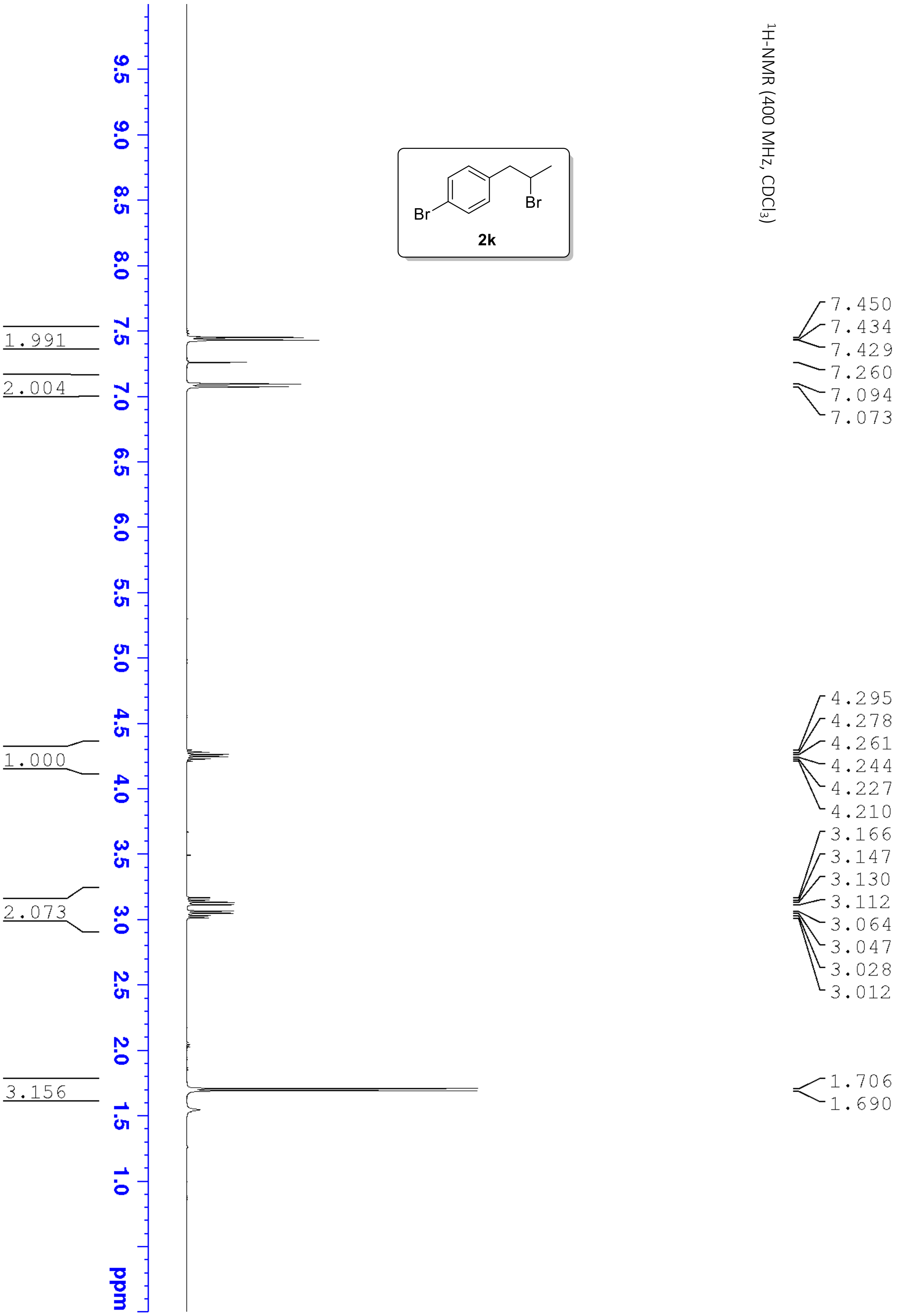

1H-NMR (400 MHz, CDCl3)
Br
Br
2k
7.450
7.434
7.429
7.260
7.094
7.073
4.295
4.278
4.261
4.244
4.227
4.210
3.166
3.147
3.130
3.112
3.064
3.047
3.028
3.012
1.706
1.690
1.991
2.004
1.000
2.073
3.156
9.5
9.0
8.5
8.0
7.5
7.0
6.5
6.0
5.5
5.0
4.5
4.0
3.5
3.0
2.5
2.0
1.5
1.0
ppm

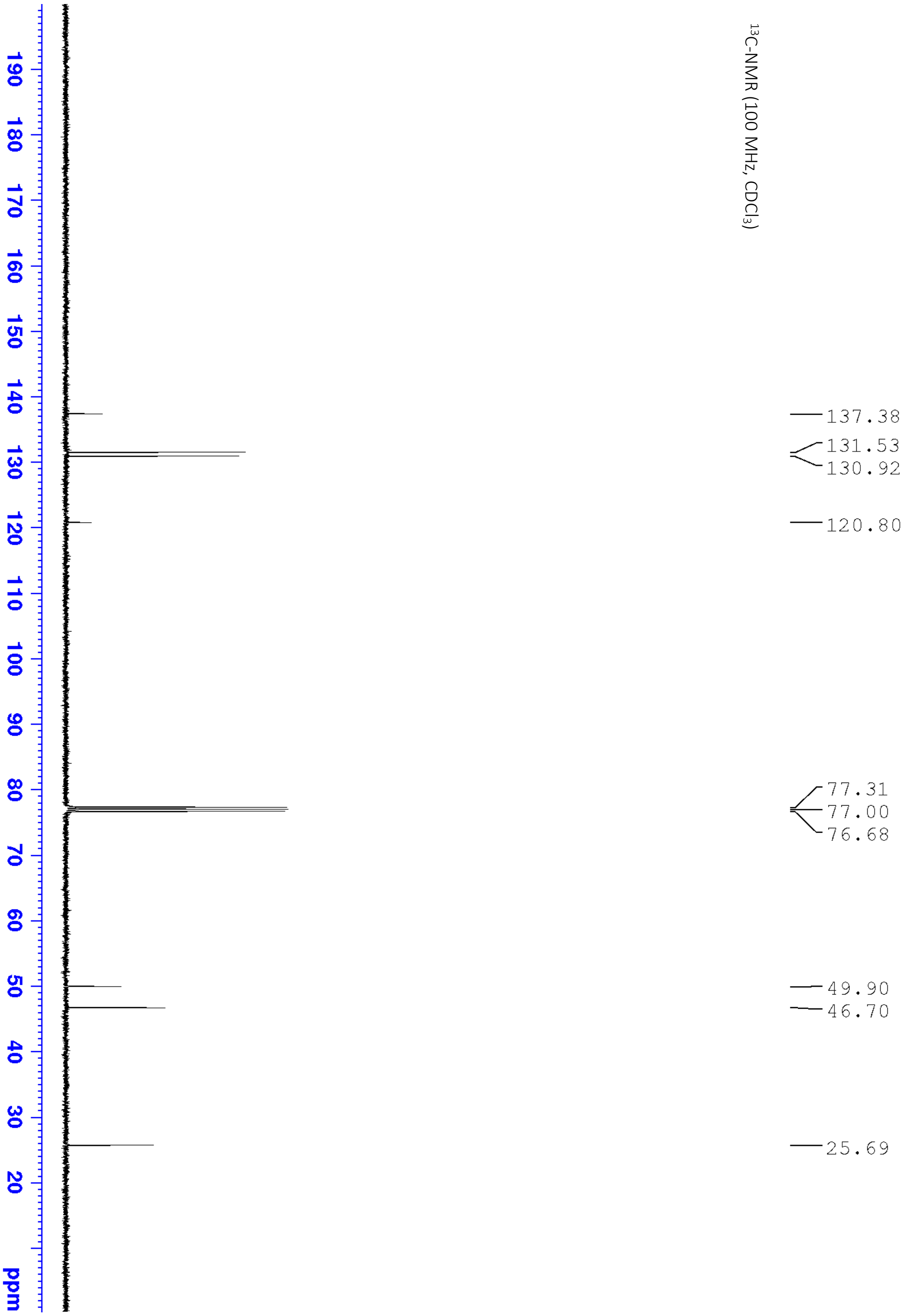

13C-NMR (100 MHz, CDCl3)
137.38
131.53
130.92
120.80
77.31
77.00
76.68
49.90
46.70
25.69
ppm

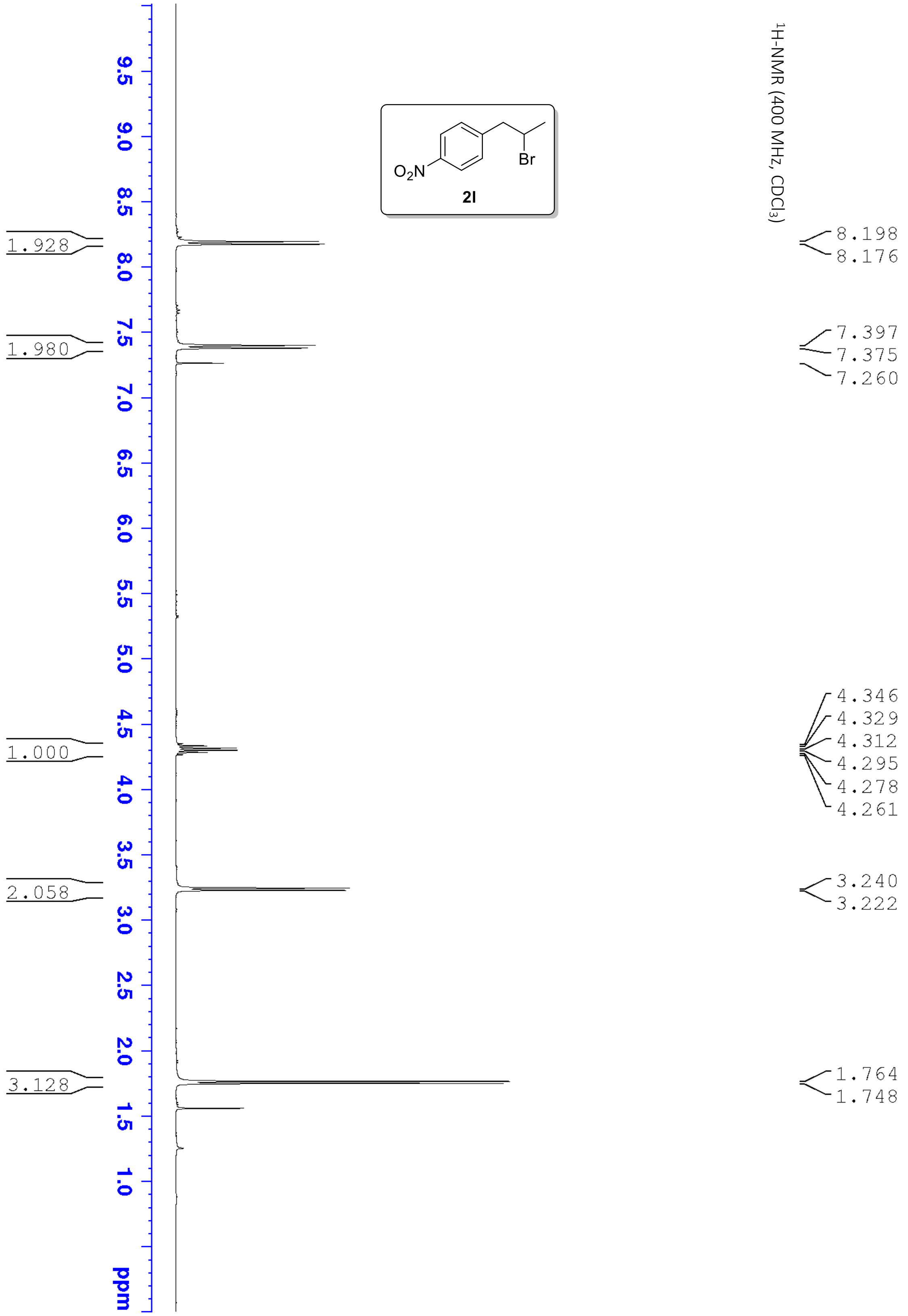
1H-NMR (400 MHz, CDCl3)
O2N
Br
2l
8.198
8.176
7.397
7.375
7.260
4.346
4.329
4.312
4.295
4.278
4.261
3.240
3.222
1.764
1.748
1.928
1.980
1.000
2.058
3.128
9.5
9.0
8.5
8.0
7.5
7.0
6.5
6.0
5.5
5.0
4.5
4.0
3.5
3.0
2.5
2.0
1.5
1.0
ppm

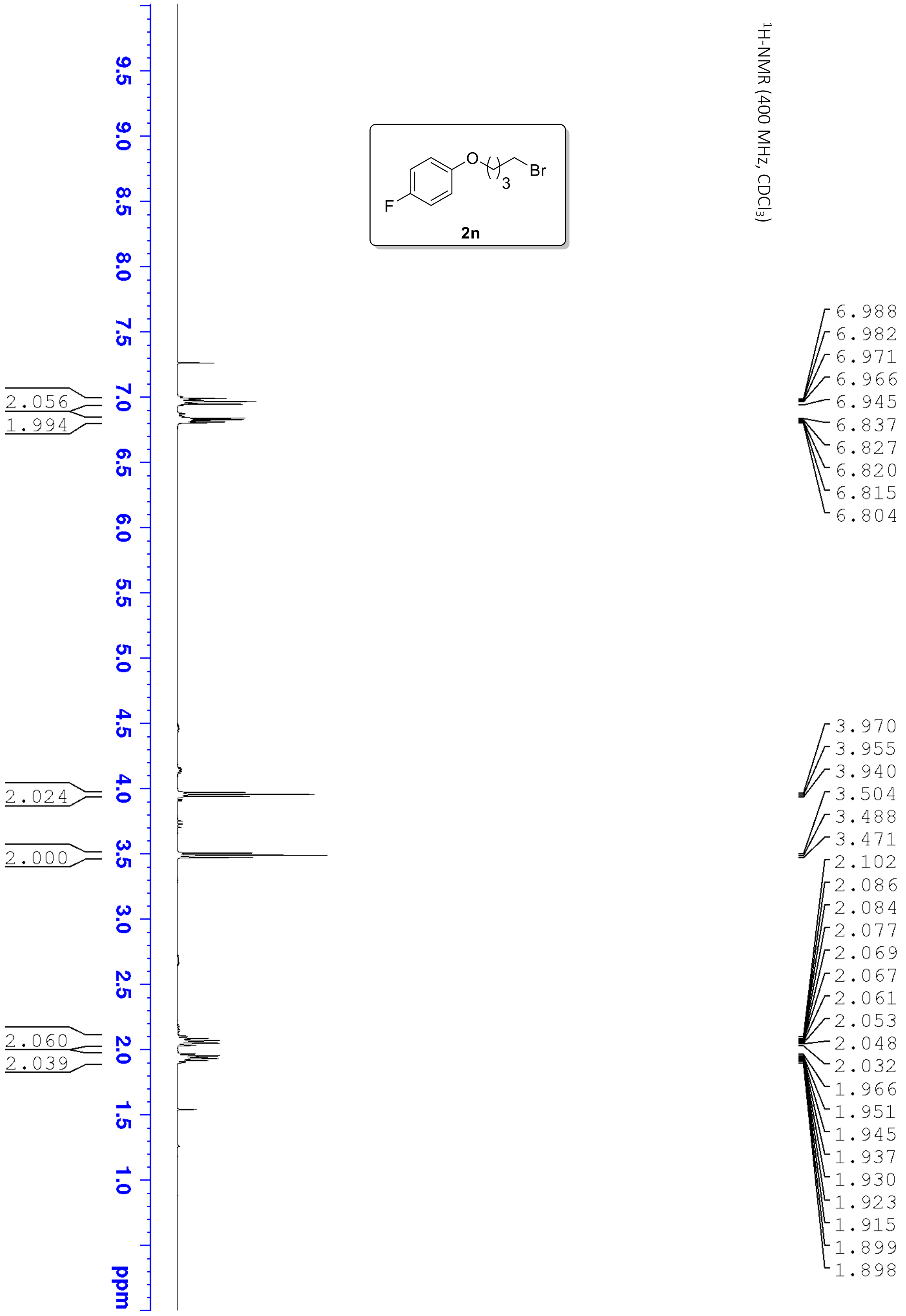

$^1$H-NMR (400 MHz, CDCl$_3$)
F
O
3
Br
2n
6.988
6.982
6.971
6.966
6.945
6.837
6.827
6.820
6.815
6.804
3.970
3.955
3.940
3.504
3.488
3.471
2.102
2.086
2.084
2.077
2.069
2.067
2.061
2.053
2.048
2.032
1.966
1.951
1.945
1.937
1.930
1.923
1.915
1.899
1.898
2.056
1.994
2.024
2.000
2.060
2.039
9.5
9.0
8.5
8.0
7.5
7.0
6.5
6.0
5.5
5.0
4.5
4.0
3.5
3.0
2.5
2.0
1.5
1.0
ppm

$^{1}H$-NMR (400 MHz, $CDCl_3$)

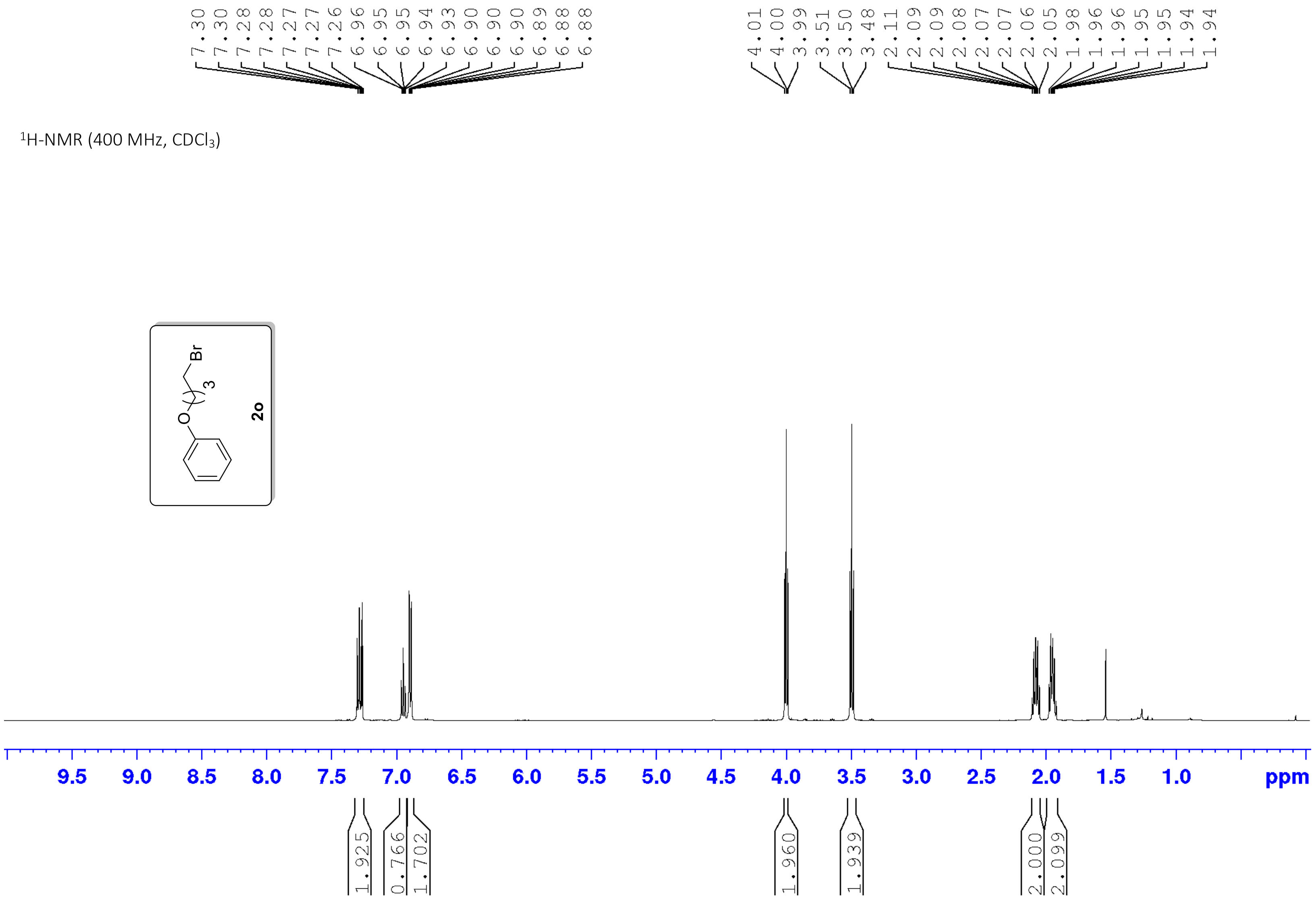

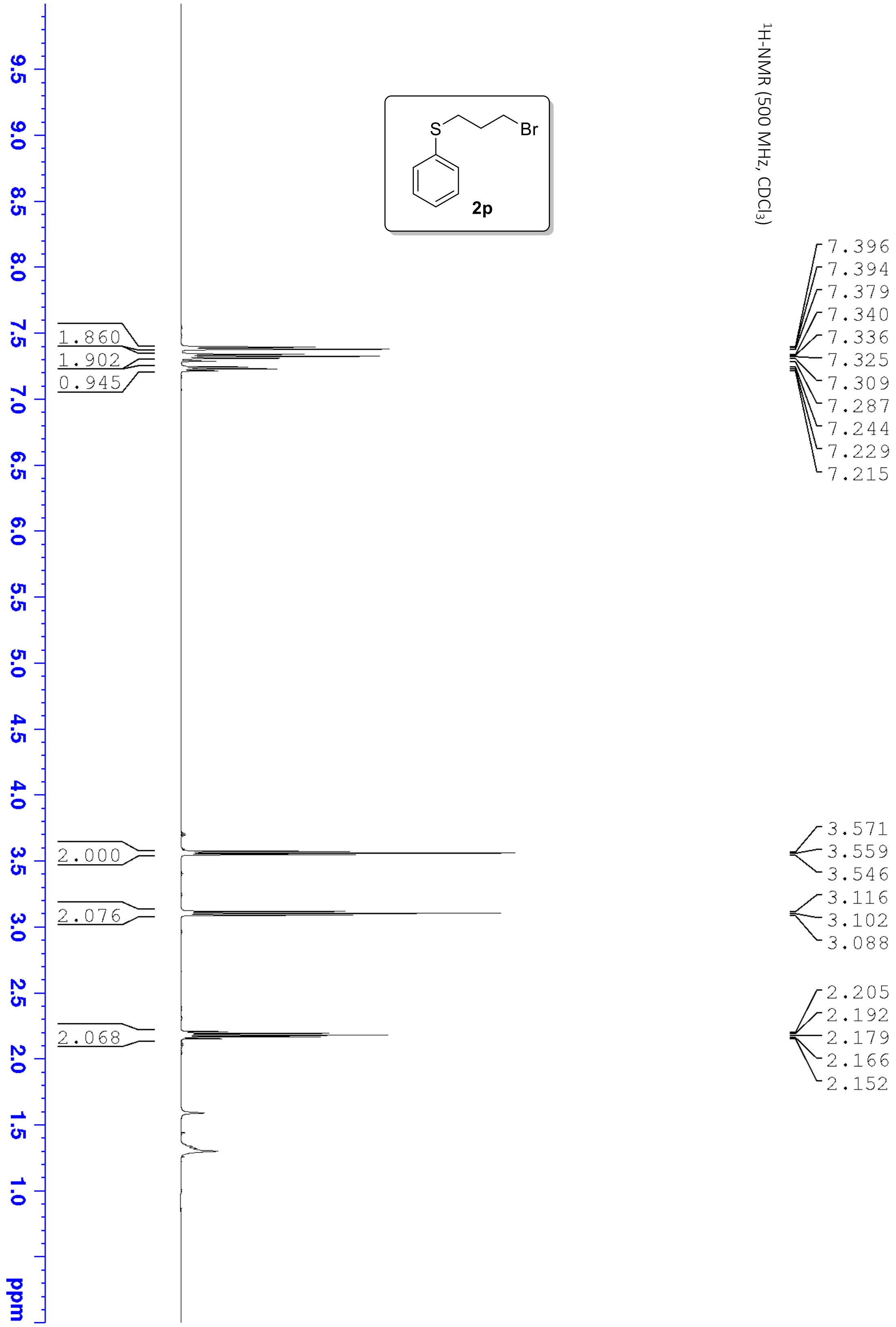

1H-NMR (500 MHz, CDCl3)
S
Br
2p
7.396
7.394
7.379
7.340
7.336
7.325
7.309
7.287
7.244
7.229
7.215
3.571
3.559
3.546
3.116
3.102
3.088
2.205
2.192
2.179
2.166
2.152
1.860
1.902
0.945
2.000
2.076
2.068
9.5
9.0
8.5
8.0
7.5
7.0
6.5
6.0
5.5
5.0
4.5
4.0
3.5
3.0
2.5
2.0
1.5
1.0
ppm

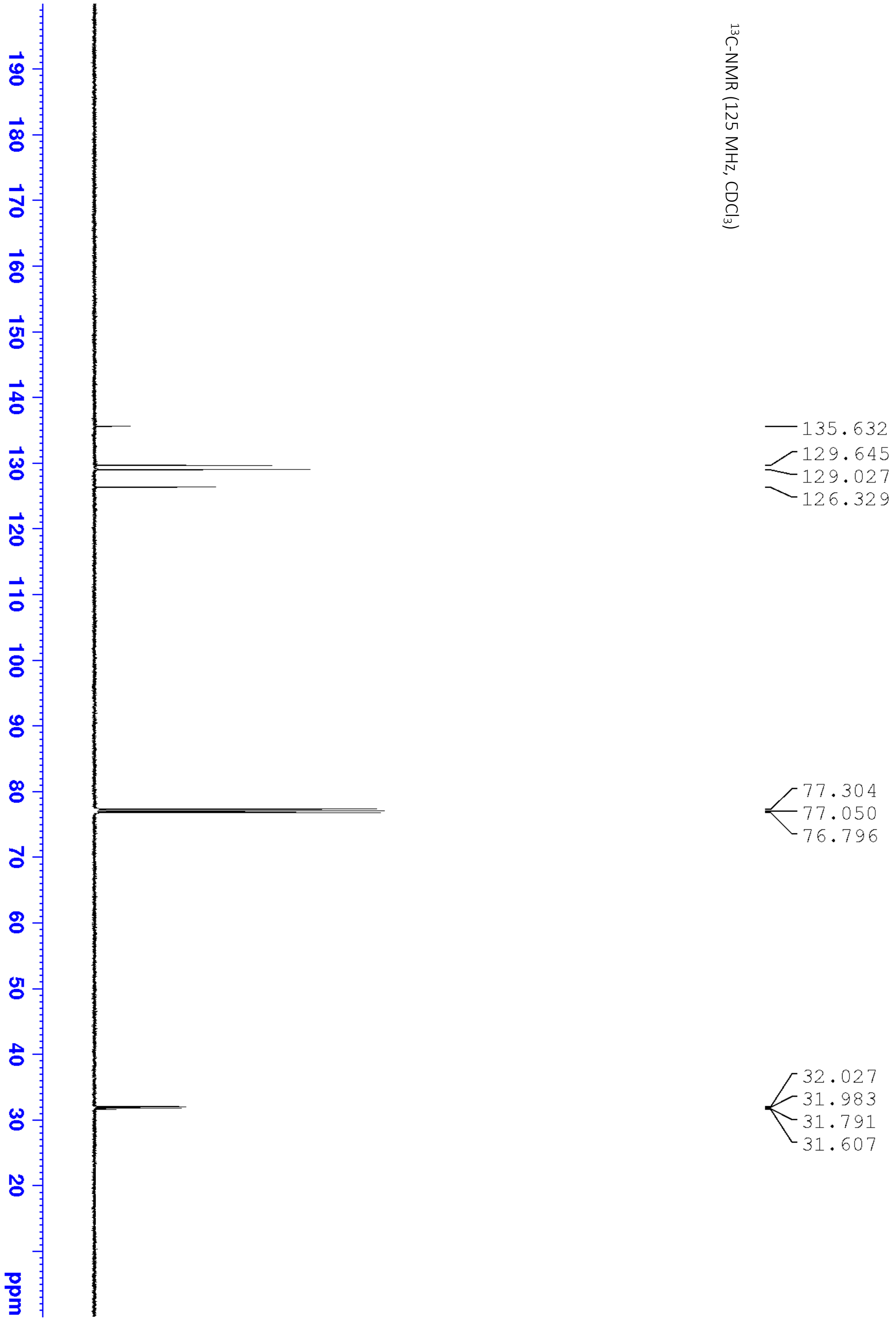

13C-NMR (125 MHz, CDCl3)
135.632
129.645
129.027
126.329
77.304
77.050
76.796
32.027
31.983
31.791
31.607
ppm

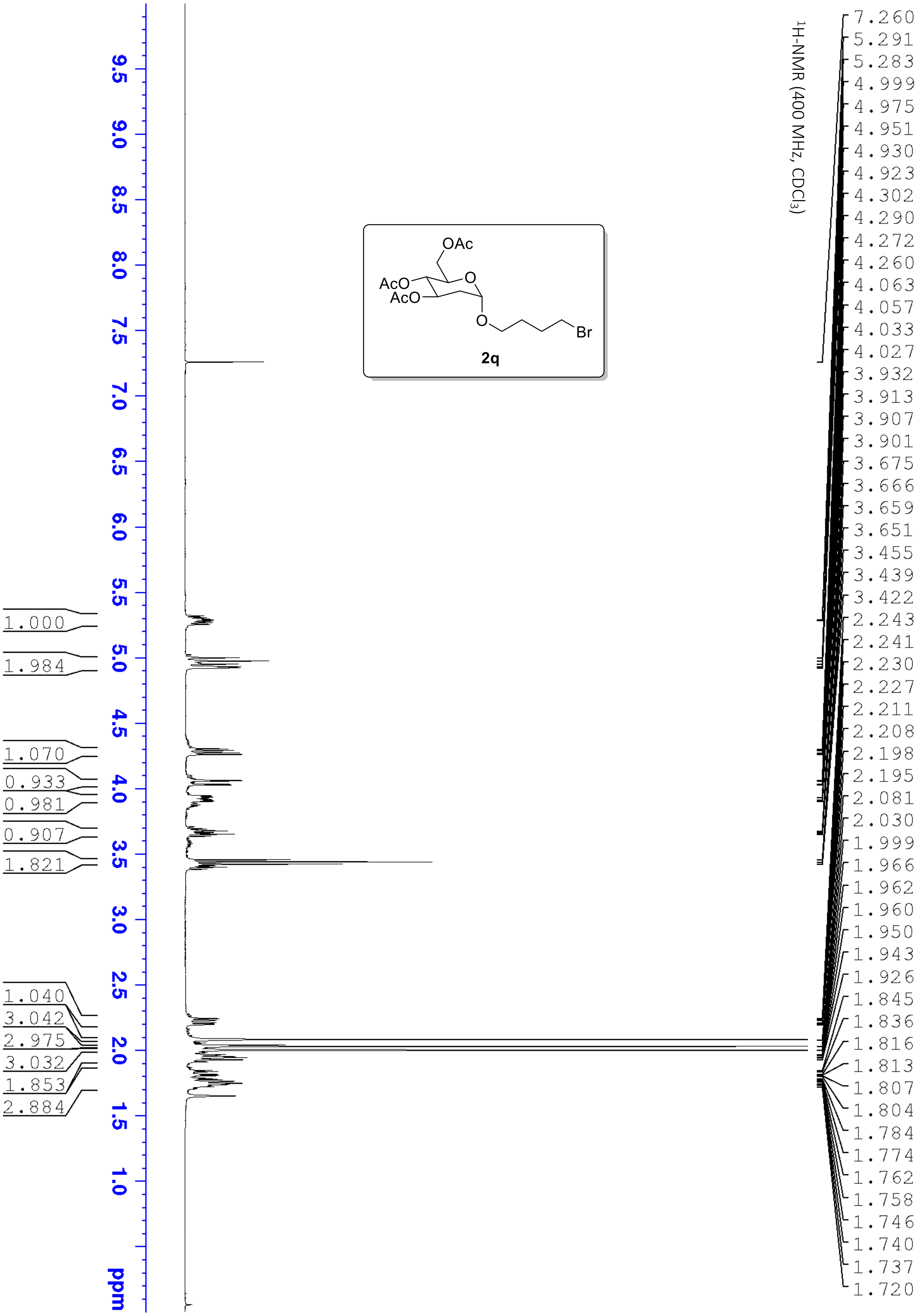

1H-NMR (400 MHz, CDCl3)
OAc
AcO
AcO
O
O
Br
2q
7.260
5.291
5.283
4.999
4.975
4.951
4.930
4.923
4.302
4.290
4.272
4.260
4.063
4.057
4.033
4.027
3.932
3.913
3.907
3.901
3.675
3.666
3.659
3.651
3.455
3.439
3.422
2.243
2.241
2.230
2.227
2.211
2.208
2.198
2.195
2.081
2.030
1.999
1.966
1.962
1.960
1.950
1.943
1.926
1.845
1.836
1.816
1.813
1.807
1.804
1.784
1.774
1.762
1.758
1.746
1.740
1.737
1.720
1.000
1.984
1.070
0.933
0.981
0.907
1.821
1.040
3.042
2.975
3.032
1.853
2.884
9.5
9.0
8.5
8.0
7.5
7.0
6.5
6.0
5.5
5.0
4.5
4.0
3.5
3.0
2.5
2.0
1.5
1.0
ppm

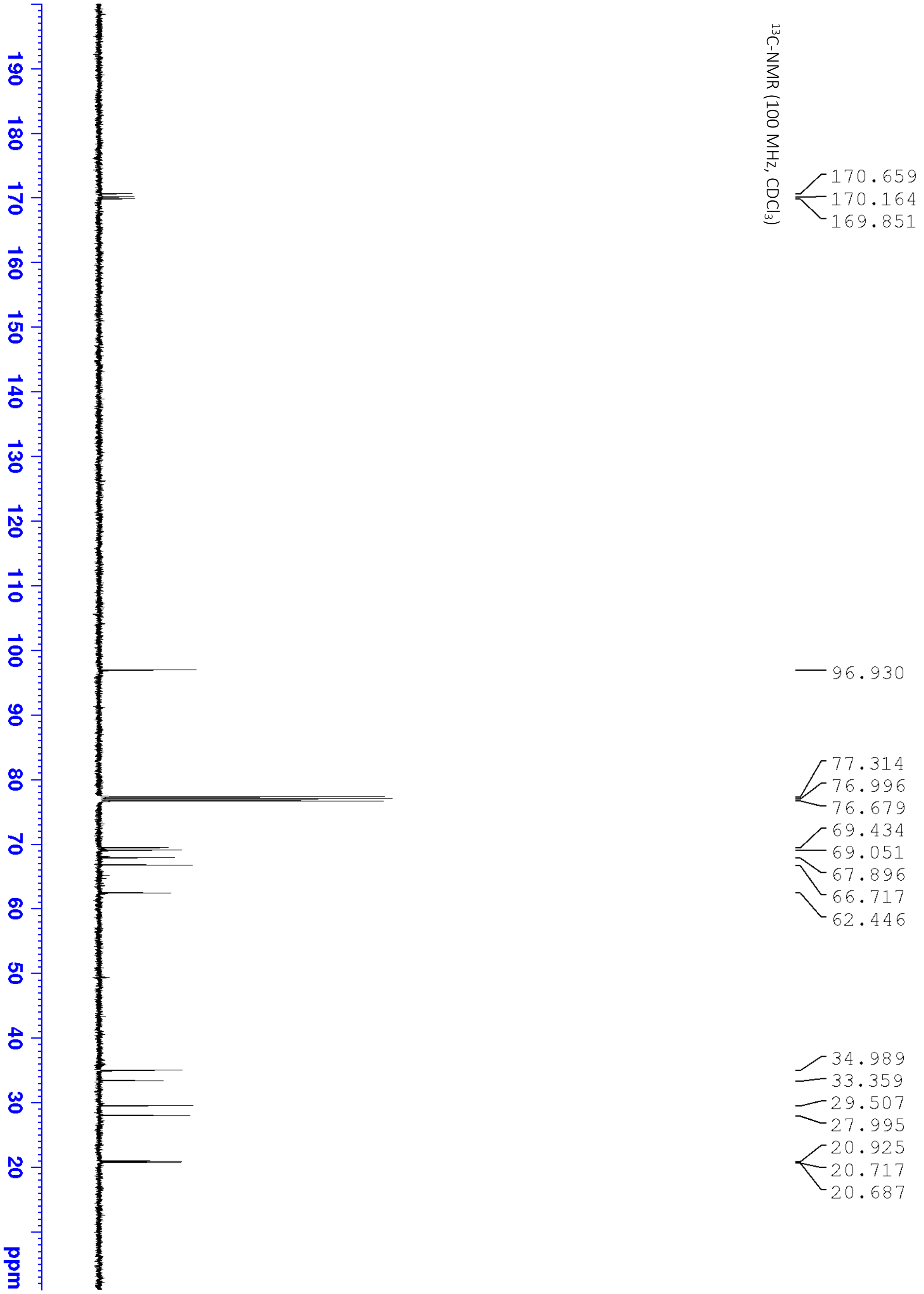

13C-NMR (100 MHz, CDCl3)
170.659
170.164
169.851
96.930
77.314
76.996
76.679
69.434
69.051
67.896
66.717
62.446
34.989
33.359
29.507
27.995
20.925
20.717
20.687
190
180
170
160
150
140
130
120
110
100
90
80
70
60
50
40
30
20
ppm

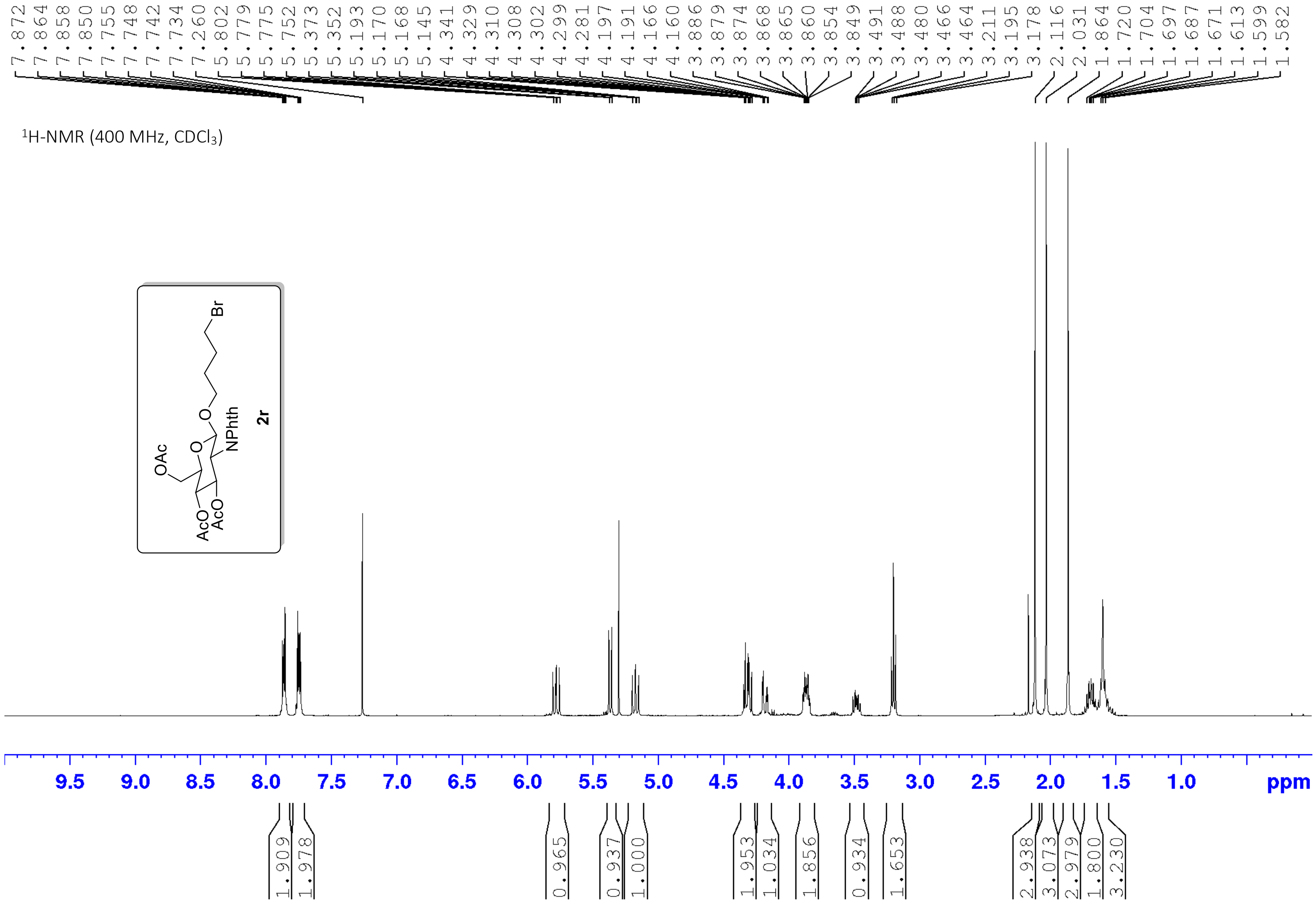
1H-NMR (400 MHz, CDCl3)
OAc
AcO
AcO
O
O
NPhth
Br
2r
7.872 7.864 7.858 7.850 7.755 7.748 7.742 7.734 7.260 5.802 5.779 5.775 5.752 5.373 5.352 5.193 5.170 5.168 5.145 4.341 4.329 4.310 4.308 4.302 4.299 4.281 4.197 4.191 4.166 4.160 3.886 3.879 3.874 3.868 3.865 3.860 3.854 3.849 3.491 3.488 3.480 3.466 3.464 3.211 3.195 3.178 2.116 2.031 1.864 1.720 1.704 1.697 1.687 1.671 1.613 1.599 1.582
9.5 9.0 8.5 8.0 7.5 7.0 6.5 6.0 5.5 5.0 4.5 4.0 3.5 3.0 2.5 2.0 1.5 1.0 ppm
1.909 1.978 0.965 0.937 1.000 1.953 1.034 1.856 0.934 1.653 2.938 3.073 2.979 1.800 3.230

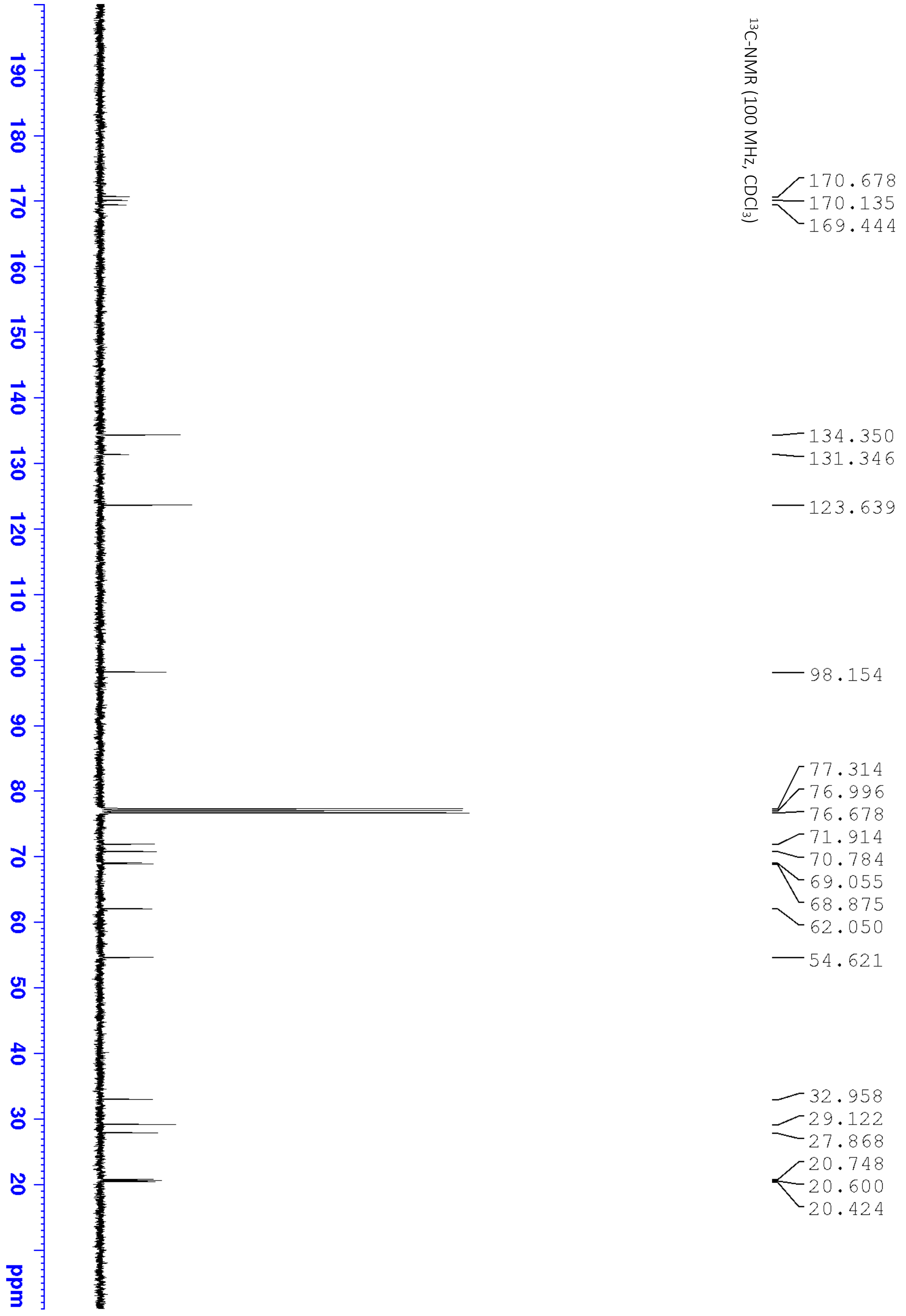

13C-NMR (100 MHz, CDCl3)
170.678
170.135
169.444
134.350
131.346
123.639
98.154
77.314
76.996
76.678
71.914
70.784
69.055
68.875
62.050
54.621
32.958
29.122
27.868
20.748
20.600
20.424
ppm

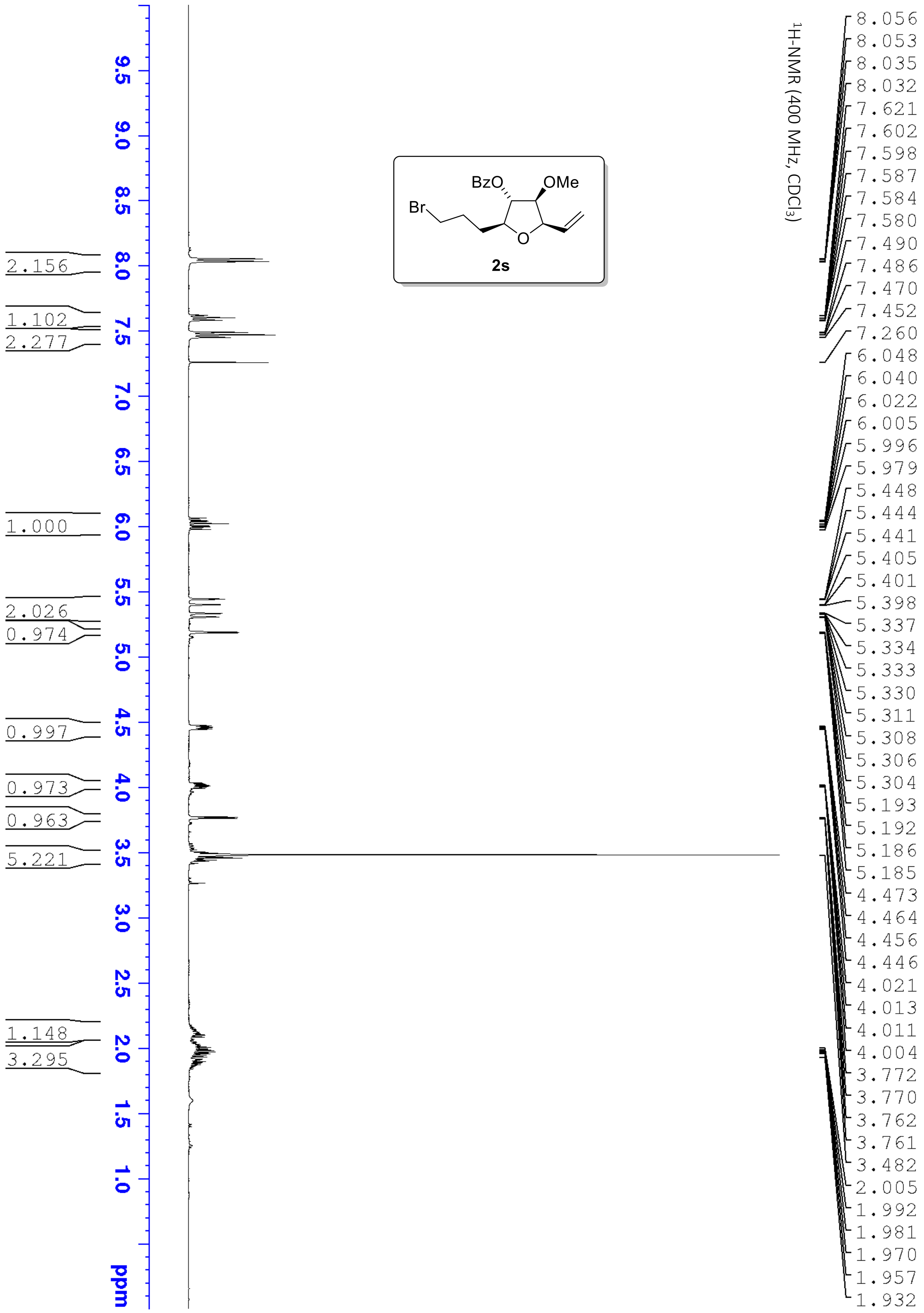

1H-NMR (400 MHz, CDCl3)
BzO
OMe
Br
O
2s
8.056
8.053
8.035
8.032
7.621
7.602
7.598
7.587
7.584
7.580
7.490
7.486
7.470
7.452
7.260
6.048
6.040
6.022
6.005
5.996
5.979
5.448
5.444
5.441
5.405
5.401
5.398
5.337
5.334
5.333
5.330
5.311
5.308
5.306
5.304
5.193
5.192
5.186
5.185
4.473
4.464
4.456
4.446
4.021
4.013
4.011
4.004
3.772
3.770
3.762
3.761
3.482
2.005
1.992
1.981
1.970
1.957
1.932
2.156
1.102
2.277
1.000
2.026
0.974
0.997
0.973
0.963
5.221
1.148
3.295
9.5
9.0
8.5
8.0
7.5
7.0
6.5
6.0
5.5
5.0
4.5
4.0
3.5
3.0
2.5
2.0
1.5
1.0
ppm

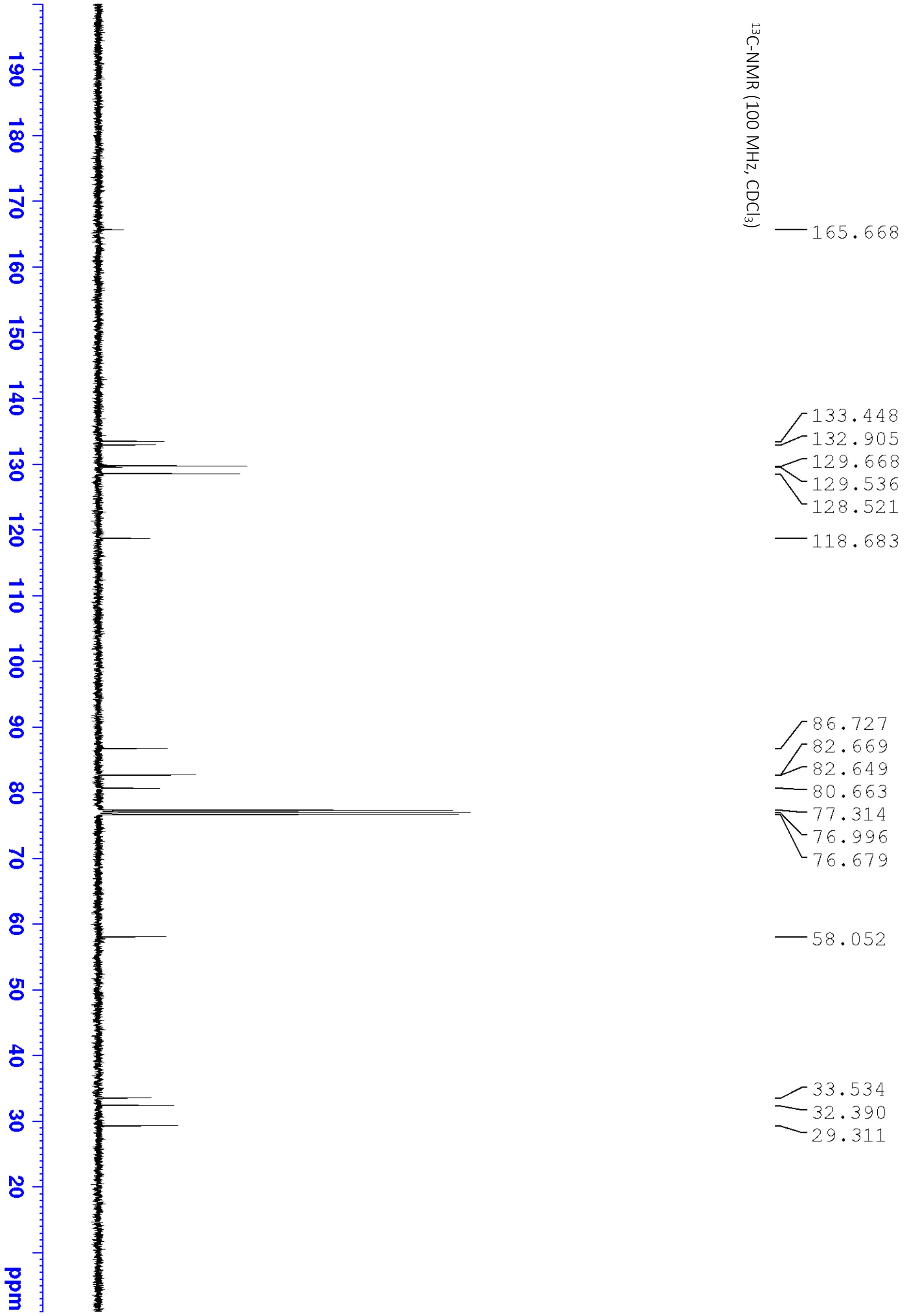
$^{13}$C-NMR (100 MHz, CDCl$_3$)
165.668
133.448
132.905
129.668
129.536
128.521
118.683
86.727
82.669
82.649
80.663
77.314
76.996
76.679
58.052
33.534
32.390
29.311
ppm
190
180
170
160
150
140
130
120
110
100
90
80
70
60
50
40
30
20

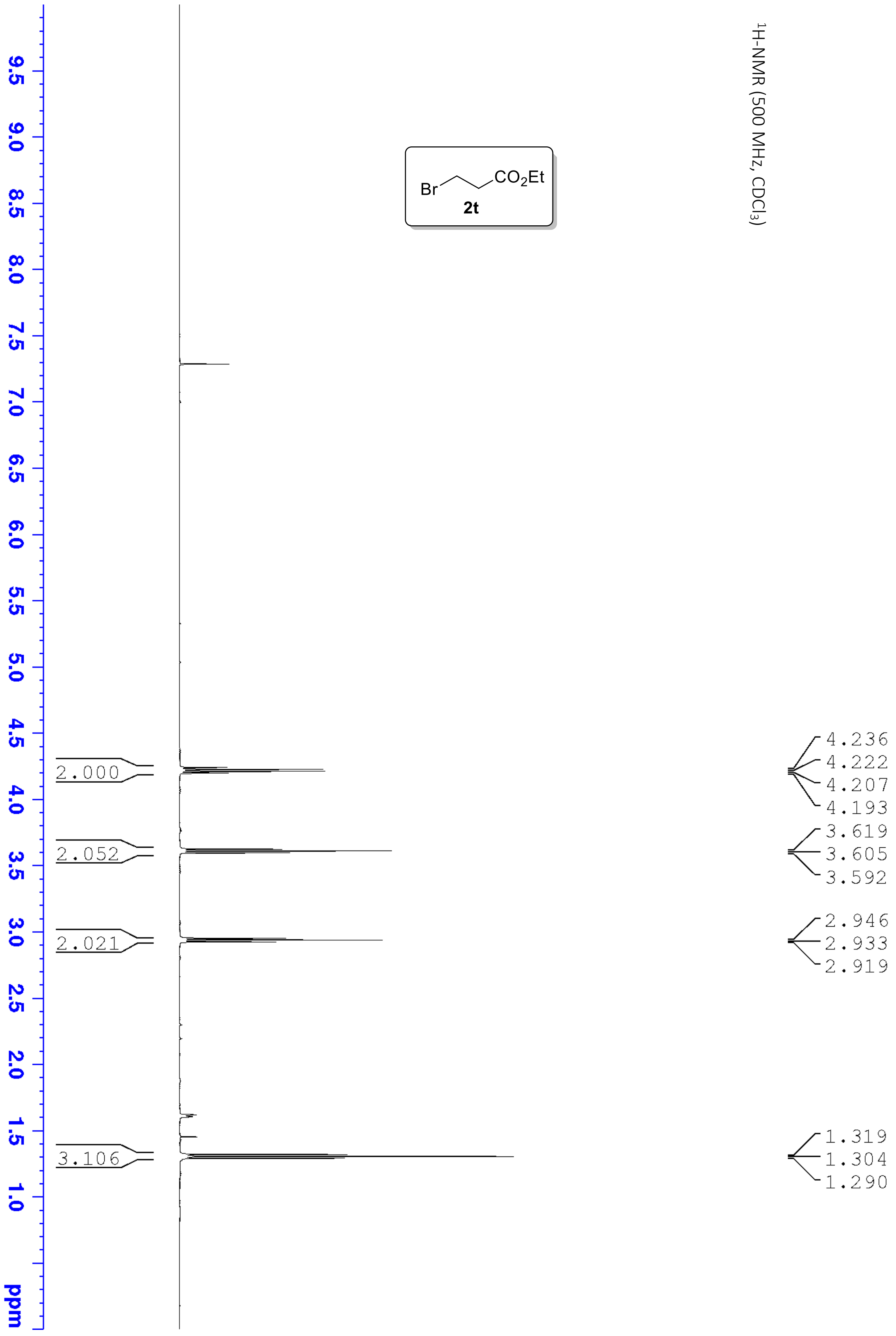
1H-NMR (500 MHz, CDCl3)
Br
CO2Et
2t
4.236
4.222
4.207
4.193
3.619
3.605
3.592
2.946
2.933
2.919
1.319
1.304
1.290
2.000
2.052
2.021
3.106
9.5
9.0
8.5
8.0
7.5
7.0
6.5
6.0
5.5
5.0
4.5
4.0
3.5
3.0
2.5
2.0
1.5
1.0
ppm

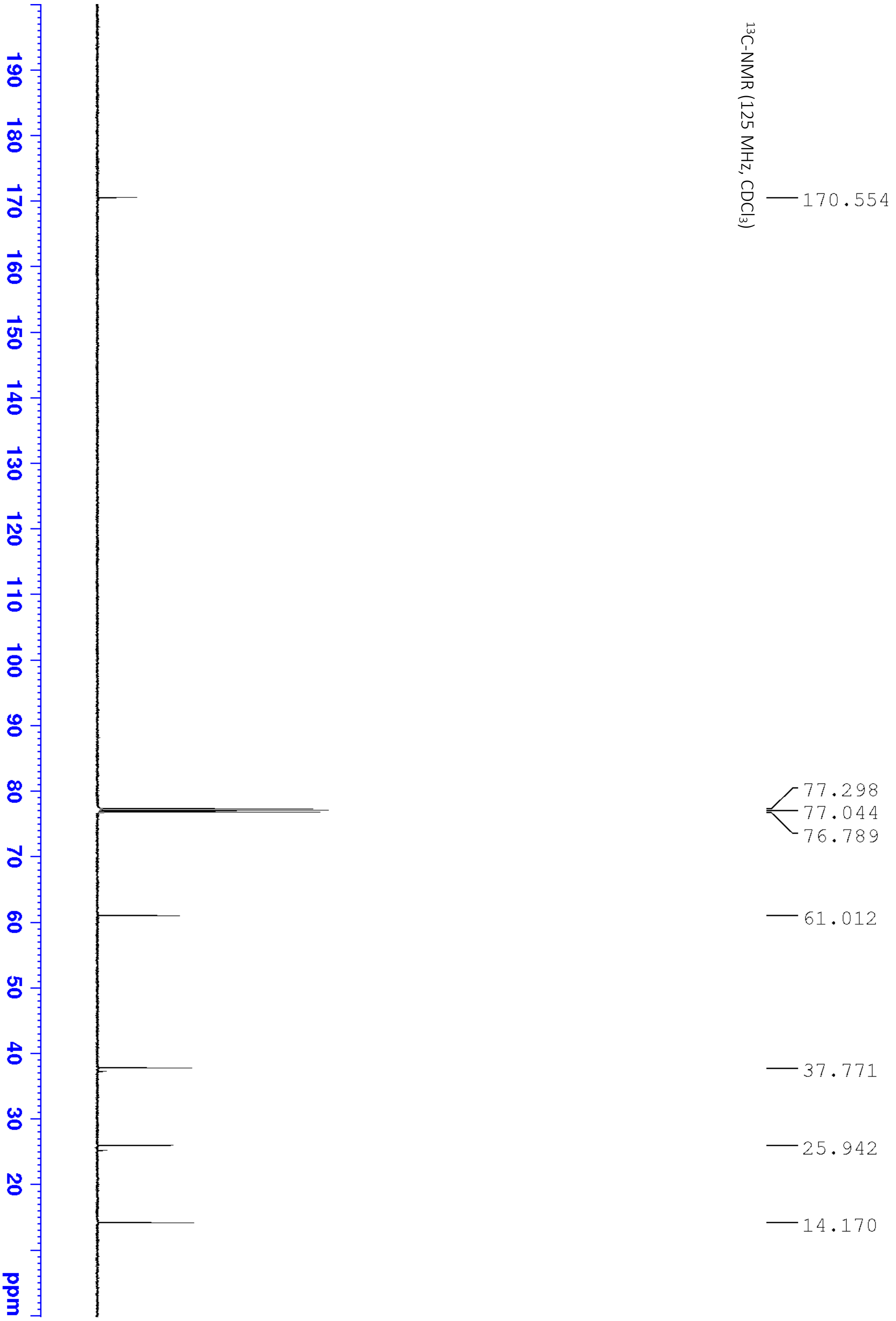

13C-NMR (125 MHz, CDCl3)
170.554
77.298
77.044
76.789
61.012
37.771
25.942
14.170
190
180
170
160
150
140
130
120
110
100
90
80
70
60
50
40
30
20
ppm

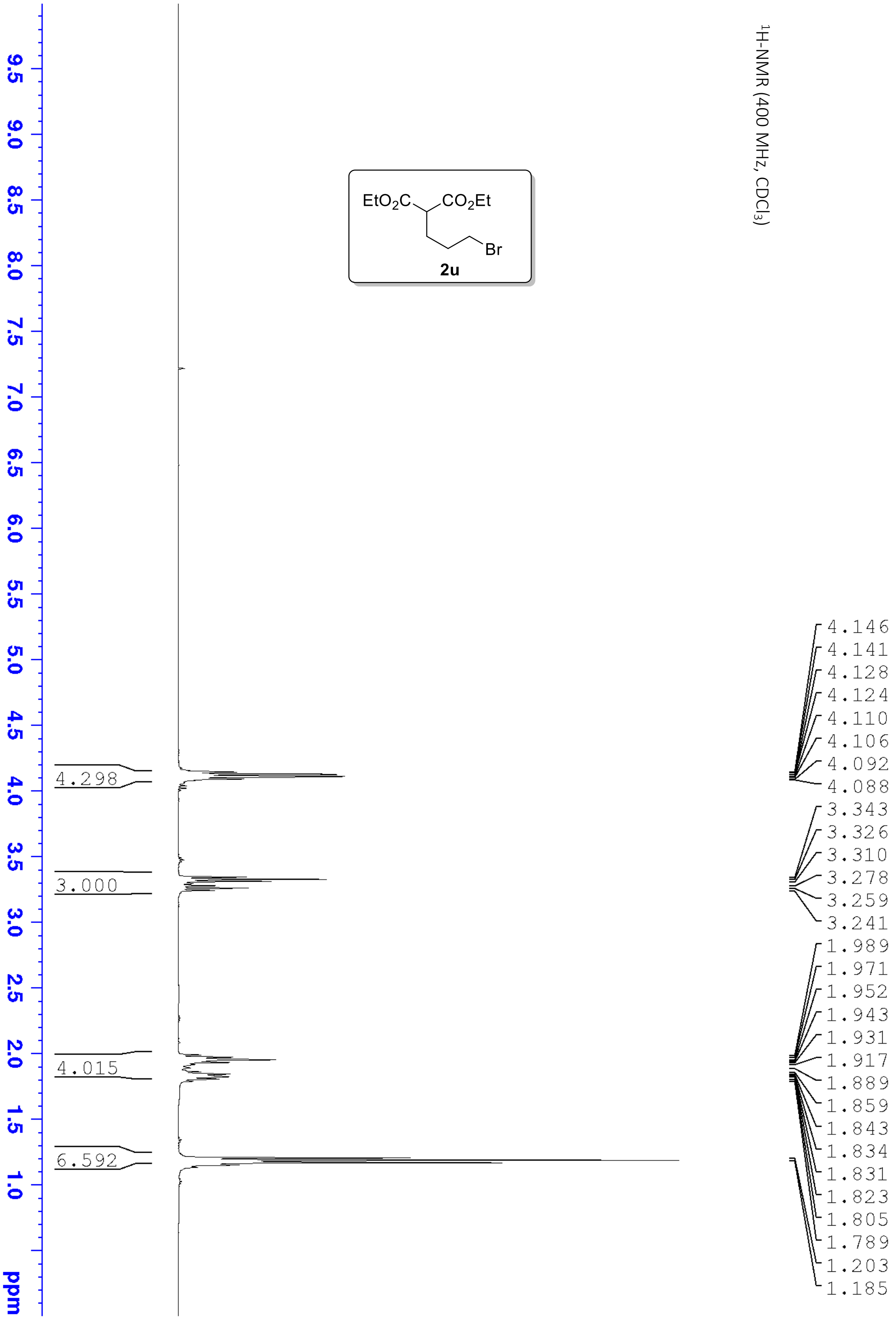

1H-NMR (400 MHz, CDCl3)
EtO2C
CO2Et
Br
2u
4.146
4.141
4.128
4.124
4.110
4.106
4.092
4.088
3.343
3.326
3.310
3.278
3.259
3.241
1.989
1.971
1.952
1.943
1.931
1.917
1.889
1.859
1.843
1.834
1.831
1.823
1.805
1.789
1.203
1.185
4.298
3.000
4.015
6.592
9.5
9.0
8.5
8.0
7.5
7.0
6.5
6.0
5.5
5.0
4.5
4.0
3.5
3.0
2.5
2.0
1.5
1.0
ppm

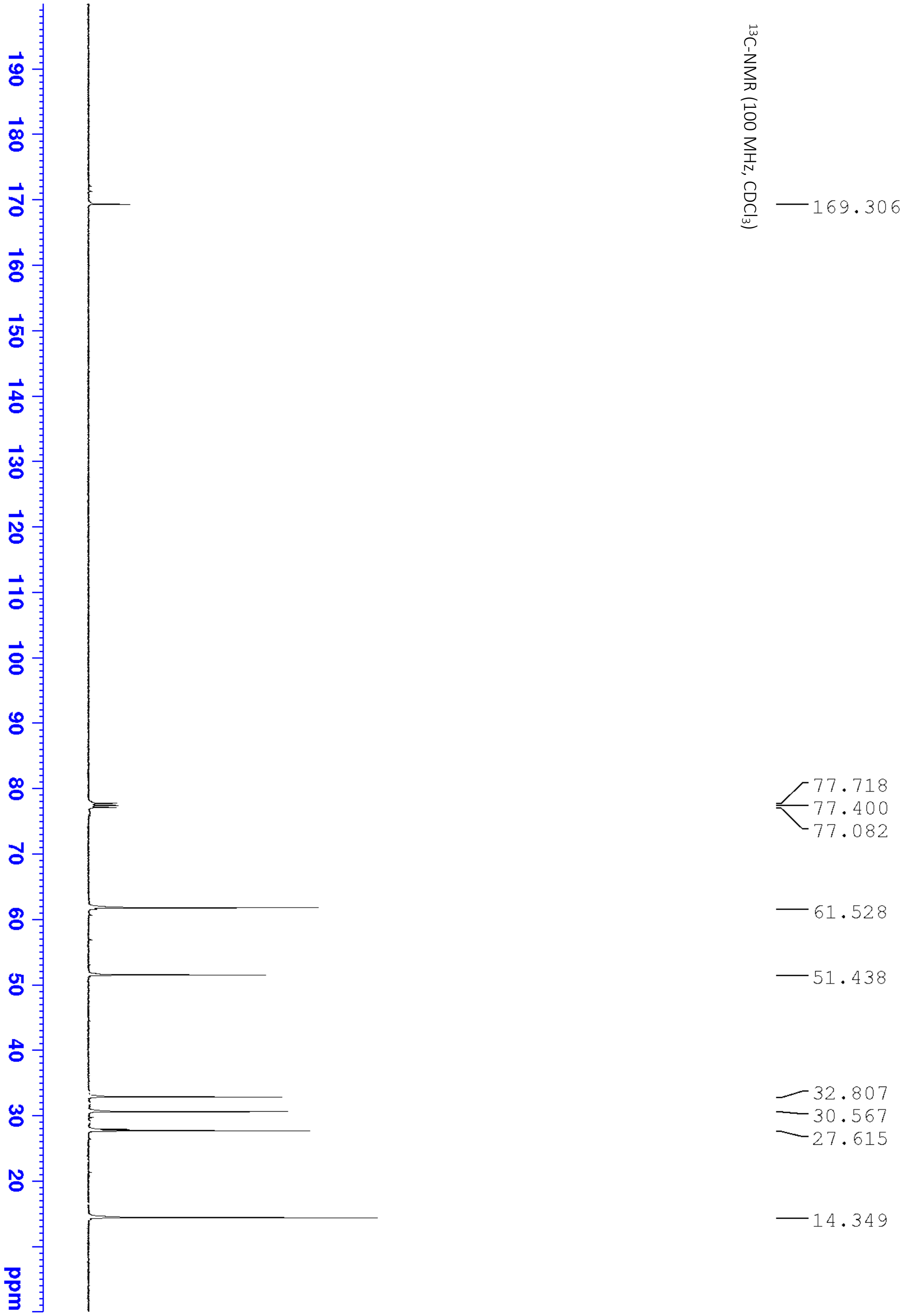

13C-NMR (100 MHz, CDCl3)
169.306
77.718
77.400
77.082
61.528
51.438
32.807
30.567
27.615
14.349
ppm

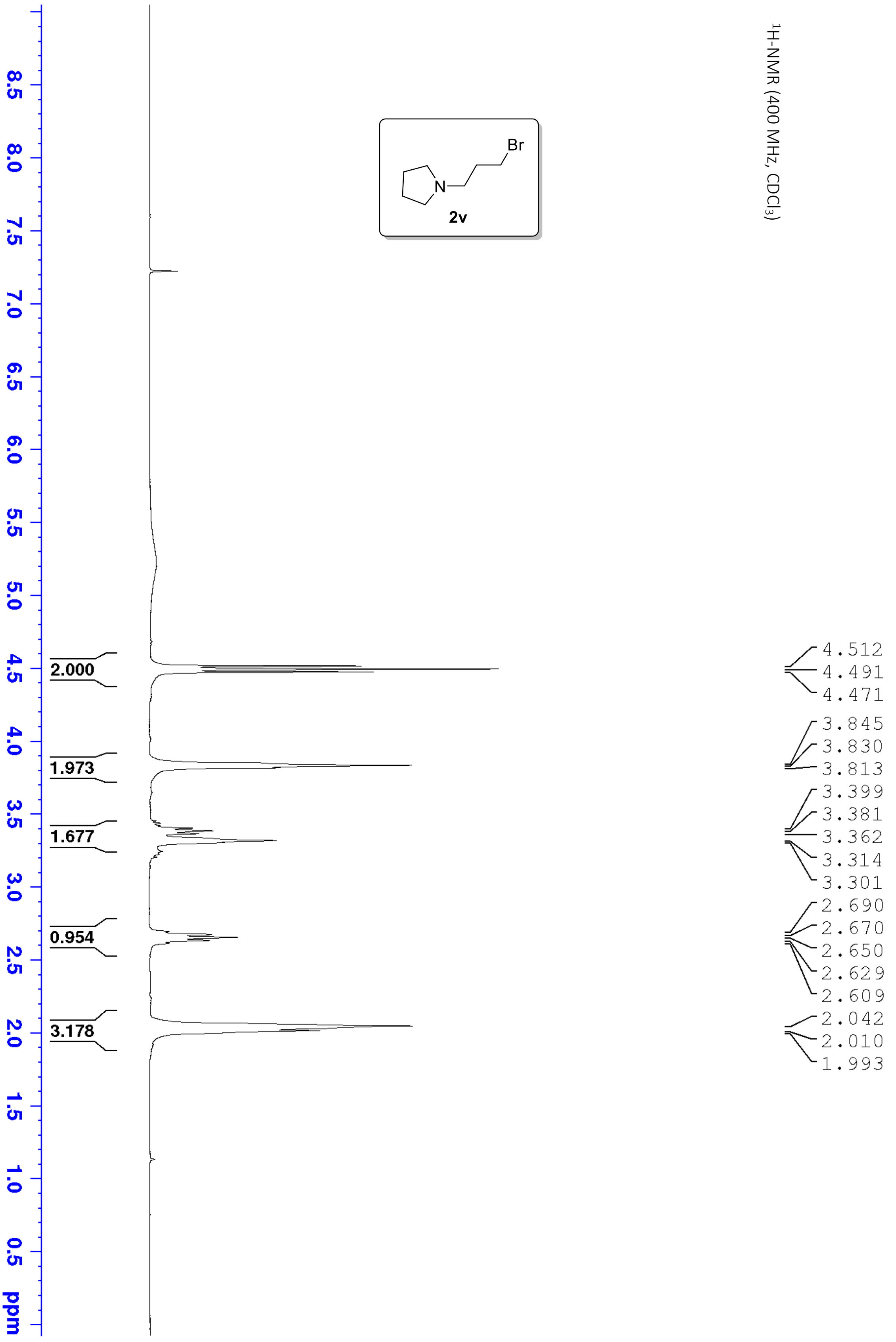

1H-NMR (400 MHz, CDCl3)
Br
N
2v
4.512
4.491
4.471
3.845
3.830
3.813
3.399
3.381
3.362
3.314
3.301
2.690
2.670
2.650
2.629
2.609
2.042
2.010
1.993
2.000
1.973
1.677
0.954
3.178
8.5
8.0
7.5
7.0
6.5
6.0
5.5
5.0
4.5
4.0
3.5
3.0
2.5
2.0
1.5
1.0
0.5
ppm

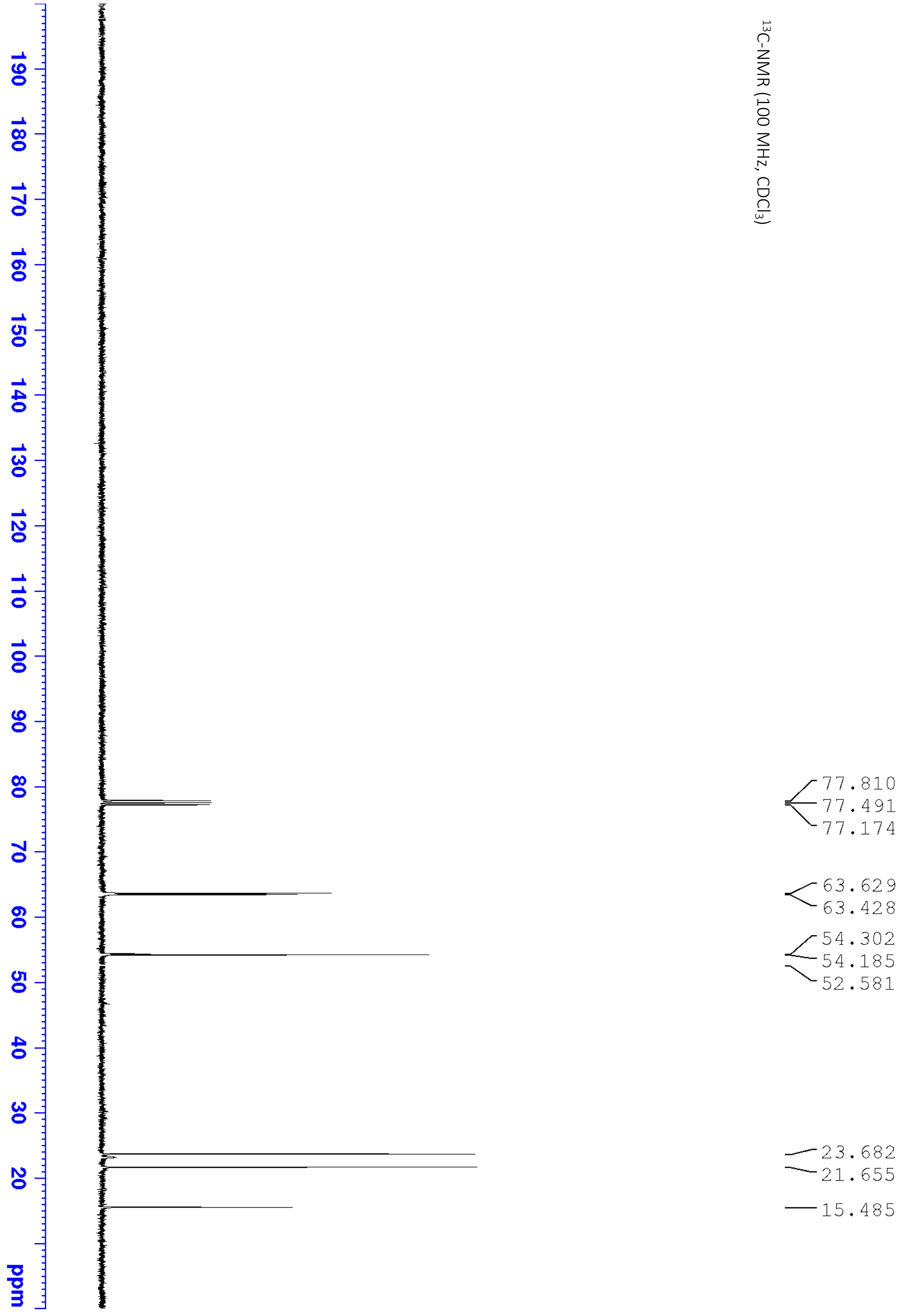
13C-NMR (100 MHz, CDCl3)
77.810
77.491
77.174
63.629
63.428
54.302
54.185
52.581
23.682
21.655
15.485
ppm

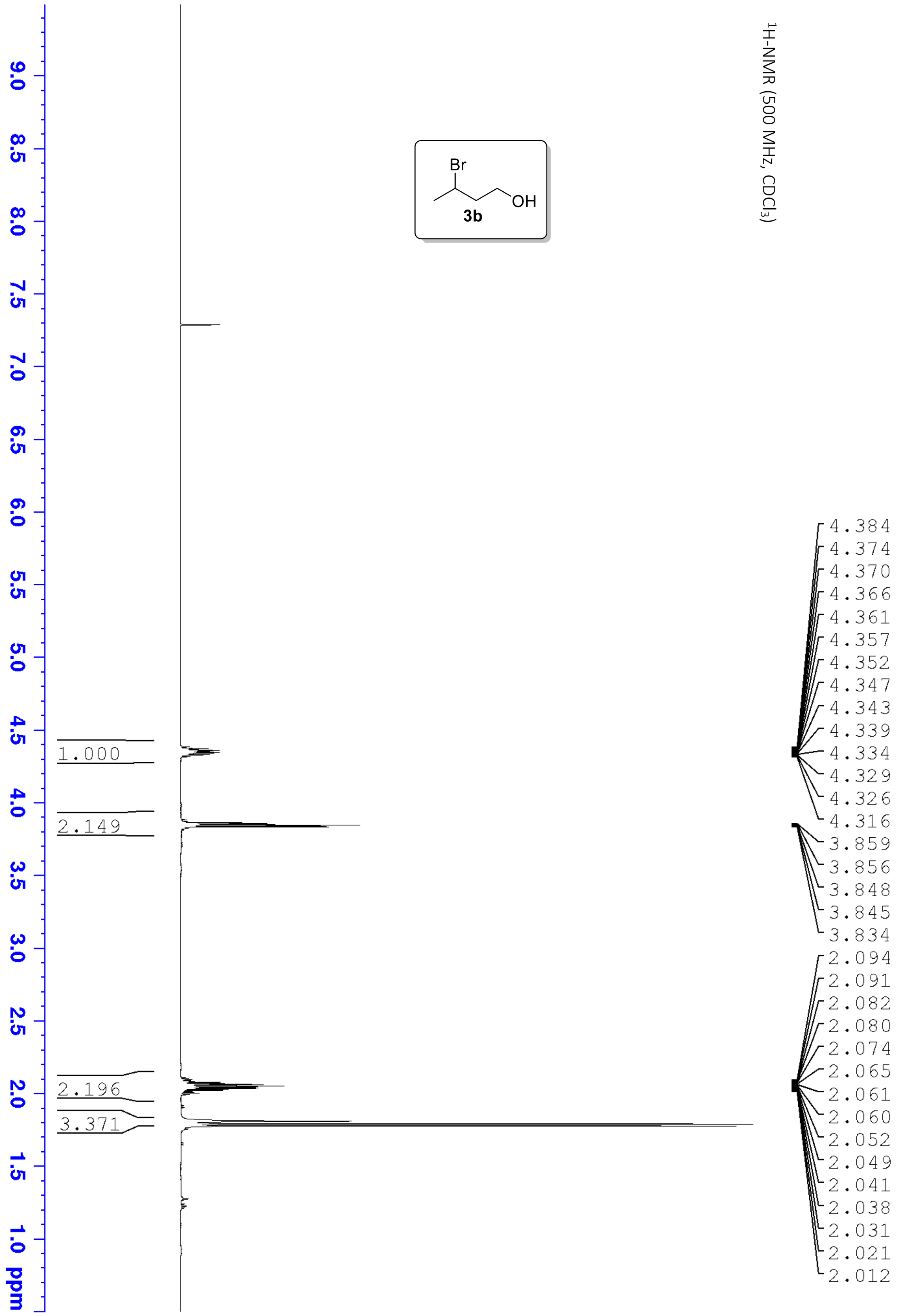

1H-NMR (500 MHz, CDCl3)
Br
OH
3b
4.384
4.374
4.370
4.366
4.361
4.357
4.352
4.347
4.343
4.339
4.334
4.329
4.326
4.316
3.859
3.856
3.848
3.845
3.834
2.094
2.091
2.082
2.080
2.074
2.065
2.061
2.060
2.052
2.049
2.041
2.038
2.031
2.021
2.012
1.000
2.149
2.196
3.371
9.0
8.5
8.0
7.5
7.0
6.5
6.0
5.5
5.0
4.5
4.0
3.5
3.0
2.5
2.0
1.5
1.0 ppm

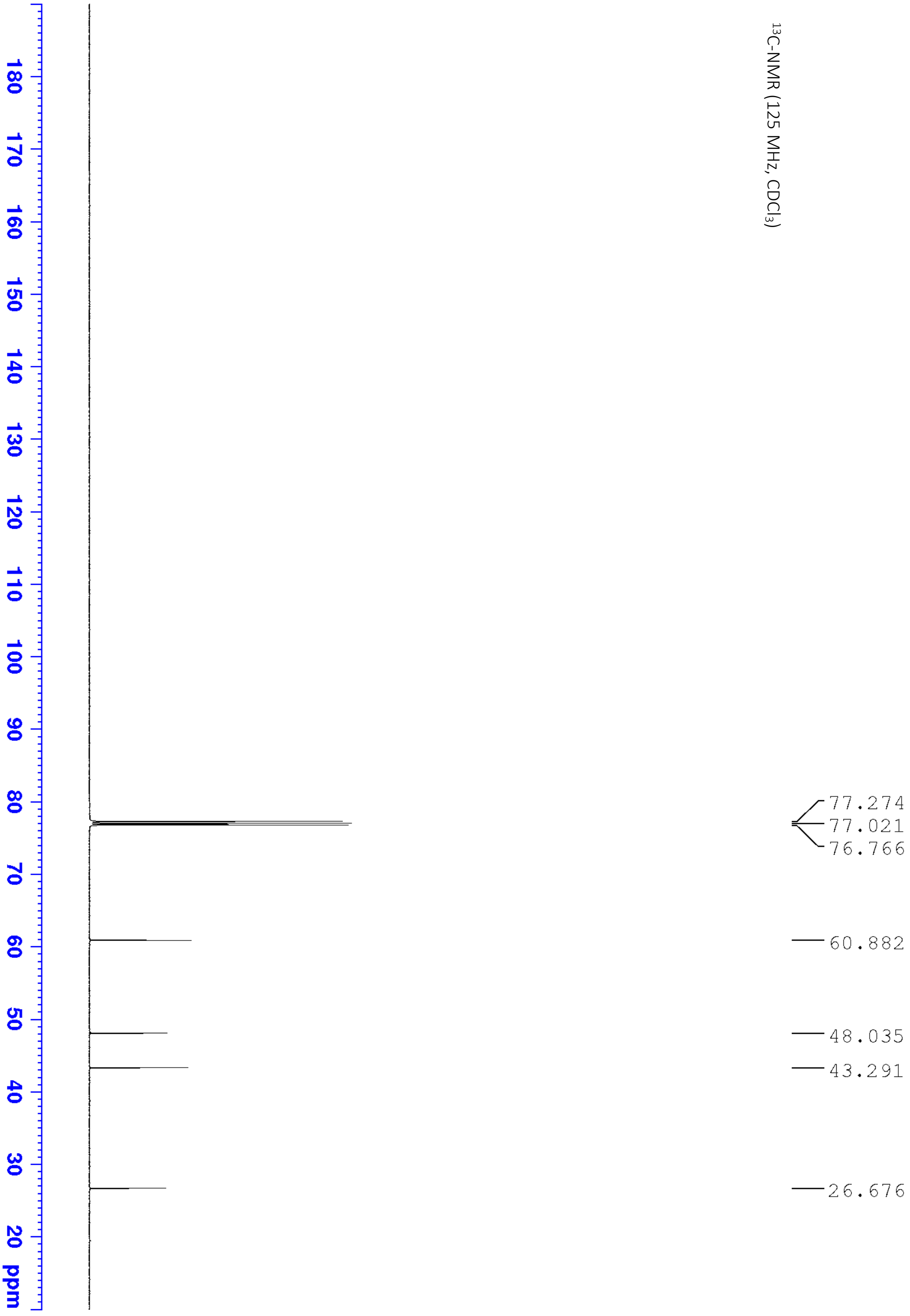

13C-NMR (125 MHz, CDCl3)
77.274
77.021
76.766
60.882
48.035
43.291
26.676
180 170 160 150 140 130 120 110 100 90 80 70 60 50 40 30 20 ppm

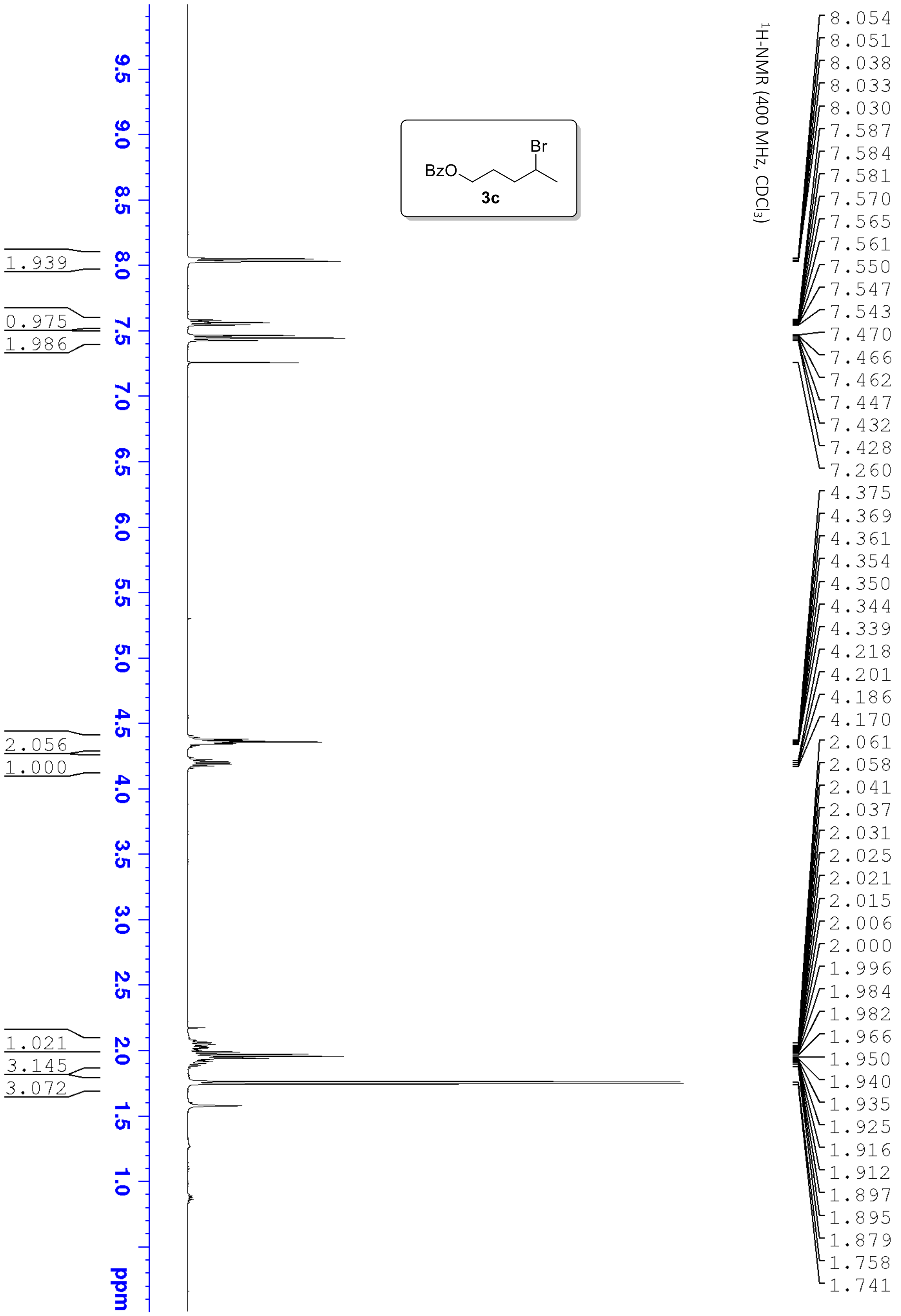
1H-NMR (400 MHz, CDCl3)
BzO
Br
3c
8.054
8.051
8.038
8.033
8.030
7.587
7.584
7.581
7.570
7.565
7.561
7.550
7.547
7.543
7.470
7.466
7.462
7.447
7.432
7.428
7.260
4.375
4.369
4.361
4.354
4.350
4.344
4.339
4.218
4.201
4.186
4.170
2.061
2.058
2.041
2.037
2.031
2.025
2.021
2.015
2.006
2.000
1.996
1.984
1.982
1.966
1.950
1.940
1.935
1.925
1.916
1.912
1.897
1.895
1.879
1.758
1.741
1.939
0.975
1.986
2.056
1.000
1.021
3.145
3.072
9.5
9.0
8.5
8.0
7.5
7.0
6.5
6.0
5.5
5.0
4.5
4.0
3.5
3.0
2.5
2.0
1.5
1.0
ppm

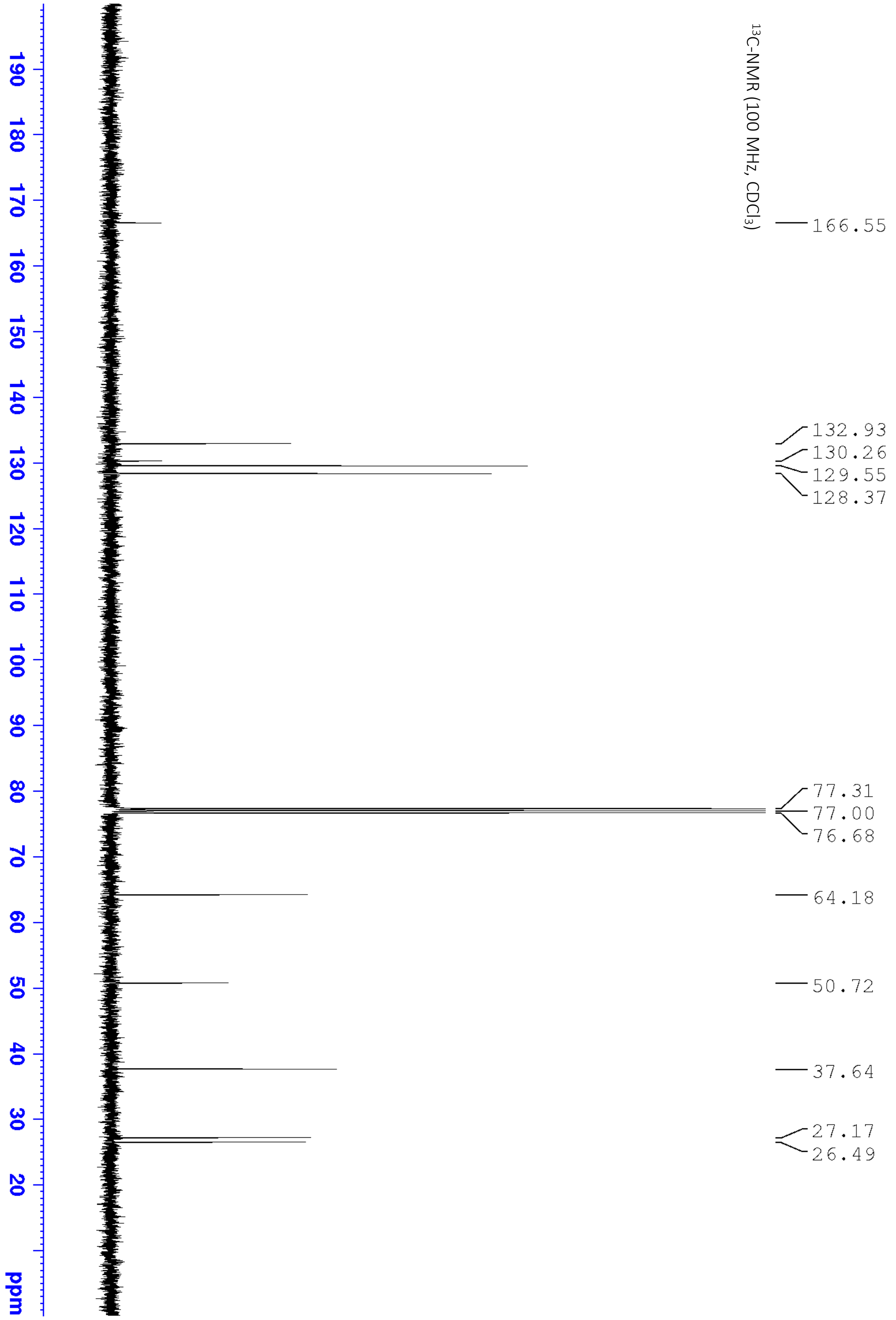

13C-NMR (100 MHz, CDCl3)
166.55
132.93
130.26
129.55
128.37
77.31
77.00
76.68
64.18
50.72
37.64
27.17
26.49
190
180
170
160
150
140
130
120
110
100
90
80
70
60
50
40
30
20
ppm

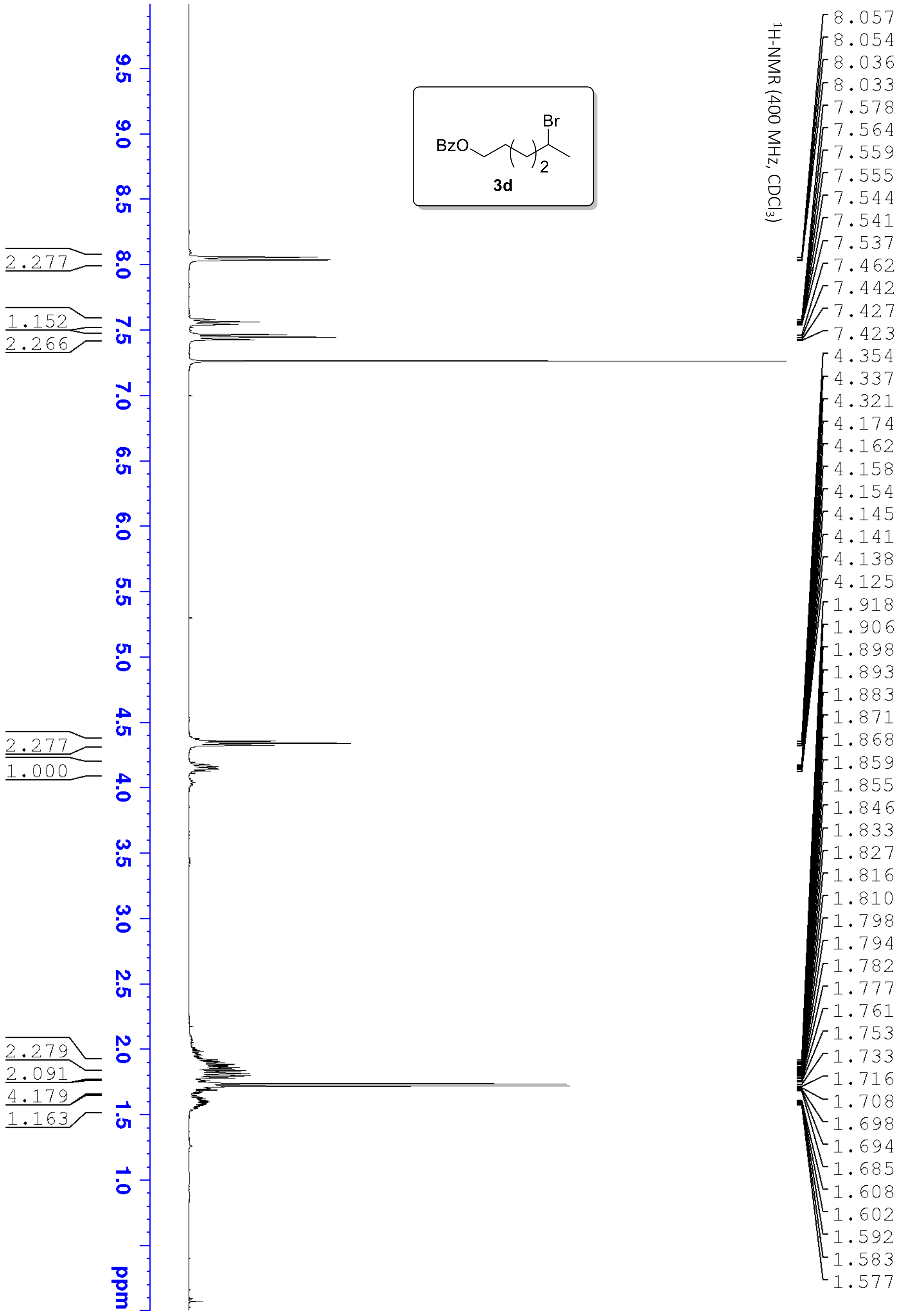
1H-NMR (400 MHz, CDCl3)
BzO
Br
2
3d
8.057
8.054
8.036
8.033
7.578
7.564
7.559
7.555
7.544
7.541
7.537
7.462
7.442
7.427
7.423
4.354
4.337
4.321
4.174
4.162
4.158
4.154
4.145
4.141
4.138
4.125
1.918
1.906
1.898
1.893
1.883
1.871
1.868
1.859
1.855
1.846
1.833
1.827
1.816
1.810
1.798
1.794
1.782
1.777
1.761
1.753
1.733
1.716
1.708
1.698
1.694
1.685
1.608
1.602
1.592
1.583
1.577
2.277
1.152
2.266
2.277
1.000
2.279
2.091
4.179
1.163
9.5
9.0
8.5
8.0
7.5
7.0
6.5
6.0
5.5
5.0
4.5
4.0
3.5
3.0
2.5
2.0
1.5
1.0
ppm

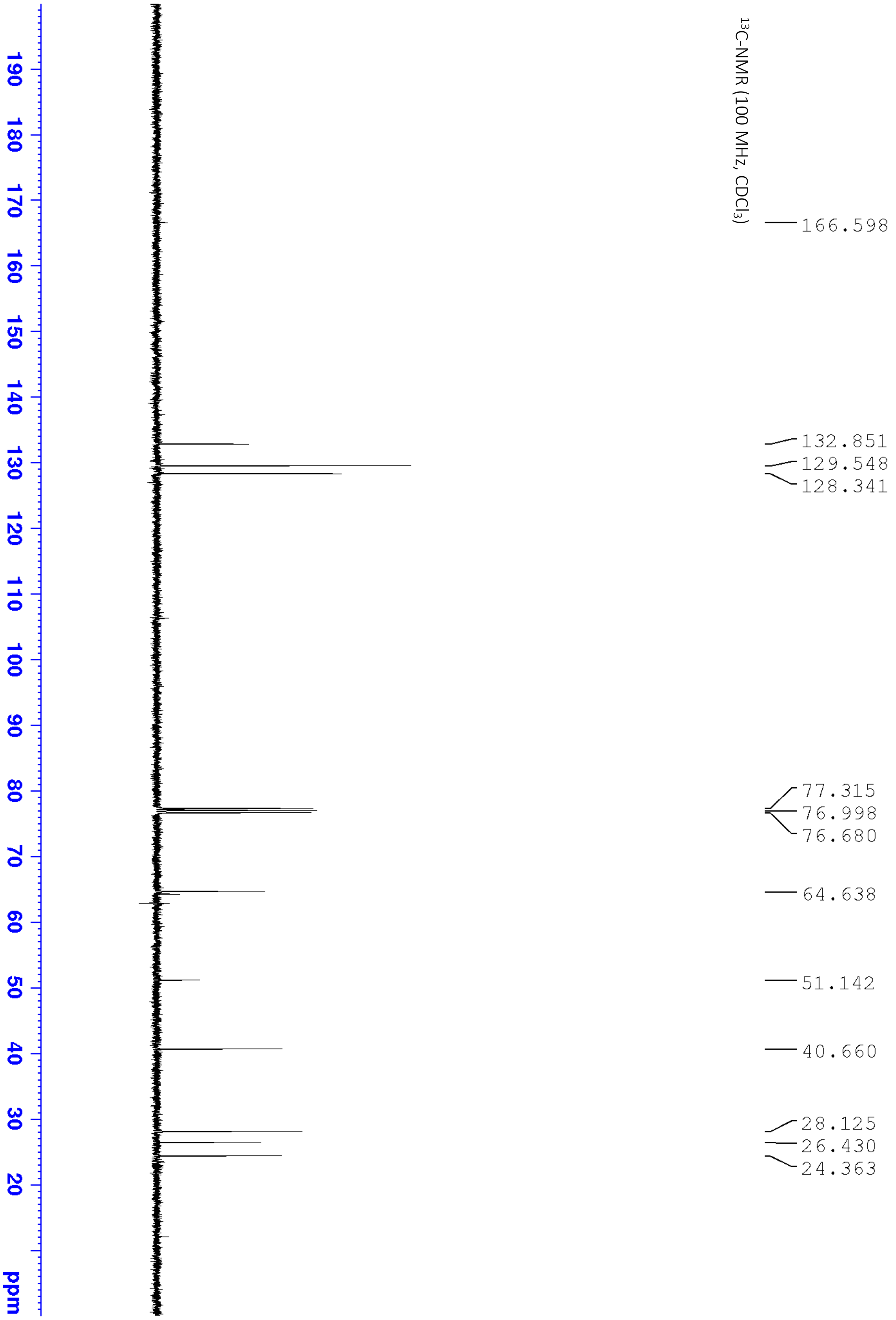

$^{13}$C-NMR (100 MHz, CDCl$_3$)
166.598
132.851
129.548
128.341
77.315
76.998
76.680
64.638
51.142
40.660
28.125
26.430
24.363
190
180
170
160
150
140
130
120
110
100
90
80
70
60
50
40
30
20
ppm

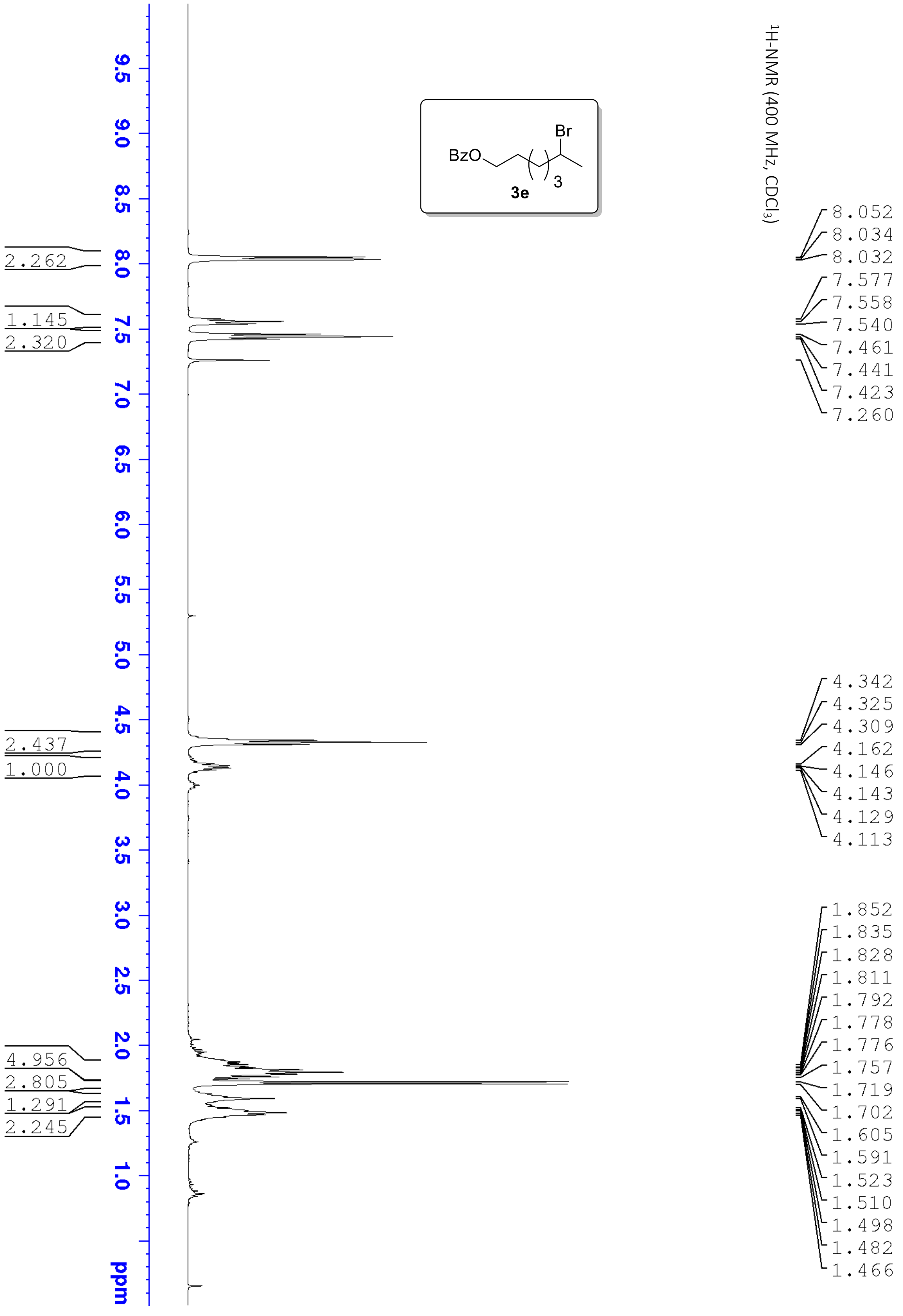

1H-NMR (400 MHz, CDCl3)
BzO
Br
3
3e
8.052
8.034
8.032
7.577
7.558
7.540
7.461
7.441
7.423
7.260
4.342
4.325
4.309
4.162
4.146
4.143
4.129
4.113
1.852
1.835
1.828
1.811
1.792
1.778
1.776
1.757
1.719
1.702
1.605
1.591
1.523
1.510
1.498
1.482
1.466
2.262
1.145
2.320
2.437
1.000
4.956
2.805
1.291
2.245
9.5
9.0
8.5
8.0
7.5
7.0
6.5
6.0
5.5
5.0
4.5
4.0
3.5
3.0
2.5
2.0
1.5
1.0
ppm

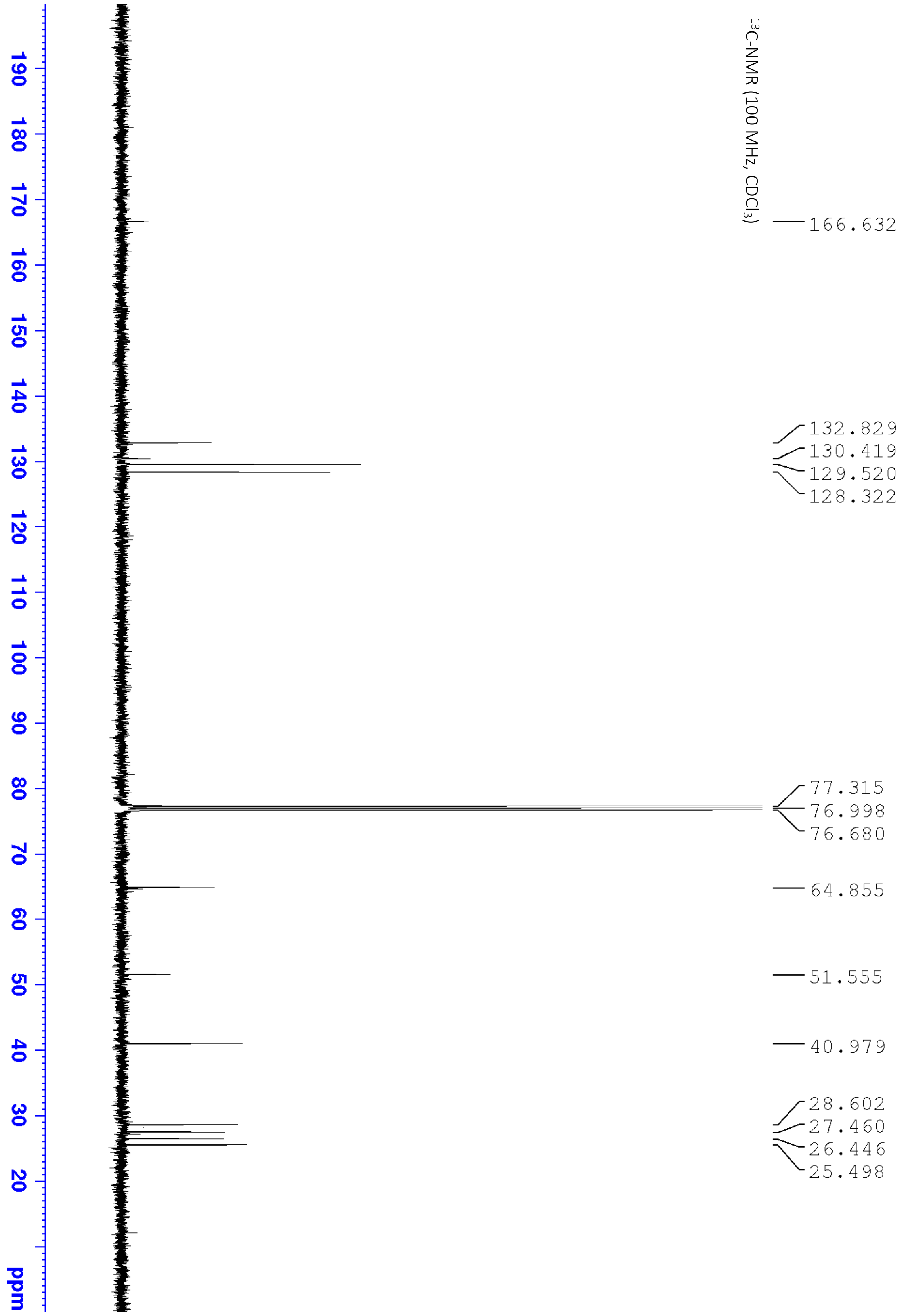

13C-NMR (100 MHz, CDCl3)
166.632
132.829
130.419
129.520
128.322
77.315
76.998
76.680
64.855
51.555
40.979
28.602
27.460
26.446
25.498
190
180
170
160
150
140
130
120
110
100
90
80
70
60
50
40
30
20
ppm

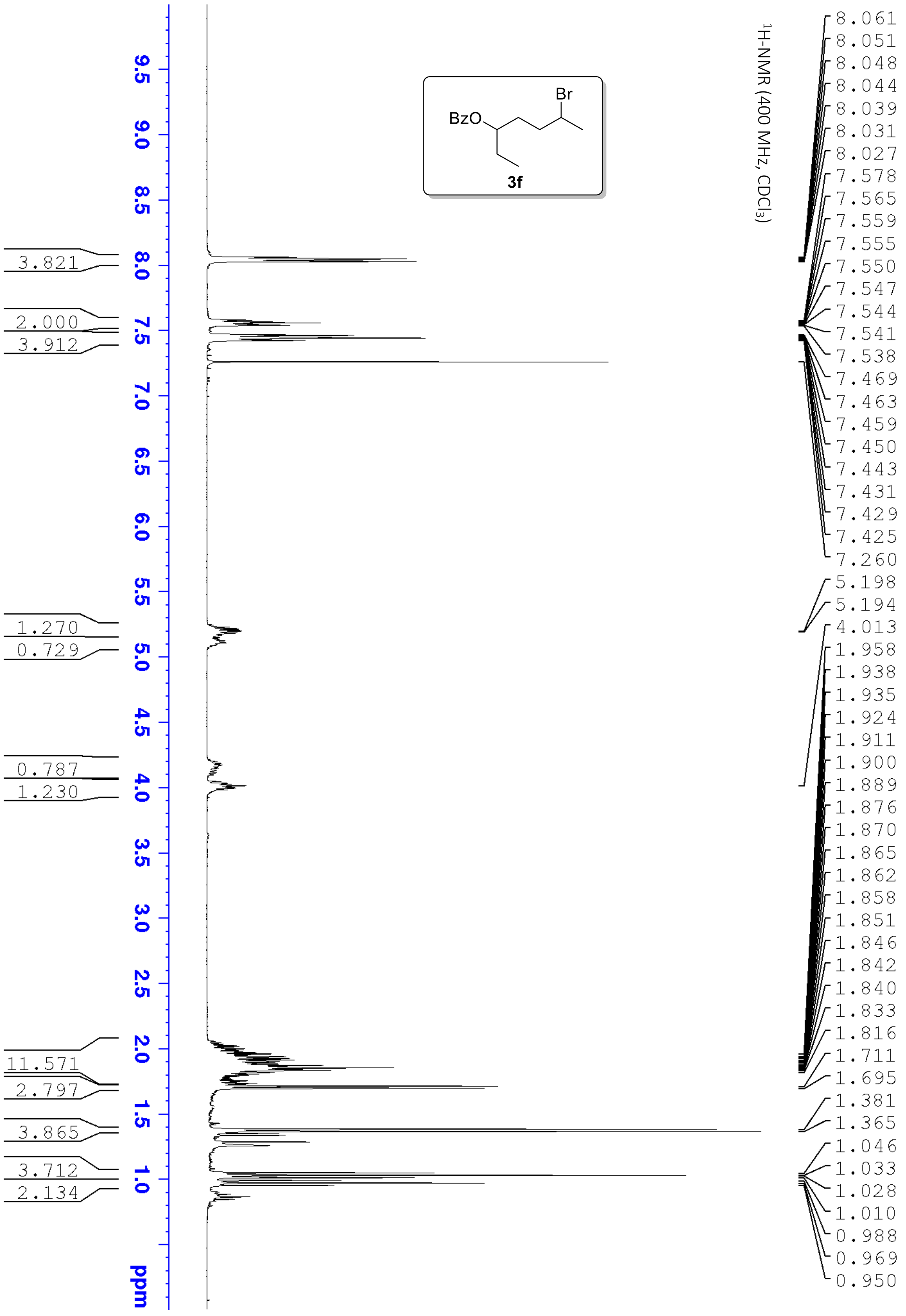
1H-NMR (400 MHz, CDCl3)
BzO
Br
3f
8.061
8.051
8.048
8.044
8.039
8.031
8.027
7.578
7.565
7.559
7.555
7.550
7.547
7.544
7.541
7.538
7.469
7.463
7.459
7.450
7.443
7.431
7.429
7.425
7.260
5.198
5.194
4.013
1.958
1.938
1.935
1.924
1.911
1.900
1.889
1.876
1.870
1.865
1.862
1.858
1.851
1.846
1.842
1.840
1.833
1.816
1.711
1.695
1.381
1.365
1.046
1.033
1.028
1.010
0.988
0.969
0.950
3.821
2.000
3.912
1.270
0.729
0.787
1.230
11.571
2.797
3.865
3.712
2.134
ppm

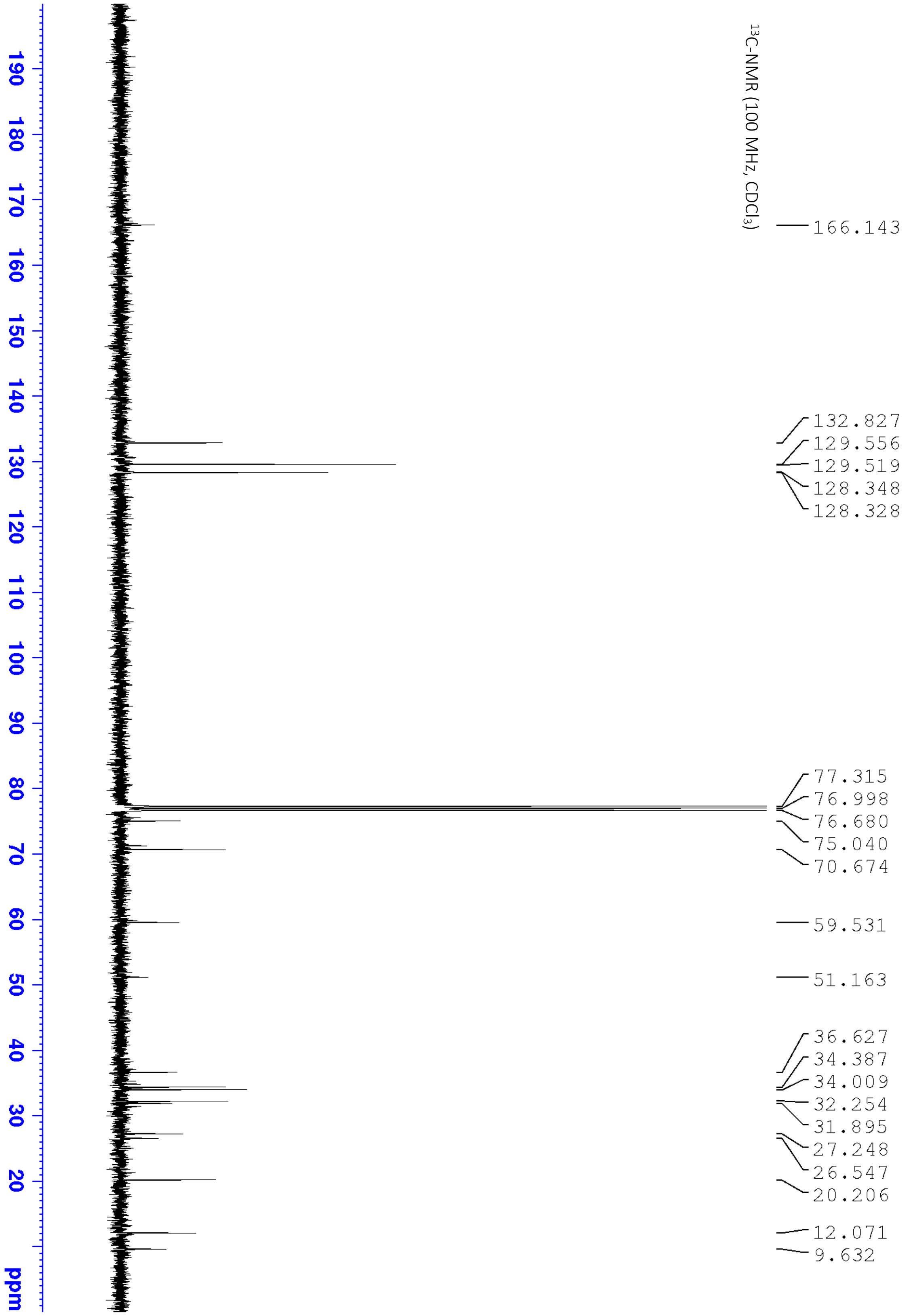

13C-NMR (100 MHz, CDCl3)
166.143
132.827
129.556
129.519
128.348
128.328
77.315
76.998
76.680
75.040
70.674
59.531
51.163
36.627
34.387
34.009
32.254
31.895
27.248
26.547
20.206
12.071
9.632
190
180
170
160
150
140
130
120
110
100
90
80
70
60
50
40
30
20
ppm

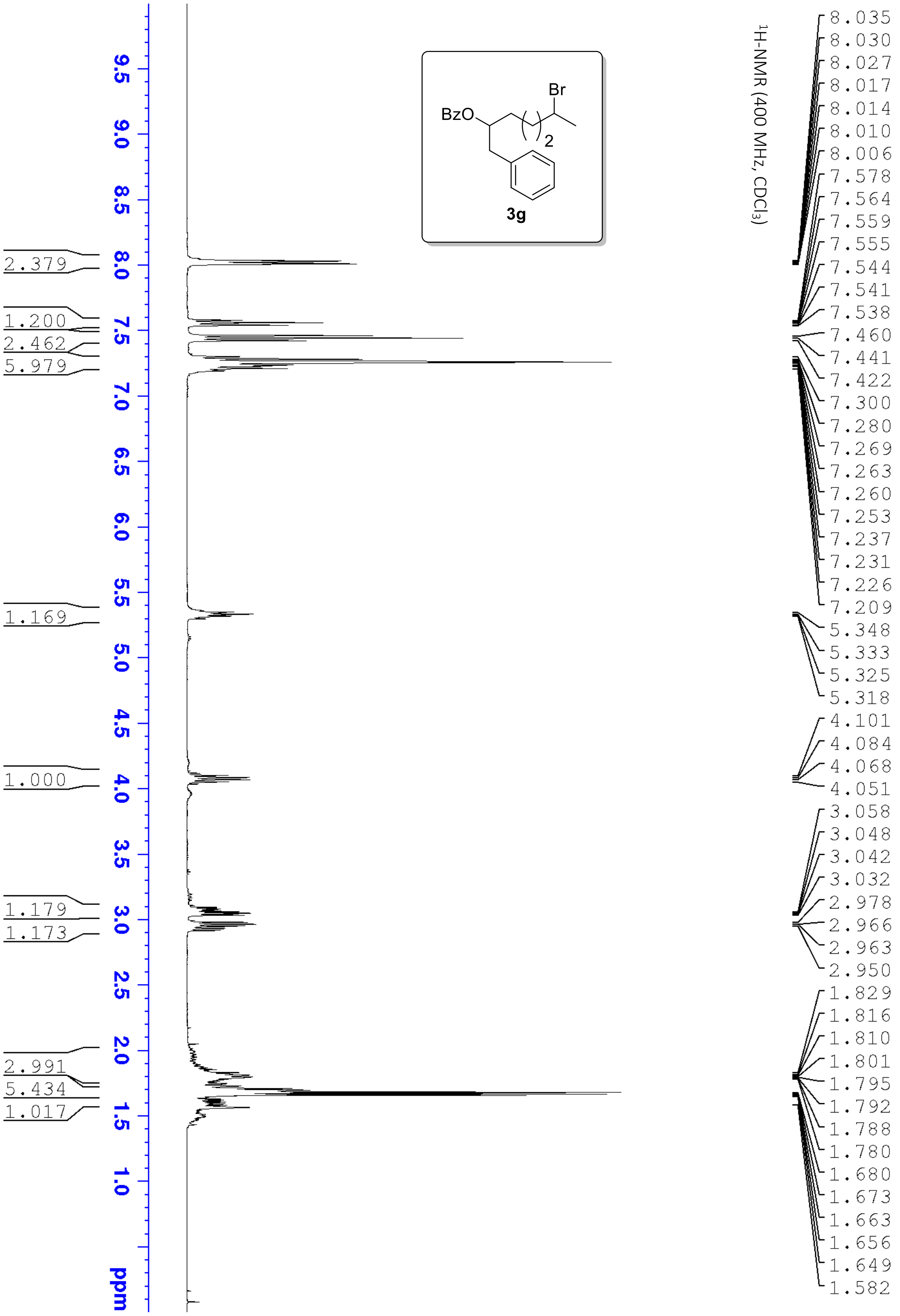
1H-NMR (400 MHz, CDCl3)
BzO
Br
2
3g
8.035
8.030
8.027
8.017
8.014
8.010
8.006
7.578
7.564
7.559
7.555
7.544
7.541
7.538
7.460
7.441
7.422
7.300
7.280
7.269
7.263
7.260
7.253
7.237
7.231
7.226
7.209
5.348
5.333
5.325
5.318
4.101
4.084
4.068
4.051
3.058
3.048
3.042
3.032
2.978
2.966
2.963
2.950
1.829
1.816
1.810
1.801
1.795
1.792
1.788
1.780
1.680
1.673
1.663
1.656
1.649
1.582
2.379
1.200
2.462
5.979
1.169
1.000
1.179
1.173
2.991
5.434
1.017
9.5
9.0
8.5
8.0
7.5
7.0
6.5
6.0
5.5
5.0
4.5
4.0
3.5
3.0
2.5
2.0
1.5
1.0
ppm

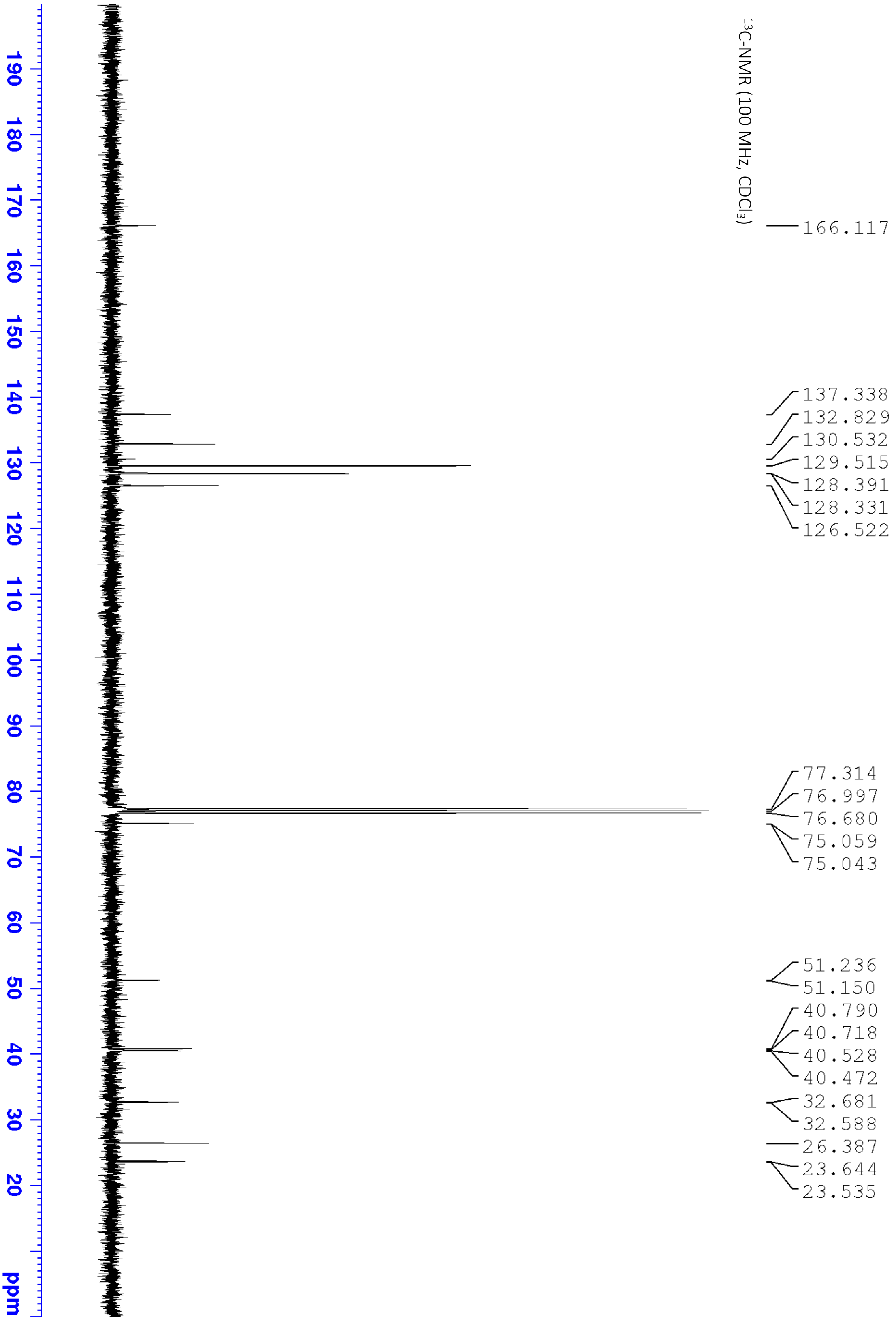

13C-NMR (100 MHz, CDCl3)
166.117
137.338
132.829
130.532
129.515
128.391
128.331
126.522
77.314
76.997
76.680
75.059
75.043
51.236
51.150
40.790
40.718
40.528
40.472
32.681
32.588
26.387
23.644
23.535
190
180
170
160
150
140
130
120
110
100
90
80
70
60
50
40
30
20
ppm

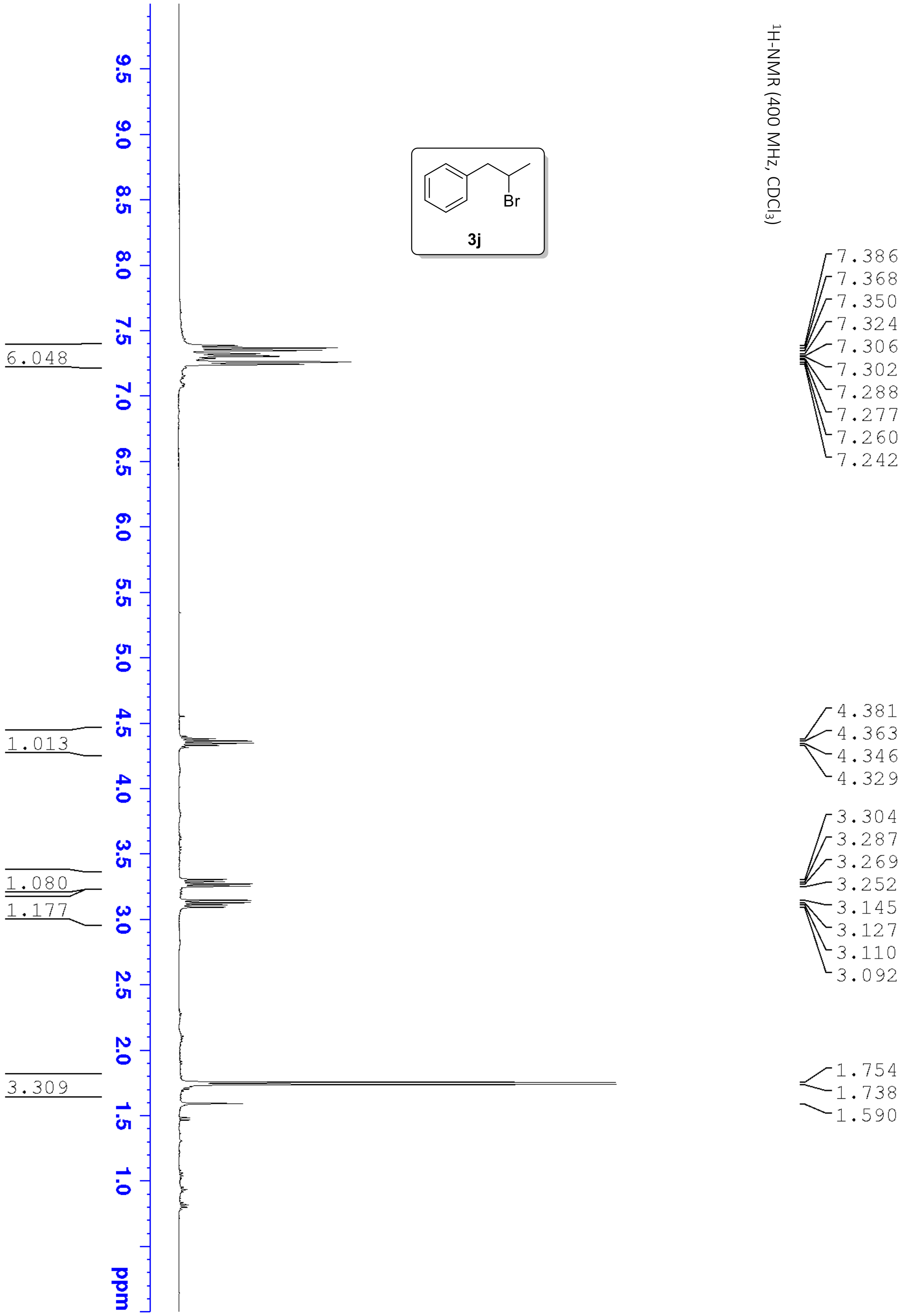

$^1$H-NMR (400 MHz, CDCl$_3$)
Br
3j
7.386
7.368
7.350
7.324
7.306
7.302
7.288
7.277
7.260
7.242
4.381
4.363
4.346
4.329
3.304
3.287
3.269
3.252
3.145
3.127
3.110
3.092
1.754
1.738
1.590
6.048
1.013
1.080
1.177
3.309
9.5
9.0
8.5
8.0
7.5
7.0
6.5
6.0
5.5
5.0
4.5
4.0
3.5
3.0
2.5
2.0
1.5
1.0
ppm

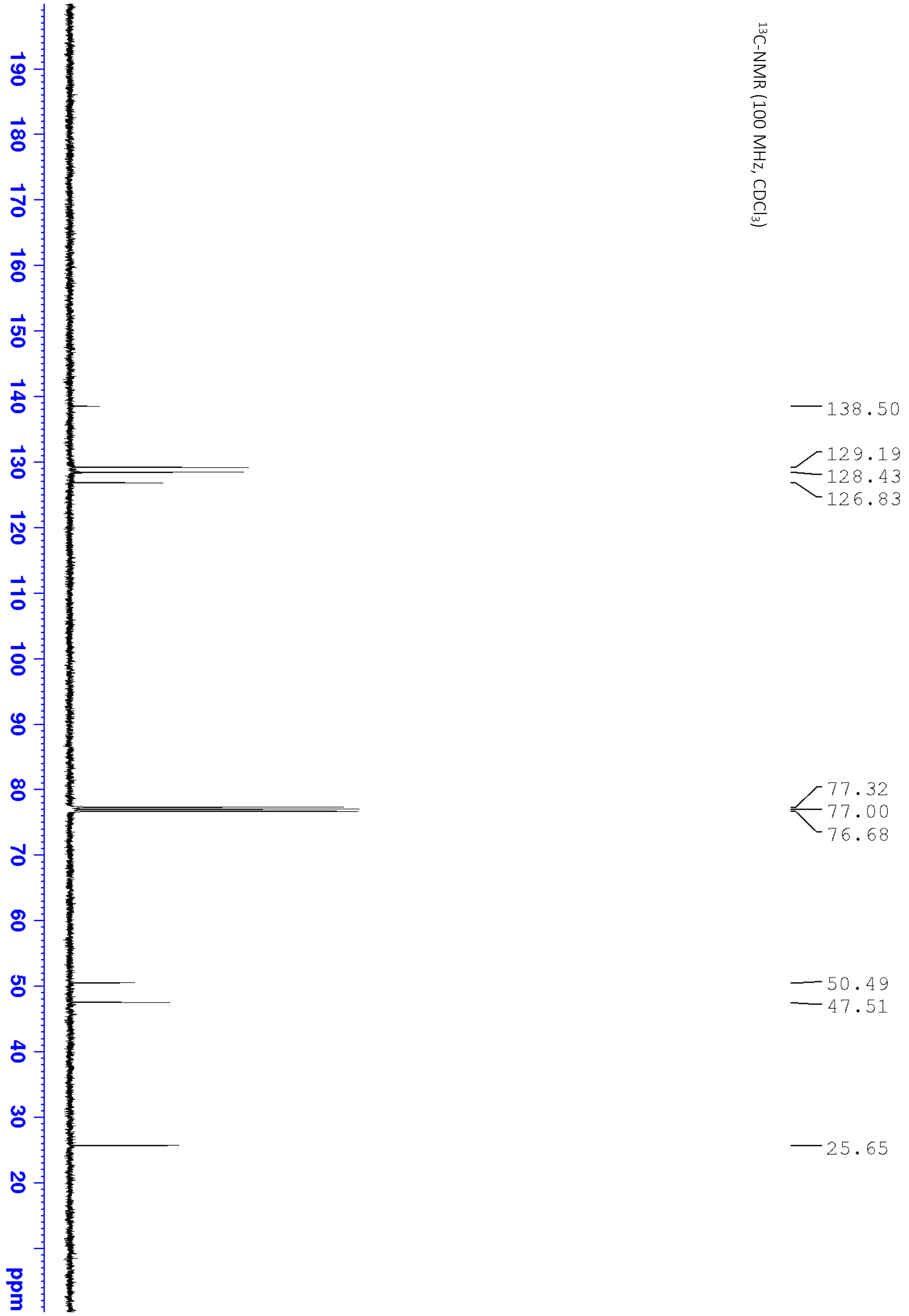

13C-NMR (100 MHz, CDCl3)
138.50
129.19
128.43
126.83
77.32
77.00
76.68
50.49
47.51
25.65
190
180
170
160
150
140
130
120
110
100
90
80
70
60
50
40
30
20
ppm

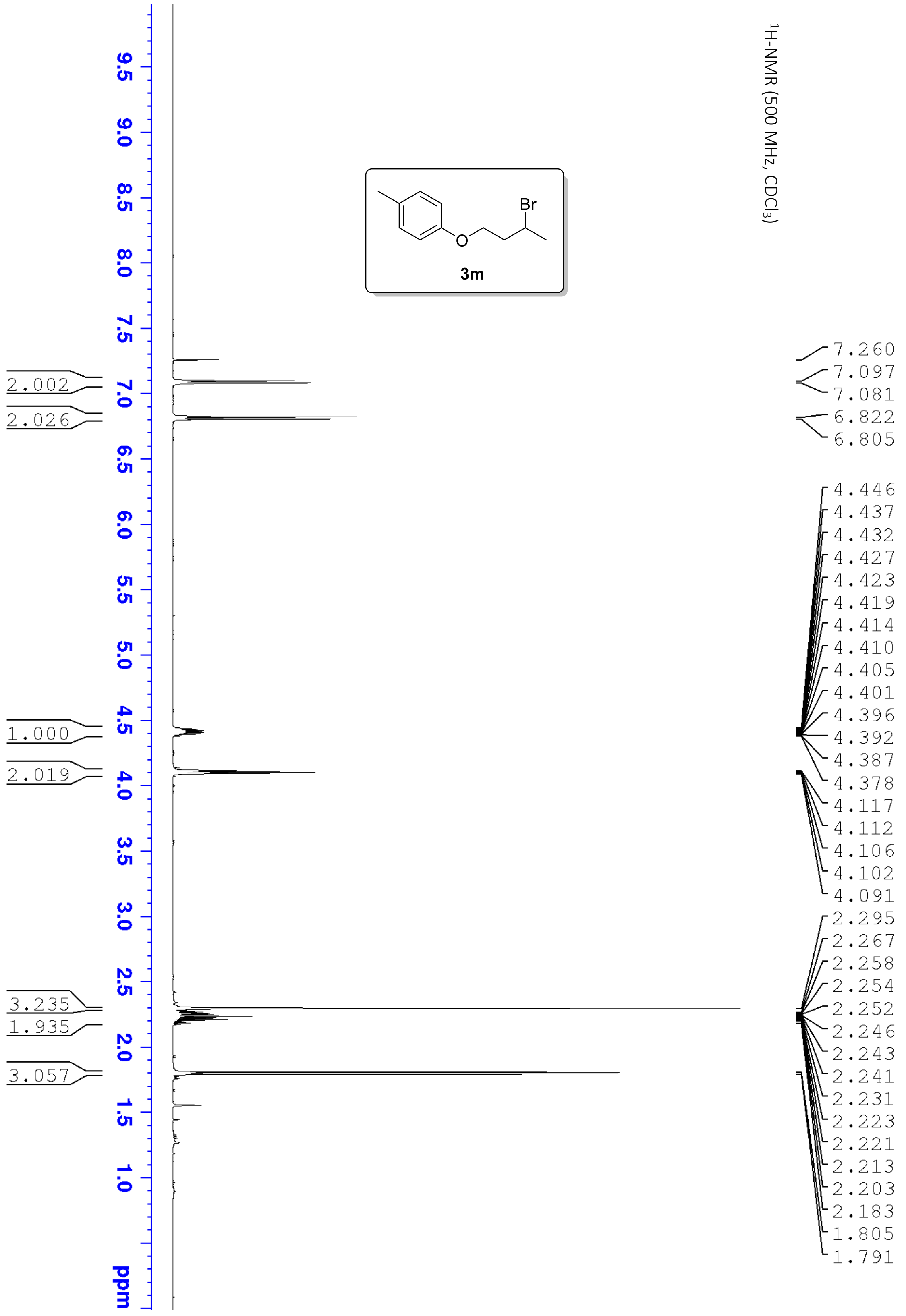
¹H-NMR (500 MHz, CDCl3)
Br
O
3m
ppm

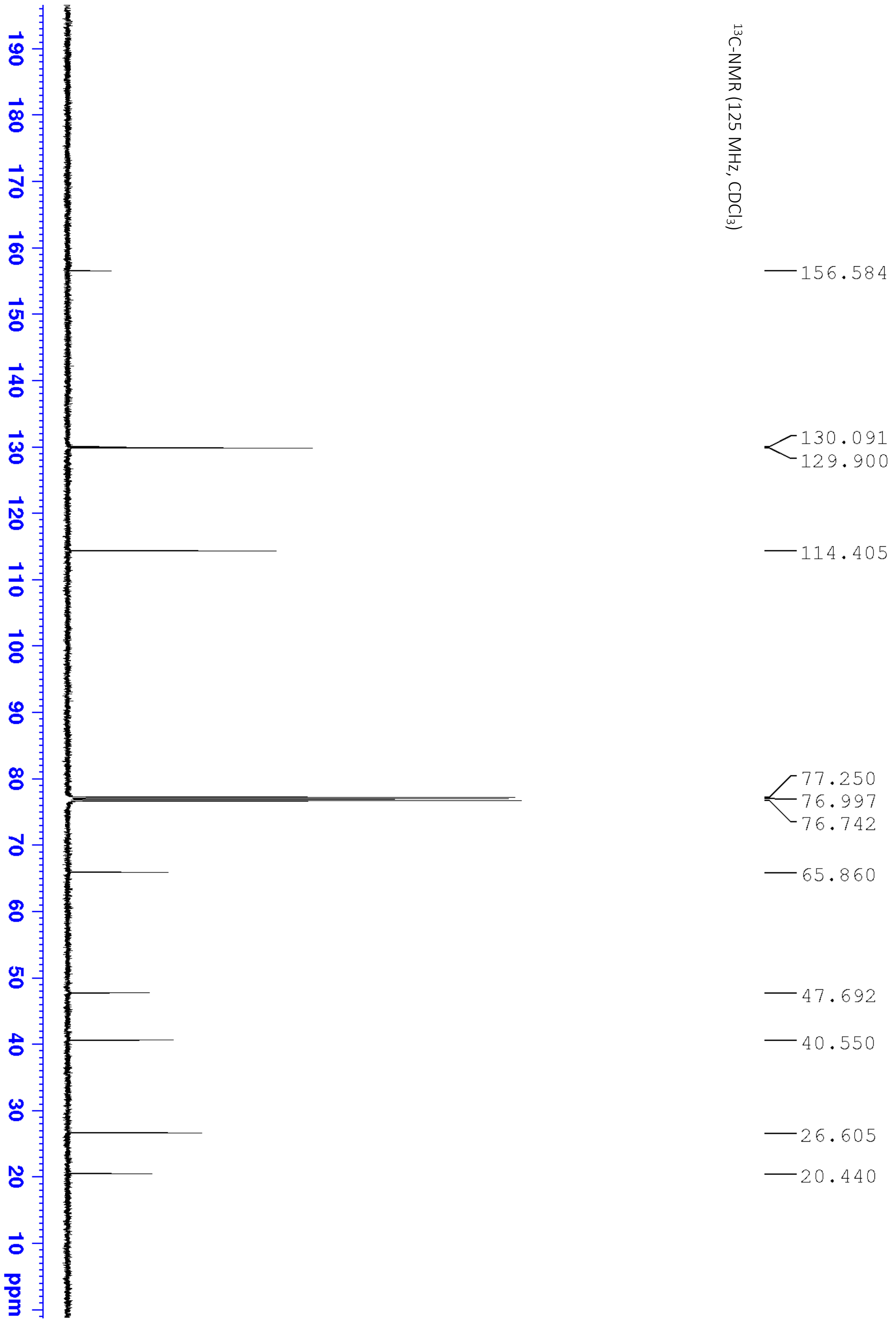

13C-NMR (125 MHz, CDCl3)
156.584
130.091
129.900
114.405
77.250
76.997
76.742
65.860
47.692
40.550
26.605
20.440
190 180 170 160 150 140 130 120 110 100 90 80 70 60 50 40 30 20 10 ppm

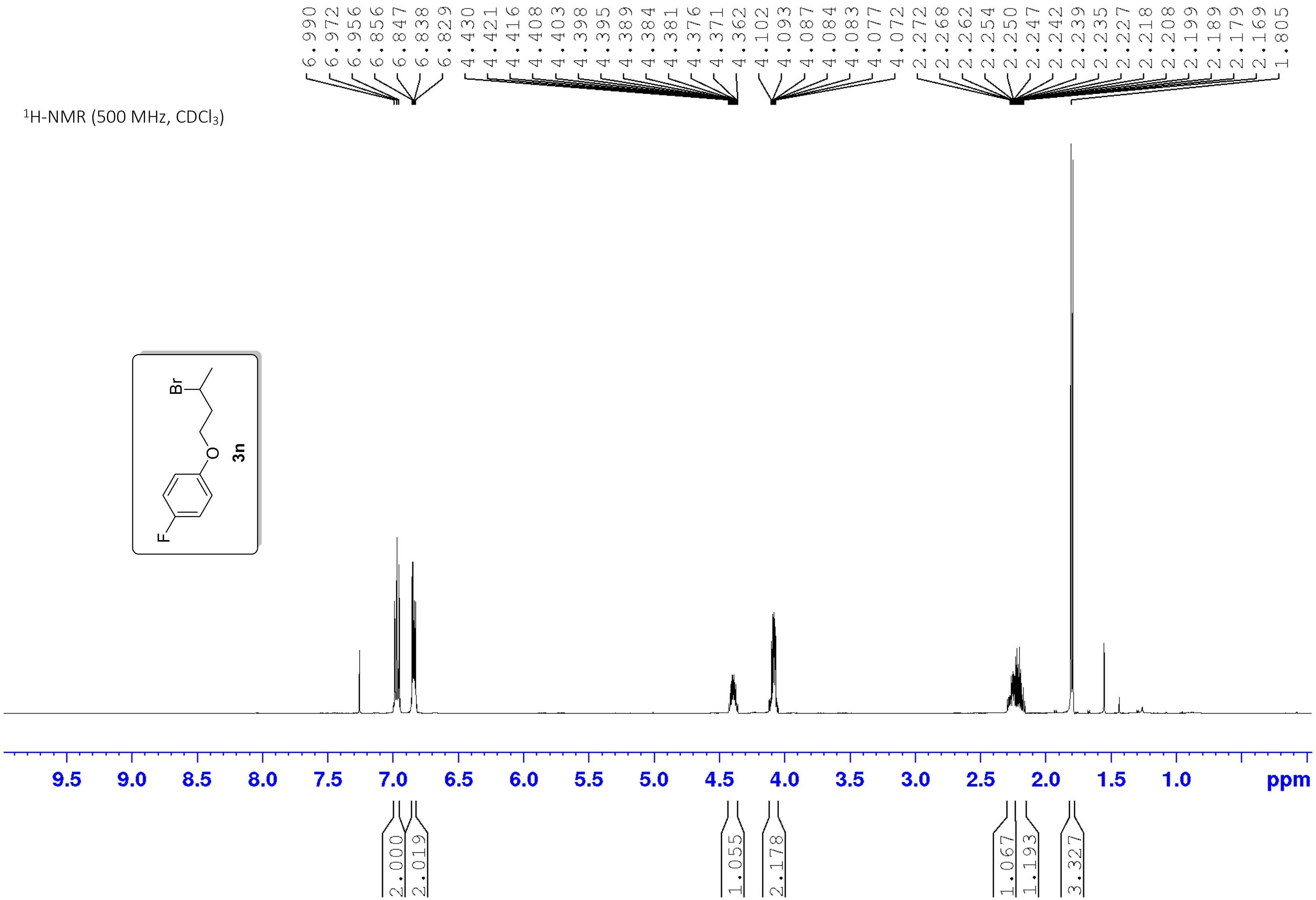

1H-NMR (500 MHz, CDCl3)
6.990
6.972
6.956
6.856
6.847
6.838
6.829
4.430
4.421
4.416
4.408
4.403
4.398
4.395
4.389
4.384
4.381
4.376
4.371
4.362
4.102
4.093
4.087
4.084
4.083
4.077
4.072
2.272
2.268
2.262
2.254
2.250
2.247
2.242
2.239
2.235
2.227
2.218
2.208
2.199
2.189
2.179
2.169
1.805
F
O
Br
3n
9.5
9.0
8.5
8.0
7.5
7.0
6.5
6.0
5.5
5.0
4.5
4.0
3.5
3.0
2.5
2.0
1.5
1.0
ppm
2.000
2.019
1.055
2.178
1.067
1.193
3.327

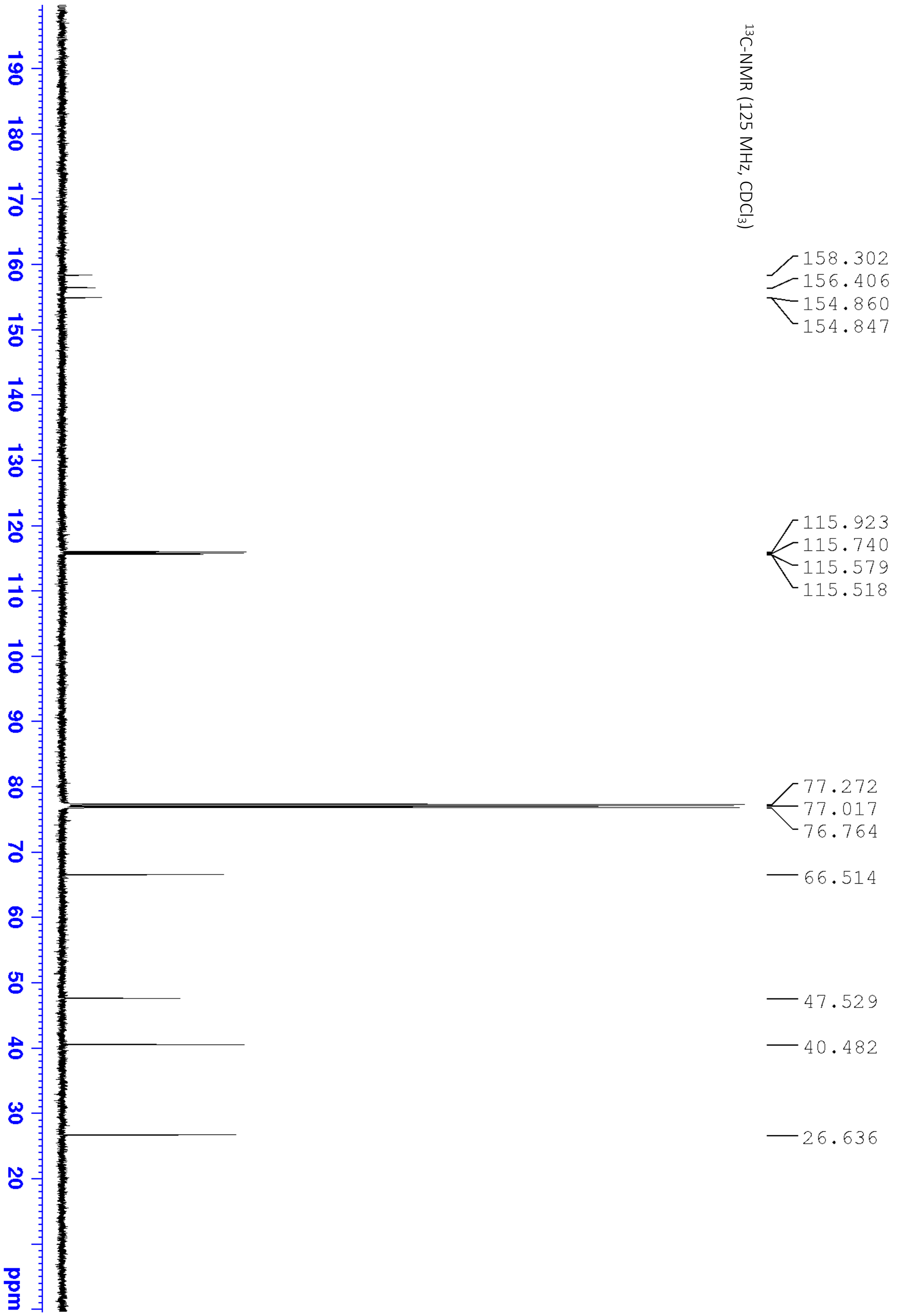

13C-NMR (125 MHz, CDCl3)
158.302
156.406
154.860
154.847
115.923
115.740
115.579
115.518
77.272
77.017
76.764
66.514
47.529
40.482
26.636
190
180
170
160
150
140
130
120
110
100
90
80
70
60
50
40
30
20
ppm

$^{1}$H-NMR (500 MHz, $CDCl_3$)

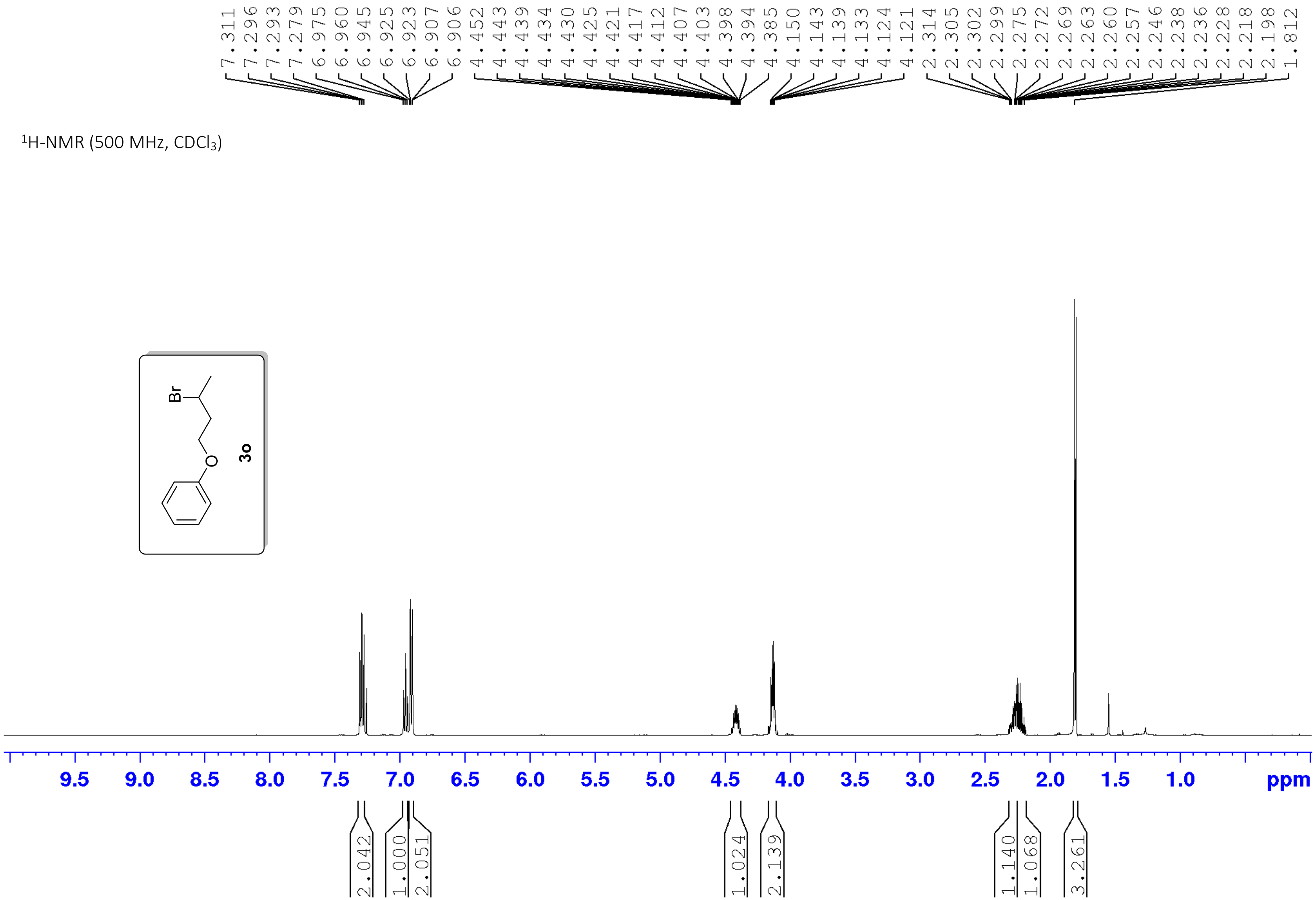

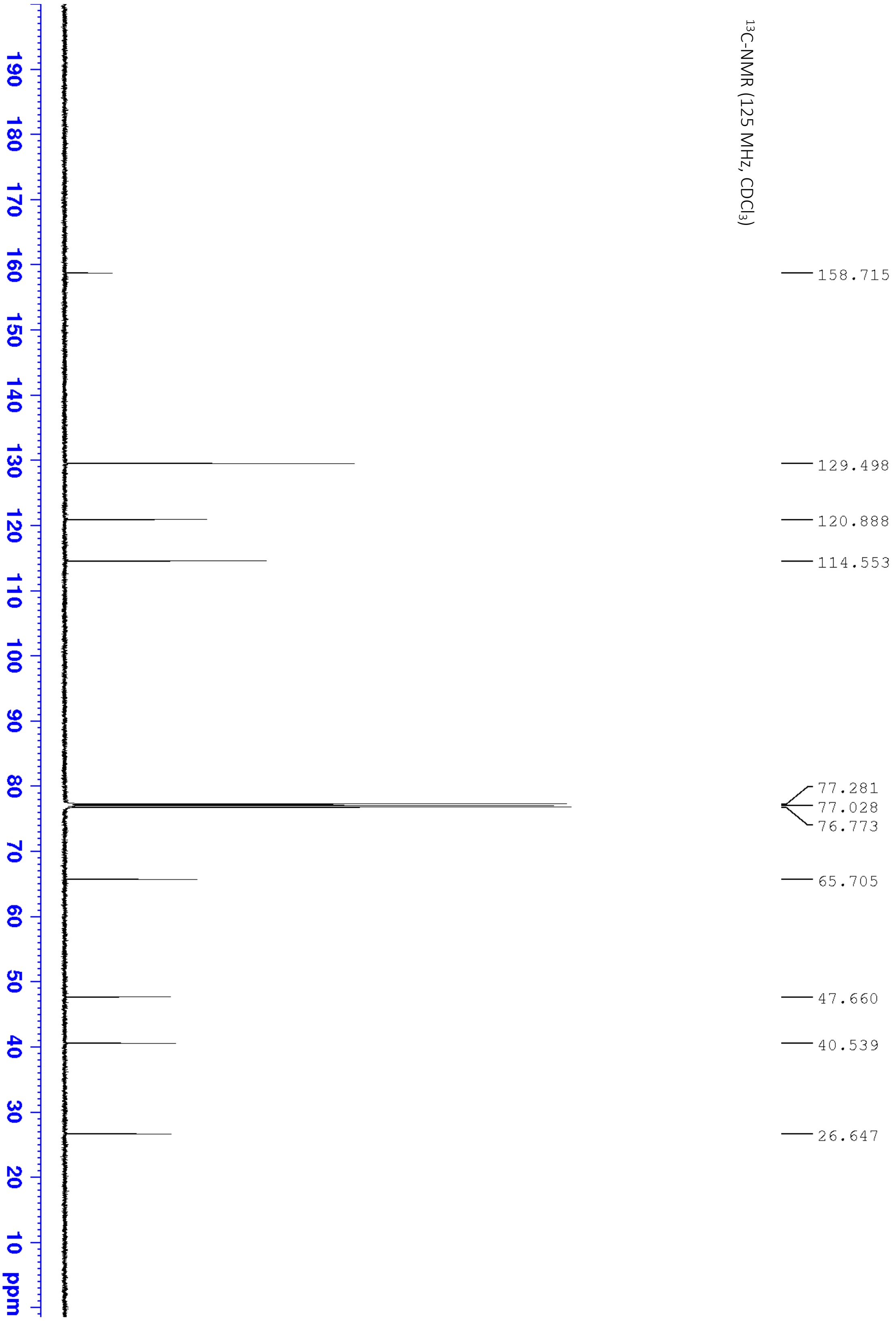

$^{13}$C-NMR (125 MHz, CDCl$_3$)
158.715
129.498
120.888
114.553
77.281
77.028
76.773
65.705
47.660
40.539
26.647
190
180
170
160
150
140
130
120
110
100
90
80
70
60
50
40
30
20
10
ppm

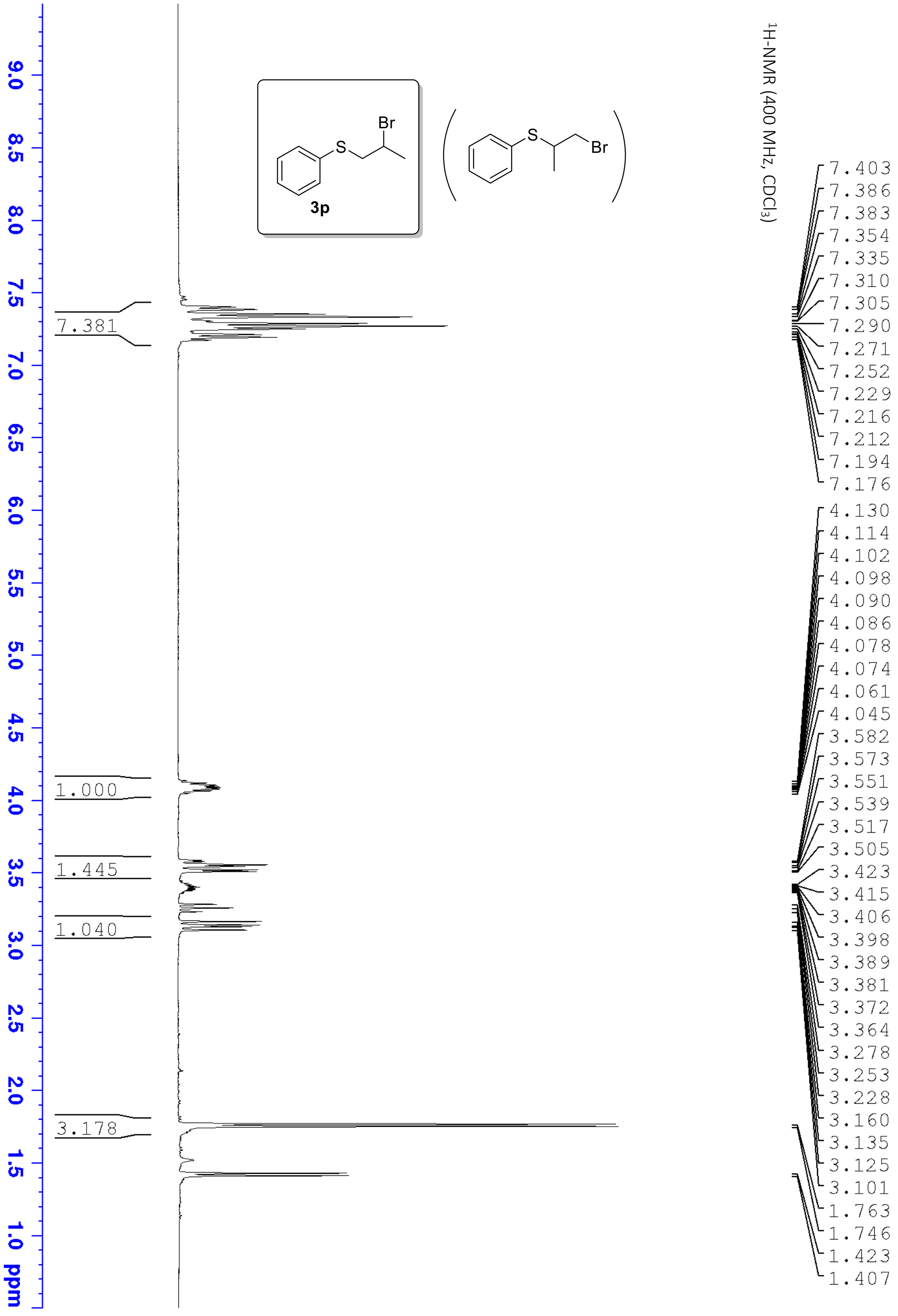

$^{1}$H-NMR (400 MHz, $CDCl_3$)
Br
S
3p
S
Br
7.403
7.386
7.383
7.354
7.335
7.310
7.305
7.290
7.271
7.252
7.229
7.216
7.212
7.194
7.176
4.130
4.114
4.102
4.098
4.090
4.086
4.078
4.074
4.061
4.045
3.582
3.573
3.551
3.539
3.517
3.505
3.423
3.415
3.406
3.398
3.389
3.381
3.372
3.364
3.278
3.253
3.228
3.160
3.135
3.125
3.101
1.763
1.746
1.423
1.407
7.381
1.000
1.445
1.040
3.178
9.0
8.5
8.0
7.5
7.0
6.5
6.0
5.5
5.0
4.5
4.0
3.5
3.0
2.5
2.0
1.5
1.0 ppm

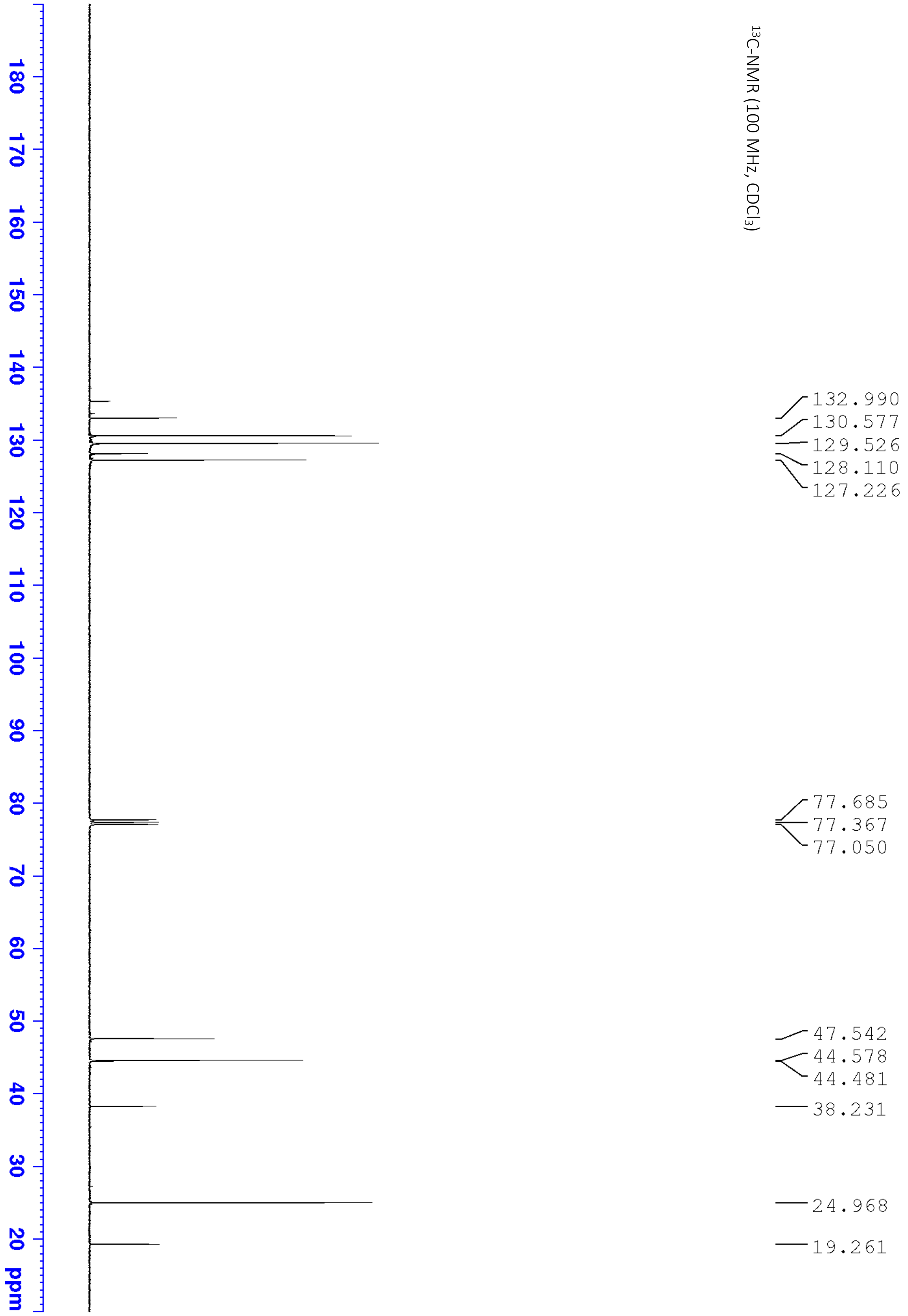

$^{13}$C-NMR (100 MHz, CDCl$_3$)
132.990
130.577
129.526
128.110
127.226
77.685
77.367
77.050
47.542
44.578
44.481
38.231
24.968
19.261
180
170
160
150
140
130
120
110
100
90
80
70
60
50
40
30
20 ppm

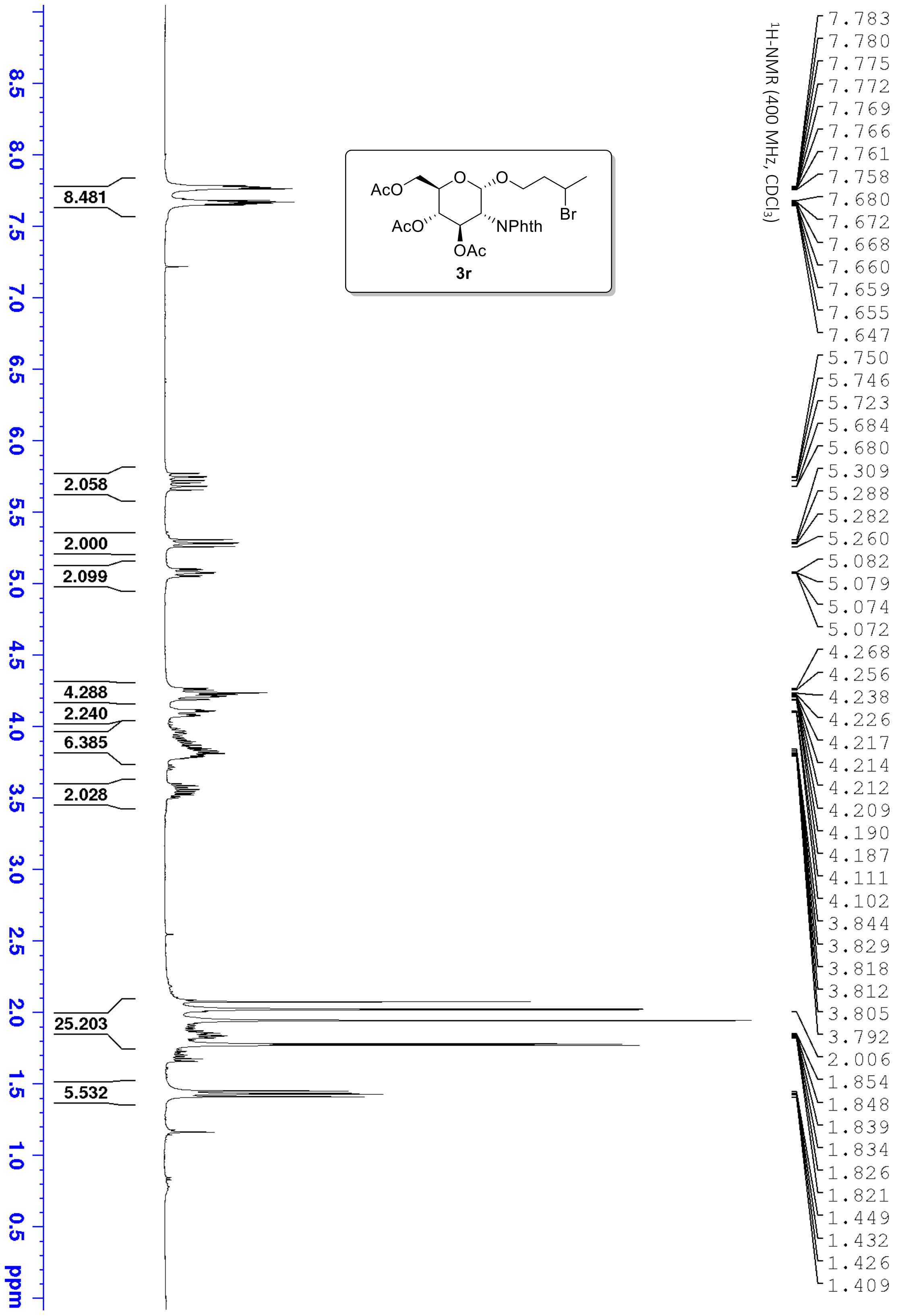
1H-NMR (400 MHz, CDCl3)
AcO
AcO
OAc
NPhth
Br
O
3r
7.783
7.780
7.775
7.772
7.769
7.766
7.761
7.758
7.680
7.672
7.668
7.660
7.659
7.655
7.647
5.750
5.746
5.723
5.684
5.680
5.309
5.288
5.282
5.260
5.082
5.079
5.074
5.072
4.268
4.256
4.238
4.226
4.217
4.214
4.212
4.209
4.190
4.187
4.111
4.102
3.844
3.829
3.818
3.812
3.805
3.792
2.006
1.854
1.848
1.839
1.834
1.826
1.821
1.449
1.432
1.426
1.409
8.481
2.058
2.000
2.099
4.288
2.240
6.385
2.028
25.203
5.532
8.5
8.0
7.5
7.0
6.5
6.0
5.5
5.0
4.5
4.0
3.5
3.0
2.5
2.0
1.5
1.0
0.5
ppm

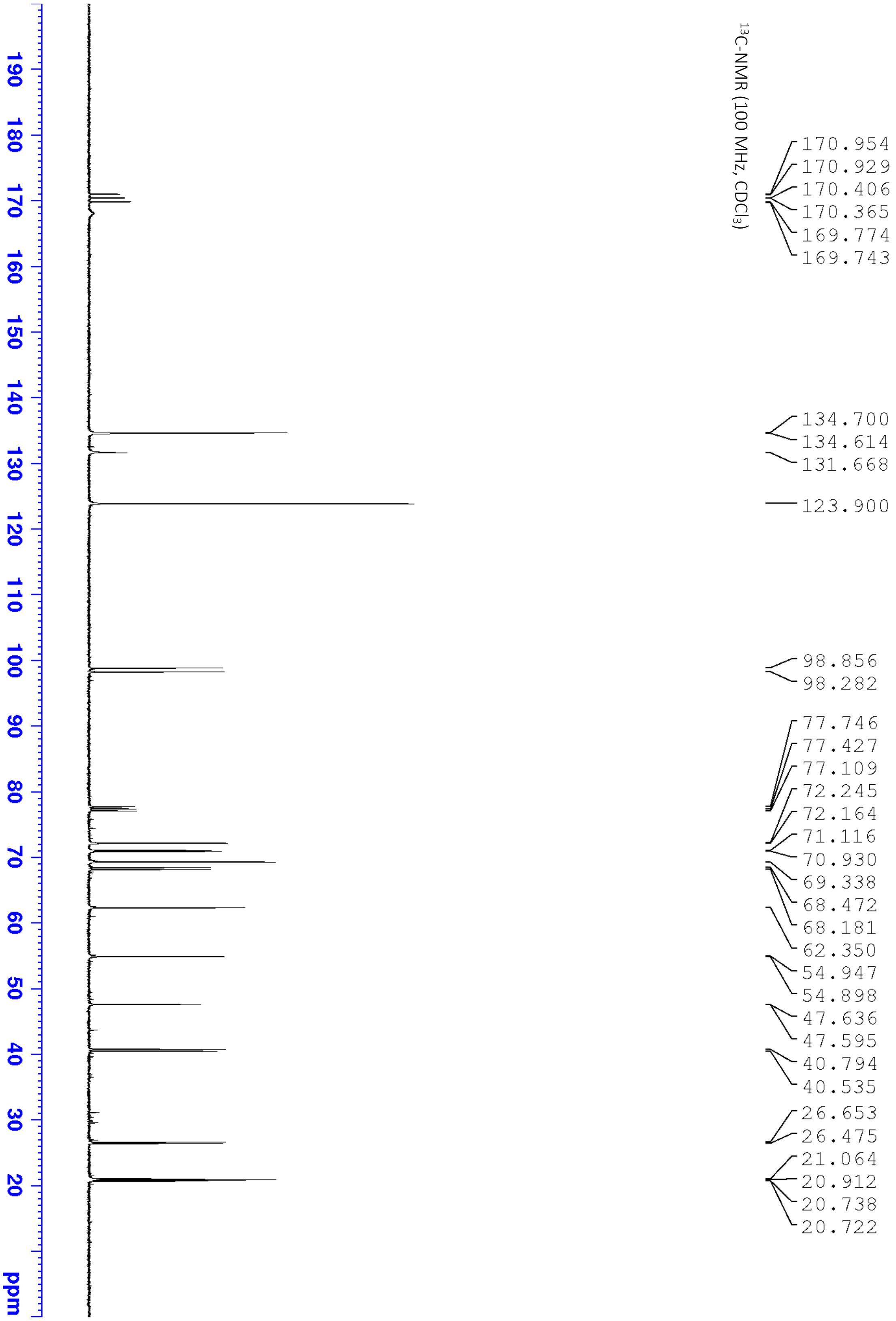

13C-NMR (100 MHz, CDCl3)
170.954
170.929
170.406
170.365
169.774
169.743
134.700
134.614
131.668
123.900
98.856
98.282
77.746
77.427
77.109
72.245
72.164
71.116
70.930
69.338
68.472
68.181
62.350
54.947
54.898
47.636
47.595
40.794
40.535
26.653
26.475
21.064
20.912
20.738
20.722
ppm

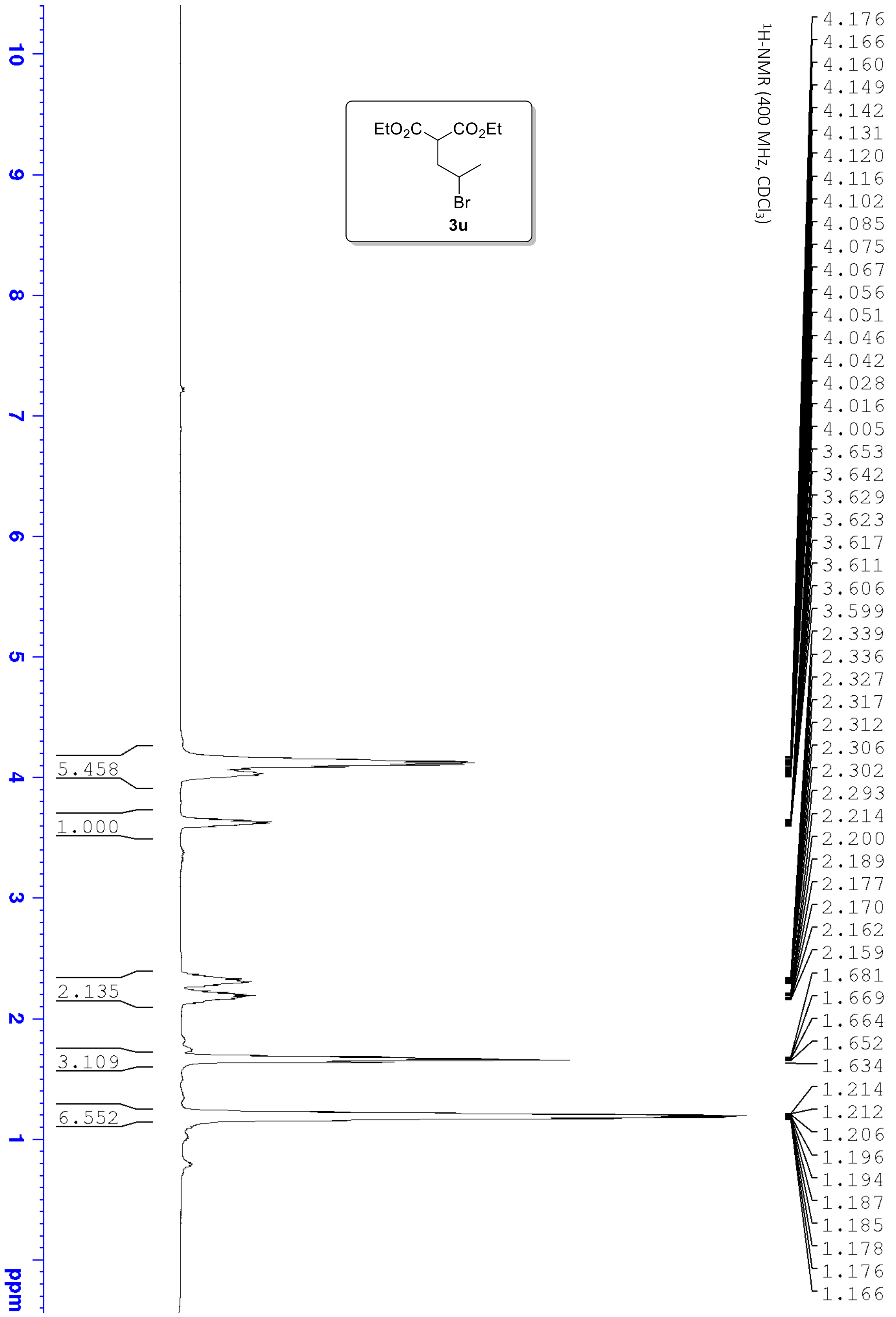

1H-NMR (400 MHz, CDCl3)
EtO2C CO2Et
Br
3u
4.176
4.166
4.160
4.149
4.142
4.131
4.120
4.116
4.102
4.085
4.075
4.067
4.056
4.051
4.046
4.042
4.028
4.016
4.005
3.653
3.642
3.629
3.623
3.617
3.611
3.606
3.599
2.339
2.336
2.327
2.317
2.312
2.306
2.302
2.293
2.214
2.200
2.189
2.177
2.170
2.162
2.159
1.681
1.669
1.664
1.652
1.634
1.214
1.212
1.206
1.196
1.194
1.187
1.185
1.178
1.176
1.166
5.458
1.000
2.135
3.109
6.552
10
9
8
7
6
5
4
3
2
1
ppm

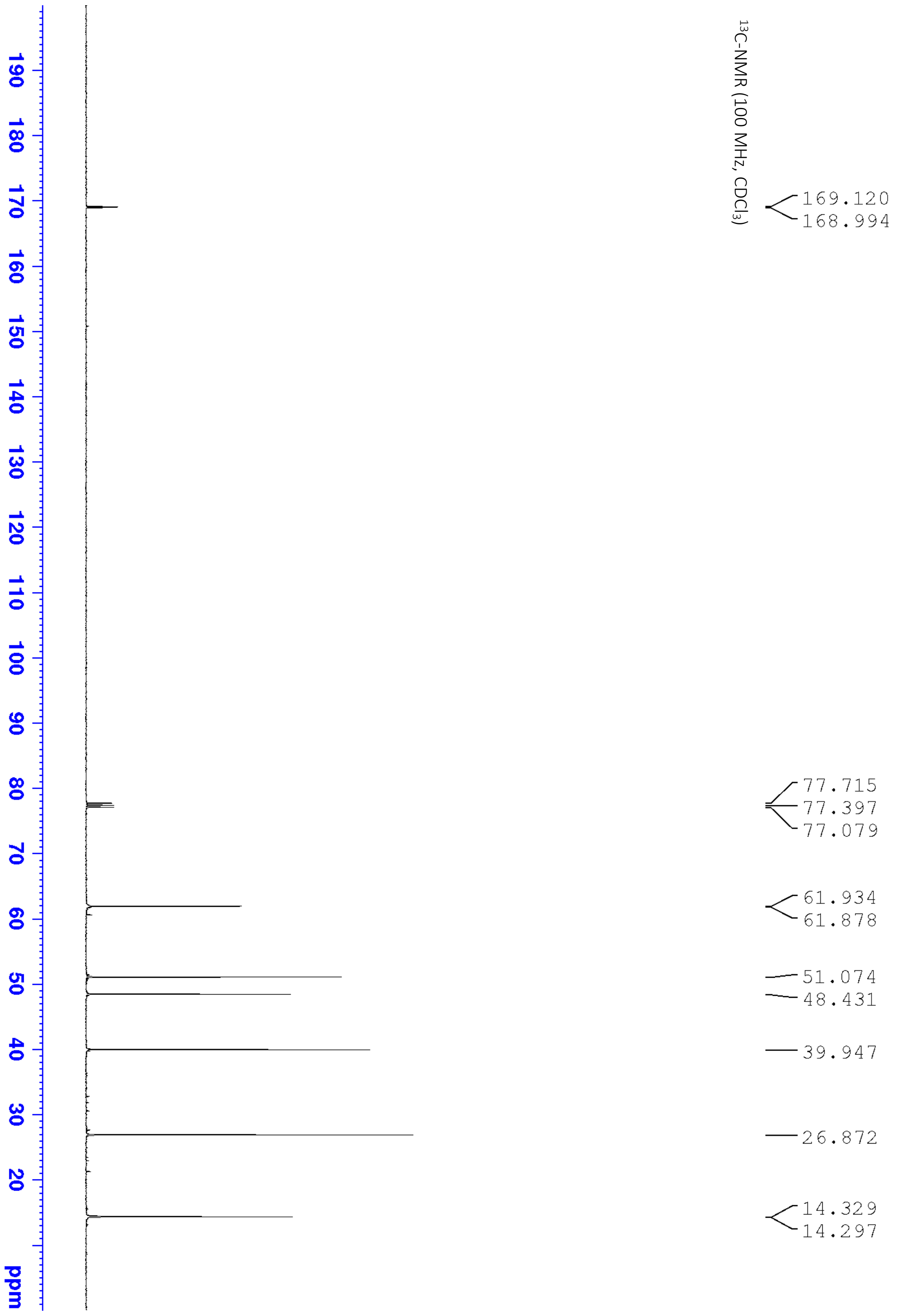

$^{13}$C-NMR (100 MHz, $CDCl_3$)
169.120
168.994
77.715
77.397
77.079
61.934
61.878
51.074
48.431
39.947
26.872
14.329
14.297
ppm

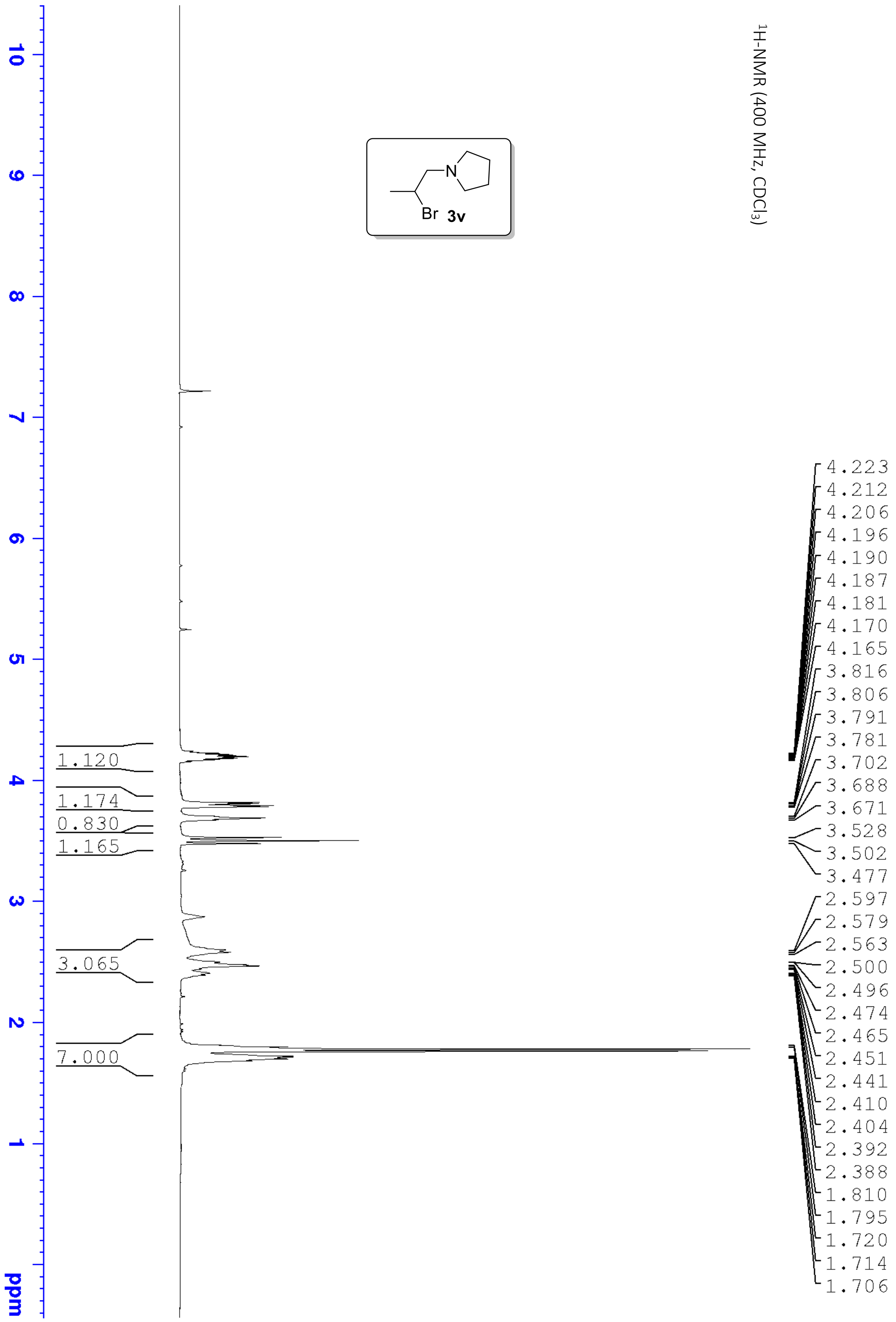
1H-NMR (400 MHz, CDCl3)
N
Br
3v
4.223
4.212
4.206
4.196
4.190
4.187
4.181
4.170
4.165
3.816
3.806
3.791
3.781
3.702
3.688
3.671
3.528
3.502
3.477
2.597
2.579
2.563
2.500
2.496
2.474
2.465
2.451
2.441
2.410
2.404
2.392
2.388
1.810
1.795
1.720
1.714
1.706
1.120
1.174
0.830
1.165
3.065
7.000
10
9
8
7
6
5
4
3
2
1
ppm

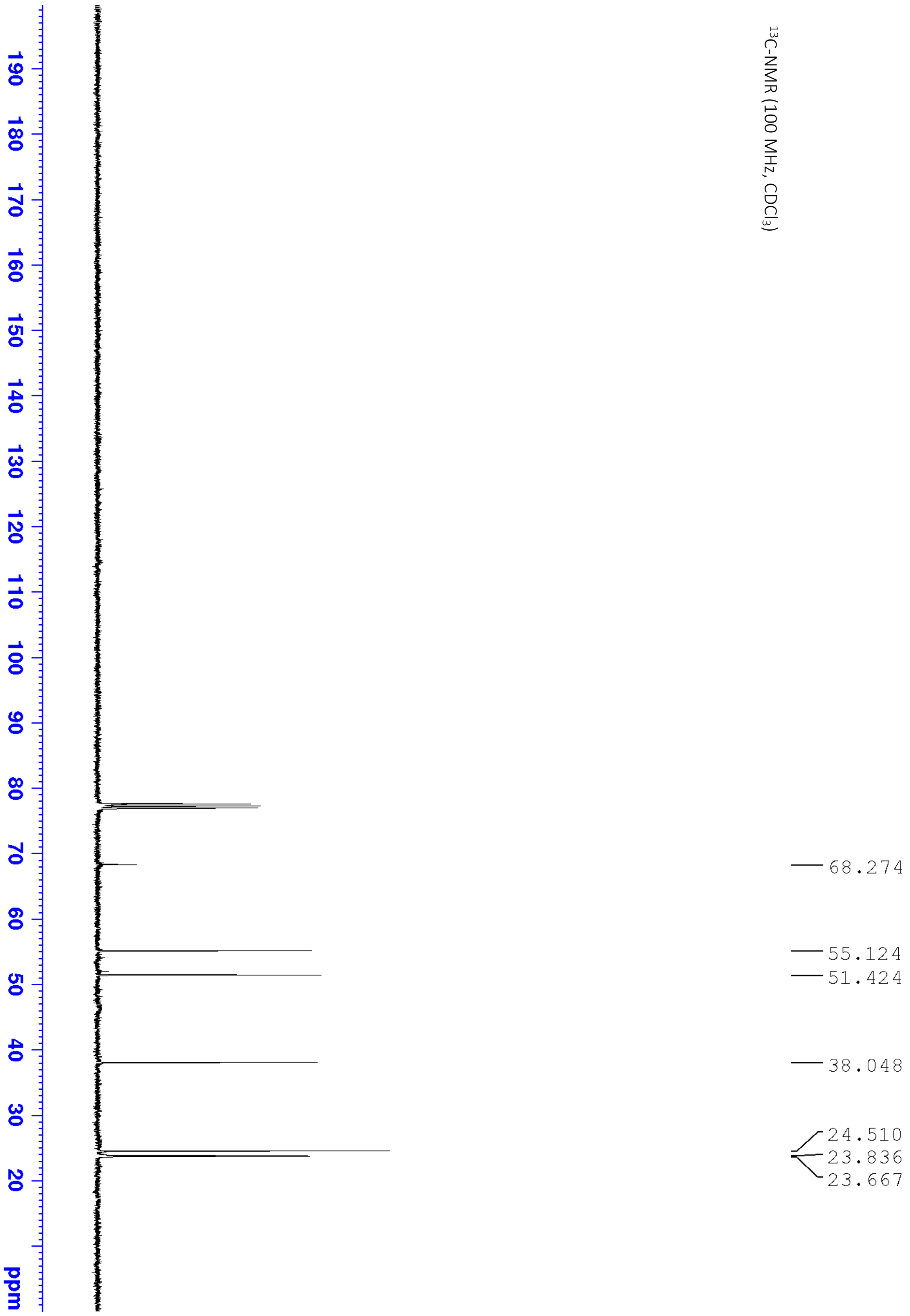

13C-NMR (100 MHz, CDCl3)
68.274
55.124
51.424
38.048
24.510
23.836
23.667
ppm

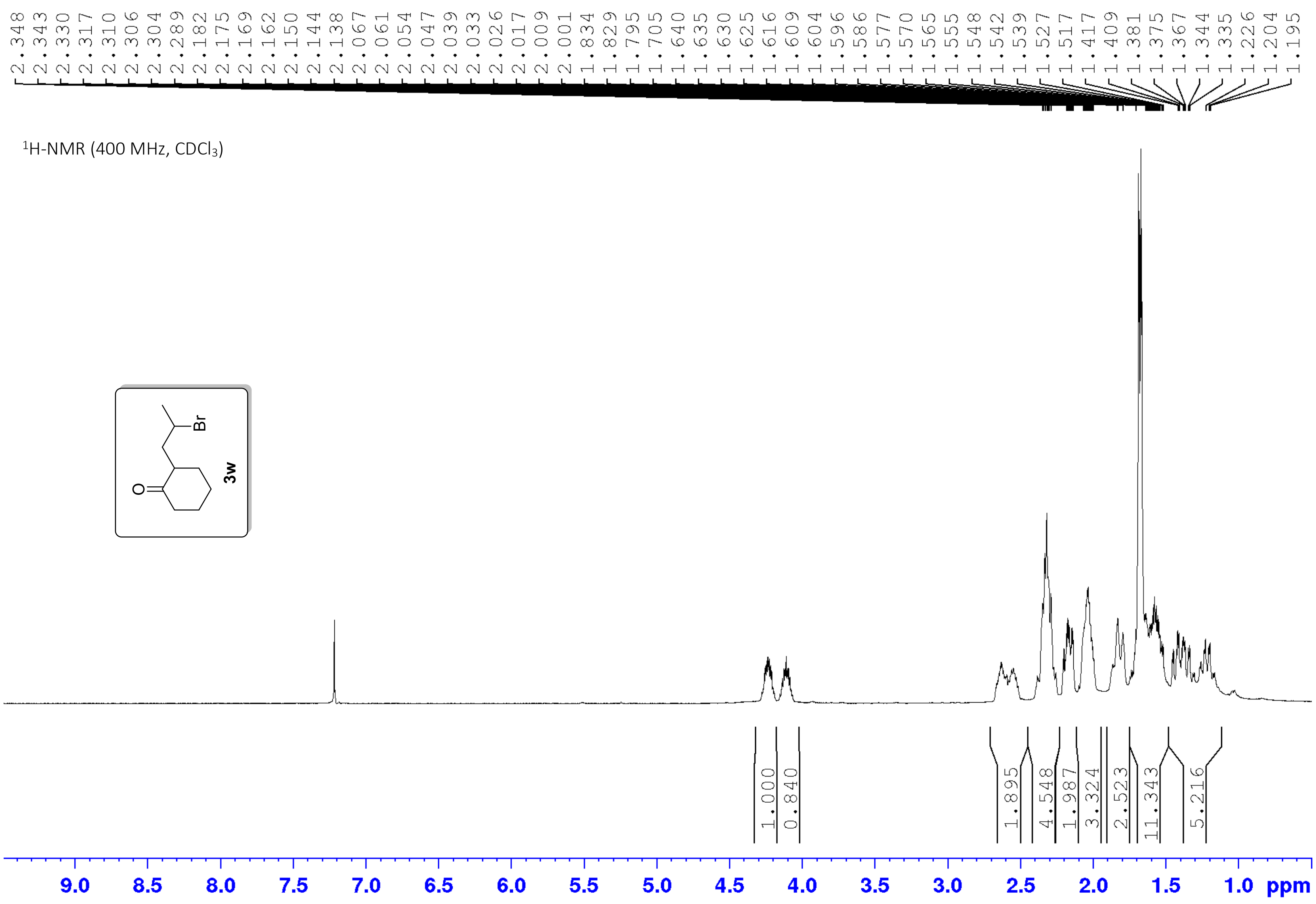
2.348
2.343
2.330
2.317
2.310
2.306
2.304
2.289
2.182
2.175
2.169
2.162
2.150
2.144
2.138
2.067
2.061
2.054
2.047
2.039
2.033
2.026
2.017
2.009
2.001
1.834
1.829
1.795
1.705
1.640
1.635
1.630
1.625
1.616
1.609
1.604
1.596
1.586
1.577
1.570
1.565
1.555
1.548
1.542
1.539
1.527
1.517
1.417
1.409
1.381
1.375
1.367
1.344
1.335
1.226
1.204
1.195
$^1$H-NMR (400 MHz, $CDCl_3$)
O
Br
3w
1.000
0.840
1.895
4.548
1.987
3.324
2.523
11.343
5.216
9.0
8.5
8.0
7.5
7.0
6.5
6.0
5.5
5.0
4.5
4.0
3.5
3.0
2.5
2.0
1.5
1.0
ppm

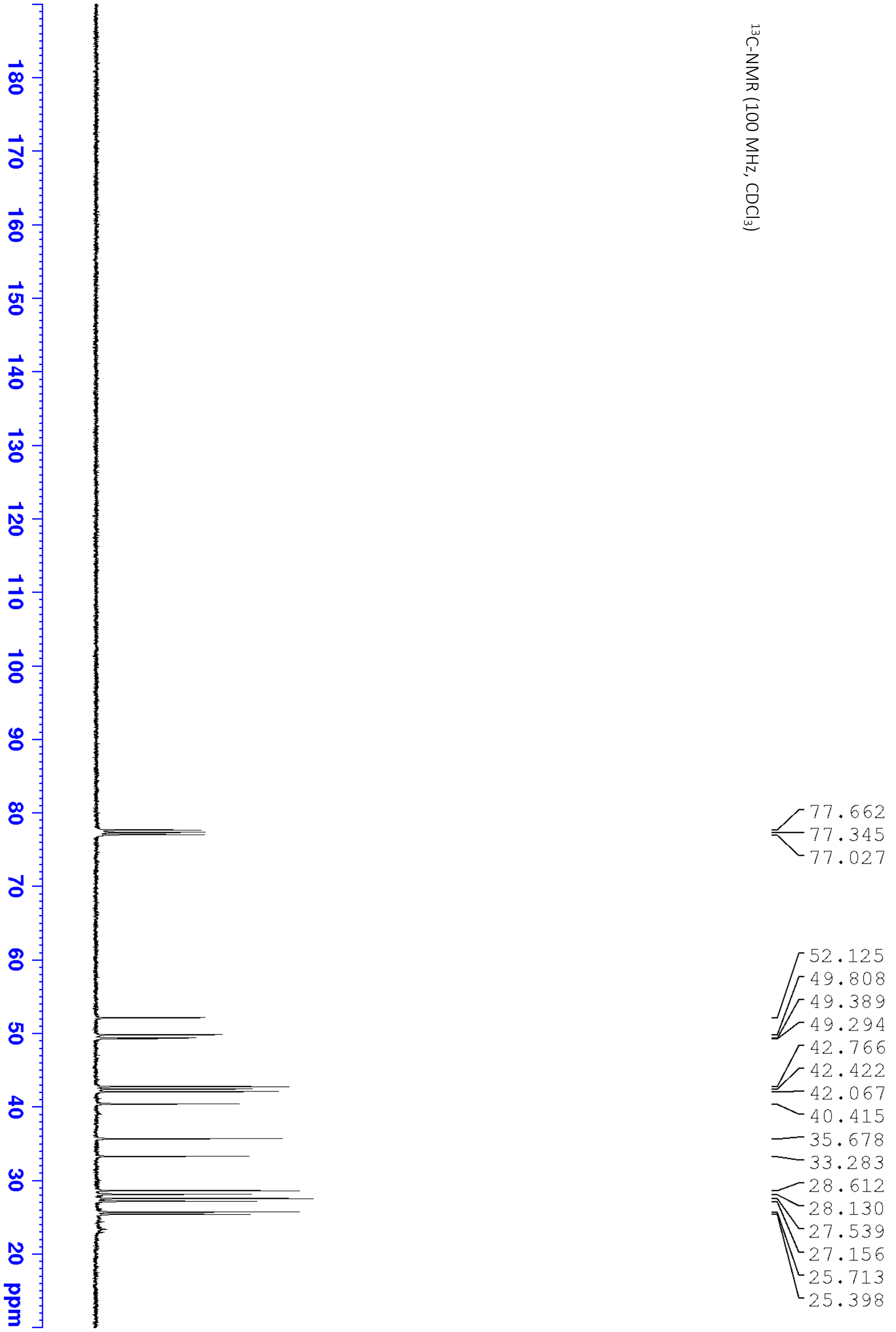
13C-NMR (100 MHz, CDCl3)
77.662
77.345
77.027
52.125
49.808
49.389
49.294
42.766
42.422
42.067
40.415
35.678
33.283
28.612
28.130
27.539
27.156
25.713
25.398
180
170
160
150
140
130
120
110
100
90
80
70
60
50
40
30
20
ppm

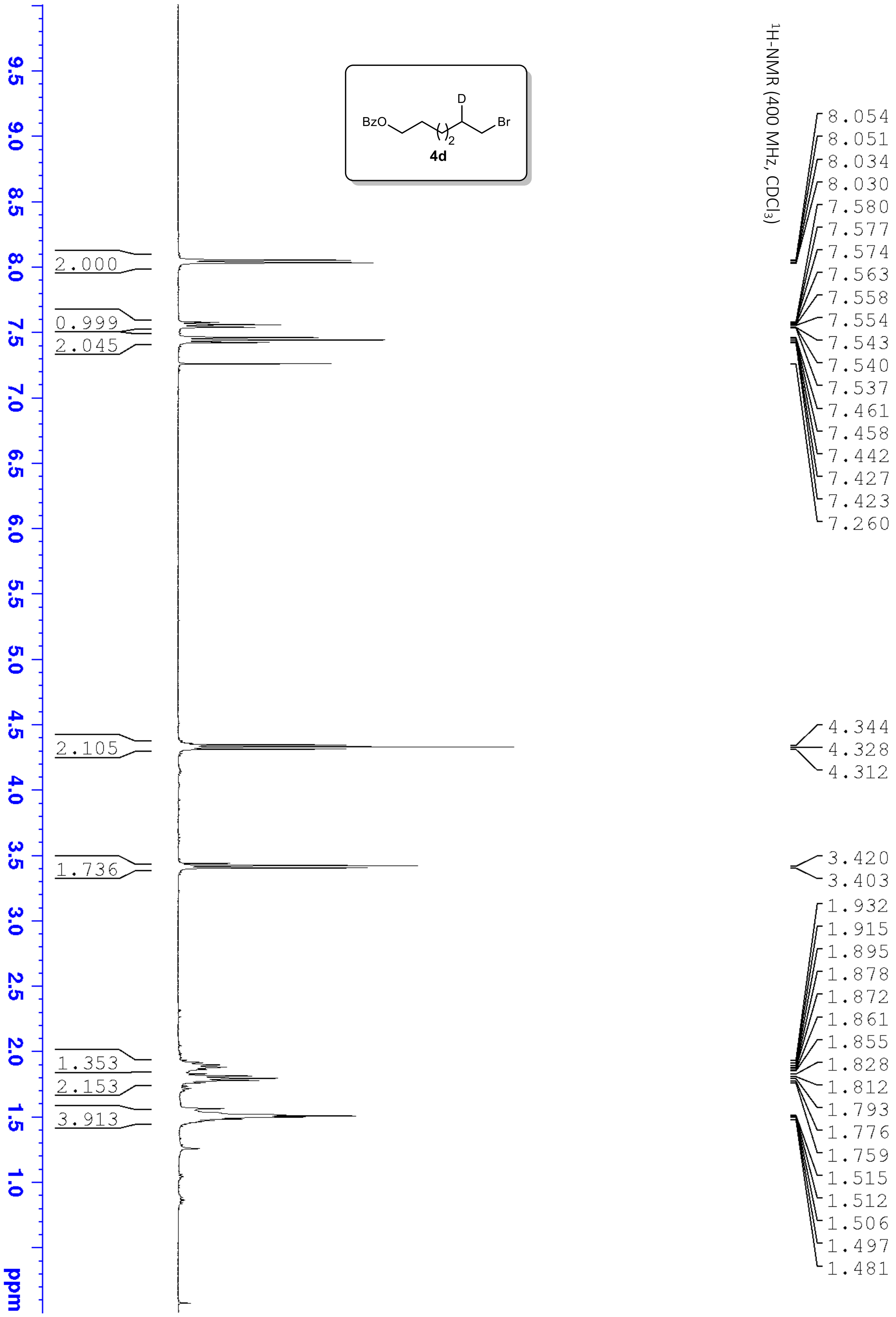
1H-NMR (400 MHz, CDCl3)
BzO
D
Br
2
4d
8.054
8.051
8.034
8.030
7.580
7.577
7.574
7.563
7.558
7.554
7.543
7.540
7.537
7.461
7.458
7.442
7.427
7.423
7.260
4.344
4.328
4.312
3.420
3.403
1.932
1.915
1.895
1.878
1.872
1.861
1.855
1.828
1.812
1.793
1.776
1.759
1.515
1.512
1.506
1.497
1.481
2.000
0.999
2.045
2.105
1.736
1.353
2.153
3.913
ppm

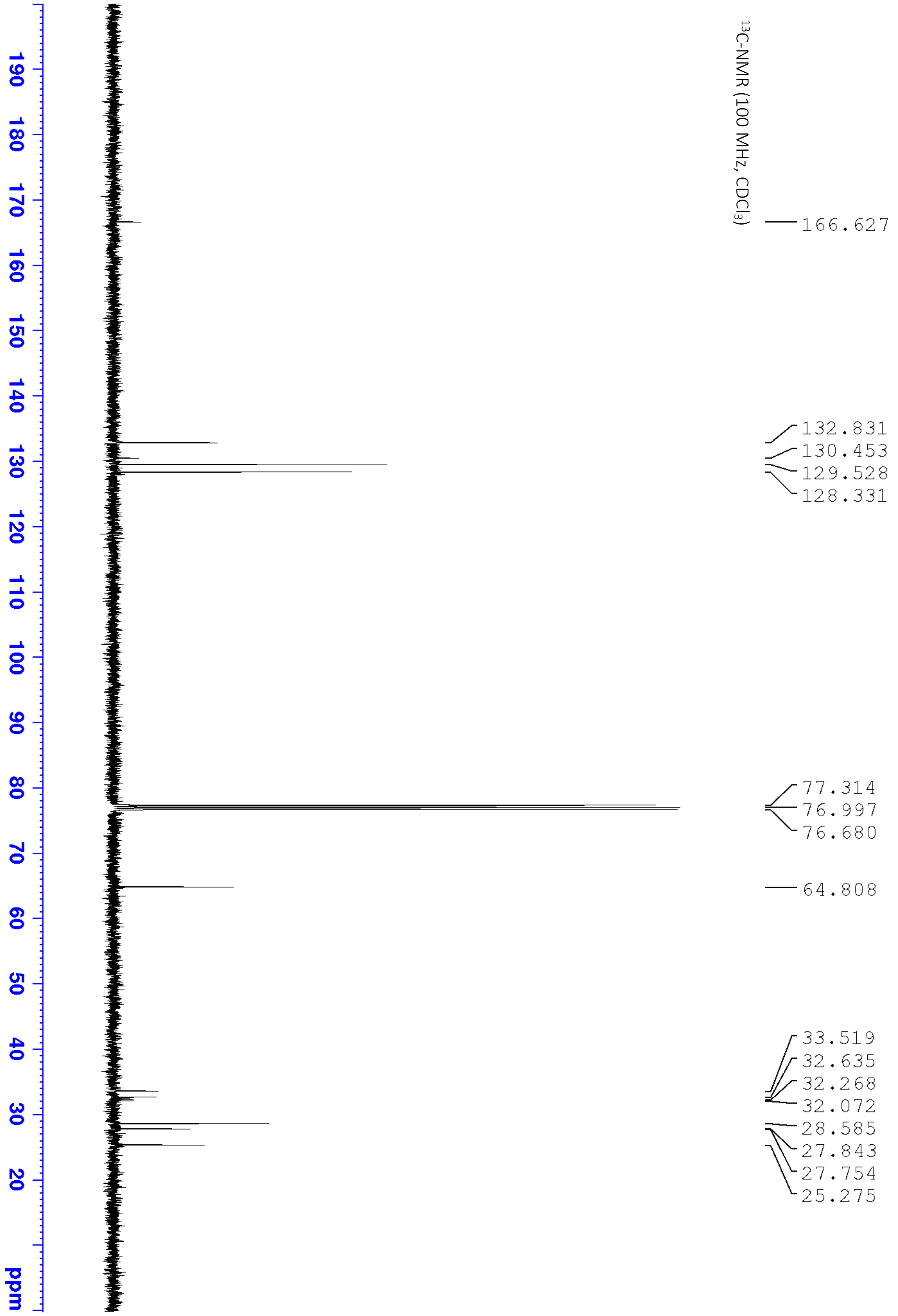
13C-NMR (100 MHz, CDCl3)
166.627
132.831
130.453
129.528
128.331
77.314
76.997
76.680
64.808
33.519
32.635
32.268
32.072
28.585
27.843
27.754
25.275
190
180
170
160
150
140
130
120
110
100
90
80
70
60
50
40
30
20
ppm

# References


[1] S. Hong, Y.-M. Lee, K. Ray, W. Nam, *Coord. Chem. Rev.* **2017**, *334*, 25–42.

[2] B. Walter, **1922**.

[3] B. Walter, **1925**.

[4] M. L. Sherrill, K. E. Mayer, G. F. Walter, *J. Am. Chem. Soc.* **1934**, *56*, 926–930.

[5] M. S. Kharasch, J. A. Norton, F. R. Mayo, *J. Org. Chem.* **1938**, *3*, 48–54.

[6] M. S. Kharasch, F. R. Mayo, *J. Am. Chem. Soc.* **1933**, *55*, 2468–2496.

[7] J. C. Smith, *J. Soc. Chem. Ind.* **1938**, *57*, 461–466.

[8] J. C. Smith, *J. Soc. Chem. Ind.* **1937**, *56*, 833–839.

[9] J. C. Smith, P. L. Harris, *Nature* **1935**, *135*, 187.

[10] Y. Urushibara, O. Simamura, *Bull. Chem. Soc. Jpn.* **1939**, *14*, 323–336.

[11] M. Takebayashi, *Bull. Chem. Soc. Jpn.* **1940**, *15*, 116–118.

[12] W. E. Vaughan, F. F. Rust, T. W. Evans, *J. Org. Chem.* **1942**, *7*, 477–490.

[13] D. Braun, *Int. J. Polym. Sci.* **2009**, *2009*, 893234.

[14] H. J. Hageman, *Prog. Org. Coatings* **1985**, *13*, 123–150.

[15] W. T. Borden, R. Hoffmann, T. Stuyver, B. Chen, *J. Am. Chem. Soc.* **2017**, *139*, 9010–9018.

[16] X. Chen, X. Li, X.-L. Chen, L.-B. Qu, J.-Y. Chen, K. Sun, Z.-D. Liu, W.-Z. Bi, Y.-Y. Xia, H.-T. Wu, et al., *Chem. Commun.* **2015**, *51*, 3846–3849.

[17] S. Acevedo, J. M. Cordero T., H. Carrier, B. Bouyssiere, R. Lobinski, *Energy & Fuels* **2009**, *23*, 842–848.

[18] C. M. Hansen, A. L. Smith, *Carbon N. Y.* **2004**, *42*, 1591–1597.

[19] C. M. Hansen, *Hansen Solubility Parameters: A User's Handbook*, CRC Press, **2007**.

[20] T. Sato, Y. Hamada, M. Sumikawa, S. Araki, H. Yamamoto, *Ind. Eng. Chem. Res.* **2014**, *53*, 19331–19337.

[21] F. R. Mayo, C. Walling, *Chem. Rev.* **1940**, *27*, 351–412.

[22] J. Durkee, *Cleaning with Solvents: Science and Technology*, William Andrew, **2013**.

[23] R. J. Cvetanović, *Can. J. Chem.* **1958**, *36*, 623–634.

[24] H. J. Lucas, A. N. Prater, R. E. Morris, *J. Am. Chem. Soc.* **1935**, *57*, 723–727.

[25] S. C. Moldoveanu, *Pyrolysis of Organic Molecules: Applications to Health and Environmental Issues*, Elsevier, **2009**.

[26] R. D. Bach, P. Y. Ayala, H. B. Schlegel, *J. Am. Chem. Soc.* **1996**, *118*, 12758–12765.

[27] J. Lalevée, J. P. Fouassier, *Encycl. Radicals Chem. Biol. Mater.* **2012**, DOI doi:10.1002/9781119953678.rad003.

[28] M. Mishra, Y. Yagci, *Handbook of Radical Vinyl Polymerization*, CRC Press, **1998**.

[29] M. S. Kharasch, W. R. Haefele, F. R. Mayo, *J. Am. Chem. Soc.* **1940**, *62*, 2047–2051.

[30] Y. Urushibara, M. Takebayashl, *Bull. Chem. Soc. Jpn.* **1937**, *12*, 51–54.

[31] H. Sakurai, K. Sasaki, A. Hosomi, *Tetrahedron Lett.* **1980**, *21*, 2329–2332.

[32] H. Clavier, S. P. Nolan, M. Mauduit, *Organometallics* **2008**, *27*, 2287–2292.

[33] K. D. Schleicher, T. F. Jamison, *Org. Lett.* **2007**, *9*, 875–878.

[34] L. Zhang, Dolbier W. R., B. Sheeller, K. U. Ingold, *J. Am. Chem. Soc.* **2002**, *124*, 6362–6366.

[35] G. Rong, D. Liu, L. Lu, H. Yan, Y. Zheng, J. Chen, J. Mao, *Tetrahedron* **2014**, *70*, 5033–5037.

[36] D. De Keukeleire, S.-L. He, D. Blakemore, A. Gilbert, *J. Photochem. Photobiol. A Chem.* **1994**, *80*, 233–240.

[37] X. Li, J. Du, Y. Zhang, H. Chang, W. Gao, W. Wei, *Org. Biomol. Chem.* **2019**, *17*, 3048–3055.

[38] P. P. Singh, M. M. Gharia, F. Dasgupta, H. C. Srivastava, *Tetrahedron Lett.* **1977**, *18*, 439–440.

[39] M. Fosso, M. N. AlFindee, Q. Zhang, V. de P. N. Nziko, Y. Kawasaki, S. K. Shrestha, J. Bearss, R. Gregory, J. Y. Takemoto, C.-W. T. Chang, *J. Org. Chem.* **2015**, *80*, 4398–4411.

[40] S. Yan, D. Klemm, *Tetrahedron* **2002**, *58*, 10065–10071.

[41] G. V. M. Sharma, K. S. Rao, R. Ravi, K. Narsimulu, P. Nagendar, C. Chandramouli, S. K. Kumar, A. C. Kunwar, *Chem. – An Asian J.* **2009**, *4*, 181–193.

[42] F. G. Calvo-Flores, P. García-Mendoza, F. Hernández-Mateo, J. Isac-García, F. Santoyo-González, *J. Org. Chem.* **1997**, *62*, 3944–3961.

[43] F. García-Tellado, P. de Armas, J. J. Marrero-Tellado, *Angew. Chemie* **2000**, *112*, 2839–2841.

[44] J. Gui, H. Xie, H. Jiang, W. Zeng, *Org. Lett.* **2019**, *21*, 2804–2807.

[45] H. Dang, N. Cox, G. Lalic, *Angew. Chemie Int. Ed.* **2014**, *53*, 752–756.

[46] X. Yu, T. Yang, S. Wang, H. Xu, H. Gong, *Org. Lett.* **2011**, *13*, 2138–2141.

[47] P. A. Zoretic, C. Bhakta, N. D. Sinha, J. Jardin, *J. Heterocycl. Chem.* **1981**, *18*, 1643–1644.

[48] Y. Nishio, R. Mifune, T. Sato, S. Ishikawa, H. Matsubara, *Tetrahedron Lett.* **2017**, *58*, 1190–1193.

[49] P. E. Dietze, W. P. Jencks, *J. Am. Chem. Soc.* **1986**, *108*, 4549–4555.

[50] B. Liu, B. S. Gaylord, S. Wang, G. C. Bazan, *J. Am. Chem. Soc.* **2003**, *125*, 6705–6714.

[51] M. Thompson, C. Carkner, N. J. Mosey, N. Kapernaum, R. P. Lemieux, *Soft Matter* **2015**, *11*, 3860–3868.

[52] P. Wang, X. Yan, F. Huang, *Chem. Commun.* **2014**, *50*, 5017–5019.

[53] A. S. Dudnik, G. C. Fu, *J. Am. Chem. Soc.* **2012**, *134*, 10693–10697.

[54] M. S. Khan, L. N. Owen, *J. Chem. Soc. Perkin Trans. 1* **1972**, 2060–2067.

[55] T. E. Boothe, J. L. Greene Jr, P. B. Shevlin, M. R. Willcott III, R. R. Inners, A. Cornelis, *J. Am. Chem. Soc.* **1978**, *100*, 3874–3879.

[56] Gaussian 09, Revision D.01, M. J. Frisch, G. W. Trucks, H. B. Schlegel, G. E. Scuseria, M. A. Robb, J. R. Cheeseman, G. Scalmani, V. Barone, B. Mennucci, G. A. Petersson, H. Nakatsuji, M. Caricato, X. Li, H. P. Hratchian, A. F. Izmaylov, J. Bloino, G. Zheng, J. L. Sonnenberg, M. Hada, M. Ehara,

K. Toyota, R. Fukuda, J. Hasegawa, M. Ishida, T. Nakajima, Y. Honda, O. Kitao, H. Nakai, T. Vreven, J. A. Montgomery, Jr., J. E. Peralta, F. Ogliaro, M. Bearpark, J. J. Heyd, E. Brothers, K. N. Kudin, V. N. Staroverov, R. Kobayashi, J. Normand, K. Raghavachari, A. Rendell, J. C. Burant, S. S. Iyengar, J. Tomasi, M. Cossi, N. Rega, J. M. Millam, M. Klene, J. E. Knox, J. B. Cross, V. Bakken, C. Adamo, J. Jaramillo, R. Gomperts, R. E. Stratmann, O. Yazyev, A. J. Austin, R. Cammi, C. Pomelli, J. W. Ochterski, R. L. Martin, K. Morokuma, V. G. Zakrzewski, G. A. Voth, P. Salvador, J. J. Dannenberg, S. Dapprich, A. D. Daniels, Ö. Farkas, J. B. Foresman, J. V. Ortiz, J. Cioslowski, and D. J. Fox, Gaussian, Inc., Wallingford CT, 2009**.**

[57] A. D. Becke, *J. Chem. Phys.* **1993**, *98*, 5648–5652.

[58] C. Lee, W. Yang, R. G. Parr, *Phys. Rev. B* **1988**, *37*, 785–789.

[59] S. H. Vosko, L. Wilk, M. Nusair, *Can. J. Phys.* **1980**, *58*, 1200–1211.

[60] S. Grimme, J. Antony, S. Ehrlich, H. Krieg, *J. Chem. Phys.* **2010**, *132*, 154104.

[61] F. Weigend, R. Ahlrichs, *Phys. Chem. Chem. Phys.* **2005**, *7*, 3297–3305.

[62] J. W. McIver Jr, A. Komornicki, *J. Am. Chem. Soc.* **1972**, *94*, 2625–2633.

[63] S. Miertuš, E. Scrocco, J. Tomasi, *Chem. Phys.* **1981**, *55*, 117–129.

[64] J. L. Pascual-ahuir, E. Silla, I. Tuñon, *J. Comput. Chem.* **1994**, *15*, 1127–1138.

[65] V. Barone, M. Cossi, *J. Phys. Chem. A* **1998**, *102*, 1995–2001.

[66] NBO 6.0. E. D. Glendening, J. K. Badenhoop, A. E. Reed, J. E. Carpenter, J. A. Bohmann, C. M. Morales, C. R. Landis, F. Weinhold (Theoretical Chemistry Institute, University of Wisconsin, Madison, WI, 2013); http://nbo6.chem.wisc.edu/

# Author Contributions

D.A.C. tuned reactions conditions and performed experiments. V.S., P.A. and H. S. S. prepared and purify substrates. I. F performed theoretical calculations. V.S.M. coordinated the experimental and theoretical results. P.O.M. and J.I.P. designed and conceived experiments.
